# Enantioselective hydrogen atom relay via non-covalent catalyst assembly

**DOI:** 10.1038/s41586-026-10692-4

**Published:** 2026-06-01

**Authors:** Navadheer Yalamanchili, Jules Hugo Alexandre, Robert L. Anderson, Giuseppe Zuccarello

**Affiliations:** Laboratory of Asymmetric Catalysis and Synthesis, Institute of Chemical Sciences and Engineering, Ecole Polytechnique Fédérale de Lausanne, Lausanne, Switzerland

**Keywords:** Synthetic chemistry methodology, Asymmetric catalysis, Stereochemistry

## Abstract

Most biological functions are regulated by chiral molecules^1^ that contain at least one tertiary stereogenic carbon, that is, a carbon with one C(*sp*^3^)–H bond. Hydrogen atom transfer (HAT)^2^ is a straightforward strategy that can be used to either edit^3^ or introduce tertiary stereocentres in multiple synthetically useful transformations^4^, especially when coupled with photoredox catalysis^5,6^. However, traditional de novo design of chiral HAT catalysts that provide sufficient enantiocontrol over short-lived open-shell intermediates^7^ has represented a major hurdle in the development of enantioselective HAT reactions. Here we describe a distinct approach in which chiral HAT catalysts are obtained in situ by non-covalent self-assembly of privileged chiral phosphoric acids and commercial 2-mercaptopyridines. The phosphoric acid serves as a modular interchangeable chiral element that renders the achiral thiol effectively chiral, thereby allowing access to a previously inaccessible combinatorial space of chiral HAT catalysts. This platform enabled the photochemical deracemization of 2-aryl pyrrolidines, which are prevalent scaffolds in active pharmaceutical ingredients. Optical enrichment occurs by means of enantioselective hydrogen atom relay, in which a single chiral assembly orchestrates hydrogen atom abstraction and delivery. This conceptual approach of relaying chiral information through non-covalent assembly paves the way for discovery of numerous asymmetric radical transformations.

## Abstract (web summary)

Chiral catalysts for hydrogen atom transfer are produced through a self-assembly mechanism that enables access to novel catalyst permutations, with applications including photochemical deracemization of 2-aryl pyrrolidines for use as pharmaceutical scaffolds.

## Main

**Fig. 1:**
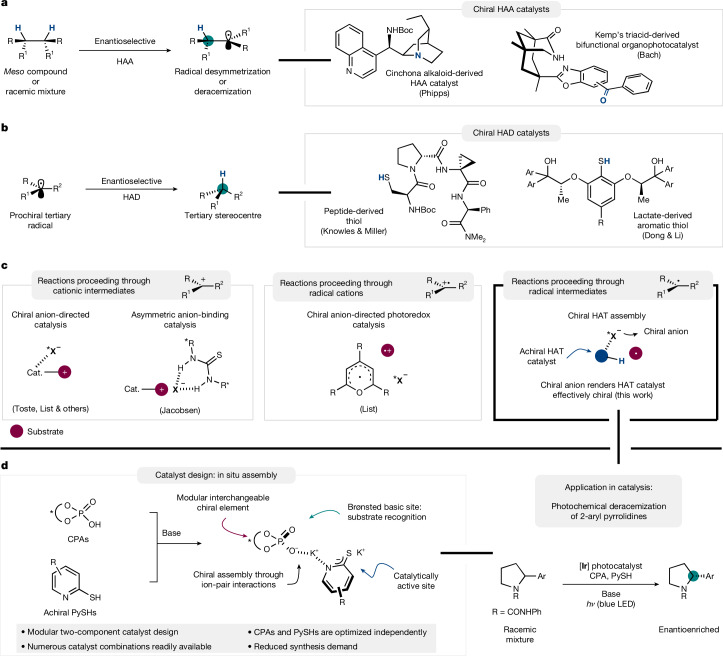
Enantioselective HAT. **a**, Enantioselective HAA. **b**, Enantioselective HAD. **c**, Use of chiral anions to exert enantiocontrol over reactions proceeding through cationic intermediates and radical cations. **d**, This work: molecular self-assembly of chiral HAT catalysts for photochemical deracemization of 2-aryl pyrrolidines. Asterisk indicates chirality. *R, chiral substituent; Ar, aryl; cat., achiral catalyst; *X^−^; chiral anion; X^−^: achiral anion.

Enantioselectivity in hydrogen atom transfer (HAT) processes^8,9^ can be achieved in two distinct elemental steps. First, a chiral hydrogen atom abstractor can induce chirality by desymmetrization of *meso* compounds or selective hydrogen atom abstraction (HAA) at one enantiomer of a racemic mixture (Fig. 1a). Recent groundbreaking work by Phipps and colleagues^10^used cinchona alkaloid-derived catalysts to promote desymmetrization of *meso*-diols through enantioselective HAA under the action of photoredox catalysis. Furthermore, Bach established that bifunctional hydrogen-bonding benzophenone organophotocatalysts^11^ derived from Kemp’s triacid could mediate photochemical deracemizations through reversible HAT, including selective HAA at one enantiomer followed by unselective back-HAT. Alternatively, hydrogen atom delivery (HAD) to a prochiral carbon-centred radical introduces a tertiary stereocentre through C(*sp*^3^)–H bond formation (Fig. 1b). Seminal work by Hyster has used enzymes with nicotinamide-^12^ and flavin-dependent^13^ cofactors to promote enantioselective HAD. De novo designed chiral organic thiols are also emerging as HAD catalysts: Knowles and Miller^14^ implemented small-molecule tetrapeptides containing a cysteine residue, and Dong designed *C*_2_-symmetric lactate-derived aromatic thiols^15^. However, the limited number of existing chiral HAT catalysts engage in only one elemental step enantioselectively (either HAA or HAD). Knowles and Miller achieved photochemical deracemization of cyclic ureas through independent sequential enantioselective deprotonation followed by stereoselective HAD using two chiral catalysts^14^. Conceptually, a single chiral HAT catalyst that controls enantioselectivity in both HAA and HAD could offer a general platform to solve shortcomings across diverse asymmetric radical transformations.

We evaluated a chiral HAT catalyst design that builds on electron-deficient organic thiols, as these can undergo HAD, and their corresponding thiyl radicals^16,17^ obtained upon single-electron oxidation of the thiolate^18,19^ promote HAA. Wendlandt recently leveraged an achiral silanethiol catalyst mediating both HAA and HAD in catalytic isomerization of *cis-*1,2-diols^20^. Although chiral thiols have been successfully used for enantioselective HAD, their corresponding thiyl radicals have not been used for enantioselective HAA. However, the design of chiral organic thiols has proven synthetically difficult^21^. Moreover, we reasoned that building a covalent chiral environment around the active site of the catalyst would mean traditional, iterative and linear reaction development, as multiple catalyst modifications would need to be individually synthesized and tested systematically.

As an alternative approach, we considered the use of chiral anions to render an achiral catalyst effectively chiral through ion-pair interactions^22–24^ (Fig. 1c). Toste^25^ and List^23^ pioneered the use of chiral anions to promote catalytic enantioselective transformations proceeding through cationic intermediates, and Jacobsen^26,27^ developed asymmetric anion-binding catalysis to access chiral cationic catalysts in situ that were otherwise not amenable to asymmetric design (Fig. 1c, left). List recently showed that weakly basic chiral anions could be paired with cationic photocatalysts to promote asymmetric transformations proceeding through radical cations^28–30^ (Fig. 1c, middle). However, the underlying principles of these conceptual catalyst designs have only been suitable for transformations operating through ionic intermediates; they have not been applied to induce enantioselectivity in reactions proceeding through neutral radical intermediates. We proposed that pairing chiral anions with achiral HAT catalysts could overcome the inherent difficulty of rendering HAT catalysts effectively chiral and induce enantioselectivity in HAT processes proceeding through neutral open-shell intermediates (Fig. 1c, right).

In this work, we present a chiral HAT catalyst that assembles in situ, pairing phosphate ions derived from privileged^31^ chiral phosphoric acids (CPAs) with commercial 2-mercaptopyridines (PySHs) (Fig. 1d). In our two-component catalyst design, the phosphate ion^32^ serves as a modularly interchangeable chiral element and provides a point of substrate fixation in a chiral microenvironment at the phosphoryl oxygen^33^, whereas the PySH unit is responsible for HAT.

Photochemical deracemization^34,35^ is emerging as a powerful strategy to convert a racemic mixture into enantioenriched material. Seminal work has been reported by Bach^11,36^, Knowles and Miller^14^, Luo^37^, Gilmour^38^, Zuo^39^, and Fu and Liu^40^. We evaluated whether our in situ-assembled chiral HAT catalysts could achieve photochemical deracemization of a general molecular scaffold such as 2-aryl pyrrolidines, a commonly used molecular subunit in active pharmaceutical ingredients. Fast reaction optimization was achieved, as the catalytic units forming the chiral assembly were optimized independently, giving ready access to a broad permutation of chiral HAT catalysts in situ while maintaining low synthesis demands. Preliminary mechanistic studies indicate that ion pairing is the dominant non-covalent interaction shaping the chiral assemblies, and that photochemical deracemization occurs through enantioselective hydrogen atom relay.

## Reaction development

**Fig. 2:**
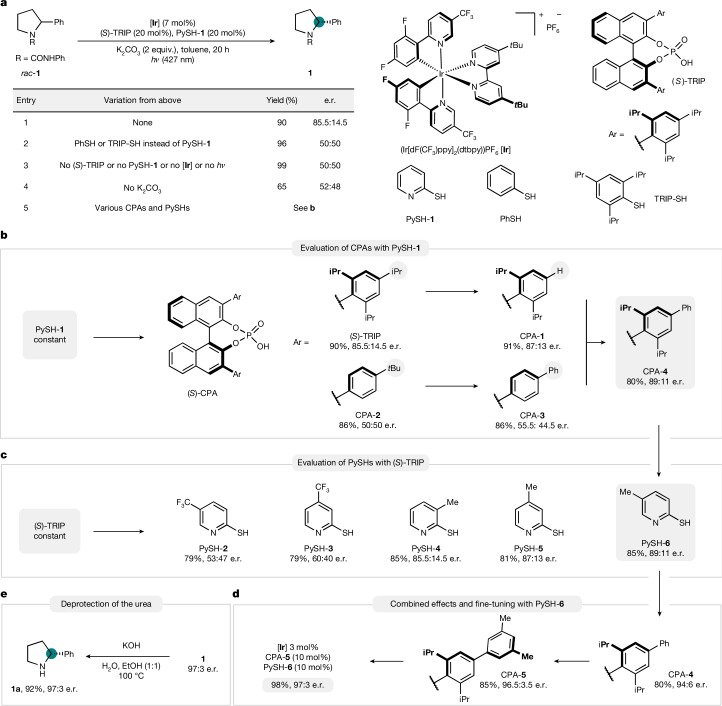
Reaction optimization and protecting group removal. **a**, Reaction discovery and preliminary control experiments. **b**, Independent optimization of CPAs with PySH-**1**. **c**, Evaluation of PySHs with (*S*)-TRIP. **d**, Combined effects and fine-tuning. **e**, Removal of protecting groups. Reactions were performed on a 0.05-mmol scale. Yields were determined by ^1^H nuclear magnetic resonance using 1,3-benzodioxole as the internal standard. e.r. values were determined by high-performance liquid chromatography analysis on a chiral stationary phase. *t*Bu, *tert*-butyl; Me, methyl; Ph, phenyl; iPr, isopropyl.

We selected urea-protected 2-phenylpyrrolidine *rac*-**1** as the model substrate, expecting H-bonding between the urea N–H bond and the phosphoryl oxygen of the chiral phosphate. In the presence of commercially available (Ir[dF(CF_3_)ppy]_2_(dtbpy))PF_6_ [**Ir**] photocatalyst (7 mol%), (*S*)-3,3′-bis(2,4,6-triisopropylphenyl)-1,1′-binaphthyl-2,2′-diyl hydrogenphosphate ((*S*)-TRIP; 20 mol%), PySH-**1** (20 mol%) and K_2_CO_3_ (2 equivalent (equiv.)), under irradiation with blue-light-emitting diodes at room temperature, enantioenriched **1** was recovered with 90% yield and 85.5:14.5 enantiomeric ratio (e.r.) (Fig. 2a, entry 1). Control experiments established that no deracemization occurred when either thiophenol or 2,4,6-triisopropylbenzenethiol (both common HAT catalysts) was used instead of PySH-**1**, or in the absence of either (*S*)-TRIP, PySH-**1**, [**Ir**] or light (Fig. 2a, entries 2 and 3). Performing the reaction without K_2_CO_3_ led to nearly racemic product in lower yield (Fig. 2a, entry 4) (see the Supplementary Information for detailed optimization).

The two-component catalyst design based on spontaneous assembly in solution allows CPAs and PySHs to be optimized independently, enabling rapid reaction optimization. We first surveyed commercial and reported CPAs by varying the 3,3′-substitution at the chiral binaphthyl backbone and maintaining PySH-**1** constant (Fig. 2b). Less sterically demanding CPA-**1** with 2,6-diisopropyl phenyl substituents gave a slightly improved e.r. (87:13) and comparable yield. We also observed that 4-*t*Bu-phenyl-substituted CPA-**2** impeded deracemization, whereas CPA-**3** with *para*-biphenyl groups gave **1** with modest optical enrichment (55.5:44:5 e.r.). An improved 89:11 e.r. was obtained by merging the substitution pattern of CPA-**1** and CPA-**3** in CPA-**4**. In parallel, we evaluated a series of commercial PySHs with (*S*)-TRIP (Fig. 2c). While electron-deficient PySH-**2** and PySH-**3** gave moderate enantioenrichment, Me-substituted PySHs restored reactivity and selectivity, with PySH-**6** (89:11 e.r.) outperforming other PySHs. The e.r. was further improved to 94:6 by combining the best-performing components, CPA-**4** and PySH-**6** (Fig. 2d). Fine-tuned CPA-**5** gave a further increase in enantioenrichment (96.5:3.5 e.r.), and with overall lowering of catalyst loadings ([**Ir**] 3 mol%, CPA-**5** (10 mol%), PySH-**6** (10 mol%)), **1** was recovered in nearly quantitative yield, with an excellent e.r. of 97:3. Furthermore, deracemization proceeded with comparable efficiency on a 1-mmol scale (96% yield, 96:4 e.r.), and under basic conditions, the urea-protecting group could be readily removed, giving 2-phenylpyrrolidine (**1a**) with 92% yield and without erosion of the e.r. (Fig. 2e).

## Substrate scope

**Fig. 3:**
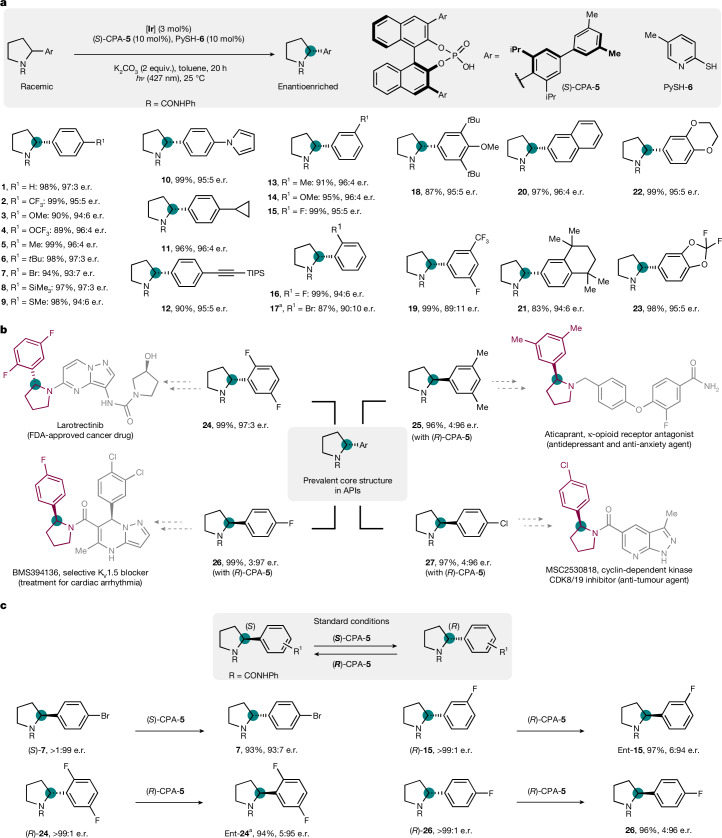
Reaction scope. **a**,**b**, Photochemical deracemization of 2-aryl pyrrolidines (**a**), including those with pharmaceutical relevance (**b**). **c**, Stereochemical interconversions of enantiopure commercially available starting materials. Reactions were run on a 0.1-mmol scale. Yields and e.r. values were measured from isolated material after purification. ^a^96 h reaction time. APIs, active pharmaceutical ingredients; Ent, enantiomer; FDA, US Food and Drug Administration; TIPS, triisopropylsilyl.

Photochemical deracemization across a range of 2-aryl pyrrolidines was achieved under optimized reaction conditions (Fig. 3a). High e.r. values and yields were obtained regardless of the electronic (**2**–**4**) or the steric (**5** and **6**) properties of the *para*-substituted aromatic ring. Different functional groups at the *para* position, including a bromide, a SiMe_3_, a thioether, a pyrrole, a cyclopropyl and an alkyne (**7**–**12**), were well tolerated without compromising reaction efficiency or selectivity. Similar results were obtained with either electron-donating or electron-deficient *meta*-substituted 2-aryl pyrrolidines (**13**–**15**). Furthermore, a fluorine located at the *ortho* position of the aryl ring (**16**) did not affect yield and selectivity, and a more congested 2-bromo phenyl substituent in **17** led to only slightly diminished selectivity. Higher-substituted 2-aryl pyrrolidines also engaged in this photochemical deracemization. For example, high reaction efficiency was conserved with pyrrolidine **18**, featuring bulky 3,5-di-*tert*-butyl-4-methoxyphenyl, whereas **19**, bearing 3-fluoro-5-(trifluoromethyl)phenyl, was obtained with promising selectivity. Substrates with an extended aromatic system (**20**) or with a fused carbocycle (**21**) or heterocycle (**22** and **23**) at the aryl substituent also underwent photochemical deracemization with excellent yield and enantioselectivity.

We demonstrated the synthetic value of our photochemical deracemization protocol by subjecting selected 2-aryl pyrrolidines with pharmaceutical relevance to our reaction conditions (Fig. 3b). For example, 2-(2,5-difluorophenyl)pyrrolidine (**24**, 99%, 97:3 e.r.) is a subunit of larotrectinib, a cancer drug approved by the US Food and Drug Administration^41^. Similarly, aticaprant^42^ has applications as an antidepressant and antianxiety agent and contains the 2-(3,5-dimethylphenyl) pyrrolidine motif (**25**), which underwent deracemization with 90% yield and 96:4 e.r. Furthermore, 2-(4-fluorophenyl)- and 2-(4-chlorophenyl)-substituted pyrrolidines **26** and **27** are present in BMS394136 (ref. ^43^), used in treatment of cardiac arrhythmia, and MSC2530818 (ref. ^44^) (an anti-tumour agent), respectively, and participated efficiently in photochemical optical enrichment. Finally, direct stereochemical interconversion of enantiopure commercial material was achieved under catalyst control (Fig. 3c).

## Mechanistic studies

**Fig. 4:**
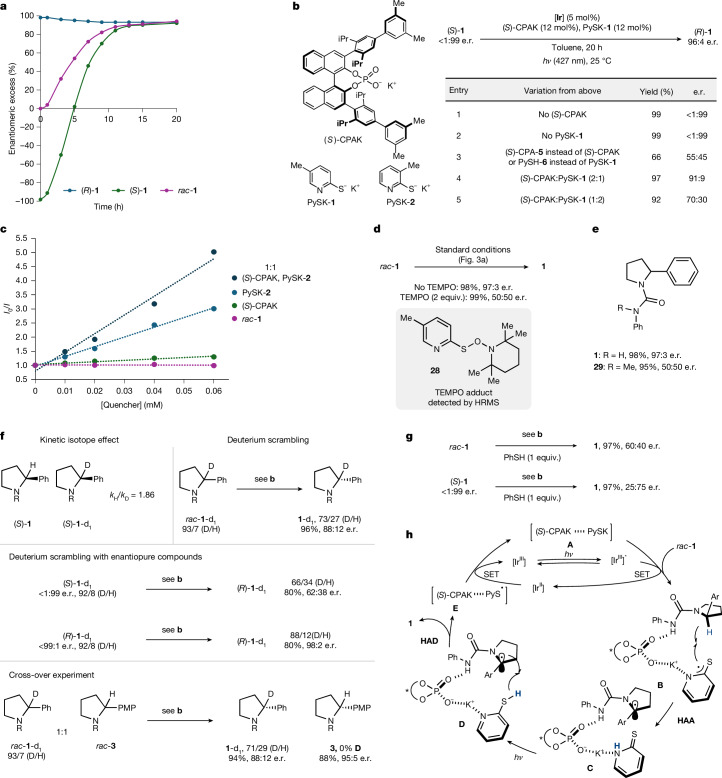
Mechanistic studies. **a**, Time-course experiments converged to steady-state e.r. under standard conditions (Fig. 3a). **b**, Control experiments with (*S*)-CPAK and PySK-**1** indicated catalyst assembly through ion-pair interactions of charged species. **c**, Stern–Volmer luminescence study. **d**, TEMPO experiments: evidence for thiyl radicals and reaction inhibition. **e**, Probing substrate–catalyst interactions. Experiments were performed under standard conditions (Fig. 3a). **f**, Deuterium labelling experiments: kinetic isotope effect (initial rates of racemization were measured under the same reaction conditions as those in **b**), deuterium scrambling with racemic and enantiopure deuterated starting materials and cross-over experiments. **g**, Control experiments with excess thiophenol. **h**, Mechanistic proposal. HRMS, high-resolution mass spectrometry; PMP, *para*-methoxyphenyl; SET, single-electron transfer.

We performed preliminary mechanistic investigations to elucidate the working mode of the chiral self-assembling HAT catalysts in this photochemical deracemization (Fig. 4). Time-course studies with *rac*-**1**, (*S*)-**1** and (*R*)-**1** under standard conditions converged to (*R*)-**1** and established a steady-state e.r. of 97:3 (Fig. 4a). Control experiments with matched enantiomer (*S*)-**1** showed that potassium salts (*S*)-CPAK and PySK-**1** rather than (*S*)-CPA-**5** and PySH-**6** were present in solution, as the stereoinversion to (*R*)-**1** occurred with essentially the same yield and e.r. in the absence of potassium carbonate (Fig. 4b). By contrast, the optical purity of (*S*)-**1** remained unaltered when we performed the reaction in the absence of either (*S*)-CPAK or PySK-**1** (Fig. 4b, entries 1 and 2). Lower selectivities were obtained when we replaced one salt with its conjugated acid or conducted the reaction with an excess of either (*S*)-CPAK or PySK-**1** (Fig. 4b, entries 3–5). These observations suggest the formation of a distinct catalytically active chiral species that assembles in solution from equimolar amounts of both salts, (*S*)-CPAK and PySK-**1**, presumably forming a tight ion pair that is required for reactivity and enantioselectivity of the optical enrichment. Ultraviolet–visible light spectroscopy analysis of the association between (*S*)-CPAK and PySK-**2** by the continuous variation method confirmed formation of a 1:1 assembly (see the Supplementary Information for details). This was further supported by a Stern–Volmer study in which an equimolar solution of (*S*)-CPAK and PySK-**2** quenched the excited state of the iridium photocatalyst more rapidly than (*S*)-CPAK or PySK-**2** individually (Fig. 4c) (see the Supplementary Information for details). Notably, no quenching activity by *rac*-**1** was observed; this precludes a working mechanism proceeding through a sequential single-electron oxidation/deprotonation of *rac*-**1** followed by stereoselective HAD^14^. Furthermore, in the presence of 2,2,6,6-tetramethyl-1-piperidinyloxy (TEMPO), deracemization was inhibited, and TEMPO adduct **28** was detected by high-resolution mass spectrometry, supporting the formation of thiyl radicals that can promote HAT^16,17^ (Fig. 4d). Finally, deracemization did not occur when we subjected *N*-methyl urea **29** to standard reaction conditions, indicating the importance of the N–H bond of *rac*-**1** presumably undergoing H-bonding with the catalyst to achieve high enantioenrichment (Fig. 4e).

We performed deuterium labelling experiments to gain insight into the origins of enantioselectivity (Fig. 4f). A primary kinetic isotope effect (*k*_H_/*k*_D_) of 1.86 was found by measuring the initial rates of racemization of (*S*)-**1** and (*S*)-**1**-d_1_ in parallel experiments (see the Supplementary Information for details). Approximately 20% deuterium scrambling occurred when *rac*-**1**-d_1_ was subjected to photochemical deracemization, and **1**-d_1_ was recovered with a good e.r., indicating that reversible deuterium atom transfer is operative upon deuterium incorporation in PySK-**1** (ref. ^45^). Mono- and di-incorporation of deuterium was confirmed by high-resolution mass spectrometry analysis, indicating that H/D exchange takes place within PySK-**1** (see the Supplementary Information for details). When we performed the reaction with enantiopure deuterated substrates, a higher degree of deuterium scrambling (approximately 25%) was observed with (*S*)-**1**-d_1_. However, only around 5% D/H exchange occurred with (*R*)-**1**-d_1_. This differential reactivity was consistent with an in-cage HAA in which the in situ-generated chiral HAT catalyst differentiates between the two enantiomeric substrates. Moreover, we performed cross-over experiments with *rac*-**1**-d_1_ and *rac*-**3**. Both substrates underwent deracemization with good to excellent e.r. values, but no deuterium incorporation in **3** was detected. This suggests that back-HAT to the postulated radical intermediate occurs intramolecularly before catalyst–substrate dissociation inside the confined chiral cavity provided by the catalyst, probably inducing enantioselectivity. This is in contrast to recent work by Bach^46^, in which photochemical deracemization through reversible HAT was achieved by initial selective HAA at one substrate enantiomer, followed by unselective intermolecular back-HAT after substrate dissociation from the catalyst. Stereoselective HAD was further supported by control experiments in the presence of excess thiophenol, which acts exclusively as an intermolecular hydrogen atom donor, undergoing unselective C(*sp*^3^)–H bond formation (Fig. 4g). When *rac*-**1** was subjected to the deracemization protocol with addition of 1 equiv. of thiophenol, little enantioenrichment was observed. Under the same reaction conditions, (*S*)-**1** reached approximately 50% racemization after 20 h, owing to the statistical unselective formation of both enantiomeric products. In the racemization of (*S*)-**1** under standard deracemization conditions (without excess thiophenol; Fig. 4a) the same 25:75 e.r. had already been achieved after 3 h of reaction time, suggesting that matched enantiomer (*S*)-**1** is removed from the reaction mixture during the deracemization process by direct interconversion to (*R*)-**1** through enantioselective HAD to the carbon-centred radical.

Our experimental evidence suggests that a single HAT assembly formed in situ from CPAs and PySHs achieves photochemical deracemization of 2-aryl pyrrolidines, inducing enantioselectivity in both HAA and the microscopic reverse HAD. This scenario, however, would result in a racemic mixture^35^, unless the two elemental steps occur through distinct diastereomeric transition states. A plausible mechanism is depicted in Fig. 4h. Under light irradiation, the excited state of the iridium photocatalyst engages in electron transfer with in situ-generated chiral HAT assembly **A** and generates delocalized chiral radical in **B**, which undergoes HAA at one enantiomer of the racemic mixture to give a carbon-centred radical. Notably, HAA can occur at two sites of the delocalized chiral radical **B** (*N* and *S*)^47^. This selectivity is dictated by the formation of the more stable tautomer after the abstraction, this being the thione in non-polar solvents^48^. Under photoirradiation, thione–phosphate complex **C** converts into thiol tautomer **D** by means of rapid excited state proton transfer^49^. The deuterium incorporation in PySK-**1** (ref. ^45^) previously observed (Fig. 4f and Supplementary Information) indicates that such phototautomerization takes place under standard reaction conditions. Thus, thiol tautomer **D** promotes stereoselective HAD to the carbon-centred radical, affording enantioenriched product and thiyl radical **E**. A subsequent electron transfer event with the reduced photocatalyst regenerates chiral assembly **A**. The two-component assembly essentially acts as a single chiral HAT catalyst, inducing enantioselectivity in both independent steps (HAA and HAD), through two distinct reactive sites of PySH (*N* and *S*). The thione–thiol phototautomerization thus enables the two elemental steps to proceed through two distinct diastereomeric transition states.

## Conclusion

The spontaneous molecular assembly of chiral HAT catalysts from CPAs and commercial PySHs provides ready access to numerous catalyst permutations and thus surmounts traditional multistep syntheses. This catalytic platform enables photochemical deracemization of general, pharmaceutically relevant *N*-heterocycles such as 2-aryl pyrrolidines. The method is operationally simple, using two readily available reagents that generate the active chiral catalyst in situ. Our findings support a mechanism in which the achiral PySHs that promote HAT are rendered chiral through non-covalent ion-pairing interactions with chiral phosphate ions. With respect to previous work, the chiral HAT assembly achieves optical enrichment through hydrogen atom relay, in which enantioselectivity is controlled in both elemental steps (HAA and HAD), taking advantage of the ability of PySH to undergo thione–thiol phototautomerization. We anticipate that this conceptual in situ assembly of chiral HAT catalysts and the underlying mechanistic blueprint by which they operate will streamline rapid reaction discoveries and advance the field of asymmetric radical chemistry.

## Methods

### General procedure 1 for deracemization of 2-aryl pyrrolidines

In a nitrogen-filled glovebox, an oven-dried 4-ml vial was charged with 5-methylpyridine-2-thiol (1.20 mg, 10.0 µmol, 10 mol%), (*S*)- or (*R*)-CPA-**5** (8.7 mg, 10.0 µmol, 10 mol%), (Ir[dF(CF_3_)ppy]_2_(dtbpy))PF_6_ (3.4 mg, 3.0 µmol, 3 mol%), K_2_CO_3_ (27.6 mg, 0.2 mmol, 2.0 equiv.), substrate (0.1 mmol, 1.0 equiv.) and a magnetic stir bar. Then, anhydrous toluene (2 ml) was added, and the vial was sealed with a PTFE-lined screw cap. The reaction was taken out of the glovebox and irradiated with Penn M2 photoreactor (420 nm) with the following settings: 100% light intensity, 6,800 rpm fan cooling, 509 rpm stirring. After 20 h, the solution was concentrated and purified by flash column chromatography with 4:1:0.5 hexane/Et_2_O/AcOH and then 1:1 hexane/Et_2_O. e.r. values were then determined by high-performance liquid chromatography analysis on a chiral stationary phase.

## Online content

Any methods, additional references, Nature Portfolio reporting summaries, source data, extended data, supplementary information, acknowledgements, peer review information; details of author contributions and competing interests; and statements of data and code availability are available at 10.1038/s41586-026-10692-4.

## Supplementary information

Supplementary Information Materials and methods; preparation of chiral phosphoric acid (*S*)-CPA-**5**; preparation of racemic 2-aryl pyrrolidines; effect of reaction parameters; photochemical deracemization of 2-aryl pyrrolidines; removal of the urea protecting group; assignments of absolute configuration; mechanistic studies; references; and nuclear magnetic resonance spectra.

Peer Review File

## Data Availability

All data are available in the main text or the Supplementary Information.
