# Supplementary material for: Enantioselective hydrogen atom relay via non-covalent catalyst assembly
Source: Nature. 2026 Jun 1;655(8122):381–8. doi: 10.1038/s41586-026-10692-4 (PMC13345913; doi:10.1038/s41586-026-10692-4)
Supplement: Supplementary file 1 — Materials and methods; preparation of chiral phosphoric acid (S)-CPA-5; preparation of racemic 2-aryl pyrrolidines; effect of reaction parameters; photochemical deracemization of 2-aryl pyrrolidines; removal of the urea protecting group; assignments of absolute configuration; mechanistic studies; references; and nuclear magnetic resonance spectra. [file 41586_2026_10692_MOESM1_ESM.pdf]

nature portfolio

https://doi.org/10.1038/s41586-026-10692-4

Supplementary information

Enantioselective hydrogen atom relay via non-covalent catalyst assembly

In the format provided by the authors and unedited

Supplementary Information

Enantioselective hydrogen atom relay via non-covalent catalyst assembly

Navadheer Yalamanchili[1], Jules Hugo Alexandre[1], Robert L. Anderson[1] and Giuseppe Zuccarello[1]*

[1]Laboratory of Asymmetric Catalysis and Synthesis, Institute of Chemical Sciences and Engineering, École Polytechnique Fédérale de Lausanne, 1015 Lausanne, Switzerland.

*email: giuseppe.zuccarello@epfl.ch

Table of Contents

Materials and Methods 3
Preparation of Chiral Phosphoric Acid (*S*)-CPA-5 4
Preparation of Racemic 2-Aryl pyrrolidines 7
Effect of Reaction Parameters 20
Photochemical Deracemization of 2-Aryl pyrrolidines 22
  General Procedures for Deracemization 22
  Characterization of Products 23
  Reactions with Enantiopure Substrates: Interconversions 51
Removal of the Urea Protecting Group 55
Assignments of Absolute Configuration 56
Mechanistic Studies 57
  Synthesis of Potassium Salts of (*S*)-CPAK, PySK-1 and PySK-2 57
  Synthesis of Deuterated Substrate *rac*-1-d_1 58
  Time-Course Profile of Deracemization 59
  Stern-Volmer Luminescence Quenching and UV-Vis Experiments 60
  Job Plot Analysis of Catalyst Components 63
  Impact of TEMPO on the Deracemization Reaction 67
  Deuterium Labeling Experiment 68
  Experiments with Excess PySH-6 73
  Experiments with Excess Thiophenol 73
  Experiments with Thiophenol instead of PySH-6 73
References 74
NMR Spectra 75

Materials and Methods

Unless otherwise indicated, all reactions were carried out under nitrogen atmosphere by using standard Schlenk or glovebox techniques in oven-dried glassware with magnetic stirring. Reagents were purchased and used as obtained from the suppliers. Chiral phosphoric acids and 2-mercaptopyridines were purchased from combi-blocks ((*S*)-TRIP) and BLD Pharm (CPA-**2** and CPA-**3**, PySH-**1** to PySH-**6**) or synthesized (CPA-**1** and CPA-**4**) according to reported procedures (*59*). Anhydrous toluene was purchased from Thermoscientific Acros (99.85%, Extra Dry over Molecular Sieve, AcroSeal) and stored in a glovebox; other solvents were obtained using a solvent purification system with an aluminum oxide column (Innovative Technologies). $[Ir[dF(CF_3)ppy]_2(dtbbpy)PF_6$ (BLD Pharm, 98%), 5-Methylpyridine-2(1H)-thione (BLD Pharm, 97%), and potassium carbonate (Sigma-Aldrich, 99%), were used as received. Flash chromatography was performed with Silicycle silica gel 60 (0.040-0.063 μm grade). Analytical thin layer chromatography was performed with commercial glass plates coated with 0.25 mm silica gel (E. Merck, Kieselgel 60 F254). Compounds were either visualized under UV-light at 254 nm or by dipping the plates in an aqueous potassium permanganate solution followed by heating. NMR spectra were recorded on a Bruker Avance 400 MHz or 600 MHz spectrometer with a BBFOz ATMA probe. The peaks were internally referenced to residual non-deuterated chloroform in $CDCl_3$ (7.26 ppm for ^1H NMR, 77.16 ppm for ^{13}C NMR), benzene in C_6D_6 (7.16 ppm for ^1H NMR, 128.06 ppm for ^{13}C NMR) or acetonitrile in CD_3CN (1.94 ppm for ^1H NMR, 1.32 ppm for ^{13}C NMR). Splitting patterns are designated as s, singlet; d, doublet; t, triplet; q, quartet; sept, septet; m, multiplet; brs, broad singlet. IR spectra were recorded on a Perkin-Elmer FT-IR spectrometer. Absorbance frequencies are reported in reciprocal centimeters (cm^{-1}). High-resolution mass spectrometry (HRMS) data were acquired on an Agilent LC-MS TOF (Multimode: ESI + APCI) or an LTQ Orbitrap FTMS instrument (LTQ Orbitrap Elite FTMS, Thermo Scientific, Bremen, Germany) equipped with an Ion Max APPI ionization source with a VUV Kr lamp (Syagen, CA, USA). The enantiomeric ratio value was determined on an Agilent HPLC using CHIRALPAK column with hexane and 2-propanol as eluent. Optical rotations were measured on a Polartronic M polarimeter using a 0.5 cm cell with a Na 589 nm filter. Stern–Volmer experiments were conducted on an Agilent Cary Eclipse Fluorescence Spectrophotometer. UV-Vis spectra were acquired on an Agilent Cary 60 UV-Visible spectrophotometer.

Preparation of Chiral Phosphoric Acid (*S*)-CPA-5

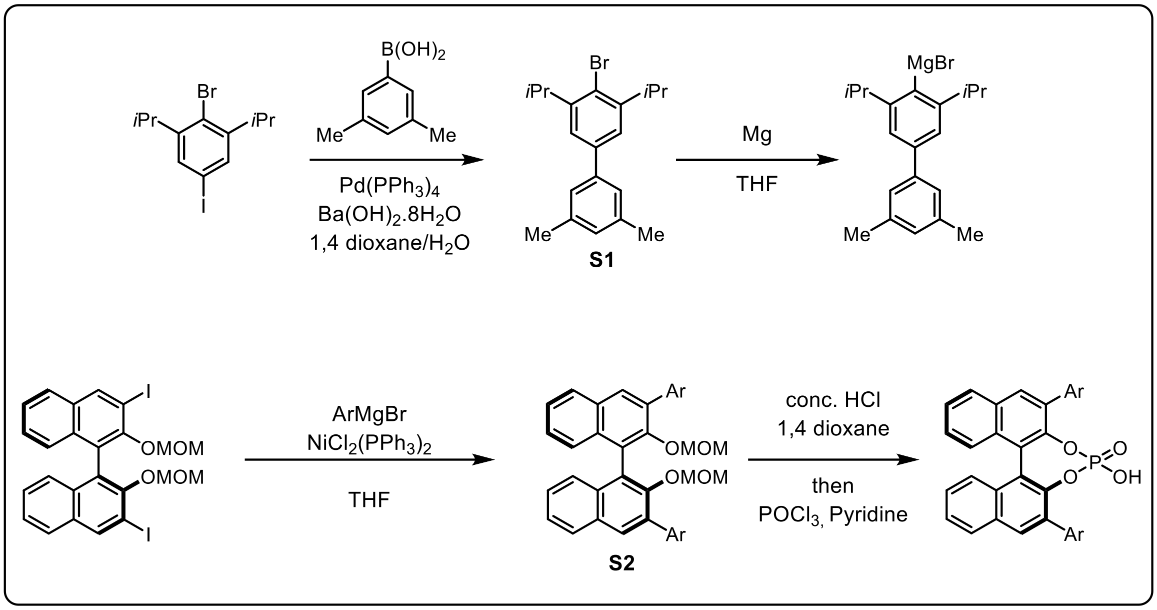

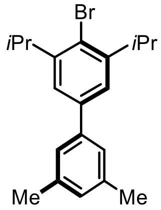

4-Bromo-3,5-diisopropyl-3',5'-dimethyl-1,1'-biphenyl (S1): A 250 mL Schlenk flask was flame-dried and charged with 2-bromo-5-iodo-1,3-diisopropylbenzene[1] (10.9 g, 29.7 mmol, 1.0 equiv), (3,5-dimethylphenyl)boronic acid (4.90 g, 32.7 mmol, 1.1 equiv), $Ba(OH)_2{\cdot}8H_2O$ (18.7 g, 59.4 mmol, 2.0 equiv), and $Pd(PPh_3)_4$ (1.71 g, 1.48 mmol, 5 mol%) in 1,4-dioxane/H_2O (90 mL/30 mL). The reaction was stirred at 110 °C for 15 h under nitrogen and then quenched by adding HCl (1M). The mixture was filtered through Celite®, and the crude mixture was extracted with EtOAc (3x) and the combined organic extracts were washed with brine, dried with Na_2SO_4, and concentrated under reduced pressure. The product was purified by flash column chromatography on silica gel (100% hexane) to obtain the title compound (6.81 g, 19.7 mmol, 66% yield) as a white solid.
M.p. = 94 – 96 °C (hexane). **^{1}H NMR** (400 MHz, $CDCl_3$) δ 7.30 (s, 2H), 7.17 (s, 2H), 7.02 (s, 1H), 3.56 (hept, J = 6.9 Hz, 2H), 2.41 (s, 6H), 1.31 (d, J = 6.8 Hz, 12H). **^{13}C NMR** (101 MHz, $CDCl_3$) δ 148.1, 141.3, 140.8, 138.5, 129.2, 125.7, 125.2, 123.3, 33.8, 23.3, 21.6. **HRMS** (ESI/QTOF) *m/z*: $[M + H]^+$ calcd for $C_{20}H_{26}Br^+$ 345.1212; Found 345.1209. **IR** (ATR): 2962, 2925, 2868, 1604, 1567, 1460, 1428, 1014, 877, 846, 703 cm^{-1}.

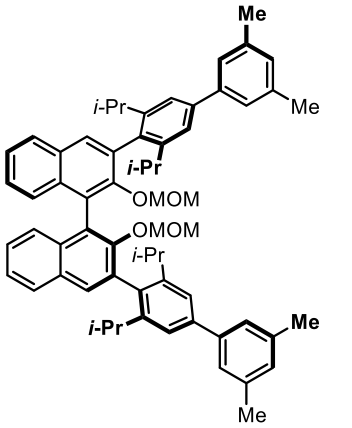

(*S*)-3,3'-Bis(3,5-diisopropyl-3',5'-dimethyl-[1,1'-biphenyl]-4-yl)-2,2'-bis(methoxymethoxy)-1,1'-binaphthalene (S2): A flame-dried 100 mL two-neck flask, equipped with a stir bar and reflux condenser was charged with magnesium turnings (133 mg, 5.49 mmol, 9.0 equiv). The flask was heated under vacuum with a heat gun and cooled to room temperature under an atmosphere of nitrogen and then THF (2.5 mL) was added. A solution of 4-bromo-3,5-diisopropyl-3',5'-dimethyl-1,1'-biphenyl (1.26 g, 3.66 mmol, 6.0 equiv) in THF (9.3 mL) was added followed by 1,2-dibromoethane (0.05 mL). The reaction mixture was heated to reflux for 3 h, upon which, a light grey solution formed.

Separately, a flame-dried 100 mL Schlenk flask, was equipped with a stir bar and charged with (*S*)-3,3'-diiodo-2,2'-bis(methoxymethoxy)-1,1'-binaphthalene (382 mg, 0.61 mmol, 1.0 equiv) and $NiCl_2(PPh_3)_2$ (39.9 mg, 0.061mmol, 0.1 equiv) and THF (1.9 mL). The previously prepared Grignard solution was added to the reaction mixture over 10 minutes and stirred at 38 ºC for 18 h. The reaction was quenched by adding sat. NH_4Cl and extracted with CH_2Cl_2 (3x). The combined organic layers were dried over Na_2SO_4, filtered and concentrated. The product was purified by flash column chromatography on silica gel (80:1→20:1, Hexane/Et_2O) to obtain the title compound (314 mg, 0.348 mmol, 57% yield) as a white solid.

M.p. = 168 – 172 °C (Et_2O). **^{1}H NMR** (400 MHz, $CDCl_3$) δ 7.91 – 7.86 (m, 2H), 7.82 (s, 2H), 7.50 – 7.41 (m, 8H), 7.40 – 7.32 (m, 2H), 7.27 (s, 4H), 7.03 (s, 2H), 4.30 (s, 4H), 3.02 – 2.85 (m, 4H), 2.43 (s, 12H), 2.31 (s, 6H), 1.32 (d, *J* = 6.9 Hz, 6H), 1.28 (d, 6.8 Hz, 6H), 1.27 (d, 6.8 Hz, 6H), 1.06 (d, *J* = 6.9 Hz, 6H). **^{13}C NMR** (101 MHz, $CDCl_3$) δ 152.5, 148.2, 147.8, 142.2, 141.2, 138.4, 135.0, 134.0, 133.8, 131.1, 130.6, 128.9, 128.0, 126.42, 126.36, 126.2, 125.2, 125.1, 122.0, 121.7, 98.0, 55.5, 31.3, 31.1, 26.1, 25.5, 23.4, 23.3, 21.6. **HRMS** (ESI/QTOF) *m/z*: $[M + Na]^+$ calcd for $C_{64}H_{70}NaO_4^+$ 925.5166; Found 925.5168. **IR** (ATR): 2959, 2923, 2866, 1598, 1461, 1388, 1156. 1078, 998, 970, 880, 750 cm^{-1}. **$[\alpha]_D^{20}$** = +90.0 ($CHCl_3$, 1.0).

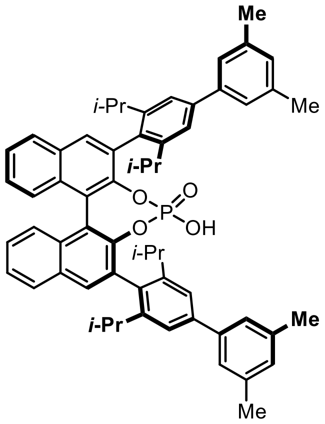

(*S*)-2,6-Bis(3,5-diisopropyl-3',5'-dimethyl-[1,1'-biphenyl]-4-yl)-4-hydroxydinaphtho[2,1-*d*:1',2'-*f*][1,3,2]dioxaphosphepine 4-oxide ((*S*)-CPA-**5**):[2] (*S*)-3,3'-bis(3,5-diisopropyl-3',5'-dimethyl-[1,1'-biphenyl]-4-yl)-2,2'-bis(methoxymethoxy)-1,1'-binaphthalene (1.46 g, 1.61 mmol, 1.0 equiv) was dissolved in 1.4-dioxane (16.8 mL) and concentrated aqueous HCl (6.30 mL, 75.6 mmol, 47.0 equiv. 37%) was added. The reaction mixture was stirred at 70 °C for 14 h and then quenched at room temperature by adding sat. $NaHCO_3$. The aqueous phase was extracted with EtOAc (3x) and the combined organic layers were washed with brine, dried over Na_2SO_4, filtered and concentrated. The crude material was used in the next step without further purification.

A flame-dried 25 mL Schlenk flask was charged with the crude deprotected binaphthol derivative and dissolved in dry pyridine (3.3 mL). $POCl_3$ (451 µL, 4.82 mmol, 3.0 equiv.) was added to the solution and the reaction mixture was stirred for 14 h at 105 °C. The reaction mixture was cooled to 0 °C and water (3.3 mL) was slowly added and the reaction was stirred for additional 3 h at 100 °C. The reaction was diluted with CH_2Cl_2 and quenched by the addition of HCl (1 M). The organic phase is thoroughly washed with HCl (3x, 1 M), dried over Na_2SO_4, filtered and concentrated. The product was purified by flash column chromatography on silica gel (4:1→2:1, Hexane/EtOAc). The obtained residue was then dissolved in CH_2Cl_2 and washed with HCl (2x, 6 M). The organic phase was dried over Na_2SO_4, filtered and concentrated to give the title compound (1.13 g, 1.28 mmol, 80% yield) as an off-white solid.

M.p. = 242 – 245 °C (Hexane/EtOAc/CH_2Cl_2). **^{1}H NMR** (400 MHz, CD_2Cl_2) δ 7.94 (d, *J* = 8.2 Hz, 2H), 7.84 (s, 2H), 7.57 – 7.46 (m, 2H), 7.33 – 7.28 (m, *J* = 4.9 Hz, 4H), 7.25 (d, *J* = 3.6 Hz, 4H), 7.17 (s, 4H), 6.94 (s, 2H), 2.67 – 2.59 (m, 2H), 2.59 – 2.48 (m, 2H), 2.28 (s, 12H), 1.13 (d, *J* = 6.8 Hz, 6H), 0.93 – 0.85 (m, 12H), 0.81 (d, *J* = 6.7 Hz, 6H). **^{13}C NMR** (101 MHz, CD_2Cl_2) δ 148.6 (d, *J* = 36.3 Hz), 146.0 (d, *J* = 9.2 Hz), 142.0 (d, *J* = 29.8 Hz), 138.3, 133.2, 133.0, 132.7, 132.0 (d, *J* = 3.4 Hz), 131.5, 129.0, 128.6, 127.6, 126.7, 126.3, 125.5, 122.8, 122.3 (d, *J* = 2.2 Hz), 121.6, 31.6, 31.2, 26.4, 25.0, 23.4, 23.2, 21.4. **^{31}P NMR** (162 MHz, CD_2Cl_2) δ 3.4. **HRMS** (ESI/QTOF) *m/z*: $[M]^-$ calcd for $C_{60}H_{60}O_4P^-$ 875.4235; Found 875.4260. **IR** (ATR): 2960, 2925, 2867, 1599, 1447, 1279, 1248, 1206, 1021, 900, 750 cm^{-1}. **$[\alpha]_D^{20}$** = +73.2 ($CHCl_3$, 1.0).

Preparation of Racemic 2-Aryl pyrrolidines

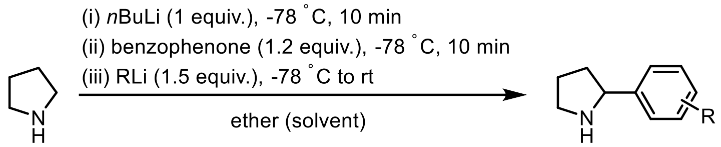

General Procedure 1 (GP-1): direct C(sp^3)–H arylation of pyrrolidine

This procedure was adapted from literature.[3] A flame-dried 50 mL two-necked flask was charged with the arylbromide (1.5 equiv, with respect to pyrrolidine) and dissolved in dry Et_2O (10 mL). The solution was cooled to –78 °C and *n*BuLi (1.5 equiv, 2.5 M in hexane) was added dropwise. The reaction mixture was allowed to stir at the same temperature for 30 min, then warmed up to room temperature over 30 min to give the corresponding aryllithium.

A flame-dried 100 mL Schlenk flask was charged with a solution of pyrrolidine (5.0 mmol, 1.0 equiv) in dry Et_2O (10 mL). The solution was cooled to –78 °C and *n*BuLi (1.0 equiv, 2.5 M in hexane) was added dropwise. The reaction mixture was stirred at the same temperature for 10 min and then a solution of benzophenone (1.2 equiv) in anhydrous ether (5 mL) was added. The resulting mixture was stirred at –78 °C for an additional 10 min followed by the dropwise addition of the freshly prepared aryllithium reagent (1.5 equiv). The reaction was then allowed to warm up to room temperature and stirred for an additional two hours before quenching by the addition of MeOH (5 mL) at 0 °C. The reaction mixture was diluted with Et_2O and washed with water. The aqueous layer was extracted with Et_2O (3x) and the combined organic layers were washed with brine, dried over Na_2SO_4, filtered and concentrated. The obtained crude was purified by flash column chromatography on silica gel.

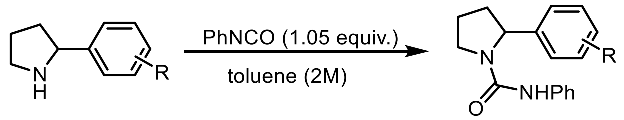

General Procedure 2 (GP-2): protection of racemic 2-aryl pyrrolidines

The 2-arylpyrrolidine was dissolved in toluene (2 M) and phenyl isocyanate (1.05 equiv) was added dropwise. The reaction was stirred at room temperature and monitored by TLC until consumption of the starting material. The crude was purified by flash column chromatography or by precipitation from a hexane/Et_2O (5:1) solution.

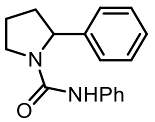

***N*,2-Diphenylpyrrolidine-1-carboxamide (*rac*-1)**: The title compound was synthesized according to **GP-2** from 2-phenylpyrrolidine (736 mg, 5.00 mmol). The product was purified by precipitation from hexane/Et_2O (1.28 g, 4.82 mmol, 96% yield). White solid.

M.p. = 142 – 144 °C ($CDCl_3$). **^{1}H NMR** (400 MHz, $CDCl_3$) δ 7.44 – 7.36 (m, 2H), 7.36 – 7.28 (m, 3H), 7.22 – 7.15 (m, 2H), 7.14 – 7.09 (m, 2H), 6.98 – 6.89 (m, 1H), 6.03 (s, 1H), 4.87 (dd, *J* = 7.9, 3.9 Hz, 1H), 3.89 – 3.79 (m, 1H), 3.79 – 3.70 (m, 1H), 2.53 – 2.36 (m, 1H), 2.08 – 1.85 (m, 3H). **^{13}C NMR** (101 MHz, $CDCl_3$) δ 154.4, 142.8, 139.2, 129.3, 128.9, 128.1, 126.0, 122.8, 119.4, 61.5, 47.7, 37.1, 23.3. **HRMS** (ESI/QTOF) *m/z*: $[M + Na]^+$ Calcd for $C_{17}H_{18}N_2NaO^+$ 289.1311;

Found 289.1313. **IR** (ATR): 3316, 3058, 3027, 2872, 1647, 1594, 1529, 1440, 1370, 1301, 1243, 1214, 904, 750, 694 cm^{-1}.

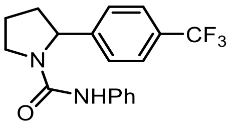

***N*-Phenyl-2-(4-(trifluoromethyl)phenyl)pyrrolidine-1-carboxamide** (*rac*-**2**): The title compound was synthesized according to **GP-2** from 2-(4-(trifluoromethyl)phenyl)pyrrolidine (528 mg, 2.45 mmol). The product was purified by flash column chromatography on silica gel (9:1→1:1, pentane/EtOAc). (777 mg, 2.32 mmol, 95% yield). Pale-yellow solid.
M.p. = 150 – 152 °C (hexane/Et_2O). **^{1}H NMR** (400 MHz, $CDCl_3$) δ 7.62 (d, *J* = 8.4 Hz, 2H), 7.40 (d, *J* = 8.4 Hz, 2H), 7.29 – 7.17 (m, 4H), 6.99 (tt, *J* = 7.0, 1.6 Hz, 1H), 6.11 (s, 1H), 5.06 (dd, *J* = 8.1, 3.7 Hz, 1H), 3.80 – 3.72 (m, 2H), 2.51 – 2.34 (m, 1H), 2.05 – 1.95 (m, 2H), 1.95 – 1.86 (m, 1H). **^{13}C NMR** (101 MHz, $CDCl_3$) δ 154.1, 147.4, 138.9, 130.5 – 129.3 (m), 129.0, 126.2, 126.0 (q, *J* = 3.8 Hz), 124.2 (q, *J* = 272.4 Hz), 123.2, 119.6, 61.0, 47.5, 36.0, 23.6. **^{19}F NMR** (376 MHz, $CDCl_3$) δ –62.5. **HRMS** (ESI/QTOF) *m/z*: $[M + H]^+$ Calcd for $C_{18}H_{18}F_3N_2O^+$ 335.1366; Found 335.1373. **IR** (ATR): 3316, 2972, 1648, 1595, 1443, 1370, 1325, 1244, 1122, 1067, 835, 754, 693 cm^{-1}

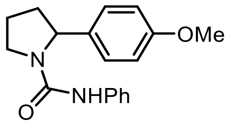

2-(4-Methoxyphenyl)-*N*-phenylpyrrolidine-1-carboxamide (*rac*-**3**): The title compound was synthesized according to **GP-2** from 2-(4-methoxyphenyl)pyrrolidine (328 mg, 1.85 mmol). The product was purified by flash column chromatography on silica gel (9:1→1:1, pentane/EtOAc). (462 mg, 1.56 mmol, 84% yield). Pale-yellow solid.
M.p. = 59 – 61 °C (pentane/EtOAc). **^{1}H NMR** (400 MHz, $CDCl_3$) δ 7.29 – 7.22 (m, 2H), 7.20 – 7.15 (m, 2H), 7.15 – 7.10 (m, 2H), 6.96 – 6.88 (m, 3H), 6.09 (s, 1H), 4.87 – 4.69 (m, 1H), 3.87 – 3.78 (m, 1H), 3.82 (s, 3H), 3.77 – 3.69 (m, 1H), 2.52 – 2.32 (m, 1H), 2.06 – 1.96 (m, 1H), 1.96 – 1.85 (m, 2H). **^{13}C NMR** (101 MHz, $CDCl_3$) δ159.4, 154.4, 139.2, 134.6, 128.8, 127.2, 122.7, 119.3, 114.7, 61.0, 55.5, 47.6, 37.3, 23.3. **HRMS** (ESI/QTOF) *m/z*: $[M + Na]^+$ Calcd for $C_{18}H_{20}N_2NaO_2^+$ 319.1417; Found 319.1417. **IR** (ATR): 3314, 2968, 2873, 1649, 1595, 1510, 1441, 1369, 1243, 1175, 1033, 829, 809, 693 cm^{-1}.

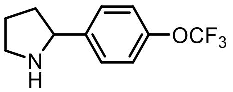

2-(4-(Trifluoromethoxy)phenyl)pyrrolidine (*rac*-**4a**): The title compound was synthesized according to **GP-1** from 1-bromo-4-(trifluoromethoxy)benzene (1.09 g, 7.5 mmol). The product was purified by flash column chromatography on silica gel (9:1 EtOAc/MeOH→ 9:1:0.1, EtOAc/MeOH/*i*$PrNH_2$), (308 mg, 1.33 mmol, 27% yield). Brown oil.
^{1}H NMR (400 MHz, $CDCl_3$) δ 7.42 – 7.35 (m, 2H), 7.18 – 7.10 (m, , 2H), 4.13 (t, *J* = 7.7 Hz, 1H), 3.23 – 3.14 (m, 1H), 3.07 – 2.97 (m, 1H), 2.24 – 2.13 (m, 1H), 2.02 (s, 1H), 1.95 – 1.78 (m, 2H), 1.69 – 1.55 (m, 1H). **^{13}C NMR** (101 MHz, $CDCl_3$) δ 147.9 (q, *J* = 1.9 Hz), 143.9, 127.8, 120.8, 120.5 (q, *J* = 256.8 Hz) 61.8, 47.0, 34.5, 25.5. **^{19}F NMR** (376 MHz, $CDCl_3$) δ –57.9. **HRMS**

(ESI/QTOF) *m/z*: $[M + H]^+$ Calcd for $C_{11}H_{13}F_3NO^+$ 232.0944; Found 232.0944. **IR** (ATR): 2962, 2871, 1507, 1368. 1258, 1221, 1104, 920, 848, 807 cm^{-1}.

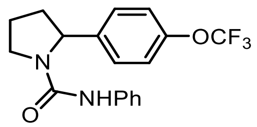

***N*-Phenyl-2-(4-(trifluoromethoxy)phenyl)pyrrolidine-1-carboxamide** (*rac*-**4**): The title compound was synthesized according to **GP-2** from 2-(4-(trifluoromethoxy)phenyl)pyrrolidine (277 mg, 1.20 mmol). The product was purified by flash column chromatography on silica gel (2:1→1:1, hexane/Et_2O, then 1:1, hexane/EtOAc). (378 mg, 1.08 mmol, 90% yield). Pale-yellow gum.

M.p. = 120 – 122 °C (hexane/EtOAc). ^{1}H **NMR** (400 MHz, $CDCl_3$) δ 7.36 – 7.28 (m, 2H), 7.25 – 7.16 (m, 6H), 7.02 – 6.92 (m, 1H), 6.12 (s, 1H), 4.99 (dd, *J* = 8.0, 3.7 Hz, 1H), 3.82 – 3.66 (m, 2H), 2.50 – 2.35 (m, 1H), 2.08 – 1.82 (m, 3H). **^{13}C NMR** (101 MHz, $CDCl_3$) δ 154.2, 148.6, 141.9, 139.0, 128.9, 127.3, 123.1, 121.6, 120.2 (q, *J* = 256.0 Hz) 119.6, 60.7, 47.5, 36.3, 23.4. **^{19}F NMR** (376 MHz, $CDCl_3$) δ –57.9. **HRMS** (ESI/QTOF) *m/z*: $[M + H]^+$ Calcd for $C_{18}H_{18}F_3N_2O_2^+$ 351.1315; Found 351.1319. **IR** (ATR): 3319, 2973, 2874, 1647, 1595, 1531, 1443, 1370, 1256, 1220, 1162, 752, 692 cm^{-1}.

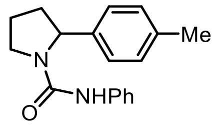

***N*-Phenyl-2-(*p*-tolyl)pyrrolidine-1-carboxamide** (*rac*-**5**): The title compound was synthesized according to **GP-2** from 2-(*p*-tolyl)pyrrolidine (350 mg, 2.71 mmol). The product was purified by precipitation from hexane/Et_2O (376 mg, 1.34 mmol, 62% yield). White solid.

M.p. =120 – 122 °C (hexane/Et_2O). **^{1}H NMR** (400 MHz, $CDCl_3$) δ 7.24 – 7.09 (m, 8H), 6.94 (tt, *J* = 7.4, 1.4 Hz, 1H), 6.05 (s, 1H), 4.81 (dd, *J* = 8.1, 4.2 Hz, 1H), 3.89 – 3.78 (m, 1H), 3.78 – 3.64 (m, 1H), 2.51 – 2.40 (m, 1H), 2.36 (s, 3H), 2.06 – 1.96 (m, 1H), 1.96 – 1.85 (m, 2H). **^{13}C NMR** (101 MHz, $CDCl_3$) δ 154.4, 139.7, 139.3, 137.9, 130.0, 128.8, 126.0, 122.7, 119.3, 61.3, 47.6, 37.3, 23.3, 21.2. **HRMS** (ESI/QTOF) *m/z*: $[M + H]^+$ Calcd for $C_{18}H_{21}N_2O^+$ 281.1648; Found 281.1650. **IR** (ATR): 3317, 3052, 2971, 2871, 1648, 1595, 1530, 1441, 1368, 1243, 751, 652 cm^{-1}.

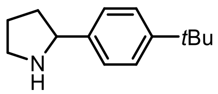

2-(4-(*tert*-Butyl)phenyl)pyrrolidine (*rac*-**6a**): The title compound was synthesized according to **GP-1** from 1-bromo-4-(*tert*-butyl)benzene (1.59 g, 7.5 mmol). The product was purified by flash column chromatography on silica gel (9:1 EtOAc/MeOH→ 9:1:0.1, EtOAc/MeOH/*i*$PrNH_2$), (411 mg, 2.02 mmol, 40% yield). Yellow oil.

^{1}H NMR (400 MHz, $CDCl_3$) δ 7.38 – 7.32 (m, 2H), 7.32 – 7.27 (m, 2H), 4.12 – 4.07 (m, 1H), 3.23 – 3.16 (m, 1H), 3.03 – 2.95 (m, 1H), 2.50 (bs, 1H), 2.24 – 2.11 (m, 1H), 1.99 – 1.89 (m, 1H), 1.89 – 1.79 (m, 1H), 1.77 – 1.63 (m, 1H), 1.32 (s, 9H). **^{13}C NMR** (101 MHz, $CDCl_3$) δ 149.9, 141.4, 126.4, 125.4, 62.5, 47.0, 34.6, 34.2, 31.5, 25.7. **HRMS** (ESI/QTOF) *m/z*: $[M + H]^+$ Calcd for $C_{14}H_{22}N^+$ 204.1747; Found 204.1744. **IR** (ATR): 2960, 2867, 1511, 1460, 1393, 1362, 1268, 1108, 827, 570 cm^{-1}.

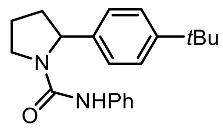

2-(4-(*tert*-Butyl)phenyl)-*N*-phenylpyrrolidine-1-carboxamide (*rac*-6): The title compound was synthesized according to **GP-2** from 2-(4-(*tert*-butyl)phenyl)pyrrolidine (368 mg, 1.81 mmol). The product was purified by precipitation from hexane/Et_2O (5:1) (467 mg, 1.45 mmol, 80% yield). Pink solid.

M.p. = 160 – 162 °C (hexane/Et_2O). **^{1}H NMR** (400 MHz, $CDCl_3$) δ 7.43 – 7.37 (m, 2H), 7.28 – 7.22 (m, 2H), 7.21 – 7.14 (m, 2H), 7.14 – 7.09 (m, 2H), 6.96 – 6.90 (m, 1H), 6.07 (s, 1H), 4.83 (dd, *J* = 8.0, 3.8 Hz, 1H), 3.88 – 3.78 (m, 1H), 3.78 – 3.69 (m, 1H), 2.50 – 2.37 (m, 1H), 2.06 – 1.85 (m, 3H), 1.33 (s, 9H). **^{13}C NMR** (101 MHz, $CDCl_3$) δ 154.4, 151.1, 139.6, 139.3, 128.8, 126.1, 125.8, 122.7, 119.4, 61.2, 47.6, 37.0, 34.7, 31.5, 23.3. **HRMS** (ESI/QTOF) *m/z*: $[M + H]^+$ Calcd for $C_{21}H_{27}N_2O^+$ 323.2118; Found 323.2120. **IR** (ATR): 3326, 2962, 2869, 1649, 1595, 1531, 1441, 1363, 1243, 828, 751, 692 cm^{-1}.

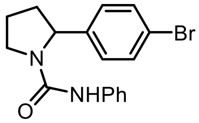

2-(4-Bromophenyl)-*N*-phenylpyrrolidine-1-carboxamide (*rac*-7): The title compound was synthesized according to **GP-1** from 2-(4-bromophenyl)pyrrolidine (300 mg, 1.32 mmol). The product was purified by precipitation from hexane/Et_2O (5:1) (414 mg, 1.20 mmol, 90% yield). White solid.

M.p. = 150 – 152 °C (hexane/Et_2O). **^{1}H NMR** (400 MHz, $CDCl_3$) δ 7.55 – 7.44 (m, 2H), 7.28 – 7.16 (m, 6H), 6.94 – 7.00 (m, 1H), 6.02 (s, 1H), 4.90 (dd, *J* = 7.9, 3.8 Hz, 1H), 3.73 – 3.77 (m, 2H), 2.35 – 2.48 (m, 1H), 2.05 – 1.93 (m, 2H), 1.93 – 184 (m, 1H). **^{13}C NMR** (101 MHz, $CDCl_3$) δ 154.1, 142.2, 139.0, 132.2, 128.9, 127.6, 123.0, 121.5, 119.5, 60.8, 47.5, 36.4, 23.4. **HRMS** (ESI/QTOF) *m/z*: $[M + H]^+$ Calcd for $C_{17}H_{18}BrN_2O^+$ 345.0597; Found 345.0609. **IR** (ATR): 3310, 2972, 2947, 2874, 1646, 1594, 1531, 1441, 1367, 1213, 1009, 820, 751, 692 cm^{-1}.

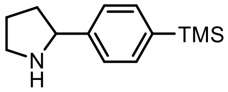

2-(4-(Trimethylsilyl)phenyl)pyrrolidine (*rac*-8a): The title compound was synthesized according to **GP-1** from (4-bromophenyl)trimethylsilane (1.72 g, 7.5 mmol). The product was purified by flash column chromatography on silica gel (9:1 EtOAc/MeOH→ 9:1:0.1, EtOAc/MeOH/*i*$PrNH_2$), (480 mg, 2.19 mmol, 44% yield). Yellow oil.

^{1}H NMR (400 MHz, $CDCl_3$) δ 7.52 – 7.47 (m, 2H), 7.39 – 7.35 (m, 2H), 4.14 – 4.8 (m, 1H), 3.24 – 3.16 (m, 1H), 3.04 – 2.96 (m, 1H), 2.76 (s, 1H), 2.25 – 2.11 (m, 1H), 1.99 – 1.89 (m, 1H), 1.89 – 1.79 (m, 1H), 0.28 (s, 9H). **^{13}C NMR** (101 MHz, $CDCl_3$) δ 145.2, 138.8, 133.5, 126.1, 62.7, 47.0, 34.3, 25.6, –10.0. **HRMS** (ESI/QTOF) *m/z*: $[M + H]^+$ Calcd for $C_{13}H_{22}NSi^+$ 220.1516; Found 220.1515. **IR** (ATR): 2953, 2870, 1400, 1246, 1104, 835, 754, 690 cm^{-1}.

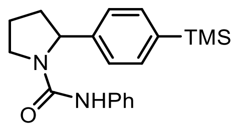

***N*-Phenyl-2-(4-(trimethylsilyl)phenyl)pyrrolidine-1-carboxamide (*rac*-8)**: The title compound was synthesized according to **GP-2** from 2-(4-(trimethylsilyl)phenyl)pyrrolidine (418 mg, 1.91 mmol). The product was purified by precipitation from hexane/Et_2O (5:1) (323 mg, 0.95 mmol, 50% yield). White solid.

M.p. = 144 – 146 °C (hexane/Et_2O). **^{1}H NMR** (400 MHz, $CDCl_3$) δ 7.54 (d, *J* = 7.5 Hz, 2H), 7.30 (d, *J* = 7.5 Hz, 2H), 7.24 – 7.08 (m, 4H), 6.95 (t, *J* = 6.9 Hz, 1H), 6.05 (s, 1H), 4.87 (dd, *J* = 8.2, 3.6 Hz, 1H), 3.88 – 3.69 (m, 2H), 2.54 – 2.35 (m, 1H), 2.09 – 1.83 (m, 3H), 0.28 (s, 9H). **^{13}C NMR** (101 MHz, $CDCl_3$) δ 154.7, 143.6, 140.6, 139.5, 134.6, 129.2, 125.7, 123.1, 119.8, 61.8, 48.0, 37.2, 23.7, –0.7. **HRMS** (ESI/QTOF) *m/z*: $[M + H]^+$ Calcd for $C_{20}H_{27}N_2OSi^+$ 339.1887; Found 339.1886. **IR** (ATR): 3318, 2953, 2873, 1648, 1595, 1531, 1442, 1369, 1246, 838, 723, 692 cm^{-1}.

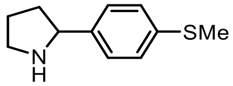

2-(4-(Methylthio)phenyl)pyrrolidine (*rac*-9a): The title compound was synthesized according to **GP-1** from (4-bromophenyl)(methyl)sulfane (1.52 g, 7.5 mmol). The residue was purified by flash column chromatography on silica gel (9:1, EtOAc/MeOH then 9:1:0.1, EtOAc/MeOH/*i*$PrNH_2$). (429 mg, 2.22 mmol, 44% yield). Yellow solid.

M.p. = 37 – 39 °C (hexane/Et_2O/ *i*$PrNH_2$). **^{1}H NMR** (400 MHz, $CDCl_3$) δ 7.28 (d, *J* = 8.1 Hz, 2H), 7.24 – 7.17 (m, 2H), 4.10 – 4.01 (m, 1H), 3.23 – 3.29 (m, 1H), 3.04 – 2.91 (m, 1H), 2.60 (s, 1H), 2.45 (s, 3H), 2.21 – 2.07 (m, 1H), 1.97 – 1.86 (m, 1H), 1.86 – 1.76 (m, 1H), 1.70 – 1.56 (m, 1H). **^{13}C NMR** (101 MHz, $CDCl_3$) δ 141.6, 136.6, 127.2, 127.0, 62.2, 46.9, 34.3, 25.5, 16.2. **HRMS** (ESI/QTOF) *m/z*: $[M + H]^+$ Calcd for $C_{11}H_{16}NS^+$ 194.0998; Found 194.0994. **IR** (ATR): 2961, 2919, 2867, 1492, 1397, 1093, 814, 526 cm^{-1}.

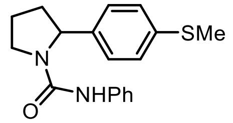

2-(4-(Methylthio)phenyl)-*N*-phenylpyrrolidine-1-carboxamide (*rac*-9): The title compound was synthesized according to **GP-2** from 2-(4-(methylthio)phenyl)pyrrolidine (400 mg, 2.06 mmol). The product was purified by precipitation from hexane/Et_2O (5:1) (496 mg, 1.59 mmol, 77% yield). pink solid.

M.p. = 103 – 105 °C (hexane/Et_2O). **^{1}H NMR** (400 MHz, $CDCl_3$) δ 7.29 – 7.23 (m, 4H), 7.20 – 7.13 m, 4H), 6.98 – 6.92 (m, 1H), 6.05 (s, 1H), 4.81 – 4.87 (m, 1H), 3.86 – 3.68 (m, 2H), 2.49 (s, 3H), 2.48 – 2.36 (m, 1H), 2.06 – 1.85 (m, 3H). **^{13}C NMR** (101 MHz, $CDCl_3$) δ 154.3, 139.6, 139.1, 138.3, 128.9, 127.3, 126.5, 122.9, 119.4, 61.0, 47.6, 36.9, 23.3, 15.9. **HRMS** (ESI/QTOF) *m/z*: $[M + H]^+$ Calcd for $C_{18}H_{21}N_2OS^+$ 313.1369; Found 313.1368. **IR** (ATR) 3308, 2971, 1875, 1703, 1647, 1595, 1441, 1367, 1298, 920, 752, 693 cm^{-1}.

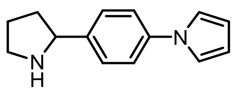

1-(4-(Pyrrolidin-2-yl)phenyl)-1*H*-pyrrole (*rac*-10a): The title compound was synthesized according to **GP-1** from 1-(4-bromophenyl)-1*H*-pyrrole (1.66 mg, 7.5 mmol). The residue was

purified by flash column chromatography on silica gel (9:1, EtOAc/MeOH then 9:1:0.1, EtOAc/MeOH/*i*$PrNH_2$) (387 mg, 1.82 mmol, 52% yield). Yellow solid.
M.p. = 78 – 80 °C (EtOAc/MeOH/*i*$PrNH_2$). **^{1}H NMR** (400 MHz, $CDCl_3$) δ 7.48 – 7.41 (m, 2H), 7.38 – 7.31 (m, 2H), 7.07 (t, J = 2.2 Hz, 2H), 6.34 (t, J = 2.2 Hz, 2H), 4.34 (s, 1H), 4.25 – 4.13 (m, 1H), 3.28 – 3.17 (m, 1H), 3.12 – 2.98 (m, 1H), 2.29 – 2.18 (m, 1H), 2.05 – 1.94 (m, 1H), 1.94 – 1.87 (m, 1H), 1.82 – 169 (m, 1H). **^{13}C NMR** (101 MHz, $CDCl_3$) δ 140.8 (d, J = 20.1 Hz), 139.9 (d, J = 2.4 Hz), 128.1 (d, J = 2.9 Hz), 120.6, 119.4, 110.4, 62.2, 48.0 – 45.0 (m), 34.1 (d, J = 5.1 Hz), 25.4 (d, J = 2.9 Hz). **HRMS** (ESI/QTOF) *m/z*: $[M + H]^+$ Calcd for $C_{14}H_{17}N_2^+$ 213.1386; Found 213.1394. **IR** (ATR) 2960, 2870, 1612, 1521, 1480, 1398, 1327, 1069, 829, 724 cm^{-1}.

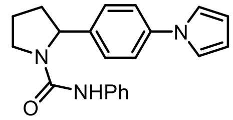

2-(4-(1*H*-pyrrol-1-yl)phenyl)-*N*-phenylpyrrolidine-1-carboxamide (*rac*-10): The title compound was synthesized according to **GP-2** from 1-(4-(pyrrolidin-2-yl)phenyl)-1*H*-pyrrole (250 mg, 1.17 mmol). The product was purified by flash column chromatography on silica gel (3:1, 2:1 then 1:1, Hex/EtOAc) (332 mg, 1.00 mmol, 85% yield). White solid.
M.p. = 60 – 62 °C (hexane/Et_2O). **^{1}H NMR** (400 MHz, $CDCl_3$) δ 7.45 – 7.33 (m, 4H), 7.21 (d, J = 4.6 Hz, 4H), 7.09 (t, J = 2.3 Hz, 2H), 7.02 – 6.92 (m, 1H), 6.36 (t, J = 2.3 Hz, 2H), 6.11 (s, 1H), 5.00 – 4.90 (m, 1H), 3.89 – 3.68 (m, 2H), 2.54 – 2.39 (m, 1H), 2.12 – 1.88 (m, 3H). **^{13}C NMR** (101 MHz, $CDCl_3$) δ 154.3, 140.4, 140.2, 139.0, 128.9, 127.1, 123.0, 121.1, 119.5, 119.4, 110.7, 60.9, 47.6, 36.7, 23.4. **HRMS** (ESI/QTOF) *m/z*: $[M + H]^+$ Calcd for $C_{21}H_{22}N_3O^+$ 332.1757; Found 332.1757. **IR** (ATR): 3317, 2971, 2872, 1649, 1611, 1519, 1441, 1368, 1327, 1243, 1070, 725 cm^{-1}.

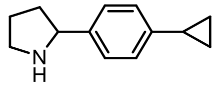

2-(4-Cyclopropylphenyl)pyrrolidine (*rac*-11a): The title compound was synthesized according to **GP-1** from 1-bromo-4-cyclopropylbenzene (1.48 g, 7.5 mmol). The residue was purified by flash column chromatography on silica gel (EtOAc/MeOH/ *i*$PrNH_2$, 9:1:0.1) (421 mg, 2.25 mmol, 45% yield). Yellow oil.
^{1}H NMR (400 MHz, $CDCl_3$) δ 7.29 – 7.24 (m, 2H), 7.07 – 7.00 (m, 2H), 4.16 – 4.08 (m, 1H), 3.91 (s, 1H), 3.23 – 3.17 (m, 1H), 3.04 – 2.96 (m, 1H), 2.23 – 2.15 (m, 1H), 1.99 – 1.94 (m, 1H), 1.90 – 1.82 (m, 2H), 1.80 – 1.66 (m, 1H), 0.97 – 0.90 (m, 2H), 0.71 – 0.64 (m, 2H). **^{13}C NMR** (101 MHz, $CDCl_3$) δ 143.0, 140.1, 126.7, 125.8, 62.5, 46.6, 33.9, 25.3, 15.1, 9.2. **HRMS** (ESI/QTOF) *m/z*: $[M + H]^+$ Calcd for $C_{13}H_{18}N^+$ 188.1434; Found 188.1431 **IR** (ATR): 3060, 2960, 1492, 1043, 819, 761, 699 cm^{-1}.

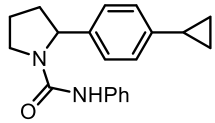

2-(4-Cyclopropylphenyl)-*N*-phenylpyrrolidine-1-carboxamide (*rac*-11): The title compound was synthesized according to **GP-2** from 2-(4-cyclopropylphenyl)pyrrolidine (300 mg, 1.60 mmol). The residue was purified by flash column chromatography on silica gel (Hex/EtOAc, 3:1, 2:1 then 1:1). (424 mg, 1.38 mmol, 86% yield). White solid.

M.p. = 49 – 51 °C (hexane/Et_2O). **^{1}H NMR** (400 MHz, $CDCl_3$) δ 7.23 – 7.17 (m, 3H), 7.17 – 7.12 (m, 3H), 7.11 – 7.06 (m, 2H), 6.97 – 6.91 (m, 1H), 6.07 (s, 1H), 4.84 – 4.76 (m, 1H), 3.87 – 3.78 (m, 1H), 3.77 – 3.68 (m, 1H), 2.48 – 2.38 (m, 1H), 2.02 – 1.85 (m, 4H), 1.01 – 0.94 (m, 2H), 0.73 – 0.67 (m, 2H). **^{13}C NMR** (101 MHz, $CDCl_3$) δ 154.4, 144.0, 139.7, 139.2, 128.8, 126.6, 126.0, 122.7, 119.3, 61.2, 47.6, 37.2, 23.3, 15.3, 9.5. **HRMS** (ESI/QTOF) *m/z*: $[M + Na]^+$ Calcd for $C_{20}H_{22}N_2NaO^+$ 329.1624; Found 329.1623. **IR** (ATR): 3320, 2970, 2872, 1650, 1531, 1441, 1368, 1243, 1176, 752, 692 cm^{-1}.

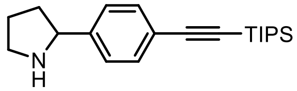

2-(4-((Triisopropylsilyl)ethynyl)phenyl)pyrrolidine (*rac*-12a): The title compound was synthesized according to **GP-1** from ((4-bromophenyl)ethynyl)triisopropylsilane (1.00 g, 2.96 mmol) and pyrrolidine (156 mg, 2.2 mmol, 1.0 equiv) The residue was purified by flash column chromatography on silica gel (9:1, EtOAc/MeOH then 9:1:0.1, EtOAc/MeOH/*i*PrNH$_2$). (197 mg, 0.60 mmol, 27% yield). Yellow oil.
^{1}H NMR (400 MHz, $CDCl_3$) δ 7.46 – 7.39 (m, 2H), 7.32 – 7.27 (m, 2H), 4.11 (t, J = 7.7 Hz, 1H), 3.23 – 3.14 (m, 1H), 3.06 – 2.97 (m, 1H), 2.23 – 2.10 (m, 1H), 1.98 – 1.78 (m, 3H), 1.64 – 1.52 (m, 3H), 1.12 (s, 18H). **^{13}C NMR** (101 MHz, $CDCl_3$) δ 145.6, 132.2, 126.5, 122.0, 107.3, 90.1, 62.5, 47.2, 34.6, 25.7, 18.8, 11.5. **HRMS** (ESI/QTOF) *m/z*: $[M + H]^+$ Calcd for $C_{21}H_{34}NSi^+$ 328.2455; Found 328.2457. **IR** (ATR) 2941, 2890, 2863, 2153, 1459, 1382, 994, 881, 830, 674, 663 cm^{-1}.

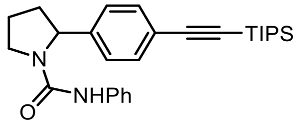

***N*-Phenyl-2-(4-((triisopropylsilyl)ethynyl)phenyl)pyrrolidine-1-carboxamide (*rac*-12)**: The title compound was synthesized according to **GP-2** from 2-(4-((triisopropylsilyl)ethynyl)phenyl)pyrrolidine (169 mg, 0.457 mmol). The product was purified by flash column chromatography on silica gel (2:1→1:1, hexane/Et_2O, then 1:1, hexane/EtOAc). (174 mg, 0.356 mmol, 78% yield). White solid.
M.p. = 69 – 71 °C (hexane/EtOAc). ^{1}H **NMR** (400 MHz, $CDCl_3$) δ 7.52 – 7.46 (m, 2H), 7.28 – 7.24 (m, 2H), 7.23 – 7.16 (m, 4H), 6.99 – 6.93 (m, 1H), 6.00 (s, 1H), 4.89 (dd, J = 8.0, 3.7 Hz, 1H), 3.86 – 3.68 (m, 2H), 2.51 – 2.38 (m, 1H), 2.02 – 1.84 (m, 3H), 1.13 (s, 21H). **^{13}C NMR** (101 MHz, $CDCl_3$) δ 154.3, 143.1, 139.0, 132.9, 128.9, 125.8, 123.3, 123.0, 119.5, 106.7, 91.3, 61.2, 47.6, 36.8, 23.3, 18.8, 11.5. **HRMS** (ESI/QTOF) *m/z*: $[M + Na]^+$ Calcd for $C_{28}H_{38}N_2NaOSi^+$ 469.2646; Found 469.2644. **IR** (ATR): 3315, 2941, 2863, 2154, 1647, 1595, 1532, 1442, 1369, 1219 882, 830, 752 cm^{-1}.

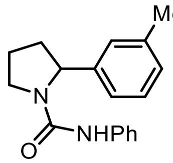

***N*-Phenyl-2-(*m*-tolyl)pyrrolidine-1-carboxamide (*rac*-13)**: The title compound was synthesized according to **GP-2** from 2-(*m*-tolyl)pyrrolidine (250 mg, 1.41 mmol). The product was purified by

flash column chromatography on silica gel (4:1→1:1, pentane/EtOAc). (354 mg, 1.26 mmol, 81% yield). White solid.

M.p. = 151 – 153 °C (pentane/EtOAc). **^{1}H NMR** (400 MHz, $CDCl_3$) δ 7.32 – 7.26 (m, 1H), 7.21 – 7.15 (m, 2H), 7.12 (d, *J* = 7.3 Hz, 5H), 6.94 (tt, *J* = 6.9, 1.4 Hz, 1H), 6.05 (s, 1H), 4.81 (dd, *J* = 7.8, 4.1 Hz, 1H), 3.90 – 3.80 (m, 1H), 3.80 – 3.69 (m, 1H), 2.54 – 2.40 (m, 1H), 2.37 (s, 3H), 2.08 – 1.97 (m, 1H), 1.97 – 1.87 (m, 2H). **^{13}C NMR** (101 MHz, $CDCl_3$) δ 154.4, 142.8, 139.2, 139.2, 129.2, 128.9, 128.8, 126.6, 123.1, 122.8, 119.4, 61.5, 47.7, 37.2, 23.3, 21.7. **HRMS** (ESI/QTOF) *m/z*: $[M + H]^+$ Calcd for $C_{18}H_{21}N_2O^+$ 281.1648; Found 281.1660. **IR** (ATR): 3317, 2970, 2946, 2872, 1646, 1594, 1528, 1440, 1366, 1301, 1241, 750, 692 cm^{-1}.

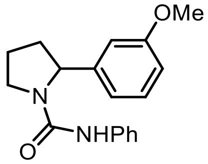

2-(3-Methoxyphenyl)-*N*-phenylpyrrolidine-1-carboxamide (*rac*-14): The title compound was synthesized according to **GP-2** from 2-(3-methoxyphenyl)pyrrolidine (250 mg, 1.55 mmol). The product was purified by flash column chromatography on silica gel (4:1→1:1, pentane/EtOAc). (335 mg, 1.13 mmol, 80% yield). White solid.

M.p. = 96 – 98 °C (pentane/EtOAc). **^{1}H NMR** (400 MHz, $CDCl_3$) δ 7.35 – 7.27 (m, 1H), 7.23 – 7.12 (m, 4H), 6.98 – 6.89 (m, 2H), 6.88 – 6.81 (m, 2H), 6.09 (s, 1H), 4.83 (dd, *J* = 8.0, 3.7 Hz, 1H), 3.86 – 3.76 (m, 1H), 3.87 (s, 3H), 3.76 (m, 1H), 2.51 – 2.35 (m, 1H), 2.08 – 1.83 (m, 3H). **^{13}C NMR** (101 MHz, $CDCl_3$) δ 160.4, 154.4, 144.6, 139.2, 130.4, 128.8, 122.8, 119.4, 118.2, 113.2, 111.7, 61.4, 55.4, 47.6, 36.9, 23.3. **HRMS** (ESI/QTOF) *m/z*: $[M + Na]^+$ Calcd for $C_{18}H_{20}N_2NaO_2^+$ 319.1417; Found 319.1415. **IR** (ATR): 3316, 2945, 2873, 2834, 1645, 1594, 1438, 1364, 1238, 1046, 750, 693 cm^{-1}.

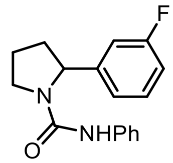

2-(3-Fluorophenyl)-*N*-phenylpyrrolidine-1-carboxamide (*rac*-15): The title compound was synthesized according to **GP-2** from 2-(3-fluorophenyl)pyrrolidine (250 mg, 1.51 mmol). The product was purified by precipitation from hexane/Et_2O (5:1) (366 mg, 1.29 mmol, 85% yield). white solid.

M.p. = 126 – 128 °C (hexane/Et_2O). **^{1}H NMR** (400 MHz, $CDCl_3$) δ 7.35 (td, *J* = 7.9, 5.7 Hz, 1H), 7.25 – 7.15 (m, 4H), 7.10 (d, *J* = 7.9 Hz, 1H), 7.05 – 6.92 (m, 3H), 6.06 (s, 1H), 4.94 (dd, *J* = 8.2, 3.8 Hz, 1H), 3.86 – 3.66 (m, 2H), 2.51 – 2.38 (m, 1H), 2.09 – 1.85 (m, 3H). **^{13}C NMR** (101 MHz, $CDCl_3$) δ 163.4 (d, *J* = 247.2 Hz), 154.2, 145.9 (d, *J* = 6.3 Hz), 139.0, 130.8 (d, *J* = 8.3 Hz), 128.9, 123.1, 121.5 (d, *J* = 2.9 Hz), 119.5, 114.8 (d, *J* = 21.3 Hz), 112.9 (d, *J* = 22.0 Hz), 61.0 (d, *J* = 1.9 Hz), 47.5, 36.4, 23.4. **^{19}F NMR** (376 MHz, $CDCl_3$) δ –111.9. **HRMS** (ESI/QTOF) *m/z*: $[M + H]^+$ Calcd for $C_{17}H_{18}FN_2O^+$ 285.1398; Found 285.1401. **IR** (ATR): 3313, 2973, 1647, 1614, 1594, 1441, 1368, 1301, 751, 692 cm^{-1}.

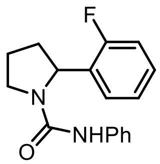

2-(2-Fluorophenyl)-*N*-phenylpyrrolidine-1-carboxamide (*rac*-16): The title compound was synthesized according to **GP-2** from 2-(2-fluorophenyl)pyrrolidine (250 mg, 1.51 mmol). The product was purified by precipitation from hexane/Et_2O (5:1) and by flash column chromatography (3:1, hexane/EtOAc) (314 mg, 1.10 mmol, 73% yield). White solid.
M.p. = 126 – 128 °C (hexane/Et_2O). **^{1}H NMR** (400 MHz, $CDCl_3$) δ 7.31 – 7.23 (m, 4H), 7.23 – 7.17 (m, 2H), 7.17 – 7.05 (m, 2H), 6.97 (tt, *J* = 7.0, 1.5 Hz, 1H), 6.11 (s, 1H), 5.30 – 5.09 (m, 1H), 3.75 (t, *J* = 6.7 Hz, 2H), 2.52 – 2.34 (m, 1H), 2.04 – 1.89 (m, 3H). **^{13}C NMR** (101 MHz, $CDCl_3$) δ 160.3 (d, *J* = 246.1 Hz), 154.3, 139.3, 130.0 (d, *J* = 13.0 Hz), 129.6 (q, *J* = 8.1 Hz), 129.2, 127.6 (d, *J* = 4.1 Hz), 124.9 (d, *J* = 3.5 Hz), 123.3, 119.9, 116.2 (d, *J* = 21.2 Hz), 55.9 (d, *J* = 3.0 Hz), 47.6, 35.1, 23.7. **^{19}F NMR** (376 MHz, $CDCl_3$) δ –118.1. **HRMS** (ESI/QTOF) *m/z*: $[M + H]^+$ Calcd for $C_{17}H_{18}FN_2O^+$ 285.1398; Found 285.1403. **IR** (ATR) 3314, 2975, 2874, 1649, 1532, 1442, 1372, 1201, 904, 754, 692 cm^{-1}.

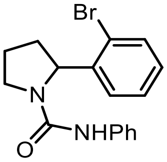

2-(2-Bromophenyl)-*N*-phenylpyrrolidine-1-carboxamide (*rac*-17): The title compound was synthesized according to **GP-2** from 2-(2-bromophenyl)pyrrolidine (250 mg, 1.11 mmol). The product was purified by flash column chromatography on silica gel (4:1→1:1, pentane/EtOAc). (359 mg, 1.04 mmol, 94% yield). White foam.
M.p. = 82 – 84 °C (pentane/EtOAc). **^{1}H NMR** (400 MHz, $CDCl_3$) δ 7.61 (dd, *J* = 7.9, 1.1 Hz, 1H), 7.35 – 7.27 (m, 2H), 7.25 – 7.20 (m, 3H), 7.20 – 7.13 (m, 2H), 6.97 (tt, *J* = 6.6, 1.9 Hz, 1H), 5.96 (s, 1H), 5.26 (dd, *J* = 8.1, 2.7 Hz, 1H), 3.85 – 3.75 (m, 2H), 2.59 – 2.42 (m, 1H), 2.03 – 1.87 (m, 3H). **^{13}C NMR** (101 MHz, $CDCl_3$) δ 154.0, 141.4, 139.0, 133.5, 129.4, 128.9, 128.2, 127.3, 123.0, 122.1, 119.6, 60.9, 47.8, 34.7, 23.0. **HRMS** (ESI/QTOF) *m/z*: $[M + H]^+$ Calcd for $C_{17}H_{18}BrN_2O^+$ 345.0597; Found 345.0595. **IR** (ATR) 3314, 1646, 1594, 1526, 1439, 1363, 1243, 1045, 881, 748, 692 cm^{-1}.

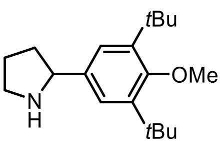

2-(3,5-Di-*tert*-butyl-4-methoxyphenyl)pyrrolidine (*rac*-18a): The title compound was synthesized according to **GP-1** from 5-bromo-1,3-di-*tert*-butyl-2-methoxybenzene (3.74 g, 12.5 mmol). The residue was purified by flash column chromatography on silica gel (EtOAc/MeOH/*i*$PrNH_2$, 9:1:0.1) (789 mg, 2.73 mmol, 55% yield). Yellow oil.
M.p. = 38 – 40 °C (hexane/Et_2O). **^{1}H NMR** (400 MHz, $CDCl_3$) 7.23 (s, 2H), 4.03 (dd, *J* = 8.7, 6.9 Hz, 1H), 3.68 (s, 3H), 3.25 – 3.16 (m, 1H), 3.03 – 2.92 (m, 1H), 2.47 (s, 1H), 2.23 – 2.10 (m, 1H), 2.00 – 1.78 (m, 2H), 1.76 – 1.62 (m, 1H), 1.43 (s, 18H). **^{13}C NMR** (101 MHz, $CDCl_3$) δ 158.5, 143.5, 137.8, 124.9, 64.3, 63.2, 47.0, 36.0, 34.1, 32.3, 25.7. **HRMS** (ESI/QTOF) *m/z*: $[M + H]^+$

Calcd for $C_{19}H_{32}NO^+$ 290.2478; Found 290.2465. **IR** (ATR): 2954, 1447, 1411, 1215, 1114, 1012, 878 cm^{-1}.

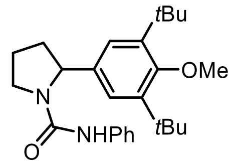

2-(3,5-Di-*tert*-butyl-4-methoxyphenyl)-*N*-phenylpyrrolidine-1-carboxamide (*rac*-**18**): The title compound was synthesized according to **GP-2** from 2-(3,5-di-*tert*-butyl-4-methoxyphenyl)pyrrolidine (433 mg, 1.45 mmol). The product was purified by precipitation from hexane/Et_2O (5:1) (422 mg, 1.03 mmol, 69% yield). White solid.
M.p. = 149 – 151 °C (hexane/Et_2O). **^{1}H NMR** (400 MHz, $CDCl_3$) δ 7.20 – 7.14 (m, 4H), 7.08 – 7.02 (m, 2H), 6.90 – 6.96 (m, 1H), 6.06 (s, 1H), 4.65 – 4.74 (m, 1H), 3.92 – 3.84 (m, 1H), 3.76 – 3.71 (m, 1H), 3.70 (s, 3H), 2.51 – 2.39 (m, 1H), 2.07 – 1.85 (m, 3H), 1.42 (s, 18H). **^{13}C NMR** (101 MHz, $CDCl_3$) δ 159.7, 155.0, 145.2, 139.7, 136.6, 129.2, 124.6, 123.0, 119.6, 64.8, 62.2, 48.0, 37.8, 36.3, 32.5, 32.5, 23.8. **HRMS** (ESI/QTOF) *m/z*: $[M + H]^+$ Calcd for $C_{26}H_{37}N_2O_2^+$ 409.2850; Found 409.2839. **IR** (ATR): 3322, 2959, 2870 1649, 1595, 1529, 1441, 1361, 1243, 1114, 751, 658 cm^{-1}.

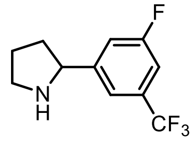

2-(3-Fluoro-5-(trifluoromethyl)phenyl)pyrrolidine (*rac*-**19a**)**:** The title compound was synthesized according to **GP-1** from 1-bromo-3-fluoro-5-(trifluoromethyl)benzene (1.82 g, 7.5 mmol). The residue was purified by flash column chromatography on silica gel (EtOAc/MeOH 9:1) (325 mg, 1.40 mmol, 28% yield). Yellow oil.
^{1}H NMR (400 MHz, $CDCl_3$) δ 7.43 (s, 1H), 7.31 (dt, *J* = 9.7, 2.0 Hz, 1H), 7.16 (dt, *J* = 8.4, 2.1 Hz, 1H), 4.21 (t, *J* = 7.7 Hz, 1H), 3.22 – 3.13 (m, 1H), 3.11 – 3.00 (m, 1H), 2.30 – 2.18 (m, 2H), 1.98 – 1.78 (m, 2H), 1.68 – 1.55 (m, 1H).**^{13}C NMR** (101 MHz, $CDCl_3$) δ 162.7 (d, *J* = 247.9 Hz), 149.8 (d, *J* = 6.9 Hz), 132.4 (qd, *J* = 32.9, 8.2 Hz), 123.6 (dq, *J* = 272.4, 3.1 Hz), 119.2 (p, *J* = 3.7 Hz), 117.1 (d, *J* = 21.5 Hz), 111.1 (dq, *J* = 24.8, 3.8 Hz), 61.6 (d, *J* = 1.9 Hz), 47.1, 34.8, 25.6. **^{19}F NMR** (376 MHz, $CDCl_3$) δ –62.7, –111.4. **HRMS** (ESI/QTOF) *m/z*: $[M + H]^+$ Calcd for $C_{11}H_{12}F_4N^+$ 234.0900; Found 234.0897. **IR** (ATR): 2967, 2874, 1603, 1451, 1342, 1226, 1122, 870, 669 cm^{-1}.

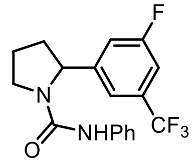

2-(3-Fluoro-5-(trifluoromethyl)phenyl)-*N*-phenylpyrrolidine-1-carboxamide (*rac*-**19**): The title compound was synthesized according to **GP-2** from 2-(3-fluoro-5-(trifluoromethyl)phenyl)pyrrolidine (280 mg, 1.20 mmol). The product was purified by precipitation from hexane/Et_2O (389 mg, 1.10 mmol, 92% yield). White solid.
M.p. = 160 – 162 °C (hexane/Et_2O). **^{1}H NMR** (400 MHz, $CDCl_3$) δ 7.38 – 7.31 (m, 3H), 7.31 – 7.27 (m, 2H), 7.26 – 7.17 (m, 2H), 7.07 – 7.01 (m, 1H), 6.24 (s, 1H), 5.14 (dd, *J* = 8.1, 3.5 Hz,

1H), 3.83 – 3.67 (m, 2H), 2.51 – 2.38 (m, 1H), 2.10 – 2.00 (m, 2H), 1.96 – 1.88 (m, 1H). **^{13}C NMR** (101 MHz, $CDCl_3$) δ 162.8 (d, J = 249.5 Hz), 154.0, 147.6 (d, J = 6.5 Hz), 138.6, 132.9 (dq, J = 33.4, 8.2 Hz), 128.9, 123.3 123.2 (dq, J = 272.3, 3.4 Hz) 119.7, 118.2 (p, J = 3.7 Hz), 116.2 (d, J = 22.1 Hz), 111.7 (dd, J = 24.7, 3.9 Hz), 60.6 (d, J = 1.8 Hz), 47.3, 35.3, 23.7. **^{19}F NMR** (376 MHz, $CDCl_3$) δ –62.7, –110.1. **HRMS** (ESI/QTOF) *m/z*: $[M + Na]^+$ Calcd for $C_{18}H_{16}F_4N_2NaO^+$ 375.1091; Found 375.1089. **IR** (ATR): 3313, 2954, 1647, 1533, 1443, 1351, 1226, 1169, 1128, 752, 693 cm^{-1}.

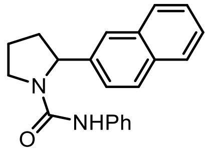

2-(Naphthalen-2-yl)-*N*-phenylpyrrolidine-1-carboxamide (*rac*-20): The title compound was synthesized according to **GP-2** from 2-(naphthalen-2-yl)pyrrolidine (342 mg, 1.73 mmol). The product was purified by precipitation from hexane/Et_2O. (442 mg, 1.40 mmol, 81% yield). Pale-yellow solid.

M.p. = 149 – 151 °C (hexane/Et_2O). ^{1}H **NMR** (400 MHz, $CDCl_3$) δ 7.89 (d, J = 8.5 Hz, 1H), 7.87 – 7.81 (m, 2H), 7.78 (s, 1H), 7.55 – 7.46 (m, 2H), 7.43 (dd, J = 8.5, 2.0 Hz, 1H), 7.20 – 7.06 (m, 4H), 6.96 – 6.87 (m, 1H), 6.15 (s, 1H), 5.05 (dd, J = 7.9, 3.7 Hz, 1H), 3.98 – 3.74 (m, 2H), 2.61 – 2.42 (m, 1H), 2.12 – 1.87 (m, 3H). **^{13}C NMR** (101 MHz, $CDCl_3$) δ 154.5, 140.1, 139.1, 133.5, 133.2, 129.5, 128.8, 128.0, 127.9, 126.8, 126.4, 124.6, 124.0, 122.8, 119.4, 61.6, 47.8, 36.9, 23.3. **HRMS** (ESI/QTOF) *m/z*: $[M + H]^+$ Calcd for $C_{21}H_{21}N_2O^+$ 317.1648; Found 317.1652. **IR** (ATR) 3320, 3053, 2969, 2876, 1650, 1595, 1530, 1441, 1363, 1270, 896, 749, 692 cm^{-1}.

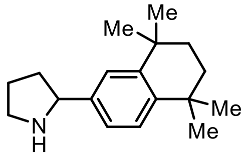

2-(5,5,8,8-Tetramethyl-5,6,7,8-tetrahydronaphthalen-2-yl)pyrrolidine (*rac*-21a): The title compound was synthesized according to **GP-1** from pyrrolidine (356 mg, 5 mmol). The residue was purified by flash column chromatography on silica gel (9:1, EtOAc/MeOH then 3:1, EtOAc/MeOH) (543 mg, 2.11 mmol, 42% yield). Yellow solid.

M.p. = 66 – 68 °C (EtOAc/MeOH). **^{1}H NMR** (400 MHz, $CDCl_3$) δ 7.29 (d, J = 2.0 Hz, 1H), 7.28 – 7.24 (d, 1H), 7.13 (dd, J = 8.1, 2.0 Hz, 1H), 4.06 (t, J = 7.7 Hz, 1H), 3.25 – 3.16 (m, 1H), 3.03 – 2.95 (m, 1H), 2.82 (s, 1H), 2.24 – 2.09 (m, 1H), 2.02 – 1.79 (m, 2H), 1.68 (s, 4H), 1.28 (s, 6H), 1.27 (s, 6H). **^{13}C NMR** (101 MHz, $CDCl_3$) δ 144.9, 143.6, 141.2, 141.1, 126.7, 125.0, 123.9, 62.9, 46.99, 46.98, 35.4, 35.2, 34.4, 34.2, 34.0, 32.03, 32.01, 25.7. **HRMS** (ESI/QTOF) *m/z*: $[M + H]^+$ Calcd for $C_{18}H_{28}N^+$ 258.2216; Found 258.2216. **IR** (ATR): 2957, 2923, 2860, 1493, 1457, 1362, 1067, 824, 538 cm^{-1}.

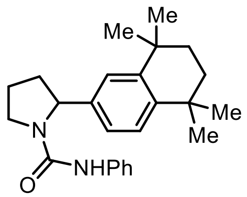

***N*-Phenyl-2-(5,5,8,8-tetramethyl-5,6,7,8-tetrahydronaphthalen-2-yl)pyrrolidine-1-carboxamide (*rac*-21)**: The title compound was synthesized according to **GP-2** from 2-(5,5,8,8-tetramethyl-5,6,7,8-tetrahydronaphthalen-2-yl)pyrrolidine (300 mg, 1.17 mmol). The product was purified by precipitation from hexane/Et_2O (5:1) (337 mg, 0.90 mmol, 77% yield). white solid.
M.p. = 155 – 157 °C (hexane/Et_2O). **^{1}H NMR** (400 MHz, $CDCl_3$) δ 7.32 (d, *J* = 8.1 Hz, 1H), 7.23 (d, *J* = 2.1 Hz, 1H), 7.20 – 7.12 (m, 2H), 7.09 – 7.02 (m, 3H), 6.93 (tt, *J* = 7.0, 1.3 Hz, 1H), 6.04 (s, 1H), 4.78 – 4.70 (m, 1H), 3.91 – 3.80 (m, 1H), 3.80 – 3.65 (m, 1H), 2.52 – 2.38 (m, 1H), 2.08 – 1.82 (m, 3H), 1.69 (s, 4H), 1.30 (s, 3H), 1.29 (s, 3H), 1.28 (s, 3H), 1.24 (s, 3H). **^{13}C NMR** (101 MHz, $CDCl_3$) δ 154.6, 146.0, 144.9, 139.5, 139.3, 128.8, 127.8, 124.1, 123.4, 122.7, 119.4, 61.7, 47.7, 37.3, 35.2, 35.1, 34.5, 34.3, 32.2, 32.0, 32.0, 23.4. **HRMS** (ESI/QTOF) *m/z*: $[M + H]^+$ Calcd for $C_{25}H_{33}N_2O^+$ 377.2587; Found 377.2592. **IR** (ATR):3319, 2959, 2864, 1650, 1596, 1531, 1441, 1363, 1190, 751, 692 cm^{-1}.

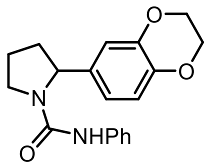

2-(2,3-Dihydrobenzo[*b*][1,4]dioxin-6-yl)-*N*-phenylpyrrolidine-1-carboxamide (*rac*-22): The title compound was synthesized according to **GP-2** from 2-(2,3-dihydrobenzo[*b*][1,4]dioxin-6-yl)pyrrolidine (250 mg, 1.22 mmol). The product was purified by flash column chromatography on silica gel (3:1→1:1, pentane/EtOAc). (330 mg, 1.02 mmol, 84% yield). White solid.
M.p. = 146 – 148 °C (hexane/Et_2O). **^{1}H NMR** (400 MHz, $CDCl_3$) δ 7.20 (s, 2H), 7.19 (s, 2H), 6.99 – 6.91 (m, 1H), 6.87 (d, *J* = 8.1 Hz, 1H), 6.84 – 6.76 (m, 2H), 6.13 (s, 1H), 4.75 (dd, *J* = 7.9, 4.1 Hz, 1H), 4.25 (s, 4H), 3.84 – 3.74 (m, 1H), 3.74 – 3.65 (m, 1H), 2.47 – 2.30 (m, 1H), 2.05 – 1.94 (dd, 1H), 1.95 – 1.84 (m, 2H). **^{13}C NMR** (101 MHz, $CDCl_3$) δ 154.4, 144.2, 143.3, 139.3, 136.1, 128.8, 122.7, 119.3, 118.9, 118.0, 114.8, 64.48, 64.43, 60.9, 47.5, 37.1, 23.3. **HRMS** (ESI/QTOF) *m/z*: $[M + Na]^+$ Calcd for $C_{19}H_{20}N_2NaO_3^+$ 347.1366; Found 347.1369. **IR** (ATR) 3318, 2873, 1648, 1593, 1528, 1502, 1440, 1365, 1285, 1241, 1067, 920, 752, 730, 692 cm^{-1}.

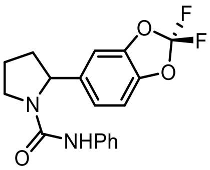

2-(2,2-Difluorobenzo[*d*][1,3]dioxol-5-yl)-*N*-phenylpyrrolidine-1-carboxamide (*rac*-23): The title compound was synthesized according to **GP-2** from 2-(2,2-difluorobenzo[*d*][1,3]dioxol-5-yl)pyrrolidine (250 mg, 1.10 mmol). The product was purified by precipitation from hexane/Et_2O (5:1). (347 mg, 1.00 mmol, 91% yield). white solid.
M.p. = 173 – 175 °C (hexane/Et_2O). **^{1}H NMR** (400 MHz, $CDCl_3$) δ 7.30 – 7.25 (m, 2H), 7.24 – 7.18 (m, 2H), 7.06 – 6.93 (m, 4H), 6.18 (s, 1H), 4.97 (dd, *J* = 7.9, 3.8 Hz, 1H), 3.71 (t, *J* = 6.7 Hz, 2H), 2.46 – 2.30 (m, 1H), 2.06 – 1.91 (m, 2H), 1.91 – 1.81 (m, 1H). **^{13}C NMR** (101 MHz, $CDCl_3$)

δ 154.1, 144.4, 143.1, 139.8, 138.9, 131.8, 128.9, 123.1, 120.8, 119.6, 109.7, 107.2, 61.0, 47.4, 36.3, 23.5. **^{19}F NMR** (376 MHz, $CDCl_3$) δ –49.81, –49.84. **HRMS** (ESI/QTOF) *m/z*: $[M + H]^+$ Calcd for $C_{18}H_{17}F_2N_2O_3^+$ 347.1202; Found 347.1202. **IR** (ATR) 3314, 2974, 2876, 1645, 1595, 1527, 1494, 1440, 1359, 1323, 1229, 1136, 1033, 902, 751, 700 cm^{-1}.

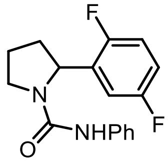

2-(2,5-Difluorophenyl)-*N*-phenylpyrrolidine-1-carboxamide (*rac*-24): The title compound was synthesized according to **GP-2** from 2-(2,5-difluorophenyl)pyrrolidine (250 mg, 1.37 mmol). The product was purified by flash column chromatography on silica gel (2:1→1:1, pentane/EtOAc). (382 mg, 1.26 mmol, 93% yield). Colorless gum.

M.p. = 123 – 125 °C (pentane/EtOAc). **^{1}H NMR** (400 MHz, $CDCl_3$) δ 7.35 – 7.29 (m, 2H), 7.28 – 7.20 (m, 2H), 7.07 – 6.97 (m, 2H), 6.97 – 6.86 (m, 2H), 6.18 (s, 1H), 5.34 – 5.17 (m, 1H), 3.80 – 3.59 (m, 2H), 2.51 – 2.34 (m, 1H), 2.09 – 1.86 (m, 3H). **^{13}C NMR** (101 MHz, $CDCl_3$) δ 159.1 (dd, *J* = 242.9, 2.2 Hz), 155.8 (dd, *J* = 241.7, 2.5 Hz), 153.9, 138.8, 132.1 (d, *J* = 17.3 Hz), 129.0, 123.2, 119.7 (d, *J* = 2.0 Hz), 116.9 (dd, *J* = 24.3, 8.5 Hz), 115.3 (dd, *J* = 24.5, 8.4 Hz), 113.9 (dd, *J* = 25.1, 4.7 Hz), 55.6 (d, *J* = 2.6 Hz), 47.2, 34.2, 23.7. **^{19}F NMR** (376 MHz, $CDCl_3$) δ –118.0, –124.2 (d, *J* = 18.0 Hz). **HRMS** (ESI/QTOF) *m/z*: $[M + H]^+$ Calcd for $C_{17}H_{17}F_2N_2O^+$ 303.1303; Found 303.1308. **IR** (ATR) 3308, 2974, 2876, 1645, 1595, 1530, 1487, 1442, 1363, 1241, 1177, 813, 730, 692 cm^{-1}.

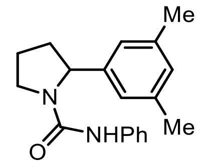

2-(3,5-Dimethylphenyl)-*N*-phenylpyrrolidine-1-carboxamide (*rac*-25): The title compound was synthesized according to **GP-2** from 2-(3,5-dimethylphenyl)pyrrolidine (267 mg, 1.52 mmol). The product was purified by precipitation from hexane/Et_2O. (365 mg, 1.24 mmol, 81% yield). Pale-yellow solid.

M.p. = 143 – 145 °C (hexane/Et_2O). **^{1}H NMR** (400 MHz, $CDCl_3$) δ 7.22 – 7.15 (m, 2H), 7.16 – 7.09 (m, 2H), 6.99 – 6.90 (m, 4H), 6.09 (s, 1H), 4.75 (dd, *J* = 7.9, 4.0 Hz, 1H), 3.90 – 3.79 (m, 1H), 3.80 – 3.67 (m, 1H), 2.49 – 2.37 (m, 1H), 2.33 (s, 6H), 2.10 – 1.84 (m, 3H). **^{13}C NMR** (101 MHz, $CDCl_3$) δ 154.5, 142.7, 139.3, 139.0, 129.8, 128.8, 123.8, 122.7, 119.4, 61.5, 47.7, 37.2, 23.3, 21.5. **HRMS** (ESI/QTOF) *m/z*: $[M + H]^+$ Calcd for $C_{19}H_{23}N_2O^+$ 295.1805; Found 295.1804. **IR** (ATR): 3321, 2871, 1646, 1594, 1527, 1499, 1439, 1364, 1241, 908, 844, 751, 730, 692 cm^{-1}.

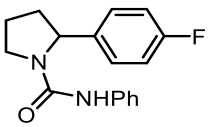

2-(4-Fluorophenyl)-*N*-phenylpyrrolidine-1-carboxamide (*rac*-26): The title compound was synthesized according to **GP-2** from 2-(4-fluorophenyl)pyrrolidine (267 mg, 1.61 mmol). The product was purified by precipitation from hexane/Et_2O. (256 mg, 0.90 mmol, 56% yield). Pale-yellow solid.

M.p. = 134 – 136 °C (hexane/Et_2O). **^{1}H NMR** (400 MHz, $CDCl_3$) δ 7.33 – 7.25 (m, 2H), 7.23 – 7.15 (m, 4H), 7.11 – 7.03 (m, 2H), 6.99 – 6.92 (m, 1H), 6.06 (s, 1H), 4.90 (dd, *J* = 7.8, 3.9 Hz, 1H), 3.86 – 3.66 (m, 2H), 2.53 – 2.32 (m, 1H), 2.07 – 1.83 (m, 3H). **^{13}C NMR** (101 MHz, $CDCl_3$) δ 162.3 (d, *J* = 246.2 Hz), 154.2, 139.0, 138.7 (d, *J* = 3.1 Hz), 128.9, 127.5 (d, *J* = 8.1 Hz), 123.0, 119.4, 116.1 (d, *J* = 21.6 Hz), 60.8, 47.5, 36.8, 23.3. **^{19}F NMR** (376 MHz, $CDCl_3$) δ –114.7. **HRMS** (ESI/QTOF) *m/z*: $[M + H]^+$ Calcd for $C_{17}H_{18}FN_2O^+$ 285.1398; Found 285.1395. **IR** (ATR): 3311, 3055, 2971, 2873, 1644, 1594, 1505, 1439, 1362, 1218, 1154, 906, 830, 750, 731, 692 cm^{-1}.

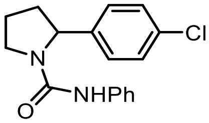

2-(4-Chlorophenyl)-*N*-phenylpyrrolidine-1-carboxamide (*rac*-27): The title compound was synthesized according to **GP-2** from 2-(4-chlorophenyl)pyrrolidine (250 mg, 1.38 mmol). The product was purified by flash column chromatography on silica gel (4:1→1:1, pentane/EtOAc). (375 mg, 1.25 mmol, 91% yield). White foam.

M.p. = 63 – 65 °C (pentane/EtOAc). **^{1}H NMR** (400 MHz, $CDCl_3$) δ 7.38 – 7.32 (m, 2H), 7.26 – 7.24 (m, 1H), 7.24 – 7.22 (m, 1H), 7.21 (d, *J* = 4.5 Hz, 4H), 6.98 (h, *J* = 4.1 Hz, 1H), 6.05 (s, 1H), 4.91 (dd, *J* = 7.9, 3.8 Hz, 1H), 3.82 – 3.67 (m, 2H), 2.50 – 2.34 (m, 1H), 2.06 – 1.94 (m, 2H), 1.94 – 1.84 (m, 1H). **^{13}C NMR** (101 MHz, $CDCl_3$) δ 154.2, 141.6, 139.0, 133.6, 129.3, 128.9, 127.3, 123.1, 119.5, 60.8, 47.5, 36.5, 23.4. **HRMS** (ESI/QTOF) *m/z*: $[M + H]^+$ Calcd for $C_{17}H_{18}ClN_2O^+$ 301.1102; Found 301.1099. **IR** (ATR): 3314, 2972, 2949, 2873, 1646, 1594, 1528, 1498, 1441, 1366, 1242, 1089, 751, 692 cm^{-1}.

Effect of Reaction Parameters

Method A (reaction optimization): In a nitrogen-filled glovebox, an oven-dried GC-vial was charged with thiol (10 µmol, 20 mol%), chiral phosphoric acid (10 µmol, 20 mol%), $(Ir[dF(CF_3)ppy]_2(dtbpy))PF_6$ (3.9 mg, 3.5 µmol, 7 mol%), base (0.1 mmol, 2.0 equiv), *N*,2-diphenylpyrrolidine-1-carboxamide (0.05 mmol, 1.0 equiv), and a magnetic stir bar. Then, anhydrous solvent (1.0 mL) was added, and the vial was sealed with a PTFE-lined screw cap. The reaction was taken out of the glovebox and irradiated with a 427 nm Kessil lamp (100% intensity, 5 to 6 cm away) and stirred at ambient temperature with a fan to cool the reaction setup. After 20 h, the reaction was filtered through a pipette containing 2 cm of silica gel and rinsed with 10 mL of EtOAc. The yields were determined by ^{1}H NMR with 1,3-Benzodioxole as an internal standard. The crude mixture was purified by preparative TLC. The enantiomeric ratios were then determined by HPLC analysis on a chiral stationary phase.

Table S1. Evaluation of bases

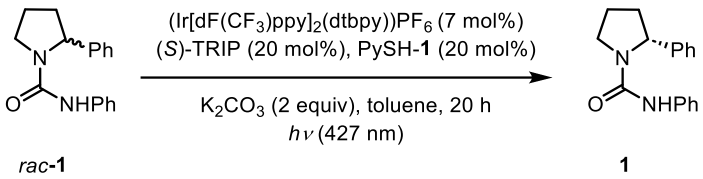

entry	base	yield[a]	er[b]
1	KOAc	80%	70:30
2	K_3PO_4	94%	55:45
3	K_2CO_3	90%	85.5:14.5
4	Na_2CO_3	74%	57:43
5	Cs_2CO_3	96%	52:48

[a]Yields were determined by ^1H NMR using 1,3-benzodioxole as the internal standard. [b]Er values were determined by HPLC analysis using a ChiralPak IB column.

Table S2. Evaluation of solvents

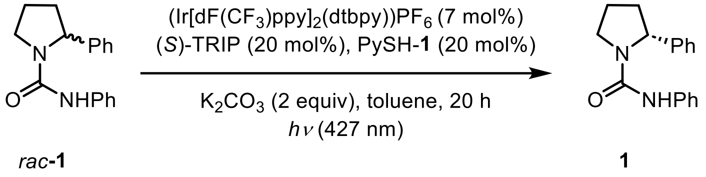

entry	solvent	yield[a]	er[b]
1	toluene	90%	85.5:14.5
2	benzene	86%	79:21
3	CH_2Cl_2	89%	53:47
4	EtOAc	85%	57:43
5	MeCN	95%	52:48
6	α,α,α-trifluorotoluene	67%	66:34
7	*n*-hexane	97%	50:50

[a]Yields were determined by ^{1}H NMR using 1,3-benzodioxole as the internal standard. [b]Er values were determined by HPLC analysis using a ChiralPak IB column.

Photochemical Deracemization of 2-Aryl pyrrolidines

General Procedures for Deracemization

Method B: In a nitrogen-filled glovebox, an oven-dried 4 mL vial was charged with 5-methylpyridine-2-thiol (1.20 mg, 10.0 µmol, 10 mol%), (*S*)- or (*R*)-CPA-**5** (8.7 mg, 10.0 µmol, 10 mol%), (Ir[dF(CF_3)ppy]$_2$(dtbpy))PF_6 (3.4 mg, 3.0 µmol, 3 mol%), K_2CO_3 (27.6 mg, 0.2 mmol, 2.0 equiv), substrate (0.1 mmol, 1.0 equiv), and a magnetic stir bar. Then, anhydrous toluene (2 mL) was added, and the vial was sealed with a PTFE-lined screw cap. The reaction was taken out of the glovebox and irradiated with Penn M2 photoreactor (420 nm) with the following settings: 100% light intensity, 6800 rpm fan cooling, 509 rpm stirring. After 20 h, the solution was concentrated and purified by flash column chromatography with 4:1:0.5 hexane/Et_2O/AcOH and then 1:1 hexane/Et_2O. The enantiomeric ratios were then determined by HPLC analysis on a chiral stationary phase.

Method C (1 mmol scale): In a nitrogen-filled glovebox, an oven-dried 40 mL vial was charged with 5-methylpyridine-2-thiol (12.0 mg, 100 µmol, 10 mol%), (*S*)-CPA-**5** (87.0 mg, 100 µmol, 10 mol%), (Ir[dF(CF_3)ppy]$_2$(dtbpy))PF_6 (34 mg, 30.0 µmol, 3 mol%), K_2CO_3 (276 mg, 2.0 mmol, 2.0 equiv), substrate (1.0 mmol, 1.0 equiv), and a magnetic stir bar. Then, anhydrous toluene (20 mL) was added, and the vial was sealed with a PTFE-lined screw cap. The reaction was taken out of the glovebox and irradiated with Penn M2 photoreactor (420 nm) with the following settings:

100% light intensity, 6800 rpm fan cooling, 1000 rpm stirring. After 42 h, the solution was concentrated, and the product was isolated by flash column chromatography with 4:1:0.5 hexane/Et_2O/AcOH and then 1:1 hexane/Et_2O. The enantiomeric ratios were then determined by HPLC analysis on a chiral stationary phase.

Characterization of Products

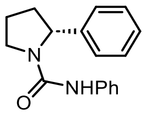

Isolated Yield: 26.0 mg, 98%
Optical Rotation: **$[\alpha]_D^{20}$** = +64.6 (c = 1, $CHCl_3$, 97:3 er).
HPLC: Chiralpak IB; eluent: *n*-hexane/*i*-propanol 95:5; flow rate: 1.0 mL/min.

Racemic sample: *rac*-**1**

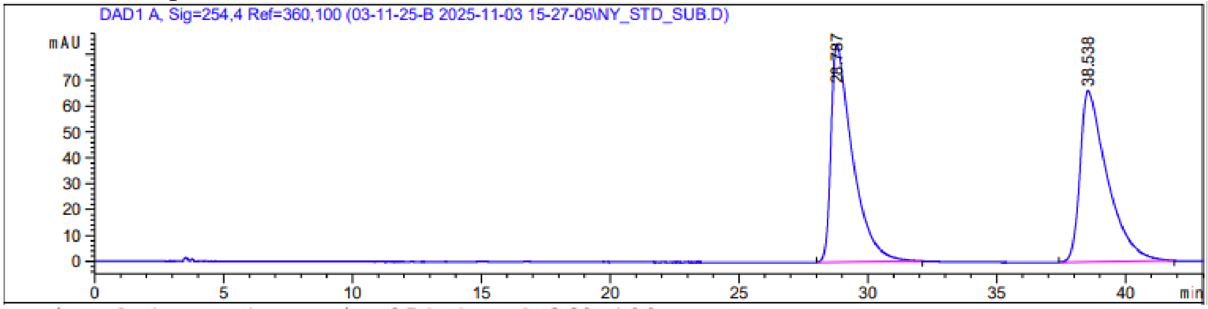

Signal 1: DAD1 A, Sig=254,4 Ref=360,100

Peak #	RetTime [min]	Type	Width [min]	Area [mAU*s]	Height [mAU]	Area %
1	28.787	BB	0.7958	4911.19971	84.28593	50.2101
2	38.538	BB	1.0264	4870.09521	66.19301	49.7899

Enantioenriched sample: **1**

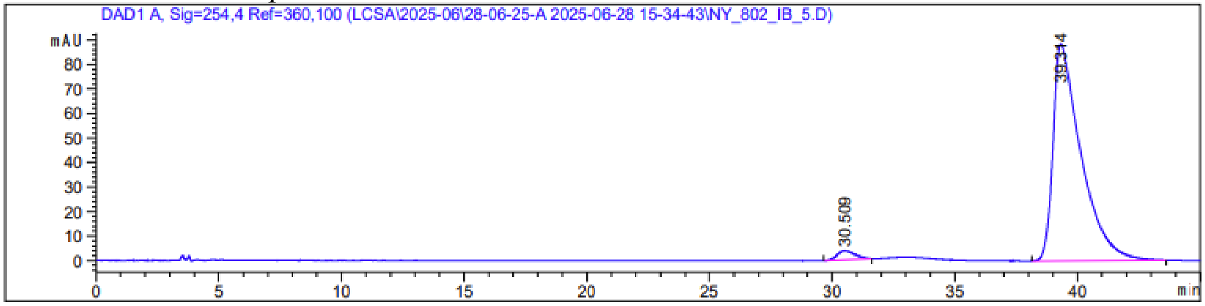

Signal 1: DAD1 A, Sig=254,4 Ref=360,100

Peak #	RetTime [min]	Type	Width [min]	Area [mAU*s]	Height [mAU]	Area %
1	30.509	BB	0.5948	184.69197	3.67227	2.5716
2	39.314	BB	1.0786	6997.27930	88.48084	97.4284

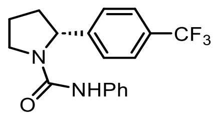

Isolated Yield: 33.0 mg, 99%
Optical Rotation: $[\alpha]_D^{20}$ = +73.3 (c = 1, $CHCl_3$, 95:5 er).
HPLC: Chiralpak IB; eluent: *n*-hexane/*i*-propanol 80:20; flow rate: 1.0 mL/min.

Racemic sample: *rac*-**2**

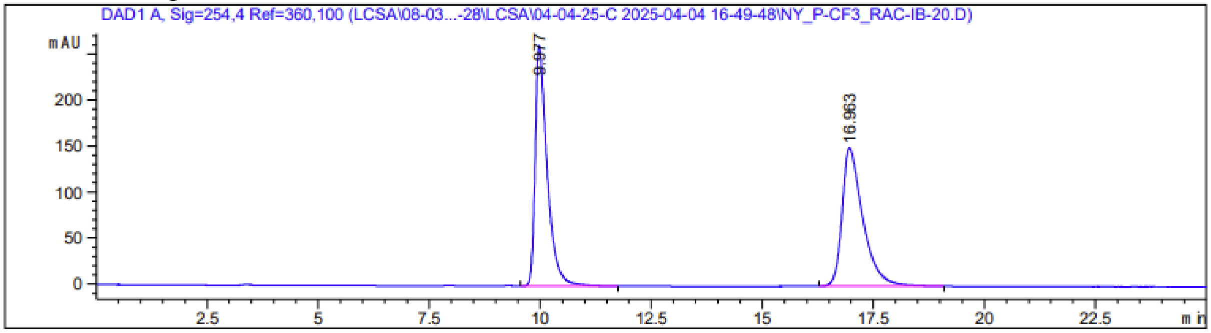

Signal 1: DAD1 A, Sig=254,4 Ref=360,100

Peak #	RetTime [min]	Type	Width [min]	Area [mAU*s]	Height [mAU]	Area %
1	9.977	BB	0.2760	4926.66699	261.22284	50.1310
2	16.963	BB	0.4762	4900.92822	150.37503	49.8690

Enantioenriched sample: **2**

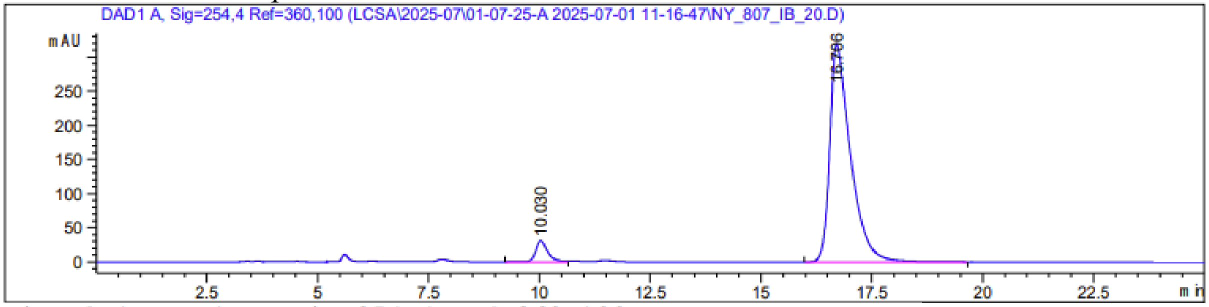

Signal 1: DAD1 A, Sig=254,4 Ref=360,100

Peak #	RetTime [min]	Type	Width [min]	Area [mAU*s]	Height [mAU]	Area %
1	10.030	BV	0.2791	578.27191	31.07106	5.2964
2	16.706	BB	0.4724	1.03400e4	318.77597	94.7036

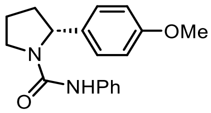

Isolated Yield: 26.8 mg, 90%
Optical Rotation: **$[\alpha]_D^{20}$** = +71.2 (c = 1, $CHCl_3$, 94:6 er).
HPLC: Chiralpak IB; eluent: *n*-hexane/*i*-propanol 90:10; flow rate: 1.0 mL/min.

Racemic sample: *rac*-**3**

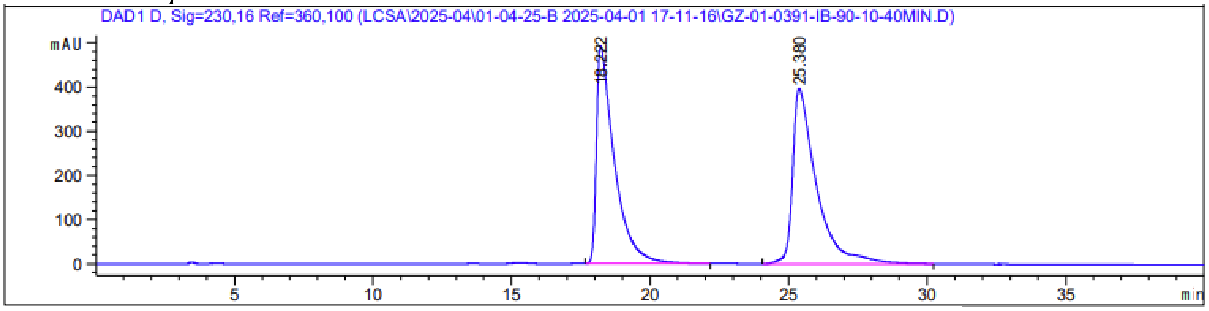

Signal 3: DAD1 D, Sig=230,16 Ref=360,100

Peak #	RetTime [min]	Type	Width [min]	Area [mAU*s]	Height [mAU]	Area %
1	18.222	BB	0.6427	2.22195e4	492.20465	48.4744
2	25.380	BB	0.8508	2.36181e4	396.60181	51.5256

Enantioenriched sample: **3**

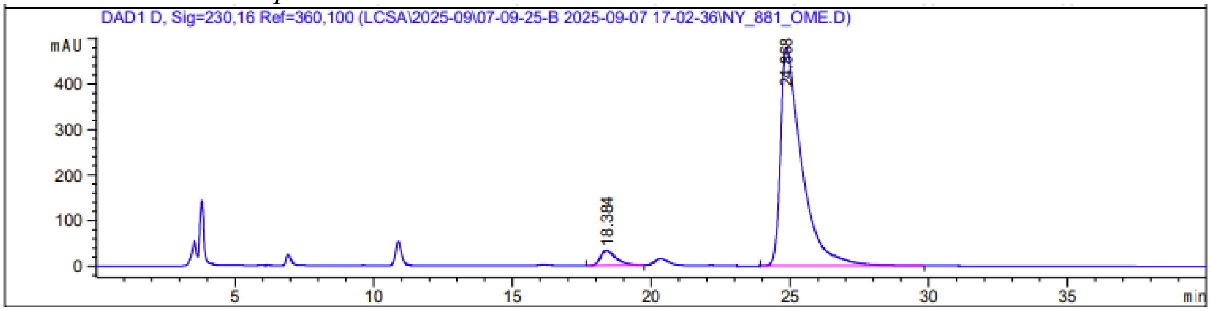

Signal 3: DAD1 D, Sig=230,16 Ref=360,100

Peak #	RetTime [min]	Type	Width [min]	Area [mAU*s]	Height [mAU]	Area %
1	18.384	BB	0.5830	1312.01147	33.35079	4.8891
2	24.868	BB	0.7464	2.55233e4	479.93076	95.1109

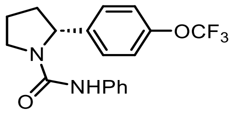

Isolated Yield: 31.2 mg, 89%
Optical Rotation: **[α]$_D^{20}$** = +74.7 (c = 1, $CHCl_3$, 96:4 er).
HPLC: Chiralpak IA; eluent: *n*-hexane/*i*-propanol 80:20; flow rate: 1.0 mL/min.

Racemic sample: *rac*-**4**

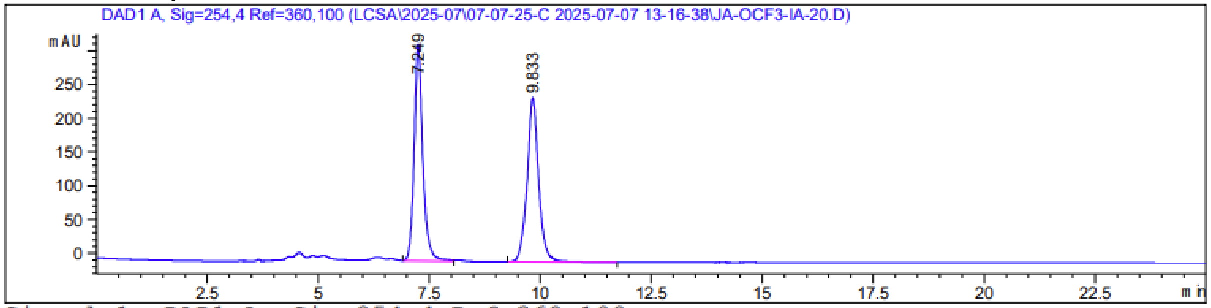

Signal 1: DAD1 A, Sig=254,4 Ref=360,100

Peak #	RetTime [min]	Type	Width [min]	Area [mAU*s]	Height [mAU]	Area %
1	7.249	BV	0.2009	4369.30762	320.88800	49.8672
2	9.833	BB	0.2613	4392.57422	242.57832	50.1328

Enantioenriched sample: **4**

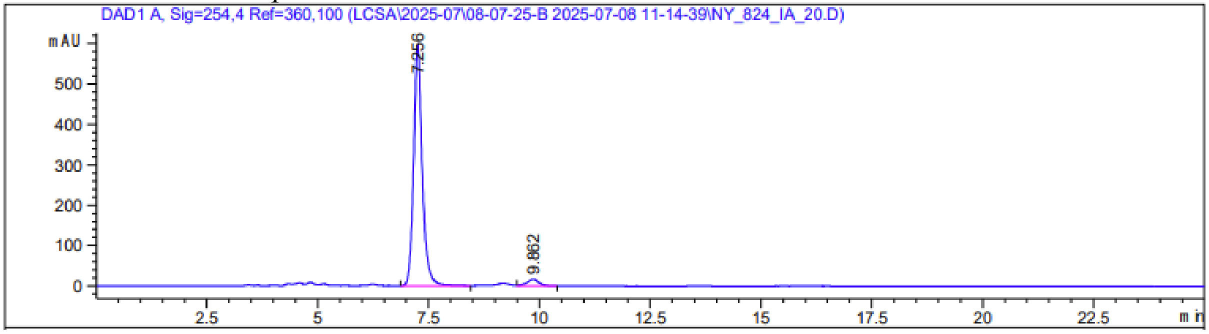

Signal 1: DAD1 A, Sig=254,4 Ref=360,100

Peak #	RetTime [min]	Type	Width [min]	Area [mAU*s]	Height [mAU]	Area %
1	7.256	BV	0.2002	8088.46143	596.34229	96.1297
2	9.862	VV	0.2776	325.65472	17.29754	3.8703

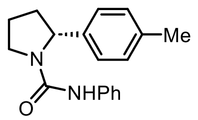

Isolated Yield: 27.8 mg, 99%
Optical Rotation: $[\alpha]_D^{20}$ = +73.2 (c = 1, $CHCl_3$, 96:4 er).
HPLC: Chiralpak IB; eluent: *n*-hexane/*i*-propanol 95:5; flow rate: 1.0 mL/min.

Racemic sample: *rac*-**5**

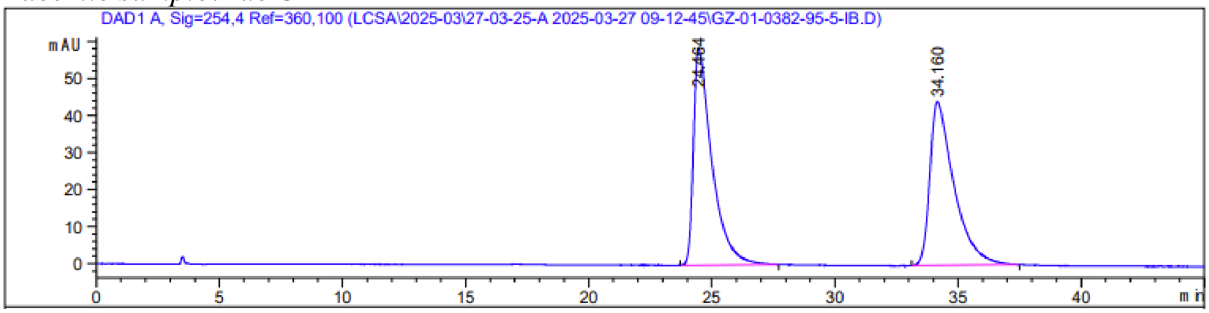

Signal 1: DAD1 A, Sig=254,4 Ref=360,100

Peak #	RetTime [min]	Type	Width [min]	Area [mAU*s]	Height [mAU]	Area %
1	24.464	BB	0.7547	3059.13159	58.63048	50.2211
2	34.160	BB	0.9345	3032.18994	44.19285	49.7789

Enantioenriched sample: **5**

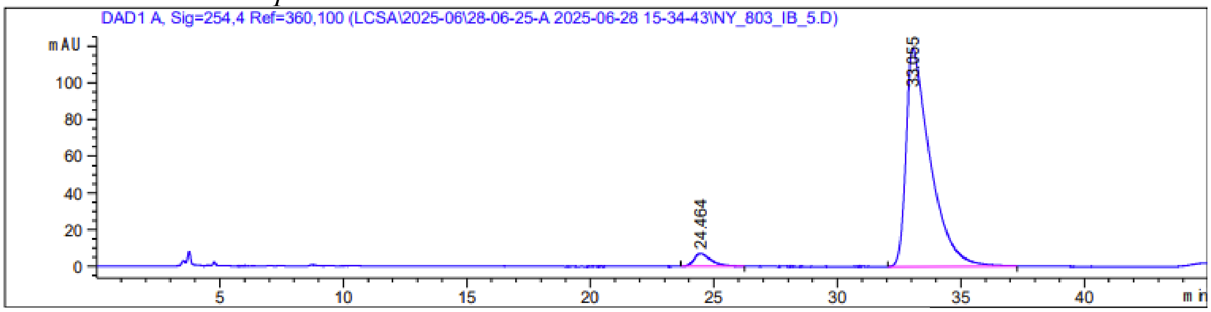

Signal 1: DAD1 A, Sig=254,4 Ref=360,100

Peak #	RetTime [min]	Type	Width [min]	Area [mAU*s]	Height [mAU]	Area %
1	24.464	BB	0.6458	338.80521	7.08620	4.0314
2	33.055	BB	0.9361	8065.32568	119.09155	95.9686

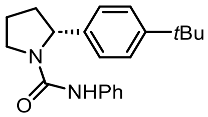

Isolated Yield: 31.5 mg, 98%
Optical Rotation: **[α]$_D$^{20}** = +97 (c = 1, $CHCl_3$, 97:3 er).
HPLC: Chiralpak IB; eluent: *n*-hexane/*i*-propanol 80:20; flow rate: 1.0 mL/min.

Racemic sample: *rac*-**6**

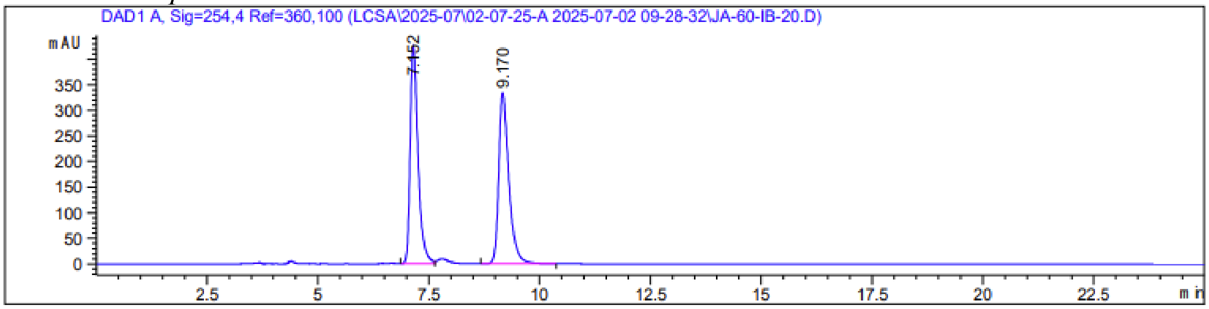

Signal 1: DAD1 A, Sig=254,4 Ref=360,100

Peak #	RetTime [min]	Type	Width [min]	Area [mAU*s]	Height [mAU]	Area %
1	7.152	BV	0.1851	5216.87549	425.41309	49.7043
2	9.170	BB	0.2368	5278.94287	333.21042	50.2957

Enantioenriched sample: **6**

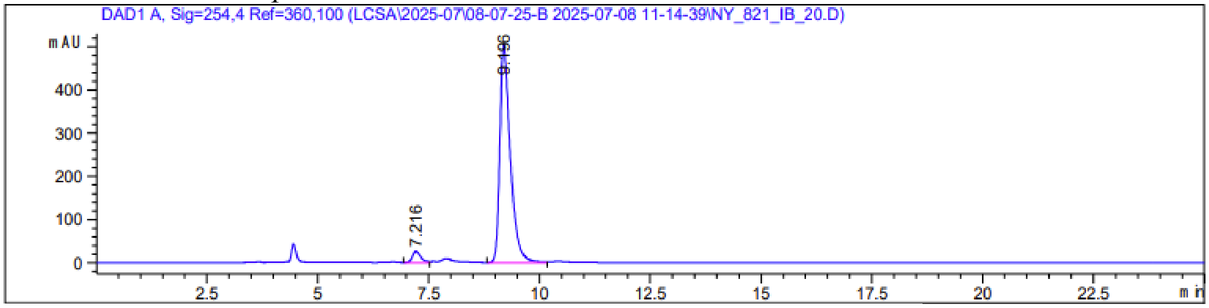

Signal 1: DAD1 A, Sig=254,4 Ref=360,100

Peak #	RetTime [min]	Type	Width [min]	Area [mAU*s]	Height [mAU]	Area %
1	7.216	BB	0.1826	283.75995	23.87849	3.4655
2	9.195	BV	0.2412	7904.37695	482.19400	96.5345

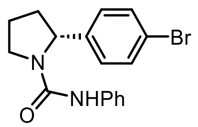

Isolated Yield: 32.4 mg, 94%
Optical Rotation: **$[\alpha]_D^{20}$** = +84.5 (c = 1, $CHCl_3$, 93:7 er).
HPLC: Chiralpak IB; eluent: *n*-hexane/*i*-propanol 80:20; flow rate: 1.0 mL/min.

Racemic sample: *rac*-**7**

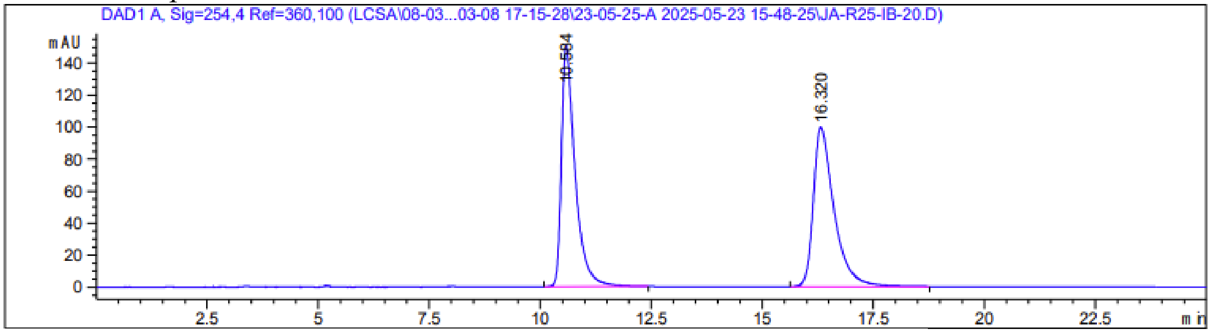

Signal 1: DAD1 A, Sig=254,4 Ref=360,100

Peak #	RetTime [min]	Type	Width [min]	Area [mAU*s]	Height [mAU]	Area %
1	10.584	BB	0.3139	3229.21997	150.76521	50.0089
2	16.320	BB	0.4758	3228.06445	99.67339	49.9911

Enantioenriched sample: **7**

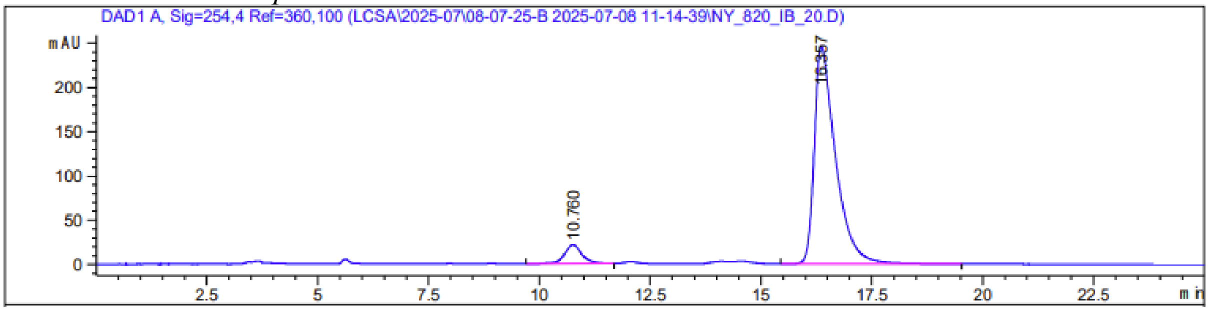

Signal 1: DAD1 A, Sig=254,4 Ref=360,100

Peak #	RetTime [min]	Type	Width [min]	Area [mAU*s]	Height [mAU]	Area %
1	10.760	BB	0.4064	599.00983	21.81241	6.8030
2	16.357	BB	0.4871	8206.12988	246.06927	93.1970

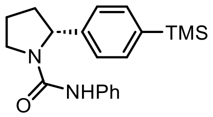

Isolated Yield: 32.8 mg, 97%
Optical Rotation: **[α]**$_D^{20}$ = +101.7 (c = 1, $CHCl_3$, 97:3 er).
HPLC: Chiralpak IB; eluent: *n*-hexane/*i*-propanol 80:20; flow rate: 1.0 mL/min.

Racemic sample: *rac*-**8**

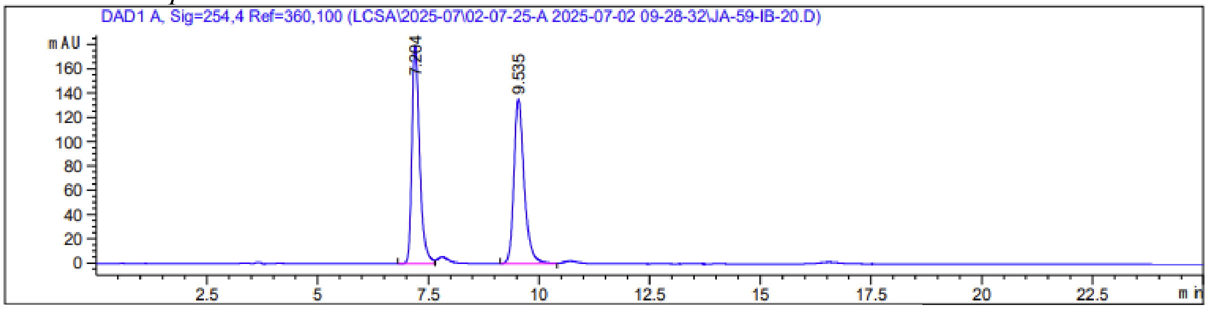

Signal 1: DAD1 A, Sig=254,4 Ref=360,100

Peak #	RetTime [min]	Type	Width [min]	Area [mAU*s]	Height [mAU]	Area %
1	7.204	BV	0.1808	2163.18457	179.22491	49.7970
2	9.535	BV	0.2420	2180.81738	135.36578	50.2030

Enantioenriched sample: **8**

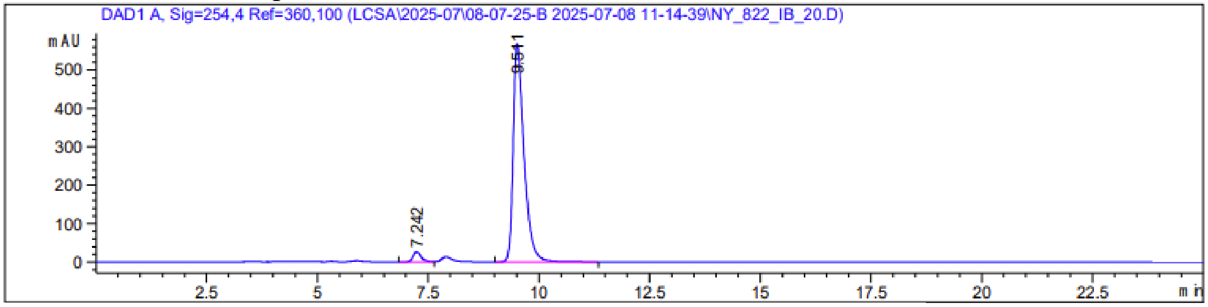

Signal 1: DAD1 A, Sig=254,4 Ref=360,100

Peak #	RetTime [min]	Type	Width [min]	Area [mAU*s]	Height [mAU]	Area %
1	7.242	BV	0.1940	339.42255	26.39078	3.4181
2	9.511	BB	0.2492	9590.82715	567.51141	96.5819

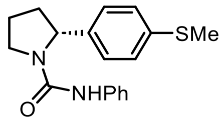

Isolated Yield: 30.6 mg, 98%
Optical Rotation: $[\alpha]_D^{20}$ = +102 (c = 1, $CHCl_3$, 94:6 er).
HPLC: Chiralpak IB; eluent: *n*-hexane/*i*-propanol 80:20; flow rate: 1.0 mL/min.

Racemic sample: *rac*-**9**

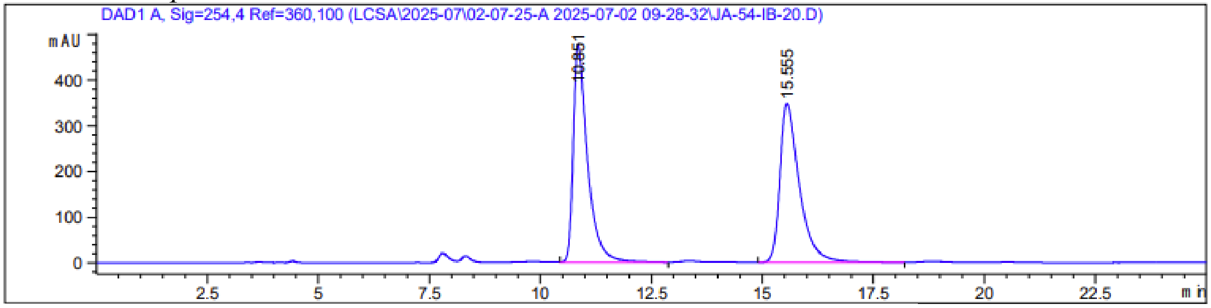

Signal 1: DAD1 A, Sig=254,4 Ref=360,100

Peak #	RetTime [min]	Type	Width [min]	Area [mAU*s]	Height [mAU]	Area %
1	10.851	VB	0.3262	1.06513e4	477.55051	49.9553
2	15.555	BV	0.4536	1.06704e4	348.10419	50.0447

Enantioenriched sample: **9**

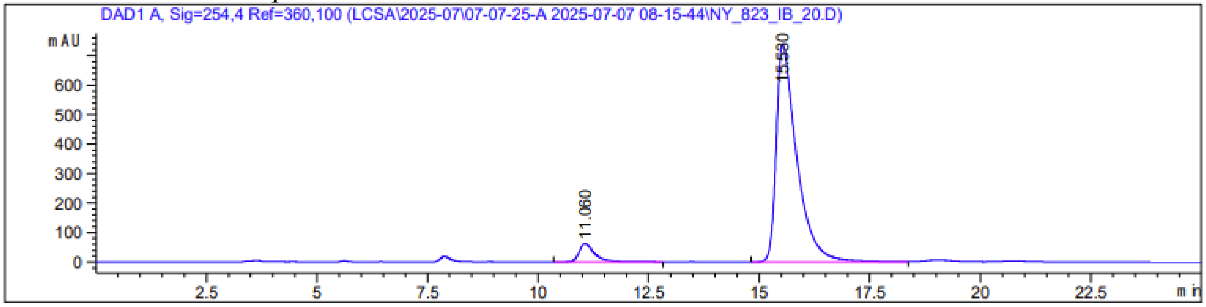

Signal 1: DAD1 A, Sig=254,4 Ref=360,100

Peak #	RetTime [min]	Type	Width [min]	Area [mAU*s]	Height [mAU]	Area %
1	11.060	BB	0.3608	1514.75623	61.60798	5.9730
2	15.530	BV	0.4680	2.38453e4	739.82117	94.0270

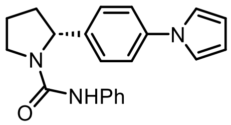

Isolated Yield: 32.9 mg, 99%
Optical Rotation: **$[\alpha]_D^{20}$** = +105 (c = 1, $CHCl_3$, 95:5 er).
HPLC: Chiralpak IB; eluent: *n*-hexane/*i*-propanol 80:20; flow rate: 1.0 mL/min.

Racemic sample: *rac*-**10**

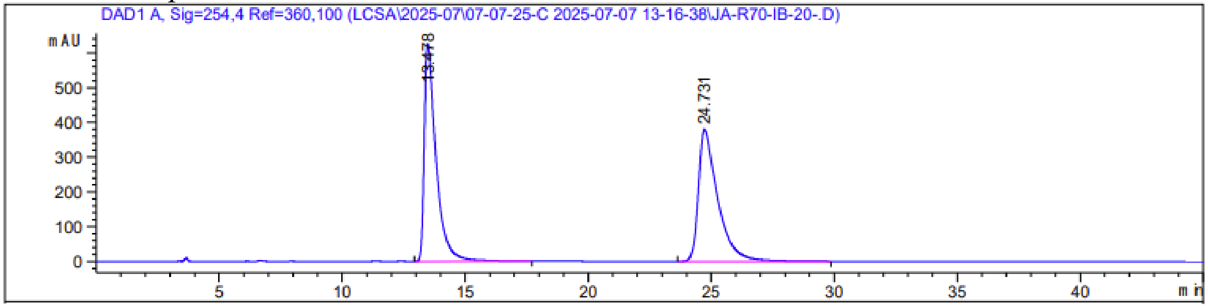

Signal 1: DAD1 A, Sig=254,4 Ref=360,100

Peak #	RetTime [min]	Type	Width [min]	Area [mAU*s]	Height [mAU]	Area %
1	13.478	BB	0.4844	2.07192e4	625.54242	49.9628
2	24.731	BB	0.7736	2.07501e4	379.65216	50.0372

Enantioenriched sample: **10**

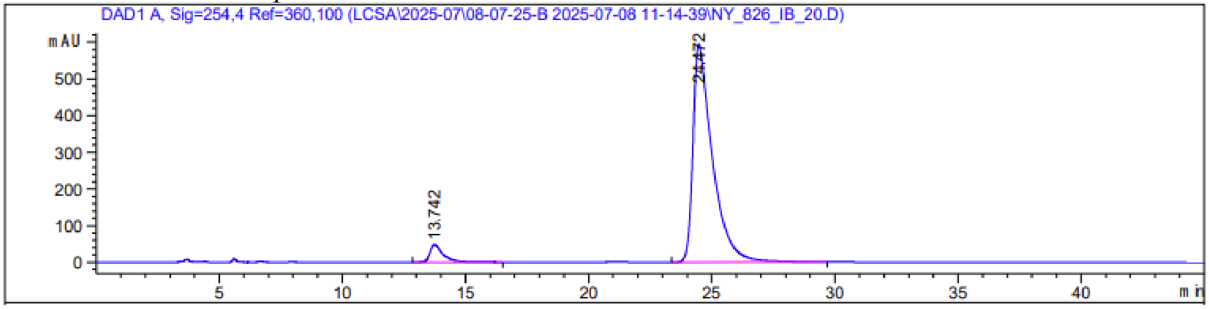

Signal 1: DAD1 A, Sig=254,4 Ref=360,100

Peak #	RetTime [min]	Type	Width [min]	Area [mAU*s]	Height [mAU]	Area %
1	13.742	BB	0.5431	1794.09766	47.66552	5.2272
2	24.472	BB	0.7686	3.25279e4	594.26001	94.7728

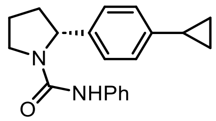

Isolated Yield: 29.3 mg, 96%
Optical Rotation: $[\alpha]_D^{20}$ = +91 (c = 1, $CHCl_3$, 96:4 er).
HPLC: Chiralpak IB; eluent: *n*-hexane/*i*-propanol 80:20; flow rate: 1.0 mL/min.

Racemic sample: *rac*-**11**

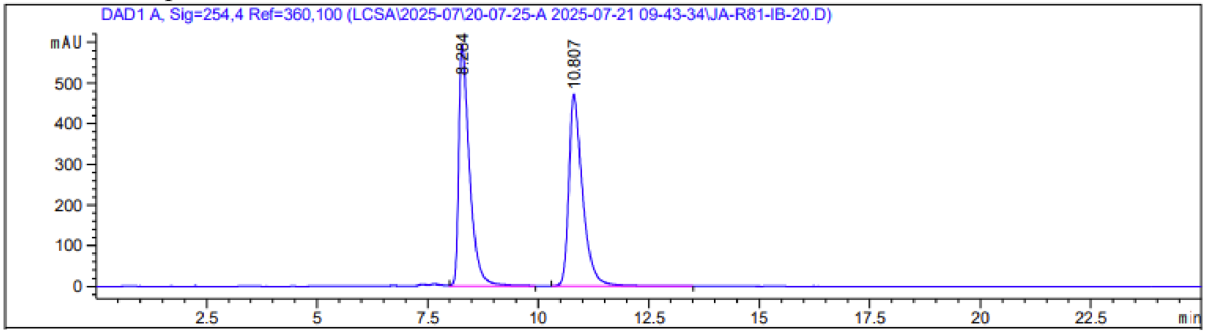

Signal 1: DAD1 A, Sig=254,4 Ref=360,100

Peak #	RetTime [min]	Type	Width [min]	Area [mAU*s]	Height [mAU]	Area %
1	8.284	VV	0.2484	1.00685e4	592.05994	49.9309
2	10.807	BB	0.3157	1.00963e4	471.74744	50.0691

Enantioenriched sample: **11**

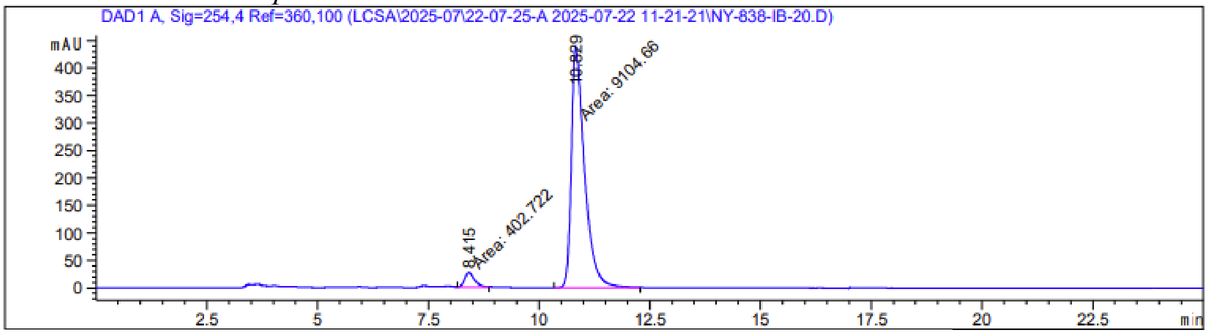

Signal 1: DAD1 A, Sig=254,4 Ref=360,100

Peak #	RetTime [min]	Type	Width [min]	Area [mAU*s]	Height [mAU]	Area %
1	8.415	MM	0.2545	402.72202	26.37855	4.2359
2	10.829	MF	0.3467	9104.66211	437.63095	95.7641

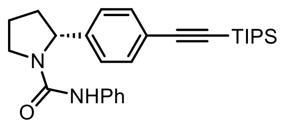

Isolated Yield: 40.0 mg, 90%
Optical Rotation: **$[\alpha]_D^{20}$** = +118 (c = 1, $CHCl_3$, 95:5 er).
HPLC: Chiralpak IB; eluent: *n*-hexane/*i*-propanol 80:20; flow rate: 1.0 mL/min.

Racemic sample: *rac*-**12**

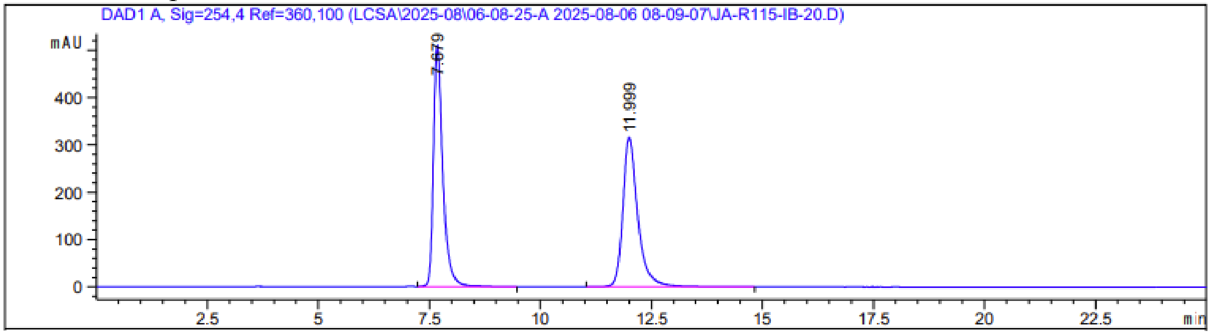

Signal 1: DAD1 A, Sig=254,4 Ref=360,100

Peak #	RetTime [min]	Type	Width [min]	Area [mAU*s]	Height [mAU]	Area %
1	7.679	VV	0.2202	7525.11133	509.93906	50.2227
2	11.999	BB	0.3500	7458.36182	315.23325	49.7773

Enantioenriched sample: **12**

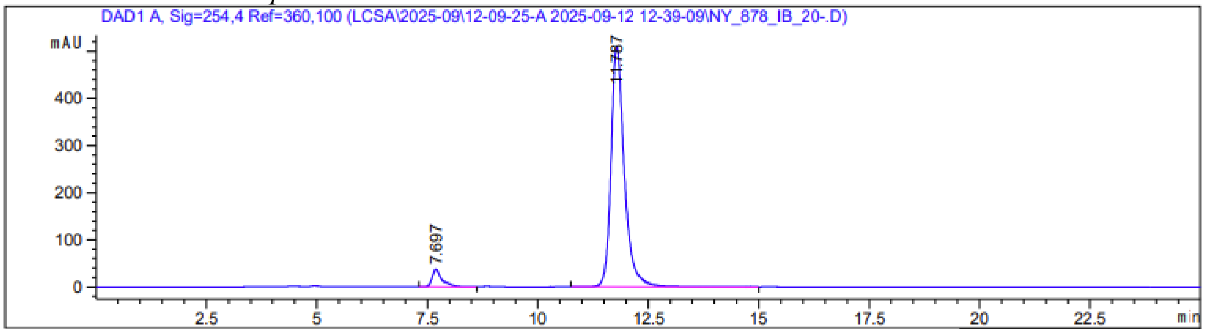

Signal 1: DAD1 A, Sig=254,4 Ref=360,100

Peak #	RetTime [min]	Type	Width [min]	Area [mAU*s]	Height [mAU]	Area %
1	7.697	BV	0.2313	590.23523	36.75897	5.2181
2	11.787	BB	0.3157	1.07210e4	509.04260	94.7819

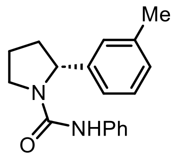

Isolated Yield: 25.6 mg, 91%
Optical Rotation: **$[\alpha]_D^{20}$** = +84 (c = 1, $CHCl_3$, 96:4 er).
HPLC: Chiralpak ID; eluent: *n*-hexane/*i*-propanol 80:20; flow rate: 1.0 mL/min.

Racemic sample: *rac*-**13**

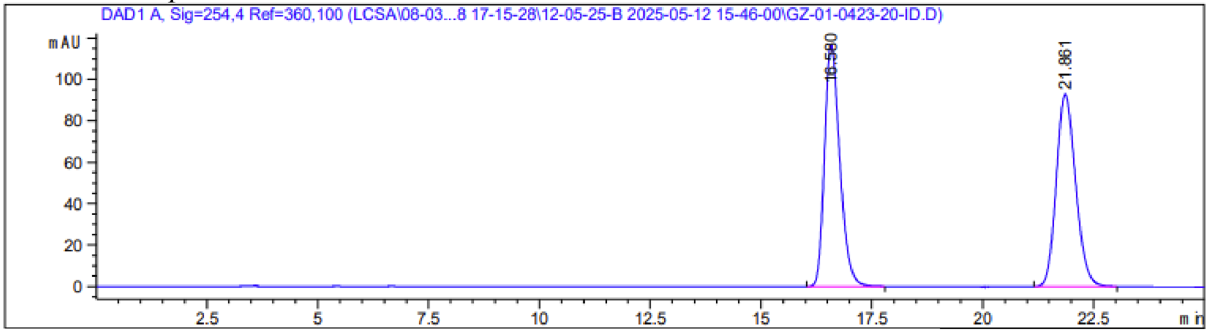

Signal 1: DAD1 A, Sig=254,4 Ref=360,100

Peak #	RetTime [min]	Type	Width [min]	Area [mAU*s]	Height [mAU]	Area %
1	16.580	BB	0.3719	2814.09448	116.47056	49.9003
2	21.861	BB	0.4664	2825.34351	93.01881	50.0997

Enantioenriched sample: **13**

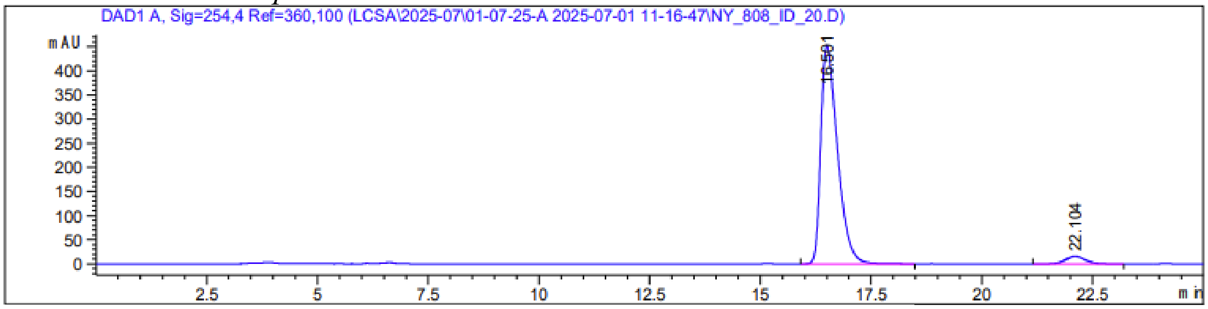

Signal 1: DAD1 A, Sig=254,4 Ref=360,100

Peak #	RetTime [min]	Type	Width [min]	Area [mAU*s]	Height [mAU]	Area %
1	16.501	BB	0.3978	1.17812e4	452.40555	96.0553
2	22.104	BB	0.4729	483.82245	15.46984	3.9447

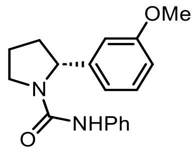

Isolated Yield: 28.3 mg, 95%
Optical Rotation: **[α]$_D$^{20}** = +64 (c = 1, $CHCl_3$, 96:4 er).
HPLC: Chiralpak IA; eluent: *n*-hexane/*i*-propanol 80:20; flow rate: 1.0 mL/min.

Racemic sample: *rac*-**14**

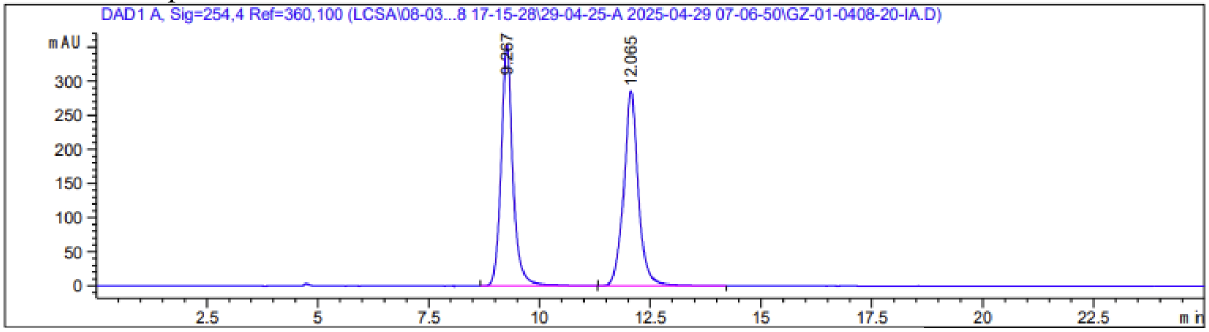

Signal 1: DAD1 A, Sig=254,4 Ref=360,100

Peak #	RetTime [min]	Type	Width [min]	Area [mAU*s]	Height [mAU]	Area %
1	9.267	BB	0.2733	6508.96533	352.77225	49.9226
2	12.065	BB	0.3308	6529.15869	285.46109	50.0774

Enantioenriched sample: **14**

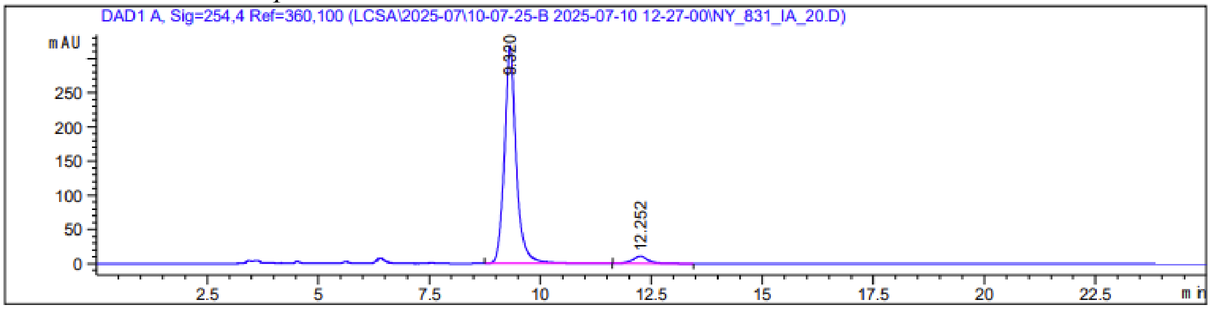

Signal 1: DAD1 A, Sig=254,4 Ref=360,100

Peak #	RetTime [min]	Type	Width [min]	Area [mAU*s]	Height [mAU]	Area %
1	9.320	BB	0.2717	5900.70947	319.09161	95.7235
2	12.252	BB	0.3480	263.61862	10.90648	4.2765

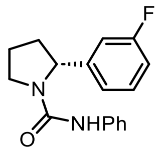

Isolated Yield: 28.1 mg, 99%
Optical Rotation: $[\alpha]_D^{20}$ = +72.8 (c = 1, $CHCl_3$, 95:5 er).
HPLC: Chiralpak IB; eluent: *n*-hexane/*i*-propanol 80:20; flow rate: 1.0 mL/min.

Racemic sample: *rac*-**15**

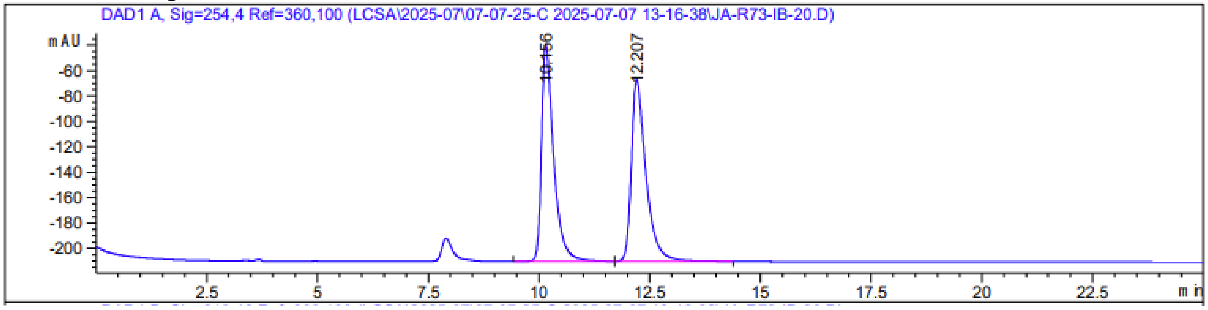

Signal 1: DAD1 A, Sig=254,4 Ref=360,100

Peak #	RetTime [min]	Type	Width [min]	Area [mAU*s]	Height [mAU]	Area %
1	10.156	BB	0.2827	3268.45728	171.19786	49.9006
2	12.207	BB	0.3369	3281.48413	143.42229	50.0994

Enantioenriched sample: **15**

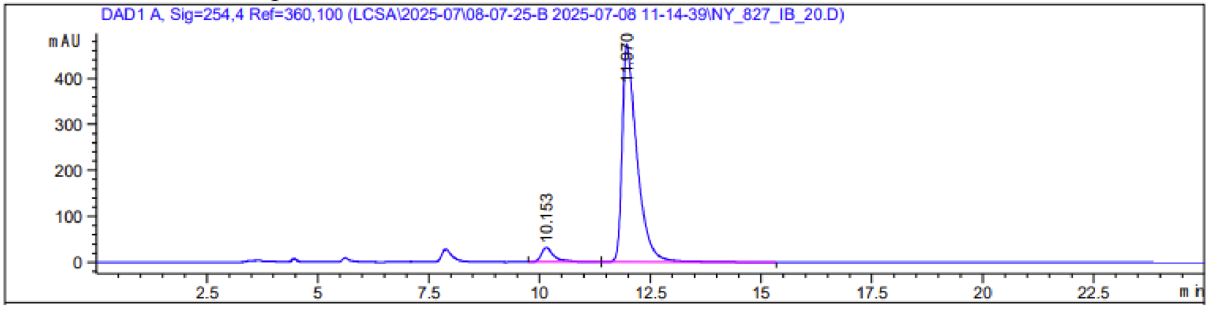

Signal 1: DAD1 A, Sig=254,4 Ref=360,100

Peak #	RetTime [min]	Type	Width [min]	Area [mAU*s]	Height [mAU]	Area %
1	10.153	BB	0.2947	627.64172	31.19942	5.4184
2	11.970	BB	0.3338	1.09559e4	473.80396	94.5816

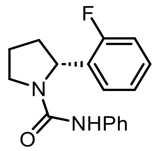

Isolated Yield: 28.2 mg, 99%
Optical Rotation: $[\alpha]_D^{20}$ = +82.3 (c = 1, $CHCl_3$, 94:6 er).
HPLC: Chiralpak IB; eluent: *n*-hexane/*i*-propanol 80:20; flow rate: 1.0 mL/min.

Racemic sample: *rac*-**16**

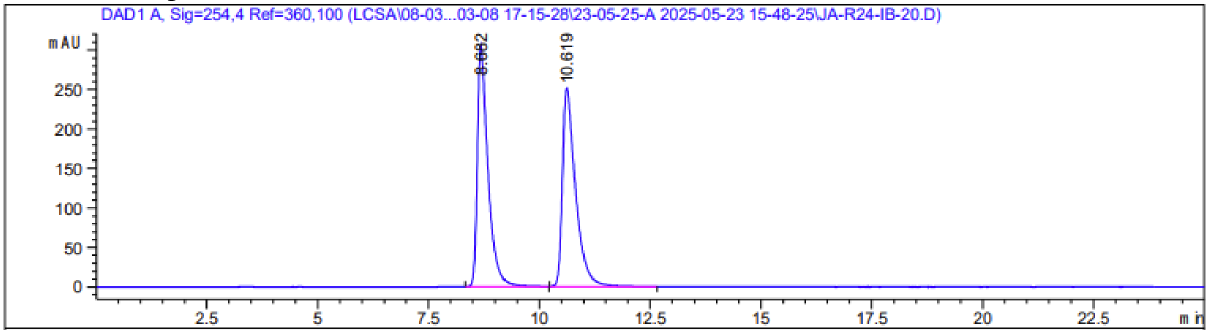

Signal 1: DAD1 A, Sig=254,4 Ref=360,100

Peak #	RetTime [min]	Type	Width [min]	Area [mAU*s]	Height [mAU]	Area %
1	8.682	BB	0.2447	5098.58643	305.55768	49.6385
2	10.619	BB	0.2998	5172.85791	251.64587	50.3615

Enantioenriched sample: **16**

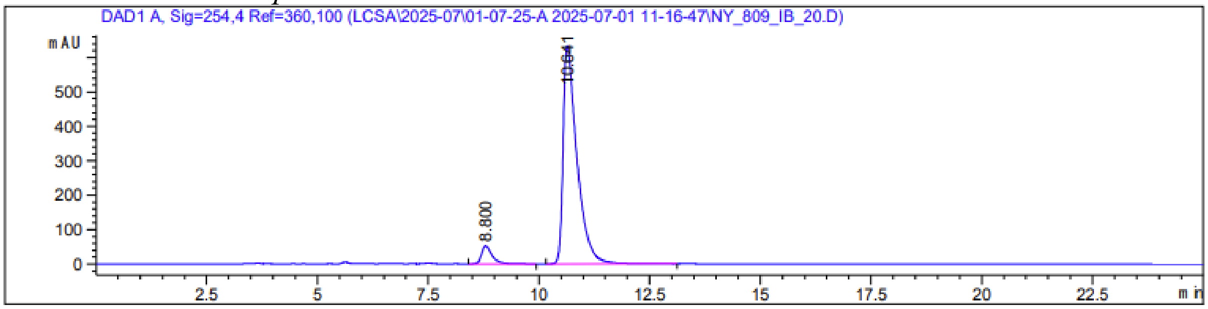

Signal 1: DAD1 A, Sig=254,4 Ref=360,100

Peak #	RetTime [min]	Type	Width [min]	Area [mAU*s]	Height [mAU]	Area %
1	8.800	BB	0.2483	862.97321	51.81617	5.9164
2	10.641	BB	0.3149	1.37231e4	632.97687	94.0836

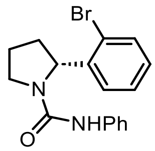

Isolated Yield: 30.1 mg, 87%
Optical Rotation: $[\alpha]_D^{20}$ = +7.5 (c = 1, $CHCl_3$, 90:10 er).
HPLC: Chiralpak IB; eluent: *n*-hexane/*i*-propanol 80:20; flow rate: 1.0 mL/min.

Racemic sample: *rac*-**17**

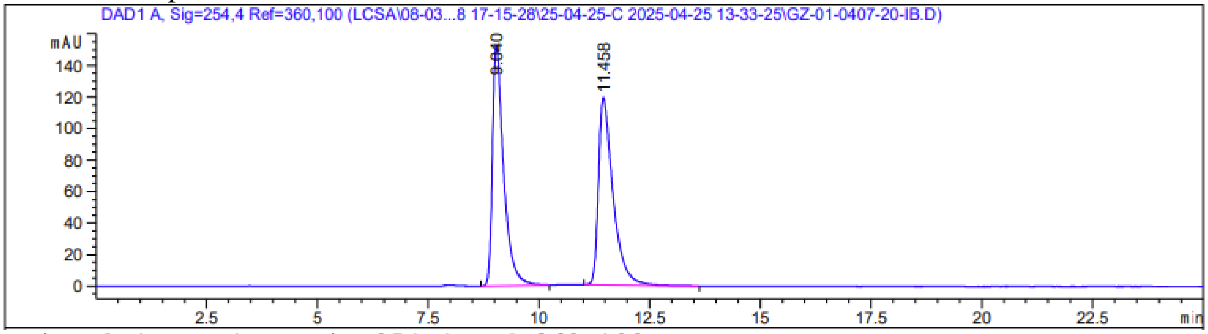

Signal 1: DAD1 A, Sig=254,4 Ref=360,100

Peak #	RetTime [min]	Type	Width [min]	Area [mAU*s]	Height [mAU]	Area %
1	9.040	BB	0.2573	2684.25098	152.54355	49.6982
2	11.458	BB	0.3345	2716.84814	118.94811	50.3018

Enantioenriched sample: **17**

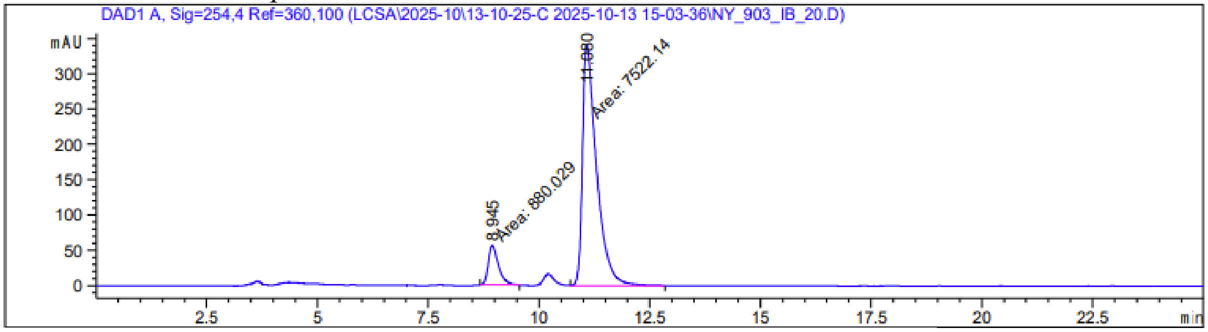

Signal 1: DAD1 A, Sig=254,4 Ref=360,100

Peak #	RetTime [min]	Type	Width [min]	Area [mAU*s]	Height [mAU]	Area %
1	8.945	MM	0.2629	880.02887	55.79972	10.4738
2	11.080	MF	0.3689	7522.13672	339.80576	89.5262

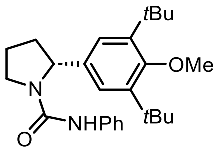

Isolated Yield: 35.4 mg, 87%
Optical Rotation: $[\alpha]_D^{20}$ = +62.7 (c = 1, $CHCl_3$, 95:5 er).
HPLC: Chiralpak ID; eluent: *n*-hexane/*i*-propanol 80:20; flow rate: 1.0 mL/min.

Racemic sample: *rac*-**18**

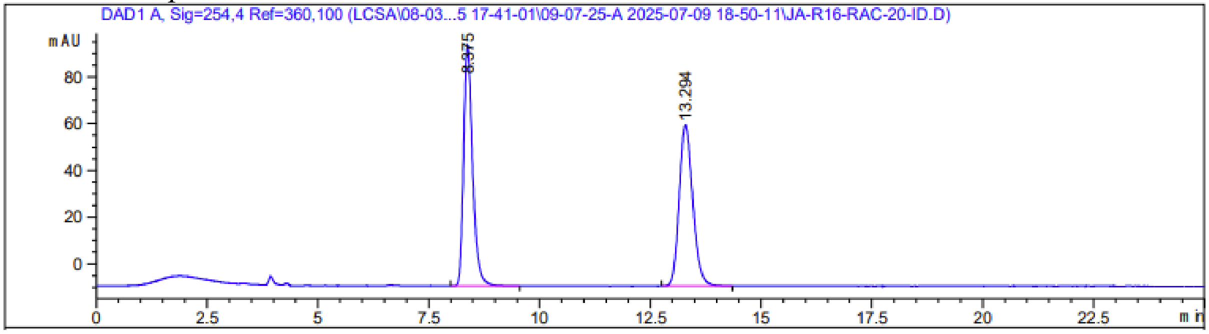

Signal 1: DAD1 A, Sig=254,4 Ref=360,100

Peak #	RetTime [min]	Type	Width [min]	Area [mAU*s]	Height [mAU]	Area %
1	8.375	BB	0.2155	1461.79163	103.07738	49.9557
2	13.294	BB	0.3259	1464.38232	68.92119	50.0443

Enantioenriched sample: **18**

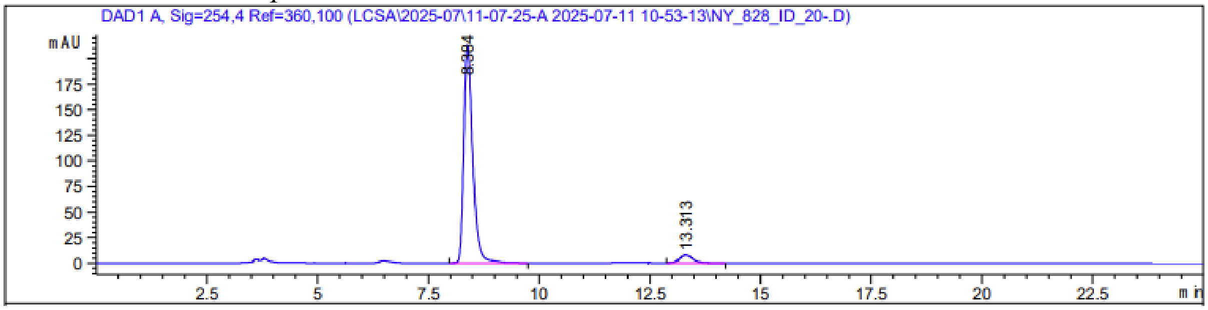

Signal 1: DAD1 A, Sig=254,4 Ref=360,100

Peak #	RetTime [min]	Type	Width [min]	Area [mAU*s]	Height [mAU]	Area %
1	8.384	BB	0.2122	2993.25146	212.66754	94.6577
2	13.313	BB	0.3255	168.93178	8.09439	5.3423

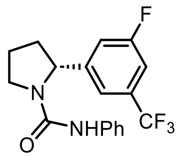

Isolated Yield: 35.0 mg, 99%
Optical Rotation: $[\alpha]_D^{20}$ = +51.33 (c = 1, $CHCl_3$, 89:11 er).
HPLC: Chiralpak IA; eluent: *n*-hexane/*i*-propanol 80:20; flow rate: 1.0 mL/min.

Racemic sample: *rac*-**19**

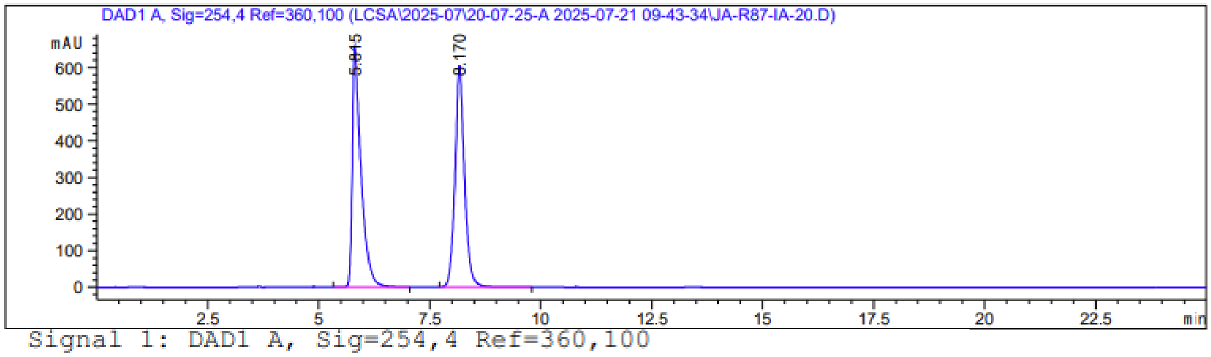

Signal 1: DAD1 A, Sig=254,4 Ref=360,100

Peak #	RetTime [min]	Type	Width [min]	Area [mAU*s]	Height [mAU]	Area %
1	5.815	BB	0.1916	9010.99023	658.59558	50.0715
2	8.170	BB	0.2171	8985.26563	605.62445	49.9285

Enantioenriched sample: **19**

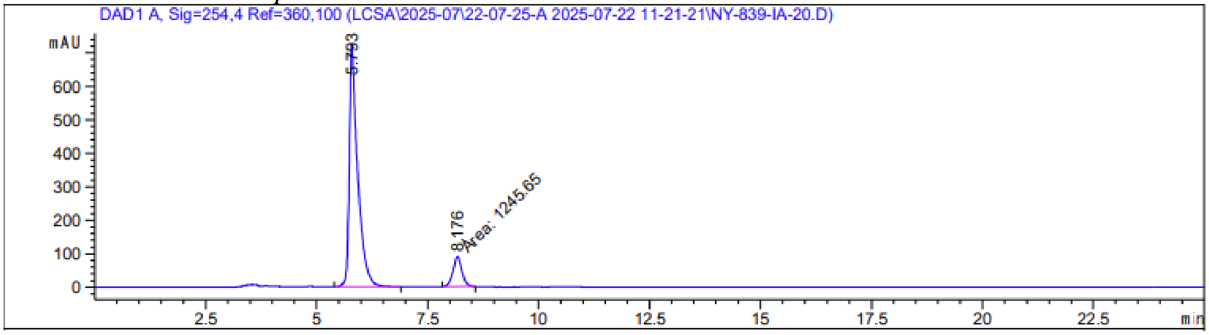

Signal 1: DAD1 A, Sig=254,4 Ref=360,100

Peak #	RetTime [min]	Type	Width [min]	Area [mAU*s]	Height [mAU]	Area %
1	5.793	BV	0.1890	9697.21582	720.75403	88.6168
2	8.176	MM	0.2318	1245.64575	89.57121	11.3832

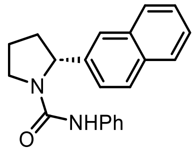

Isolated Yield: 30.6 mg, 97%
Optical Rotation: $[\alpha]_D^{20}$ = +108.7 (c = 1, $CHCl_3$, 96:4 er).
HPLC: Chiralpak IB; eluent: *n*-hexane/*i*-propanol 90:10; flow rate: 1.0 mL/min.

Racemic sample: *rac*-**20**

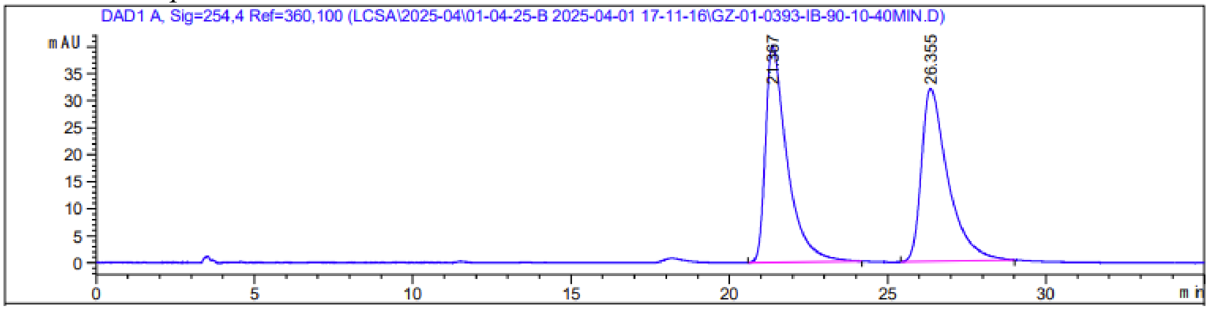

Signal 1: DAD1 A, Sig=254,4 Ref=360,100

Peak #	RetTime [min]	Type	Width [min]	Area [mAU*s]	Height [mAU]	Area %
1	21.367	BB	0.6810	1918.01526	40.17622	50.6479
2	26.355	BB	0.8269	1868.94507	32.02107	49.3521

Enantioenriched sample: **20**

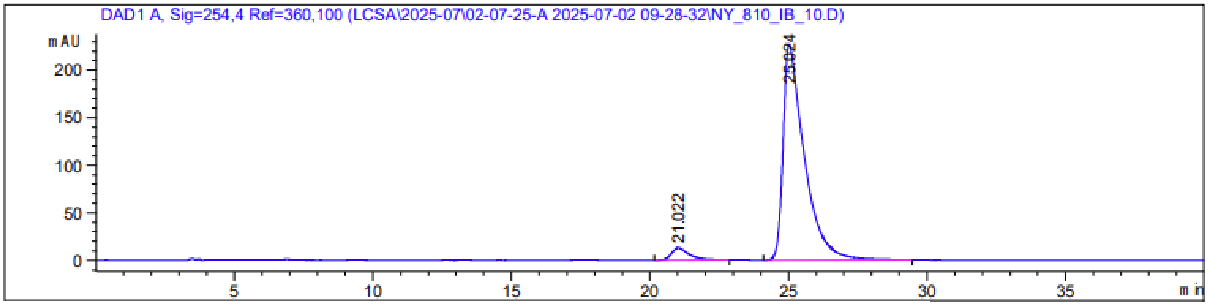

Signal 1: DAD1 A, Sig=254,4 Ref=360,100

Peak #	RetTime [min]	Type	Width [min]	Area [mAU*s]	Height [mAU]	Area %
1	21.022	BB	0.6224	571.08118	13.00388	4.5143
2	25.024	BB	0.7683	1.20794e4	227.19142	95.4857

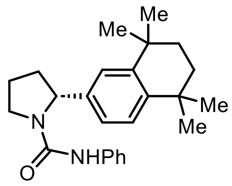

Isolated Yield: 31.3 mg, 83%
Optical Rotation: **[α]D**[20] = +84.2 (c = 1, $CHCl_3$, 94:6 er).
HPLC: Chiralpak IF; eluent: *n*-hexane/*i*-propanol 80:20; flow rate: 1.0 mL/min.

Racemic sample: *rac*-**21**

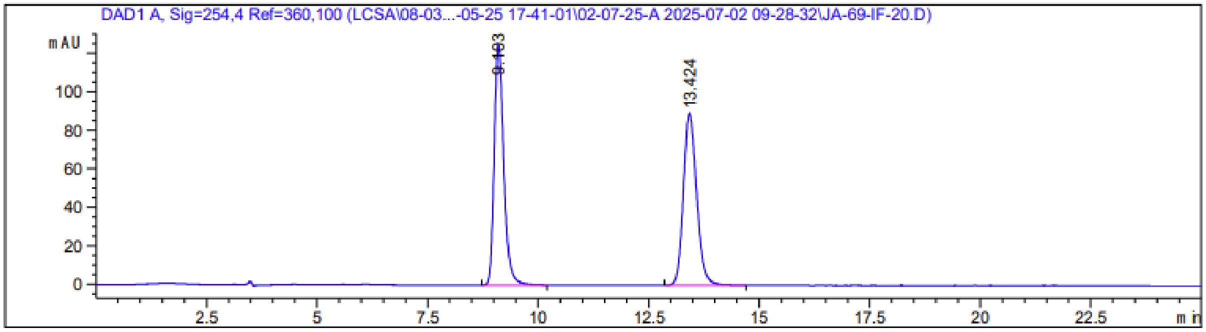

Signal 1: DAD1 A, Sig=254,4 Ref=360,100

Peak #	RetTime [min]	Type	Width [min]	Area [mAU*s]	Height [mAU]	Area %
1	9.103	BB	0.2278	1857.36194	124.68591	49.9598
2	13.424	BB	0.3189	1860.35107	89.34611	50.0402

Enantioenriched sample: **21**

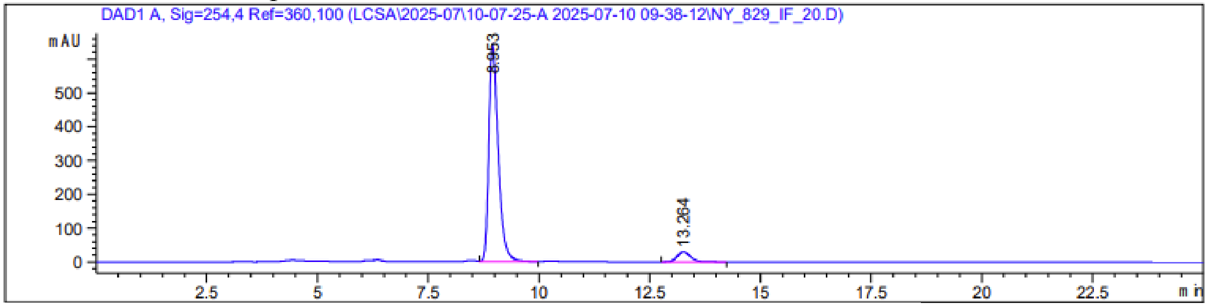

Signal 1: DAD1 A, Sig=254,4 Ref=360,100

Peak #	RetTime [min]	Type	Width [min]	Area [mAU*s]	Height [mAU]	Area %
1	8.953	VV	0.2253	9566.59375	644.14984	93.9716
2	13.264	BB	0.3155	613.70447	29.90026	6.0284

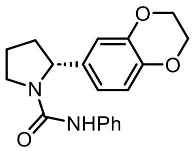

Isolated Yield: 32.0 mg, 99%
Optical Rotation: **$[\alpha]_D^{20}$** = +83 (c = 1, $CHCl_3$, 95:5 er).
HPLC: Chiralpak IA; eluent: *n*-hexane/*i*-propanol 80:20; flow rate: 1.0 mL/min.

Racemic sample: *rac*-**22**

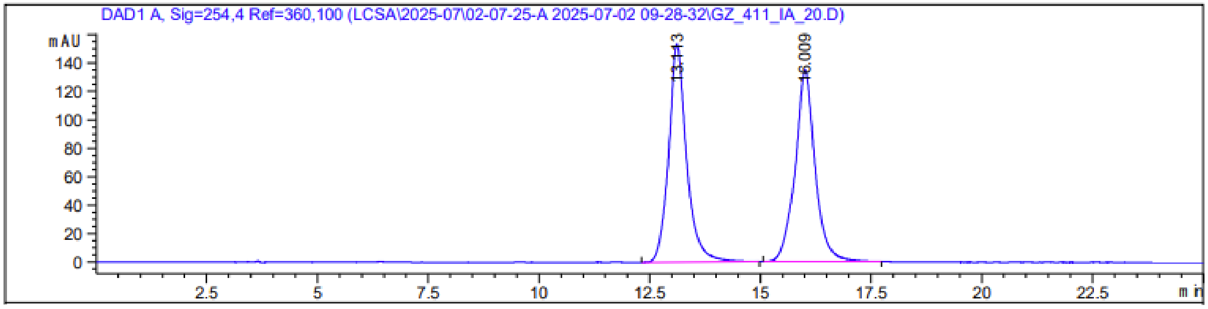

Signal 1: DAD1 A, Sig=254,4 Ref=360,100

Peak #	RetTime [min]	Type	Width [min]	Area [mAU*s]	Height [mAU]	Area %
1	13.113	BB	0.4047	4318.86621	153.28220	50.4507
2	16.009	BB	0.4505	4241.70898	135.01952	49.5493

Enantioenriched sample: **22**

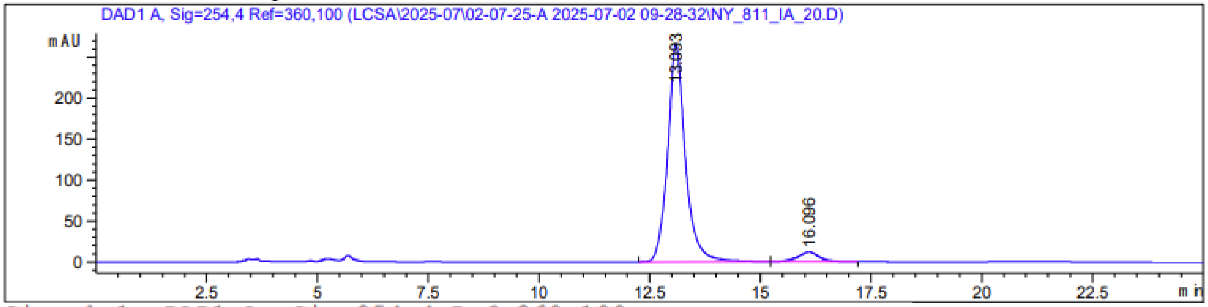

Signal 1: DAD1 A, Sig=254,4 Ref=360,100

Peak #	RetTime [min]	Type	Width [min]	Area [mAU*s]	Height [mAU]	Area %
1	13.093	BB	0.4004	7342.63867	265.68719	95.1197
2	16.096	BB	0.4681	376.72482	11.81303	4.8803

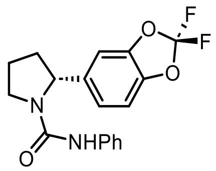

Isolated Yield: 34.0 mg, 98%
Optical Rotation: $[\alpha]_D^{20}$ = +72.2 (c = 1, $CHCl_3$, 95:5 er).
HPLC: Chiralpak IB; eluent: *n*-hexane/*i*-propanol 80:20; flow rate: 1.0 mL/min.

Racemic sample: *rac*-**23**

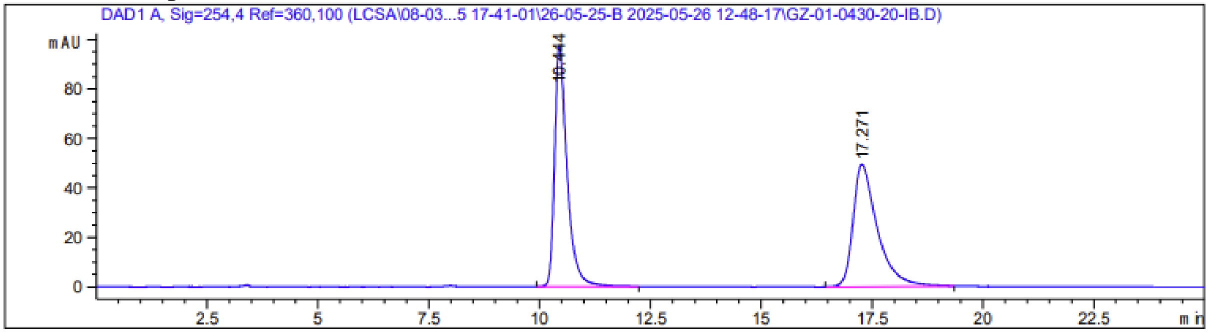

Signal 1: DAD1 A, Sig=254,4 Ref=360,100

Peak #	RetTime [min]	Type	Width [min]	Area [mAU*s]	Height [mAU]	Area %
1	10.444	BB	0.2897	1903.48279	97.52848	50.3932
2	17.271	BB	0.5512	1873.77942	49.32771	49.6068

Enantioenriched sample: **23**

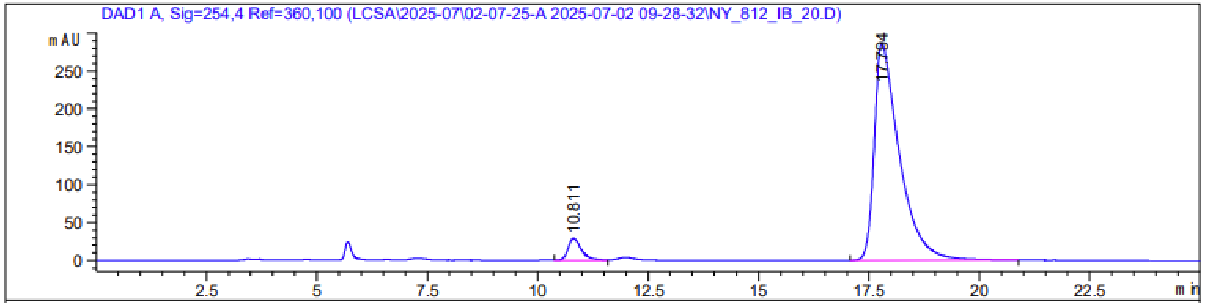

Signal 1: DAD1 A, Sig=254,4 Ref=360,100

Peak #	RetTime [min]	Type	Width [min]	Area [mAU*s]	Height [mAU]	Area %
1	10.811	VV	0.3158	605.77081	28.74822	5.0676
2	17.794	BB	0.5779	1.13480e4	286.62839	94.9324

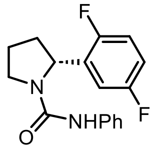

Isolated Yield: 30.0 mg, 99%
Optical Rotation: $[\alpha]_D^{20}$ = +83.2 (c = 1, $CHCl_3$, 97:3 er).
HPLC: Chiralpak IB; eluent: *n*-hexane/*i*-propanol 80:20; flow rate: 1.0 mL/min.

Racemic sample: *rac*-**24**

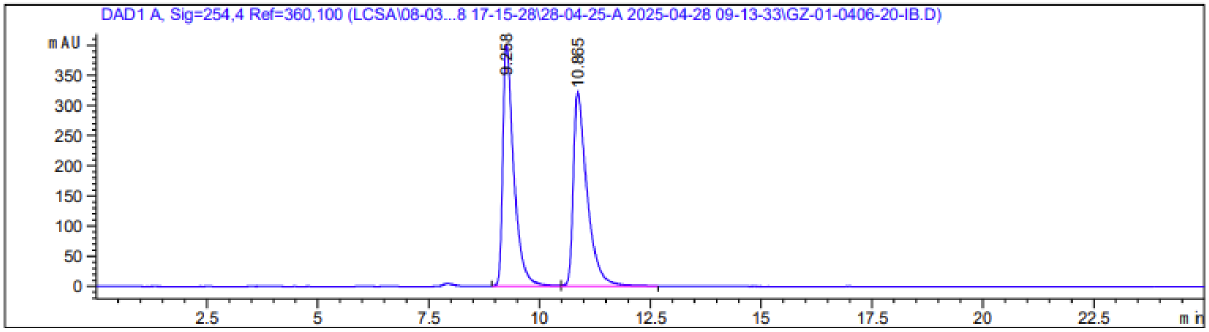

Signal 1: DAD1 A, Sig=254,4 Ref=360,100

Peak #	RetTime [min]	Type	Width [min]	Area [mAU*s]	Height [mAU]	Area %
1	9.258	BV	0.2532	6888.62061	399.47003	49.9003
2	10.865	VB	0.3133	6916.15869	321.10779	50.0997

Enantioenriched sample: **24**

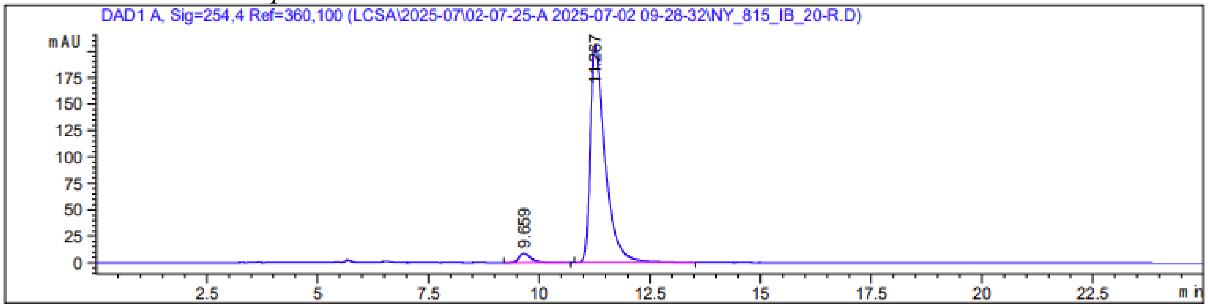

Signal 1: DAD1 A, Sig=254,4 Ref=360,100

Peak #	RetTime [min]	Type	Width [min]	Area [mAU*s]	Height [mAU]	Area %
1	9.659	BB	0.2933	170.83702	8.76717	3.4345
2	11.267	BB	0.3422	4803.31006	205.90154	96.5655

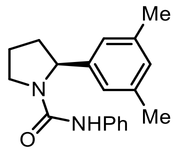

Isolated Yield: 28.2 mg, 96%
Optical Rotation: **$[\alpha]_D^{20}$** = –74.3 (c = 1, $CHCl_3$, 96:4 er).
HPLC: Chiralpak IA; eluent: *n*-hexane/*i*-propanol 90:10; flow rate: 1.0 mL/min.

Racemic sample: *rac*-**25**

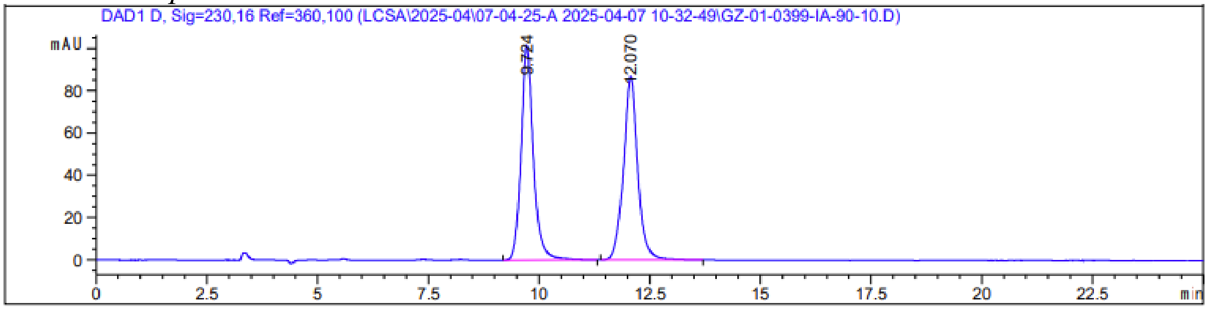

Signal 3: DAD1 D, Sig=230,16 Ref=360,100

Peak #	RetTime [min]	Type	Width [min]	Area [mAU*s]	Height [mAU]	Area %
1	9.724	BB	0.2837	1964.88135	101.55924	50.1515
2	12.070	BB	0.3263	1953.00964	86.85226	49.8485

Enantioenriched sample: **25** (with *(R)*-CPA-**5**)

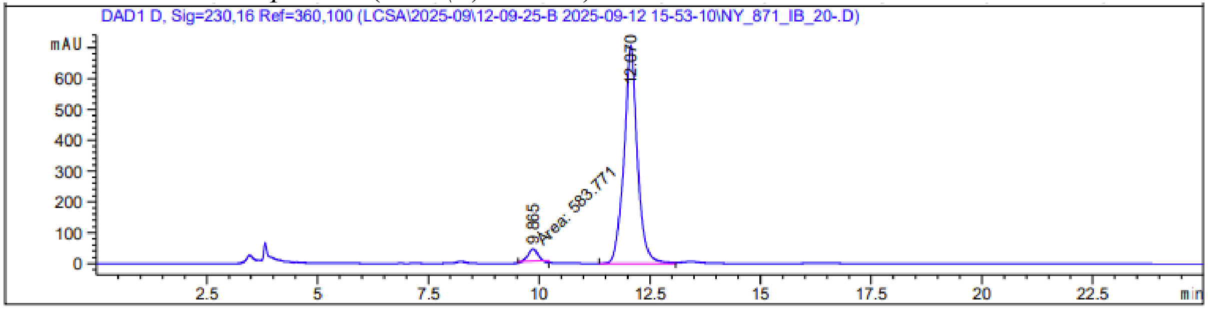

Signal 3: DAD1 D, Sig=230,16 Ref=360,100

Peak #	RetTime [min]	Type	Width [min]	Area [mAU*s]	Height [mAU]	Area %
1	9.865	MM	0.2454	583.77142	39.64289	3.7748
2	12.070	BV	0.3020	1.48811e4	705.76563	96.2252

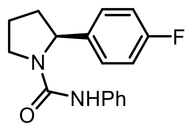

Isolated Yield: 28.2 mg, 99%
Optical Rotation: **$[\alpha]_D^{20}$** = –68.3 (c = 1, $CHCl_3$, 97:3 er).
HPLC: Chiralpak IB; eluent: *n*-hexane/*i*-propanol 90:10; flow rate: 1.0 mL/min.

Racemic sample: *rac*-**26**

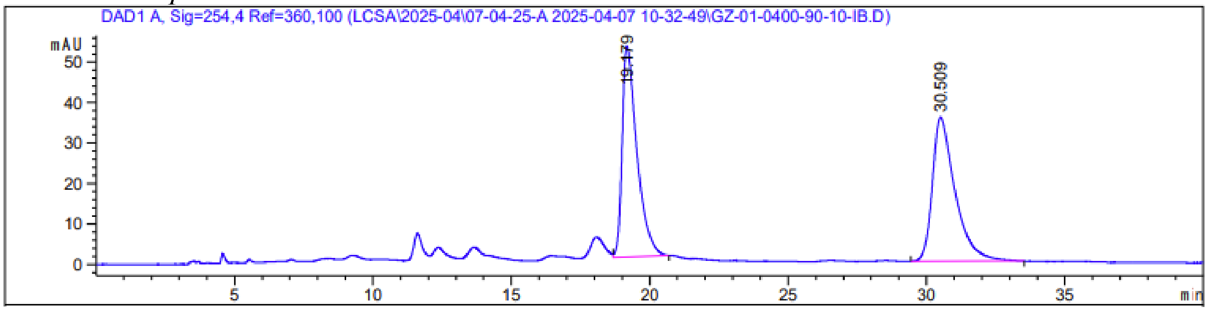

Signal 1: DAD1 A, Sig=254,4 Ref=360,100

Peak #	RetTime [min]	Type	Width [min]	Area [mAU*s]	Height [mAU]	Area %
1	19.179	VB	0.5360	1929.16479	52.10355	48.6273
2	30.509	BB	0.8128	2038.08167	35.65855	51.3727

Enantioenriched sample: **26** (with *(R)*-CPA-**5**)

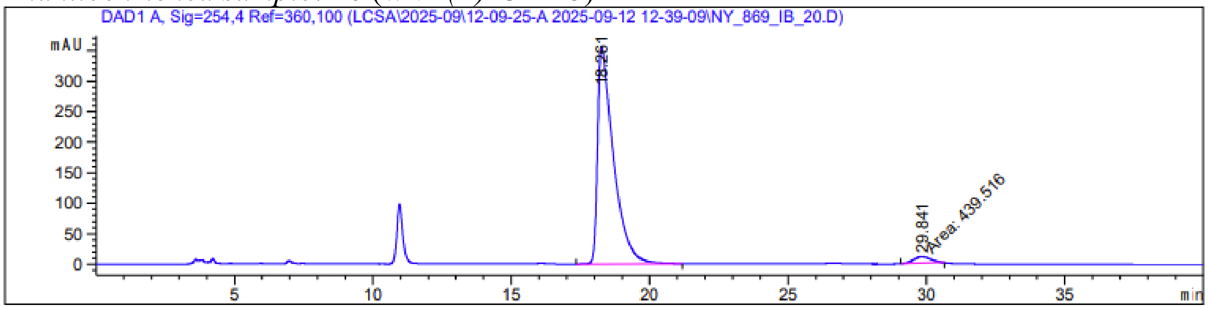

Signal 1: DAD1 A, Sig=254,4 Ref=360,100

Peak #	RetTime [min]	Type	Width [min]	Area [mAU*s]	Height [mAU]	Area %
1	18.261	BB	0.5691	1.41457e4	356.34030	96.9866
2	29.841	MM	0.6943	439.51627	10.55088	3.0134

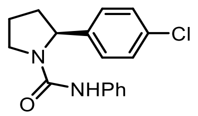

Isolated Yield: 29.2 mg, 97%
Optical Rotation: **[α]$_D^{20}$** = –85.5 (c = 1, $CHCl_3$, 96:4 er).
HPLC: Chiralpak IB; eluent: *n*-hexane/*i*-propanol 80:20; flow rate: 1.0 mL/min.

Racemic sample: *rac*-**27**

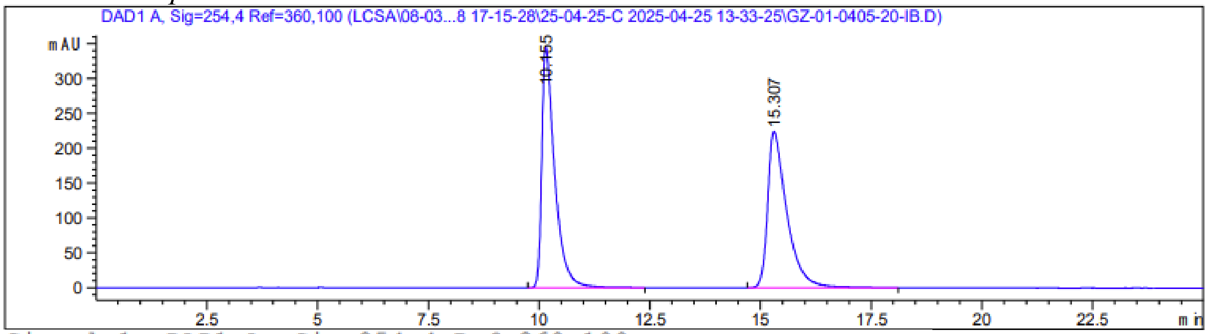

Signal 1: DAD1 A, Sig=254,4 Ref=360,100

Peak #	RetTime [min]	Type	Width [min]	Area [mAU*s]	Height [mAU]	Area %
1	10.155	BB	0.2950	7010.17090	345.03592	50.8986
2	15.307	BB	0.4402	6762.64453	224.00067	49.1014

Enantioenriched sample: **27** (with *(R)*-CPA-**5**)

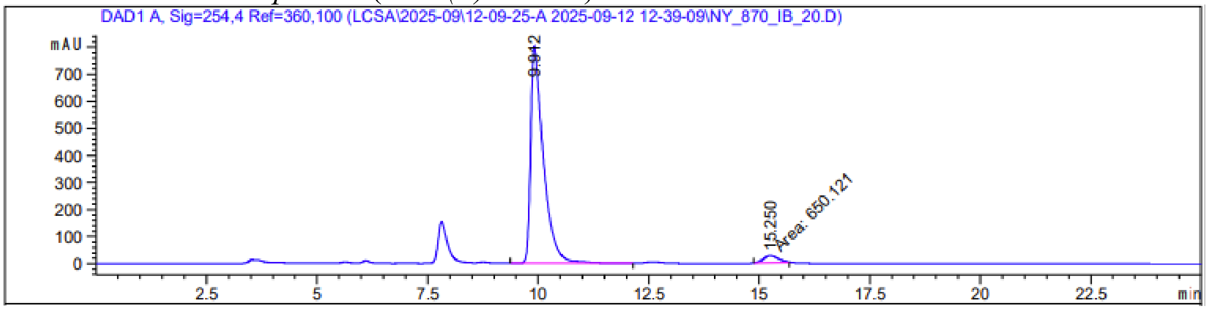

Signal 1: DAD1 A, Sig=254,4 Ref=360,100

Peak #	RetTime [min]	Type	Width [min]	Area [mAU*s]	Height [mAU]	Area %
1	9.912	BB	0.2896	1.60896e4	803.53003	96.1163
2	15.250	MM	0.3988	650.12128	27.17325	3.8837

Reactions with Enantiopure Substrates: Interconversions

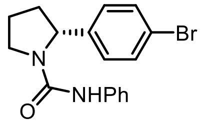

Isolated Yield: 32.0 mg, 93%
Optical Rotation: $[\alpha]_D^{20}$ = +86 (c = 1, $CHCl_3$, 93:7 er).
HPLC: Chiralpak IB; eluent: *n*-hexane/*i*-propanol 80:20; flow rate: 1.0 mL/min.

Enantioenriched sample before reaction: (S)-**7**

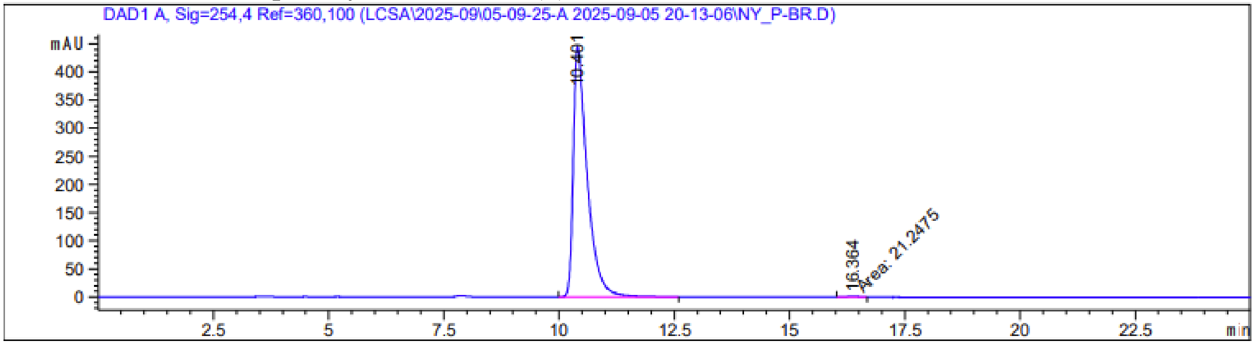

Signal 1: DAD1 A, Sig=254,4 Ref=360,100

Peak #	RetTime [min]	Type	Width [min]	Area [mAU*s]	Height [mAU]	Area %
1	10.401	BB	0.3074	9368.98828	445.34866	99.7737
2	16.364	MM	0.4013	21.24747	8.82421e-1	0.2263

Sample after irradiation: **7**

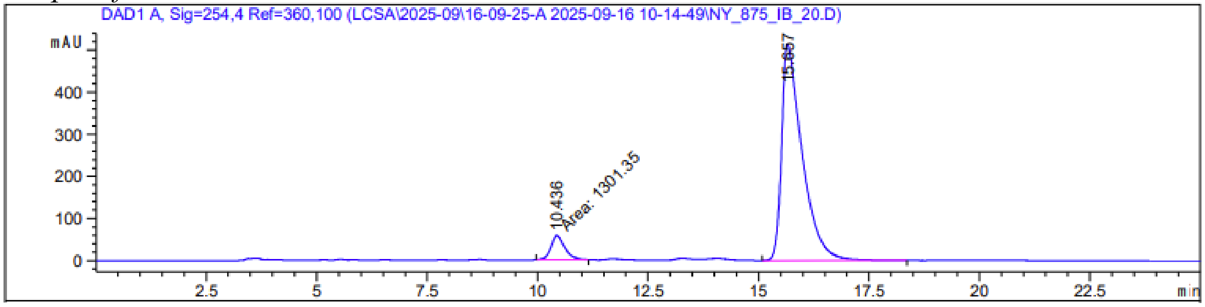

Signal 1: DAD1 A, Sig=254,4 Ref=360,100

Peak #	RetTime [min]	Type	Width [min]	Area [mAU*s]	Height [mAU]	Area %
1	10.436	MM	0.3686	1301.35120	58.83467	7.2729
2	15.657	BB	0.4625	1.65919e4	514.17419	92.7271

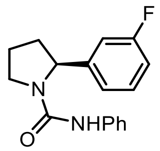

Isolated Yield: 27.6 mg, 97%
Optical Rotation: $[\alpha]_D^{20}$ = –76.33 (c = 1, $CHCl_3$, 94:6 er).
HPLC: Chiralpak IB; eluent: *n*-hexane/*i*-propanol 80:20; flow rate: 1.0 mL/min.

*Enantioenriched sample before reaction: (R)-***15**

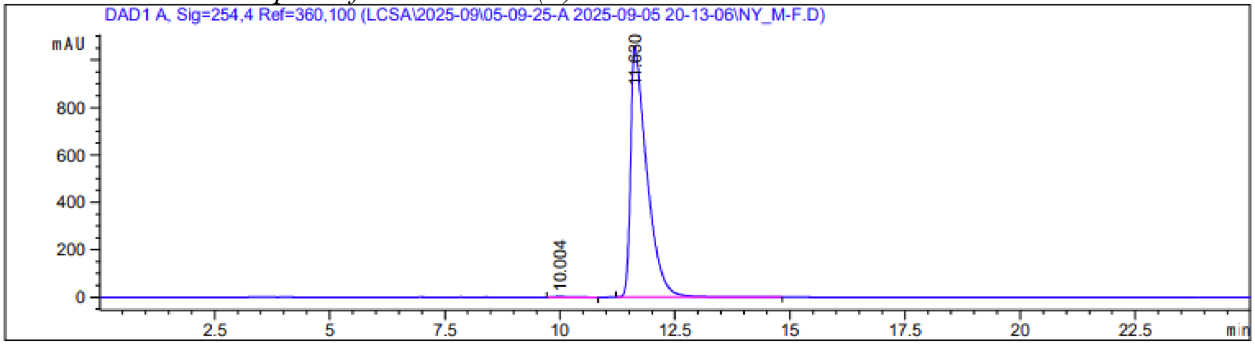

Signal 1: DAD1 A, Sig=254,4 Ref=360,100

Peak #	RetTime [min]	Type	Width [min]	Area [mAU*s]	Height [mAU]	Area %
1	10.004	BB	0.3049	62.64076	2.98391	0.2414
2	11.630	BB	0.3543	2.58885e4	1054.92432	99.7586

Sample after irradiation: *ent*-**15**

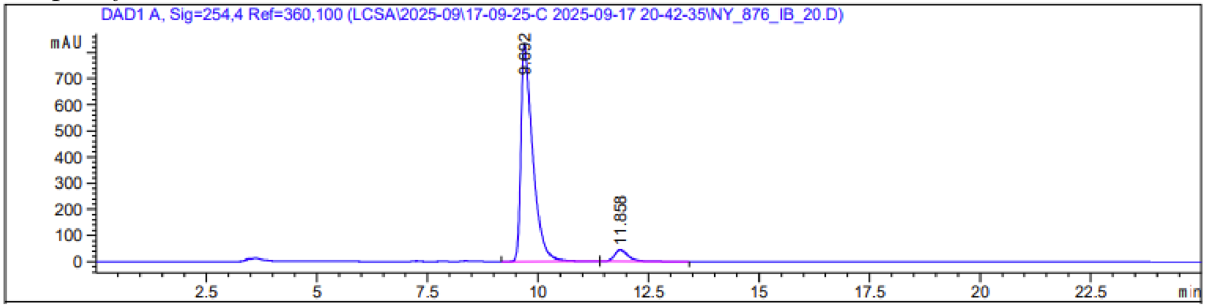

Signal 1: DAD1 A, Sig=254,4 Ref=360,100

Peak #	RetTime [min]	Type	Width [min]	Area [mAU*s]	Height [mAU]	Area %
1	9.692	BB	0.2721	1.55437e4	831.37537	94.1818
2	11.858	BB	0.3188	960.23352	45.02652	5.8182

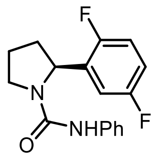

Isolated Yield: 28.5 mg, 94%
Optical Rotation: **[α]$_D$^{20}** = –80.2 (c = 1, $CHCl_3$, 95:5 er).
HPLC: Chiralpak IB; eluent: *n*-hexane/*i*-propanol 80:20; flow rate: 1.0 mL/min.

Enantioenriched sample before reaction: *(R)*-**24**

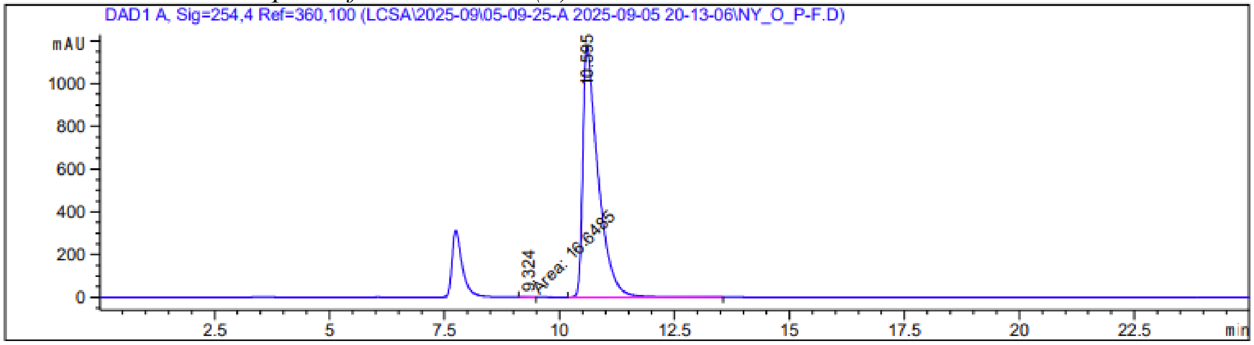

Signal 1: DAD1 A, Sig=254,4 Ref=360,100

Peak #	RetTime [min]	Type	Width [min]	Area [mAU*s]	Height [mAU]	Area %
1	9.324	MM	0.2682	16.64846	1.03445	0.0610
2	10.595	BB	0.3356	2.72618e4	1171.17139	99.9390

Sample after irradiation: *ent*-**24**

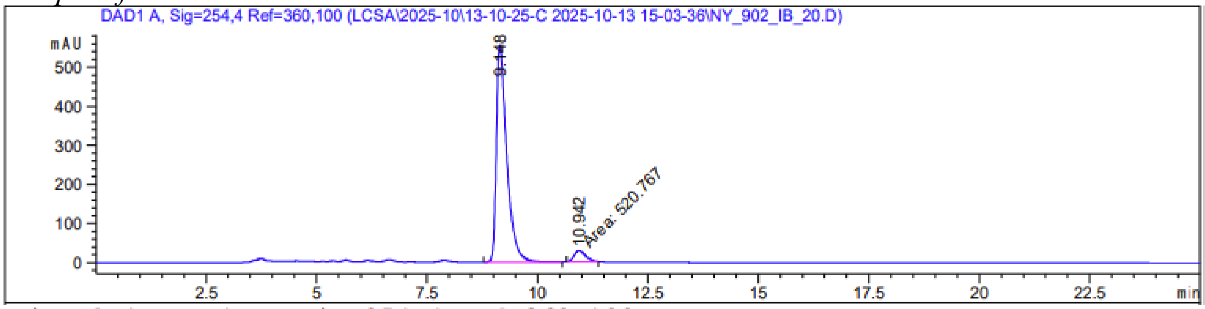

Signal 1: DAD1 A, Sig=254,4 Ref=360,100

Peak #	RetTime [min]	Type	Width [min]	Area [mAU*s]	Height [mAU]	Area %
1	9.148	BB	0.2386	9065.24316	554.90576	94.5674
2	10.942	MM	0.3033	520.76733	28.62133	5.4326

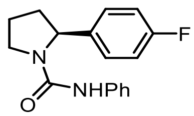

Isolated Yield: 27.4 mg, 96%
Optical Rotation: $[\alpha]_D^{20}$ = –66.8 (c = 1, $CHCl_3$, 96:4 er).
HPLC: Chiralpak IB; eluent: *n*-hexane/*i*-propanol 90:10; flow rate: 1.0 mL/min.

Enantioenriched sample before reaction: *ent*-**26**

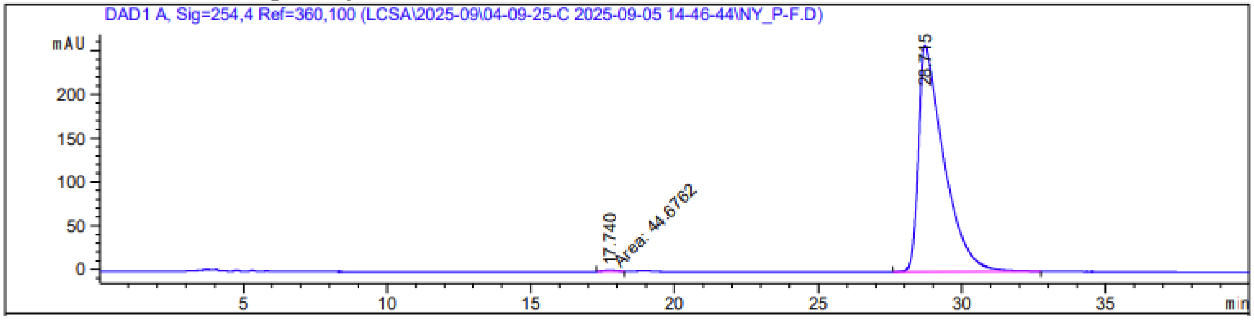

Signal 1: DAD1 A, Sig=254,4 Ref=360,100

Peak #	RetTime [min]	Type	Width [min]	Area [mAU*s]	Height [mAU]	Area %
1	17.740	MM	0.5314	44.67623	1.40110	0.2817
2	28.715	BB	0.8221	1.58172e4	258.42947	99.7183

Sample after irradiation: ***(S)*-26**

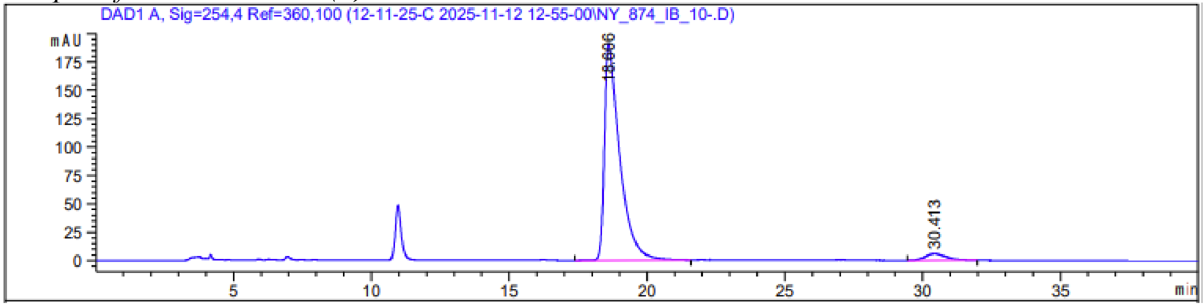

Signal 1: DAD1 A, Sig=254,4 Ref=360,100

Peak #	RetTime [min]	Type	Width [min]	Area [mAU*s]	Height [mAU]	Area %
1	18.606	BB	0.5561	7358.46191	190.72922	95.9019
2	30.413	BB	0.7362	314.44397	5.91543	4.0981

Removal of the Urea Protecting Group

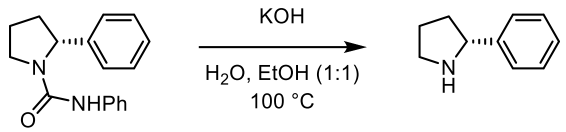

1
97:3 er

1a
92%, 97:3 er

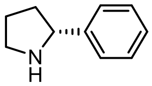

(*R*)-2-phenylpyrrolidine (1a): (*R*)-*N*,2-diphenylpyrrolidine-1-carboxamide (21.0 mg, 78.9 µmol, 1.0 equiv) was dissolved in a mixture of EtOH and water (4 mL, 1:1) and KOH (100 mg, 1.78 mmol, 22 equiv) was added. The reaction mixture was stirred at 100 °C for 48 h and then quenched by the addition of HCl (1 mL, 1 M). The pH was adjusted to 9-10 by the addition of sat. Na_2CO_3 solution and the aqueous phase was extracted with CH_2Cl_2 (3x). The combined organic layers were dried over Na_2SO_4, filtered and concentrated. The product was purified by flash column chromatography on silica gel (EtOAc/MeOH, 9:1→ EtOAc/MeOH/*i*$PrNH_2$, 9:1:0.1) to obtain the title compound (10.7 mg, 72.0 µmol, 92% yield) as a brown oil.
The characterization data are comparable to the data reported in the literature.[4] The title compound was derivatized to determine the enantiomeric ratio.

(*R*)-2-phenylpyrrolidine (10.7 mg, 72.0 µmol, 1.0 equiv) was dissolved in dry CH_2Cl_2 (1.0 mL) and NEt_3 (40.6 µL, 291 µmol, 4.0 equiv) was added. The reaction mixture was cooled 0 °C and bezoyl chloride (10.2 µL, 87.2 µmol, 1.2 equiv) was added dropwise. The reaction mixture was stirred at room temperature until full consumption of the starting material and then quenched by the addition of sat NH_4Cl. The aqueous phase was extracted with CH_2Cl_2 (3x 2.0 mL) and the combined organic layers were washed with Brine, dried over Na_2SO_4, filtered and concentrated. The residue was purified by preparative TLC (1:1 hexane/EtOAc). White solid. The enantiomeric ratio was measured by HPLC.

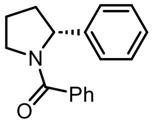

HPLC: Chiralpak IB; eluent: *n*-hexane/*i*-propanol 90:10; flow rate: 1.0 mL/min.

Racemic sample:

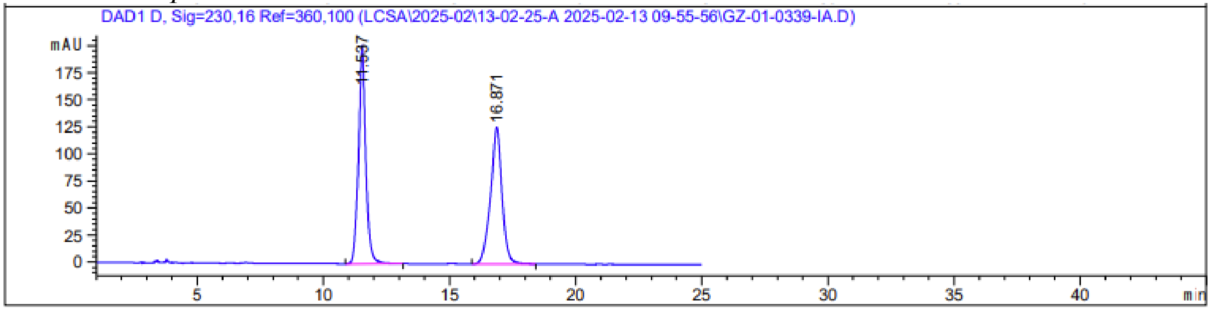

Signal 3: DAD1 D, Sig=230,16 Ref=360,100

Peak #	RetTime [min]	Type	Width [min]	Area [mAU*s]	Height [mAU]	Area %
1	11.537	BB	0.2877	4026.51587	200.97847	50.0848
2	16.871	BB	0.4561	4012.87305	126.51298	49.9152

Enantioenriched sample:

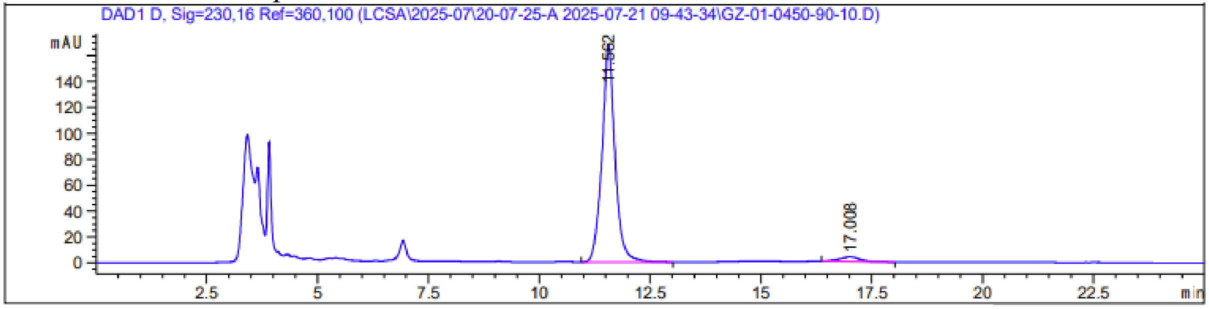

Signal 3: DAD1 D, Sig=230,16 Ref=360,100

Peak #	RetTime [min]	Type	Width [min]	Area [mAU*s]	Height [mAU]	Area %
1	11.562	BB	0.2917	3423.12085	168.01884	96.8638
2	17.008	BB	0.4020	110.83117	3.67373	3.1362

Assignments of Absolute Configuration

The specific rotations and the HPLC retention times of *(R)*-**1** and *(S)*-**1** (prepared from commercial *(R)*-2-phenylpyrrolidine and *(S)*-2-phenylpyrrolidine following **GP-2**) were compared to those of the deracemization products formed from *rac*-**1**, which determined that *(R)*-**1** was the major enantiomer in the deracemization product using *(S)*-CPA-**5**. The absolute configurations of other substrates were inferred by analogy.

Mechanistic Studies

Synthesis of Potassium Salts of (*S*)-CPAK, PySK-1 and PySK-2

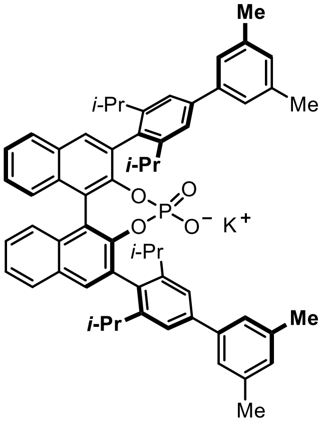

(*S*)-Potassium 2,6-bis(3,5-diisopropyl-3',5'-dimethyl-[1,1'-biphenyl]-4-yl)-4-hydroxydinaphtho[2,1-*d*:1',2'-*f*][1,3,2]dioxaphosphepine 4-oxide ((*S*)-CPAK): In a nitrogen-filled glovebox a 4 mL vial was charged with (*S*)-CPA-**5** (200 mg, 0.228 mmol, 1.0 equiv) and KO^{t}Bu (25.6 mg, 0.228 mmol, 1.0 equiv) and dissolved in CH_2Cl_2:MeOH (2 mL, 1:1). The reaction mixture was stirred for 3 h at room temperature and then the solvent was removed in vacuo. The residue was dried at the high vacuum to afford the title compound (207 mg, 0.226 mmol, 99%) as a white solid.

M.p. = >300 °C (CH_2Cl_2/MeOH). **^{1}H NMR** (400 MHz, CD_2Cl_2) δ 7.92 (d, *J* = 8.2 Hz, 2H), 7.77 (s, 2H), 7.51 – 7.41 (m, 2H), 7.36 – 7.29 (m, 4H), 7.29 – 7.23 (m, 4H), 7.11 (s, 4H), 6.82 (s, 2H), 2.86 – 2.63 (m, 4H), 2.10 (s, 12H), 1.15 (dd, *J* = 9.9, 6.8 Hz, 12H), 0.95 (d, *J* = 6.8 Hz, 12H). **^{13}C NMR** (101 MHz, CD_2Cl_2) δ 149.3 (d, *J* = 40.0 Hz), 148.2 (d, *J* = 9.3 Hz), 141.6 (d, *J* = 19.5 Hz), 138.7, 135.2, 133.2, 132.3 (d, *J* = 2.7 Hz), 132.0, 130.8, 129.2, 128.4, 127.6, 126.2, 125.3, 123.0 (d, *J* = 2.2 Hz), 122.1, 121.6, 31.4, 31.3, 26.2, 25.0, 23.7, 23.4, 21.1. **^{31}P NMR** (162 MHz, CD_2Cl_2) δ 5.9. **HRMS** (ESI/QTOF) *m/z*: $[M]^-$ Calcd for $C_{60}H_{60}O_4P^-$ 875.4235; Found 875.4240. **IR** (ATR): 2963, 1598, 1258, 1098, 897, 752, 709 cm^{-1}. **$[\alpha]_D^{20}$** = +185.3 ($CHCl_3$, 1.0).

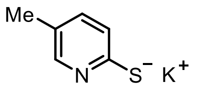

Potassium 5-methylpyridine-2-thiolate (PySK-1): In a nitrogen-filled glovebox a 4 mL vial was charged with 5-methylpyridine-2-thiol (51.9 mg, 0.415 mmol, 1.0 equiv) and KO^{t}Bu (46.5 mg, 0.415 mmol, 1.0 equiv) and THF (0.70 mL) was added. The suspension was stirred for 1 h at room temperature and then, the solvent was removed in vacuo. The residue was dried at the high vacuum to afford the title compound (65.6 mg, 0.402 mmol, 97%) as a white solid.

^{1}H NMR (400 MHz, CD_3CN) δ 7.73 (dt, *J* = 2.7, 0.9 Hz, 1H), 7.02 (d, *J* = 8.1 Hz, 1H), 6.82 (ddd, *J* = 8.1, 2.6, 0.7 Hz, 1H), 2.06 (s, 3H). **^{13}C NMR** (101 MHz, CD_3CN) δ 177.7, 148.5, 135.2, 129.4, 122.0, 17.7. **HRMS** (APCI/QTOF) *m/z*: $[M]^-$ Calcd for $C_6H_6NS^-$ 124.0226; Found 124.0224. **IR** (ATR): 3302, 2920, 2863, 1593, 1539, 1453, 1362, 1107, 820, 491 cm^{-1}.

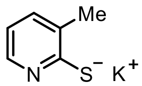

Potassium 3-methylpyridine-2-thiolate (PySK-2): In a nitrogen-filled glovebox a 4 mL vial was charged with 3-methylpyridine-2-thiol (15.5 mg, 120 µmol, 1.0 equiv) and KO^tBu (13.5 mg, 120 µmol, 1.0 equiv) and toluene (1.5 mL) was added. The suspension was stirred for 1 h at room temperature and then, the solvent was removed in vacuo. The residue was dried at the high vacuum to afford the title compound (18.0 mg, 110 µmol, 92%) as a white solid.
^{1}**H NMR** (400 MHz, CD_3CN) δ 7.80 (dd, J = 4.9, 2.0 Hz, 1H), 7.06 (dd, J = 7.2, 2.0, Hz, 1H), 6.43 (dd, J = 7.1, 4.9 Hz, 1H), 2.22 (s, 3H). ^{13}**C NMR** (101 MHz, CD_3CN) δ 146.0, 135.6, 134.0, 113.6, 31.4, 23.6. **HRMS** (APCI/QTOF) *m/z*: $[M]^-$ Calcd for $C_6H_6NS^-$ 124.0226; Found 124.0232. **IR** (ATR): 3346, 3321, 1648, 1577, 1383, 1306, 1261, 1086, 825, 801, 774 cm^{-1}.

Synthesis of Deuterated Substrate *rac*-1-d_1

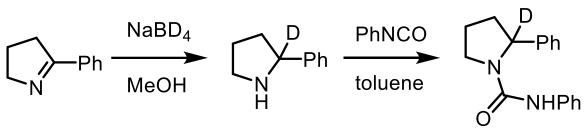

rac-**1a**-d_1 *rac*-**1**-d_1

5-Phenyl-3,4-dihydro-2*H*-pyrrole[5] (200 mg, 1.37 mmol, 1.0 equiv) was dissolved in dry MeOH (6.9 mL) in a flame-dried Schlenk flask. The solution was cooled to 0 °C and $NaBD_4$ (172 mg, 4.11 mmol, 3.0 equiv) was added. The reaction was allowed to reach room temperature and stirred for additional 5 h. After consumption of the starting material (reaction monitoring by TLC) the volatiles were evaporated, and the residue was partitioned between CH_2Cl_2 and water. The phases were separated, and the water layer was extracted with CH_2Cl_2. The combined organic layers were dried over Na_2SO_4 filtered and concentrated to obtain 2-phenylpyrrolidine-2-*d* as a brown oil. The crude material was taken into the next step without further purification.
A 4 mL vial was charged with a solution of 2-phenylpyrrolidine-2-*d* (187 mg, 1.27 mmol, 1.0 equiv) and toluene (0.6 mL). Phenyl isocyanate (152 µL, 1.40 mmol, 1.1 quiv) was added to the solution and the reaction mixture was stirred for 1 h at room temperature. The crude material was purified by precipitation from hexane/Et_2O (5:1) to obtain *rac*-**1**-d_1 as a white solid. (297 mg, 1.11 mmol, 87%, D/H: 93:7)
M.p. = 141 – 143 °C (hexane/Et_2O). ^{1}**H NMR** (400 MHz, $CDCl_3$) δ 7.44 – 7.36 (m, 2H), 7.36 – 7.28 (m, 3H), 7.21 – 7.15 (m, 2H), 7.15 – 7.07 (m, 2H), 6.94 (td, J = 7.1, 1.4 Hz, 1H), 6.02 (s, 1H), 3.89 – 3.79 (m, 1H), 3.79 – 3.69 (m, 1H), 2.51 – 2.37 (m, 1H), 2.08 – 1.83 (m, 3H). ^{13}**C NMR** (101 MHz, $CDCl_3$) δ 154.4, 142.8, 139.2, 129.4, 128.9, 128.1, 126.0, 122.8, 119.4, 61.8 – 60.5 (m), 47.7, 37.0, 23.3. **HRMS** (nanochip-ESI/LTQ-Orbitrap) *m/z*: $[M + H]^+$ Calcd for $C_{17}H_{18}[^2H]N_2O^+$ 268.1555; Found 268.1551. **IR** (ATR): 3308, 2972, 1648, 1594, 1531, 1500, 1441, 1367, 1244, 752, 694 cm^{-1}.

Separation of enantiomers

To access the deuterated substrate in optically enriched form, *rac*-**1**-d_1 was separated on a semi-preparative HPLC (CHIRALPAK IB, 95:5 hexane/IPA, rate 18 mL / min, 254 nm). For each run, 30 mg of the *rac*-**1**-d_1 was dissolved in CH_2Cl_2 (approx. 0.5 mL) and injected as a solution.

Time-Course Profile of Deracemization

The evaluation of ee as a function of time was conducted under standard conditions for *rac*-**1**, *(R)*-**1** and *(S)*-**1** with *(S)*-CPA-**5**. 20 µL of the reaction mixture were taken out for HPLC analysis at various time points. All the three substrates converged to *(R)*-**1** and established the steady-state enantiomeric ratio of 97:3.

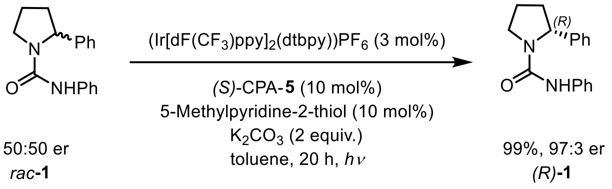

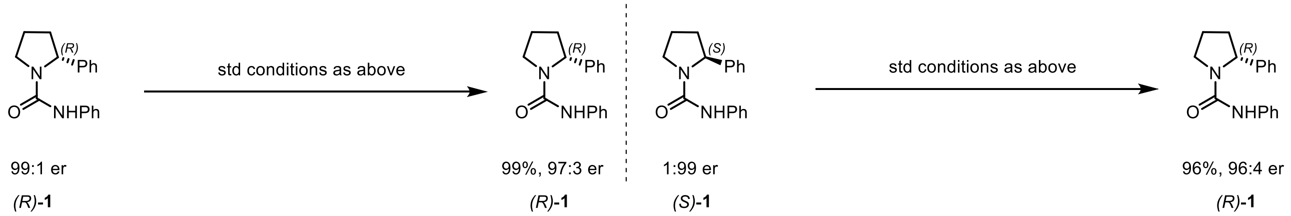

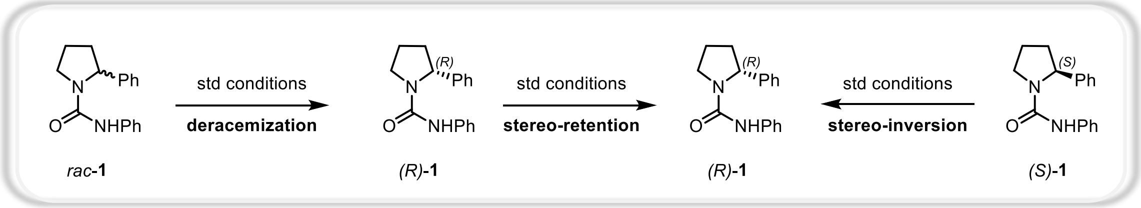

According to Method B, in a nitrogen-filled glovebox, three separate oven-dried 4 mL vials were charged with *rac*-**1**, *(R)*-**1**, and *(S)*-**1** (26.6 mg, 0.1 mmol, 1.0 equiv), respectively. Then all the vials were individually charged with 5-methylpyridine-2-thiol (1.25 mg, 10.0 µmol, 10 mol%), *(S)*-CPA-**5**, (8.77 mg, 10.0 µmol, 10 mol%), (Ir[dF(CF_3)ppy]$_2$(dtbpy))PF_6 (3.37 mg, 3.0 µmol, 3 mol%), and a magnetic stir bar. Then anhydrous toluene (2 mL) was added in each vial, and the vials were sealed with a PTFE-lined screw cap. The reaction vials were taken out of the glovebox and irradiated with Penn M2 photoreactor (420 nm). During the reactions, aliquots were taken from the solutions simultaneously at various intervals. The aliquot solvent was removed under reduced pressure and subjected to purification by preparative TLC. The enantiomeric ratios were determined by HPLC analysis on a chiral stationary phase.

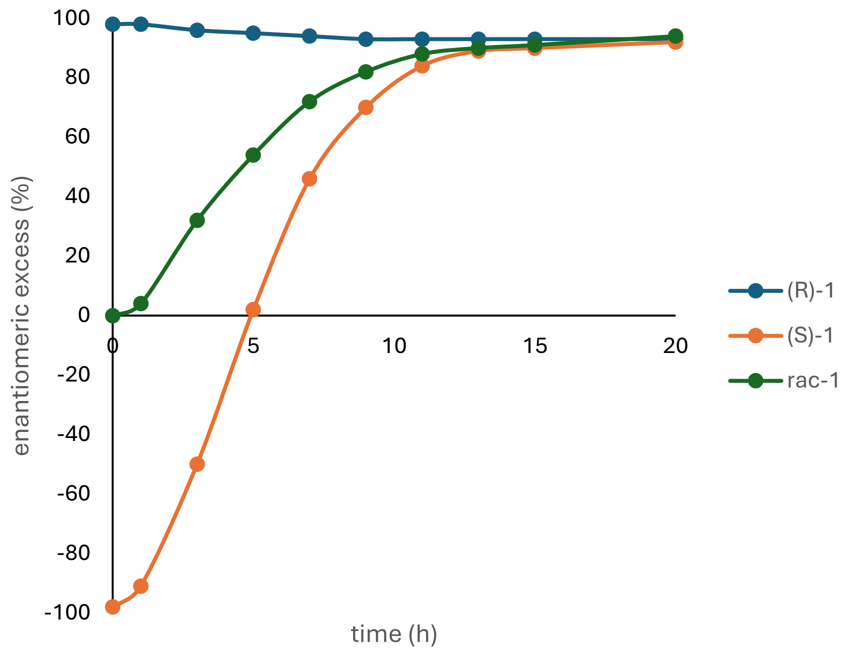

Fig. S1. Kinetic analysis of the deracemization of *rac*-**1**, *(R)*-**1**, and *(S)*-**1**

Stern-Volmer Luminescence Quenching and UV-Vis Experiments

Stern-Volmer quenching experiments were performed in degassed EtOAc, using PySK-**2** due to its higher solubility. Under these reaction conditions photochemical deracemization is achieved with 95% yield and 66:34 er. The solutions were irradiated at 383 nm in a quartz-glass cuvette and luminescence was measured at 476 nm under inert atmosphere.

In a nitrogen-filled glovebox, a 10 mL volumetric flask was charged with 1 mL of a (Ir[dF(CF_3)ppy]$_2$(dtbpy))PF_6 photocatalyst stock solution (10^{-4} M) and varying amounts of a quencher solution (10^{-3} M) (0, 0.1 mL, 0.2 mL, 0.4 mL, 0.6 mL). Degassed EtOAc was added to reach the total volume of 10 mL. The quartz cuvette was filled with 3 mL of the latter solution. The luminescence quenching studies were performed with single reaction components including *rac*-**1**, (*S*)-CPAK, PySK-**2** and a previously stirred (1 h), equimolar mixture of (*S*)-CPAK and PySK-**2**.

Luminescence quenching with *rac*-**1**

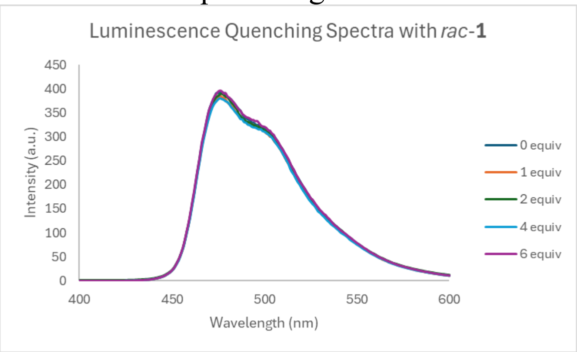

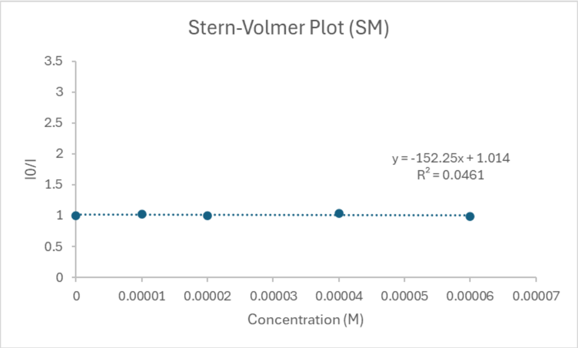

Luminescence quenching with (*S*)-CPAK

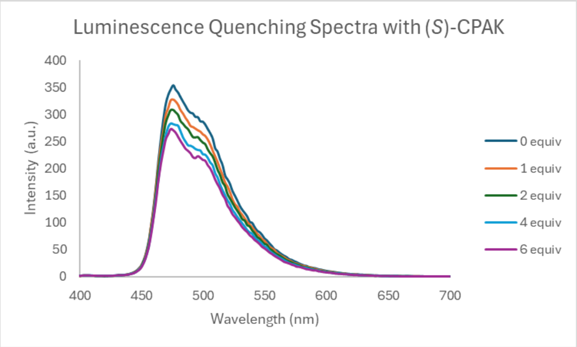

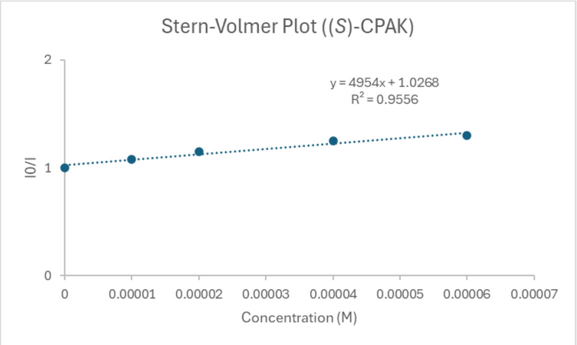

Luminescence quenching with PySK-**2**

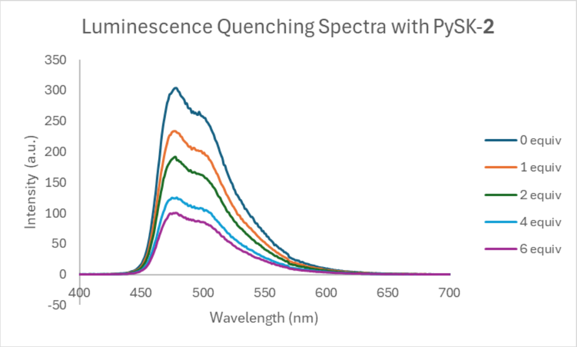

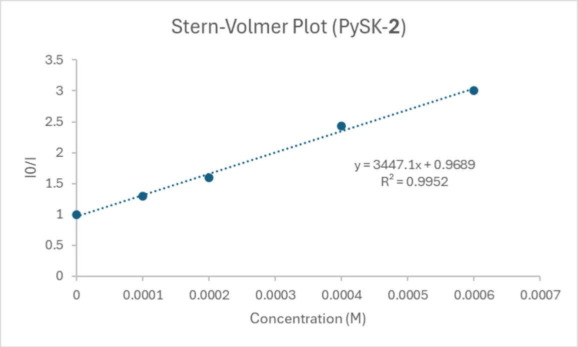

Luminescence quenching with a prestirred, 1:1 solution of (*S*)-CPAK and PySK-**2**

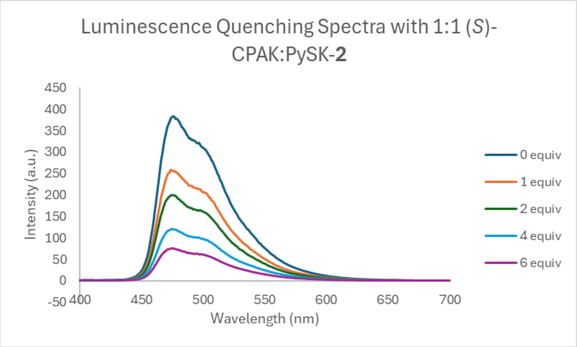

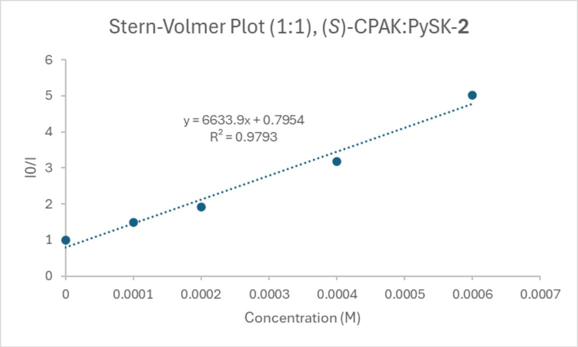

The substrate *rac*-**1** does not quench the excited state of the $(Ir[dF(CF_3)ppy]_2(dtbpy))PF_6$ photocatalyst. Although quenching activities for (*S*)-CPAK and PySK-**2** have been recorded, no deracemization occurs in the absence of either catalyst. A prestirred, equimolar solution of (*S*)-CPAK and PySK-**2** showed the fastest quenching activity indicating the formation of a distinct species.

Luminescence quenching with a prestirred, 1:1:1 solution of (*S*)-CPAK, PySK-**2** and (*S*)-**1**

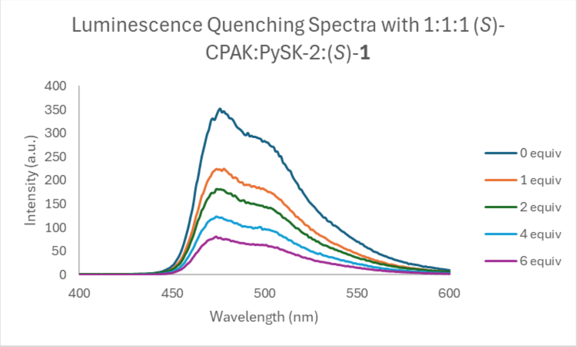

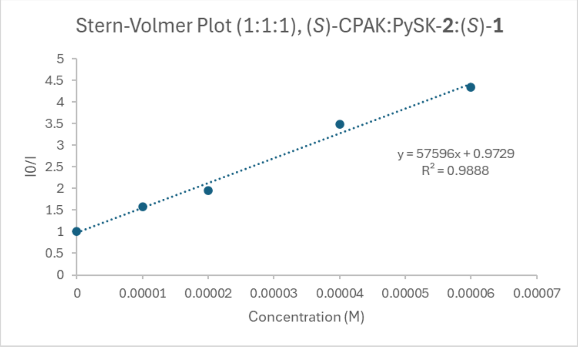

Luminescence quenching with a prestirred, 1:1:1 solution of (*S*)-CPAK, PySK-**2** and (*R*)-**1**

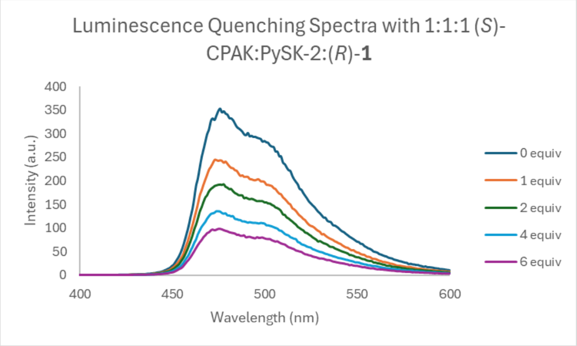

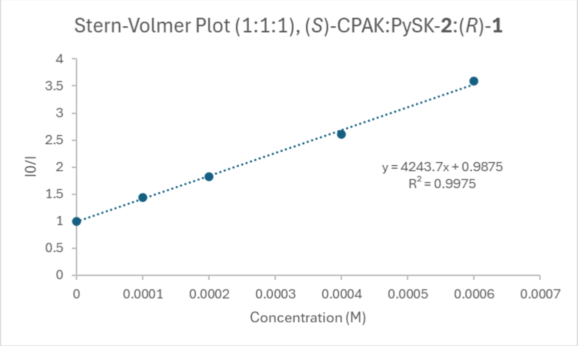

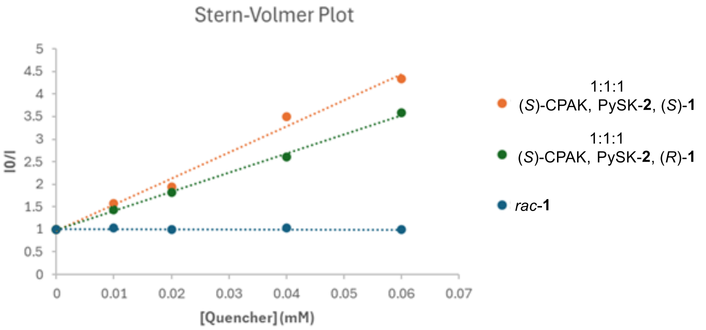

Stern-Volmer luminescence analysis of a triple combination (*S*)-CPAK, PySK-**2** and (*S*)- or (*R*)-**1** revealed a faster quenching activity for the matched (*S*)-**1** substrate. This is consistent with a selective HAA at one enantiomeric substrate.

Luminescence quenching with a prestirred, 1:1 solution of *rac*-CPAK and *rac*-**1**

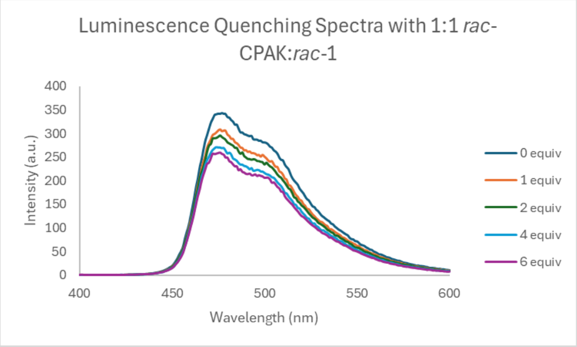

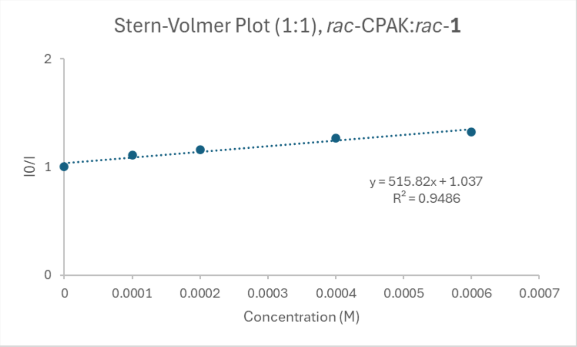

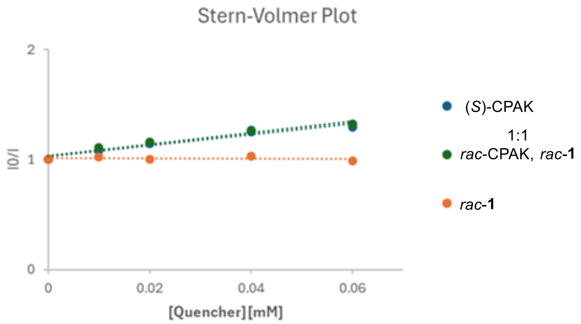

Stern-Volmer Luminescence analysis of a 1:1 combination of *rac*-CPAK and *rac*-**1** revealed the same quenching activity as (*S*)-CPAK. Thus, the addition of *rac*-**1** does not alter the quenching activity of (*S*)-CPAK. This is consistent with our previous observation that the substrate does not show any quenching activity.

Job Plot Analysis of Catalyst Components

Job plot analysis of catalyst components were performed in degassed EtOAc using PySK-**2** due to its increased solubility.

In a nitrogen-filled glovebox, separate 10 mL volumetric flasks were charged with either (*S*)-CPAK (45.8 mg, 0.05 mmol) or PySK-**2** (8.2 mg, 0.05 mmol) and filled with ethyl acetate to create stock solutions of $5 \cdot 10^{-3}$ M concentration. Solutions of varying mole fractions were made by adding varying amounts of the (*S*)-CPAK, and PySK-**2** solutions and ethyl acetate to generate solutions with varying mol fractions (χ) of thiolate inside of 4 mL volume quartz cuvettes.

Solutions for Job Plot Analysis

PySK-**2** solution (μL)	(*S*)-CPAK solution (μL)	Ethyl Acetate (μL)	Final volume (μL)	$X_{thiolate}$
2000	0	1000	3000	1.00
1600	400	1000	3000	0.80
1500	500	1000	3000	0.75
1222	666	1000	3000	0.66
1000	1000	1000	3000	0.50
666	1222	1000	3000	0.33
500	1500	1000	3000	0.25
400	1600	1000	3000	0.20

0	2000	1000	3000	0.00

UV-Vis absorption curve for Job plot analysis solutions

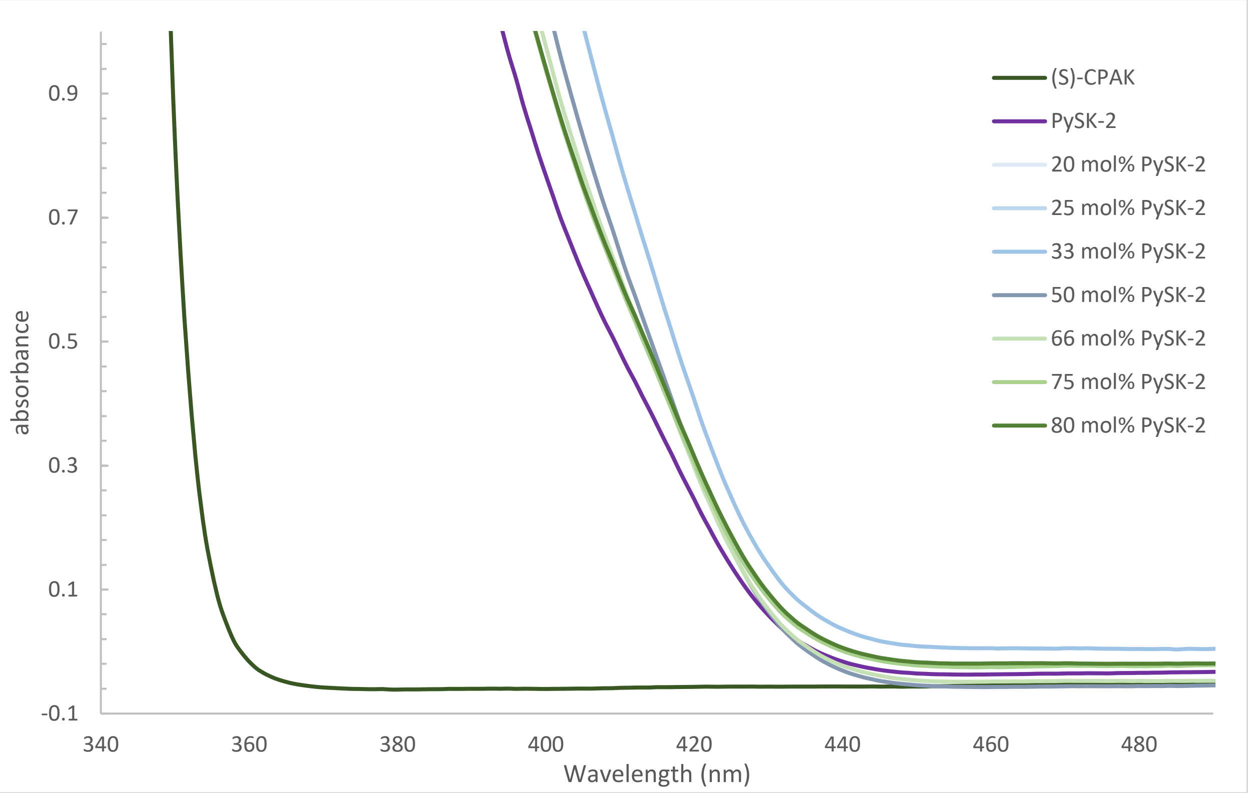

To determine the optimal ratio of (*S*)-CPAK, and PySK-**2** for complex formation, the change in observed absorbance at 410 nm (ΔA) multiplied by the mole fraction of PySK-**2** (χ) was plotted with respect to mol fraction of PySK-**2**. ΔA was calculated by following formula:

$$\Delta A^{410} = A_{obs}^{410\,nm} - A_{free}^{410\,nm} \cdot \chi$$

Where $A_{obs}^{410\,nm}$ is the observed absorbance at 410 nm, and $A_{free}^{410\,nm}$ is the absorbance of the solution when only thiol is present.

The linear fit of the first four points and last four points intersect closest to χ = 0.5. This implies that the optimal stoichiometry of the (*S*)-CPAK, and PySK-**2** complex is 1:1.

Job plot of $\Delta A_{obs}^{410\,nm}$•χ versus mole fraction of thiol (χ)

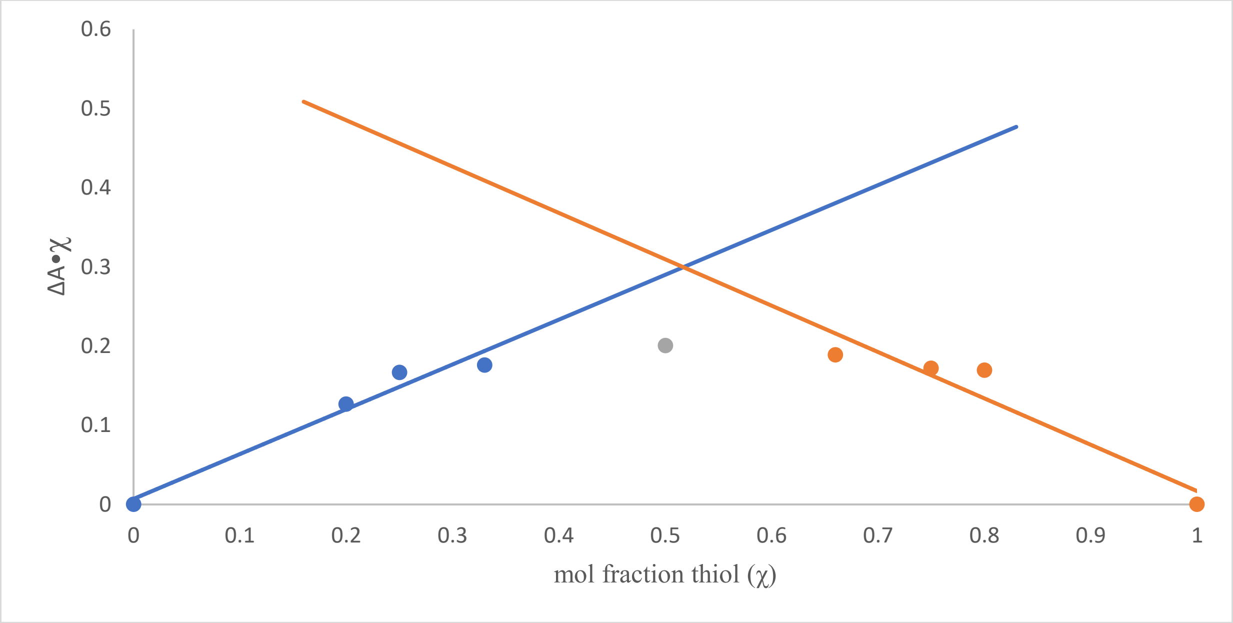

UV-Vis of 5-methylpyridine thiol (PySH-**6)** and (*S*)-CPAK

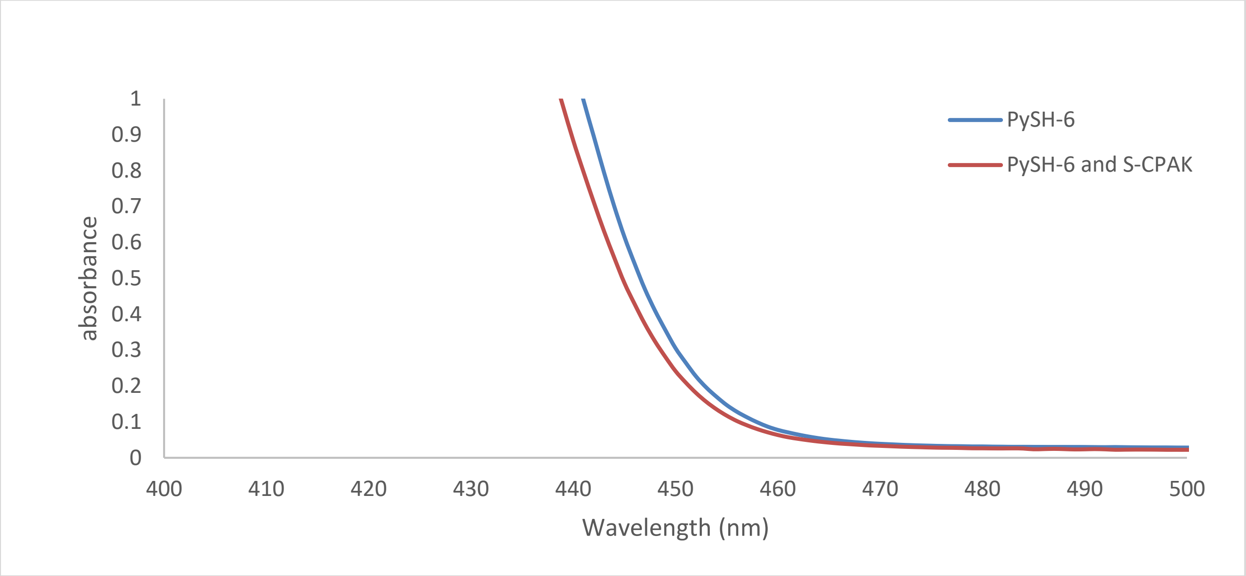

Impact of TEMPO on the Deracemization Reaction

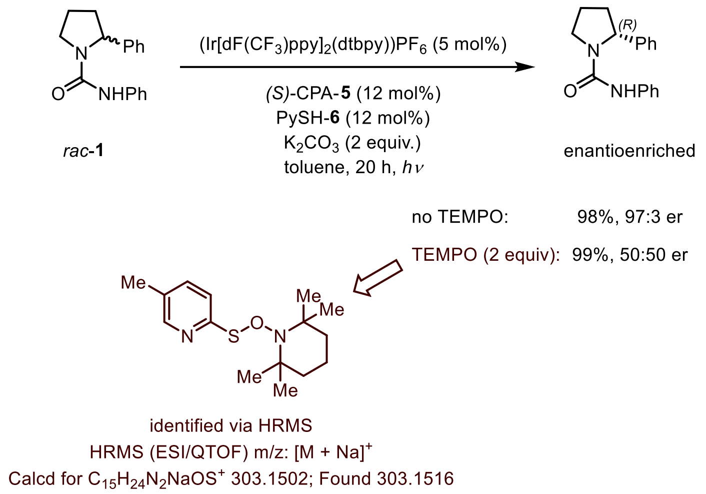

According to Method B, in a nitrogen-filled glovebox, an oven-dried 4 mL vial was charged with PySH-**6** (1.20 mg, 10.0 µmol, 10 mol%), *(S)*-CPA-**5** (8.70 mg, 10.0 µmol, 10 mol%), (Ir[dF(CF_3)ppy]$_2$(dtbpy))PF_6 (3.77 mg, 3.0 µmol, 3 mol%), K_2CO_3 (27.6 mg, 0.2 mmol, 2.0 equiv), *rac*-**1** (26.6 mg, 0.1 mmol, 1.0 equiv), 2,2,6,6-tetramethyl-1-piperidinyloxy (TMEPO) (31.3 mg, 0.2 mmol, 2.0 equiv) and a magnetic stir bar. Then, anhydrous toluene (2 mL) was added, and the vial was sealed with a PTFE-lined screw cap. The reaction was taken out of the glovebox and irradiated with Penn M2 photoreactor (420 nm) with the following settings: 100% light intensity, 6800 rpm fan cooling, 509 rpm stirring. After 20 h, the solution was concentrated, and the crude reaction mixture was analyzed via ^{1}H NMR spectroscopy and HRMS. HRMS analysis confirmed the presence of the 5-methylpyridine-2-thiol/TEMPO adduct. The enantiomeric ratios were then determined by HPLC analysis with chiral stationary phase after purification by preparative TLC.

Deuterium Labeling Experiment

Determination of the Kinetic Isotope Effect (KIE):

The rates for racemization of substrates *(S)*-**1** and of *(S)*-**1**-*d*$_1$ with (*S*)-CPA-**5** were measured using the method of initial rates.[6] For this study, the enantiopure substrates with *(S)*-configuration were used to prevent undesired side reactions or inhibition of the catalyst by the unprocessed *(R)*-configured substrates and to ensure a concentration-independent zero order reaction (y = kx + a). Moreover, only data points until a conversion of 20% (60:40 er) were considered for the linear regression to exclude catalyst inhibition by the formed enantiomer *(R)*-**1** and, thus, a deviation from zero order kinetic. The slopes of the linear regressions give rise to the rate constants k_H and k_D to deliver KIE = k_H/k_D.

According to Method B, in a nitrogen-filled glovebox, two separate oven-dried 4 mL vials were charged with *(S)*-**1** (26.6 mg, 0.1 mmol, 1.0 equiv) and *(S)*-**1**-*d*$_1$ (26.7 mg, 0.1 mmol, 1.0 equiv), respectively. Then, both vials were individually charged with PySK-**1** (1.96 mg, 12.0 µmol, 12 mol%), *(S)*-CPAK, (11.0 mg, 12.0 µmol, 12 mol%), $(Ir[dF(CF_3)ppy]_2(dtbpy))PF_6$ (5.6 mg, 5.0 µmol, 5 mol%), and a magnetic stir bar. Then, anhydrous toluene (2 mL) was added separately in both vials, and the vials were sealed with a PTFE-lined screw cap. The reaction vials were taken out of the glovebox and irradiated with a Penn M2 photoreactor (420 nm). During the reactions, aliquots were taken from the solutions simultaneously, the solvents were removed under reduced pressure and subjected to purification by small scale preparative TLC. The enantiomeric ratios were then determined by HPLC analysis with chiral stationary phase. The KIE of k_H/k_D = 1.86 was found.

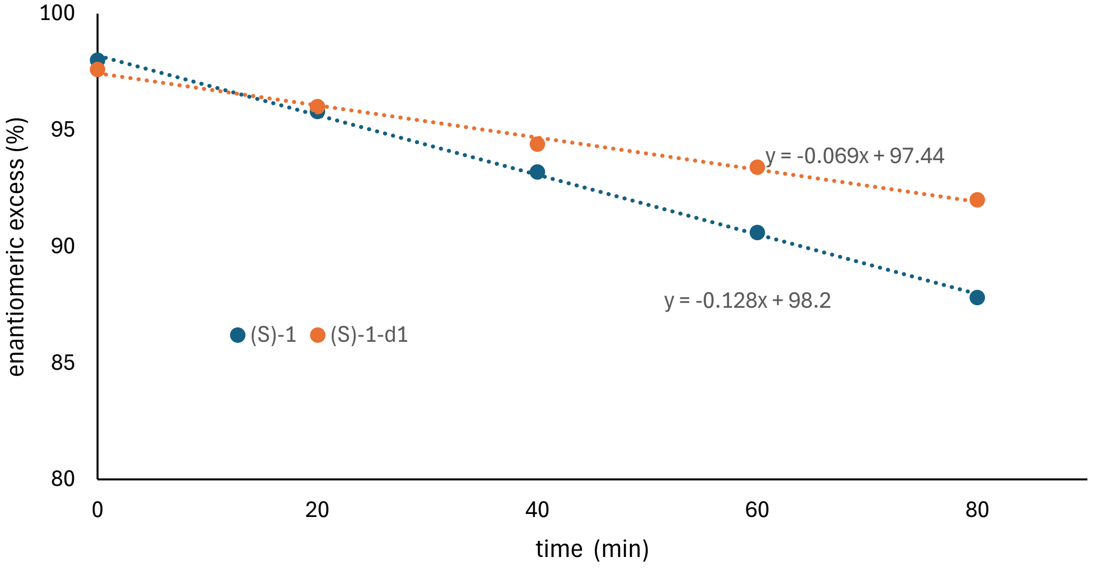

Fig. S2. Kinetic profile of the racemization of *(S)*-**1** and *(S)*-**1**-d$_1$

H/D Exchange Experiments

According to Method B, in a nitrogen-filled glovebox, three separate oven-dried 4 mL vials were charged with *rac*-**1**-*d*1, *(R)*-**1**-*d*1, and *(S)*-**1**-*d*1 (26.7 mg, 0.1 mmol, 1.0 equiv), respectively. Then, all the vials were individually charged with PySK-**1** (1.96 mg, 12.0 µmol, 12 mol%), *(S)*-CPAK, (11.0 mg, 12.0 µmol, 12 mol%), (Ir[dF(CF_3)ppy]$_2$(dtbpy))PF_6 (5.6 mg, 5.0 µmol, 5 mol%), and a magnetic stir bar. Then, anhydrous toluene (2 mL) was added separately in all the vials and the vials were sealed with a PTFE-lined screw cap. The reaction vials were taken out of the glovebox and irradiated with Penn M2 photoreactor (420 nm). After 20 h, the solution was concentrated, and the crude reaction mixture was analyzed via ^{1}H NMR spectroscopy. The enantiomeric ratios were determined by HPLC analysis on a chiral stationary phase after purification by preparative TLC.

Racemate

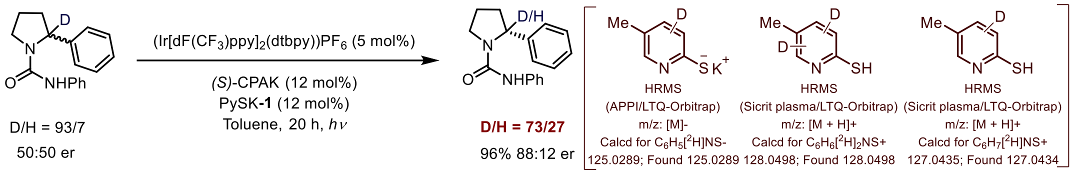

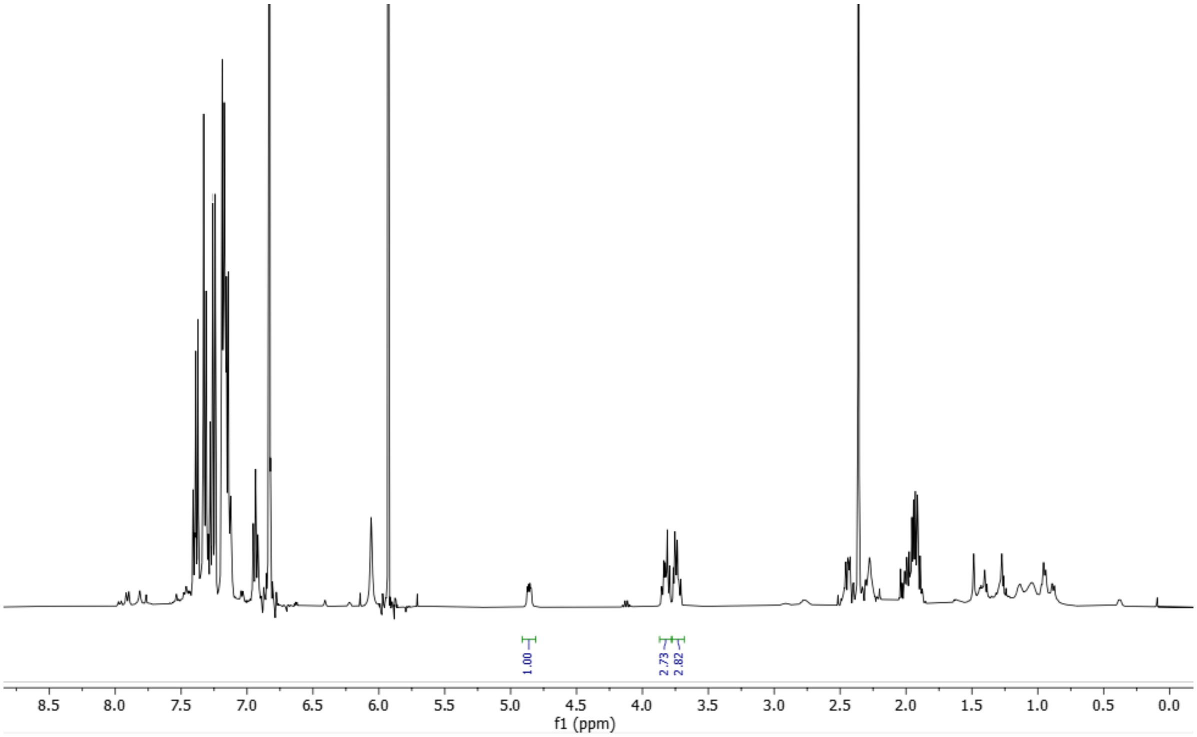

Fig. S3. Deuterium scrambling experiment of *rac*-**1**-*d*1 under standard conditions. Determination of degree of scrambling in 2-position from the crude reaction mixture by ^{1}H NMR.

Racemate with benzene as the solvent

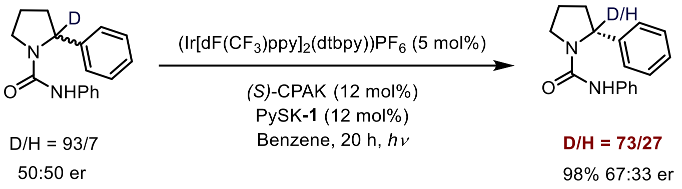

Performing the deracemization of the racemic deuterated substrate in benzene leads to the same degree of deuterium erosion observed with toluene. Thus, D/H exchange does not occur with the solvent.

Enantiopure - matched

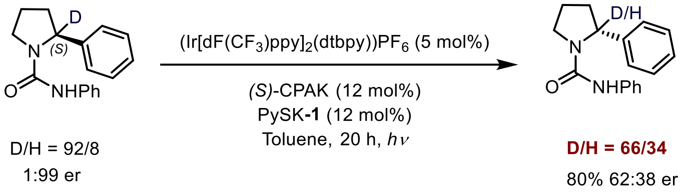

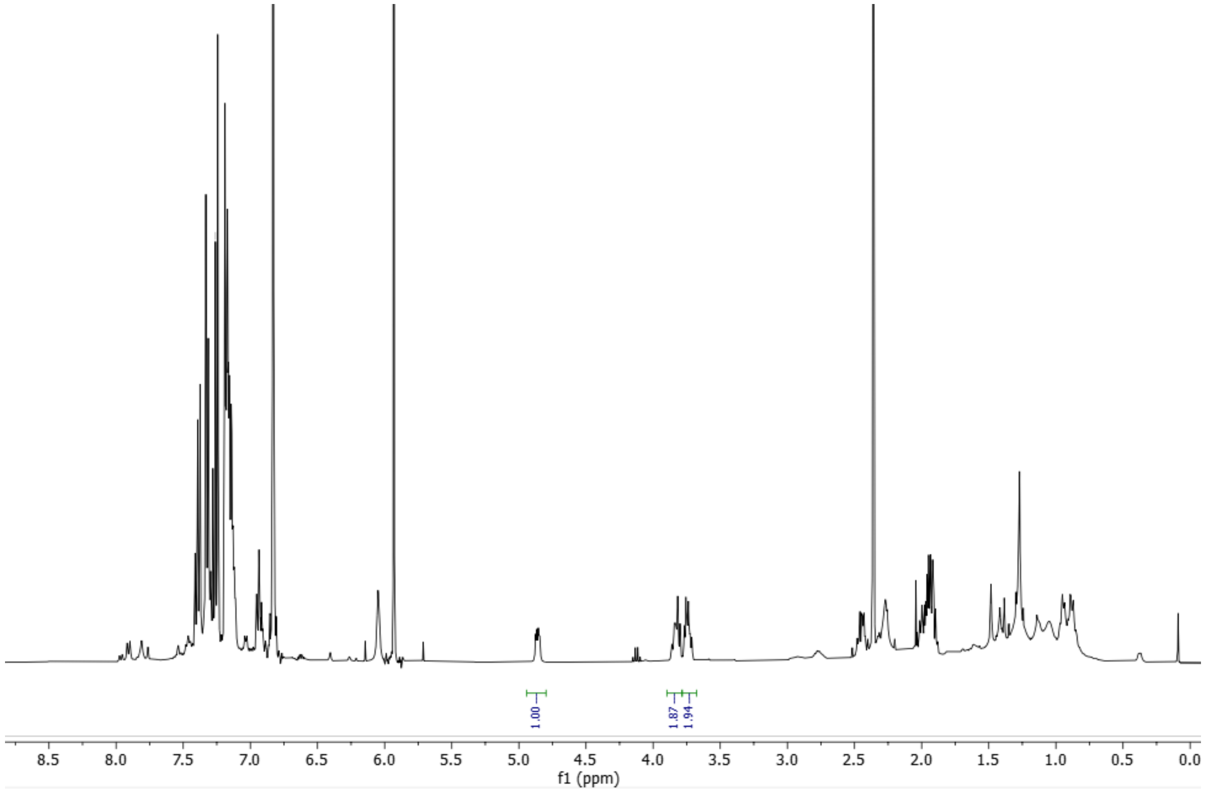

Fig. S4. Deuterium scrambling experiment of *(S)*-**1**-d_1 under standard conditions. Determination of degree of scrambling in 2-position from the crude reaction mixture by ^{1}H NMR.

Enantiopure – mismatched

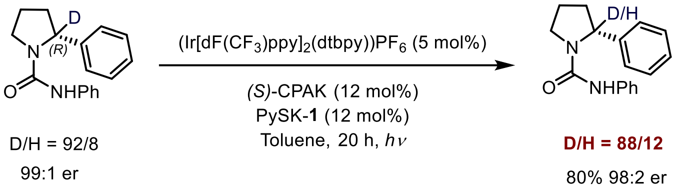

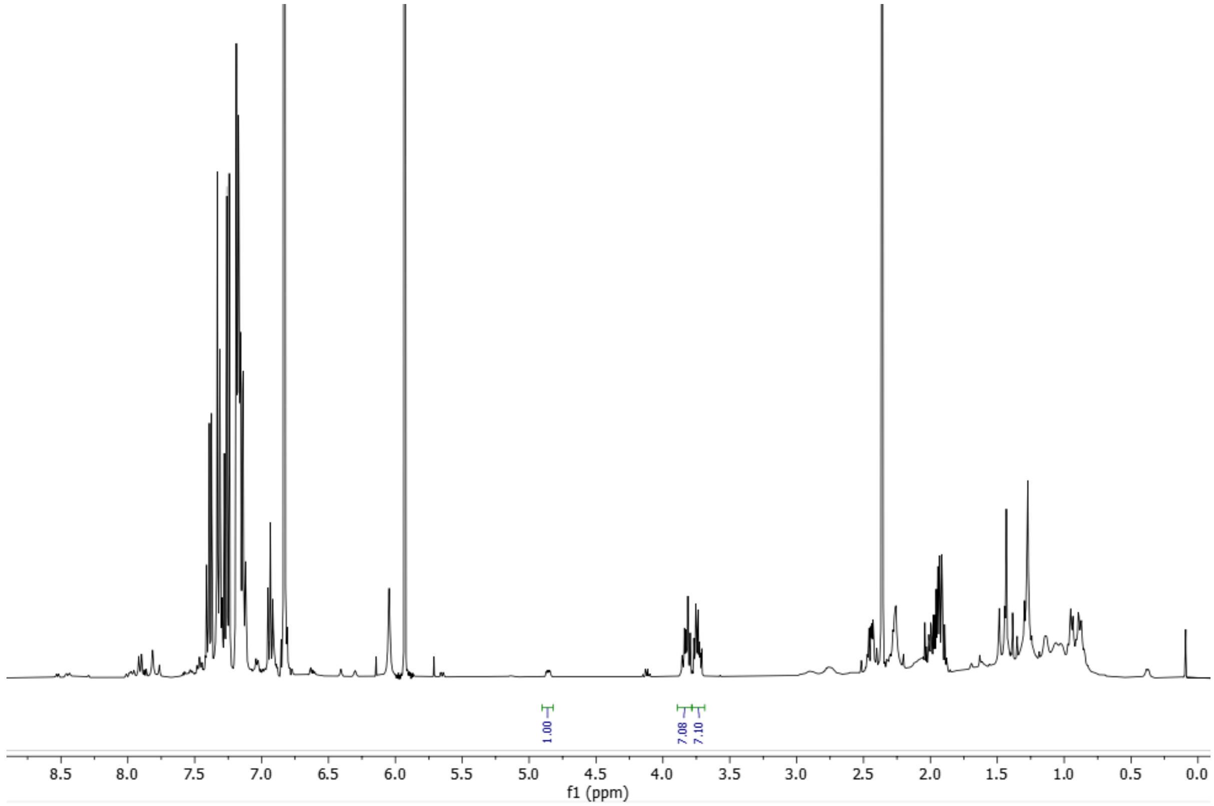

Fig. S5. Deuterium scrambling experiment of *(R)*-**1**-d_1 under standard conditions. Determination of degree of scrambling in 2-position from the crude reaction mixture by ^{1}H NMR.

H/D Crossover Experiments:

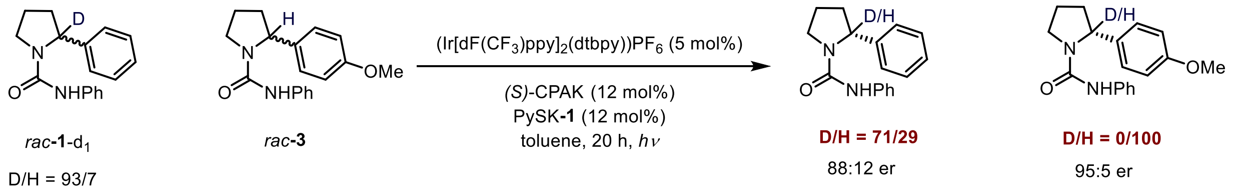

According to Method B, in a nitrogen-filled glovebox, an oven-dried 4 mL vial was charged with PySK-**1** (1.96 mg, 12.0 µmol, 12 mol%), *(S)*-CPAK, (11.0 mg, 12.0 µmol, 12 mol%), (Ir[dF(CF_3)ppy]$_2$(dtbpy))PF_6 (5.6 mg, 5.0 µmol, 5 mol%), *rac*-**1**-d_1 (13.4 mg, 0.05 mmol, 1.0 equiv), 14.8 mg of *rac*-**3**, and a magnetic stir bar. Then, anhydrous toluene (2 mL) was added, and

the vial was sealed with a PTFE-lined screw cap. The reaction was taken out of the glovebox and irradiated with Penn M2 photoreactor (420 nm) with the following settings: 100% light intensity, 6800 rpm fan cooling, 509 rpm stirring. After 20 h, the solution was concentrated and purified by flash column chromatography with 4:1:0.5 hexane/Et_2O/AcOH to 3:2 hexane/ Et_2O and then 1:1 hexane/Et_2O. The enantiomeric ratios were then determined by HPLC analysis on a chiral stationary phase.

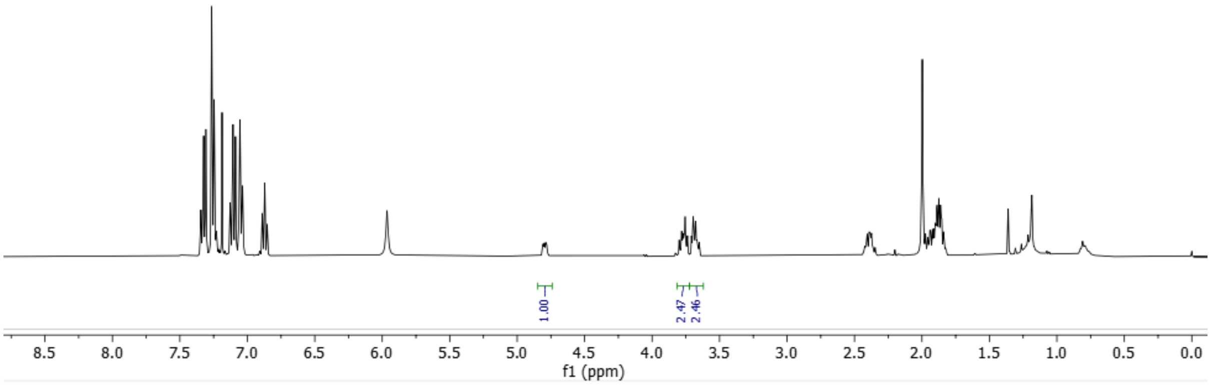

Fig. S6. Competition experiment of *rac*-**1**-d_1 under standard conditions. The degree of deuterium scrambling was determined after column chromatography by ^{1}H NMR

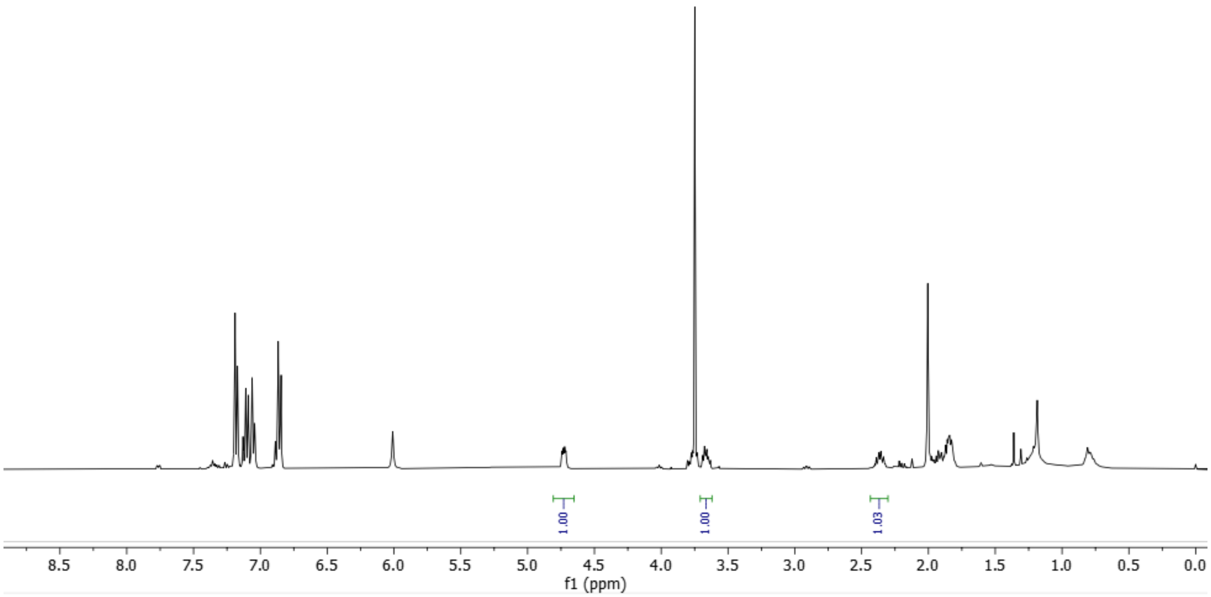

Fig. S7. Competition experiment of *rac*-**3** under standard conditions. The degree of deuterium scrambling was determined after column chromatography by ^{1}H NMR.

Experiments with Excess PySH-6

The control experiments with excess PySH-**6** have been conducted according to Method B.

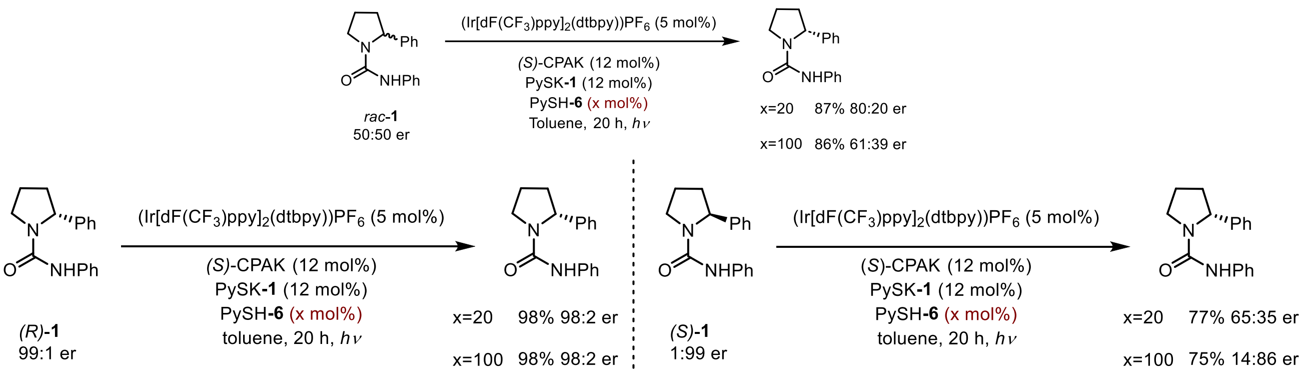

Experiments with Excess Thiophenol

The control experiments with excess thiophenol have been conducted according to Method B.

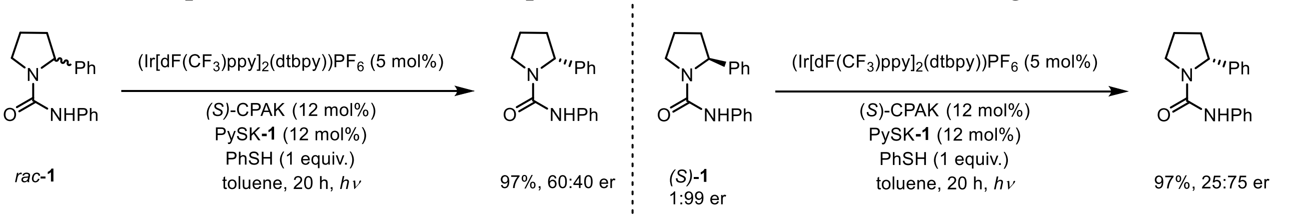

Experiments with Thiophenol instead of PySH-6

The control experiments replacing PySH-**6** with thiophenol have been conducted according to Method B.

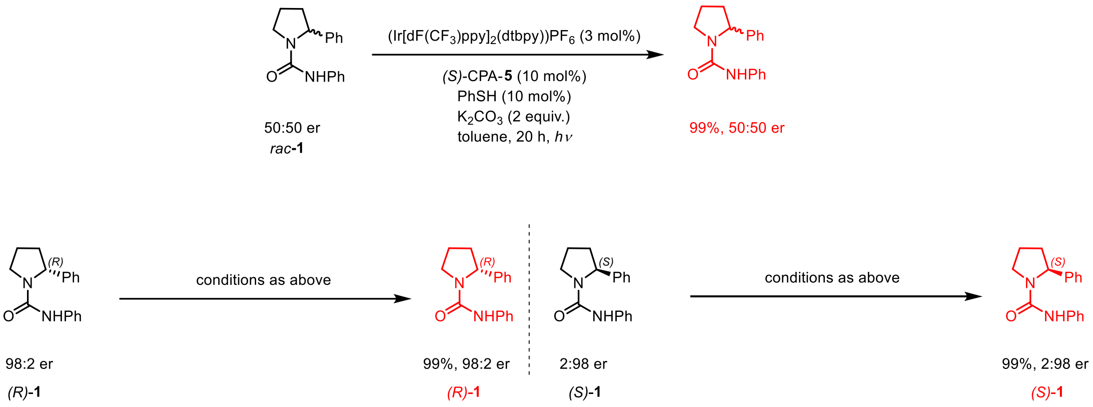

References

1. Sasaki, S. *et al.* Synthesis of crowded triarylphosphines carrying functional sites. *J. Organomet. Chem.* **690**, 2664–2672 (2005).
2. Li, J., Grosslight, S., Miller, S. J., Sigman, M. S. & Toste, F. D. Site-selective acylation of natural products with BINOL-derived phosphoric acids. *ACS Catal.* **9**, 9794–9799 (2019).
3. Chen, W., Ma, L., Paul, A. & Seidel, D. Direct α-C–H bond functionalization of unprotected cyclic amines. *Nat. Chem.* **10**, 165–169 (2018).
4. Matteson, D. S. & Kim, G. Y. Asymmetric Alkyldifluoroboranes and Their Use in Secondary Amine Synthesis. *Org. Lett.* **4**, 2153–2155 (2002).
5. Coindet, C., Comel, A. & Kirsch, G. Easy one-pot access to substituted 2-phenylpyrrolines from 2-pyrrolidinone. *Tetrahedron Lett.* **42**, 6101–6104 (2001).
6. Escoubet, S. *et al.* Thiyl Radical Mediated Racemization of Nonactivated Aliphatic Amines. *J. Org. Chem.* **71**, 7288–7292 (2006).
7. Renny, J. S., Tomasevich, L. L., Tallmadge, E. H. & Collum, D. B. Method of continuous variations: applications of job plots to the study of molecular associations in organometallic chemistry. *Angew. Chem. Int. Ed Engl.* **52**, 11998–12013 (2013).
8. Du, R. *et al.* Resonance Raman spectroscopic and theoretical investigation of the excited state proton transfer reaction dynamics of 2-thiopyridone. *J. Phys. Chem. B* **115**, 8266–8277 (2011).

NMR Spectra

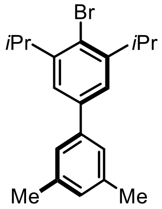

S1
(^{1}H NMR, 400 MHz, $CDCl_3$)

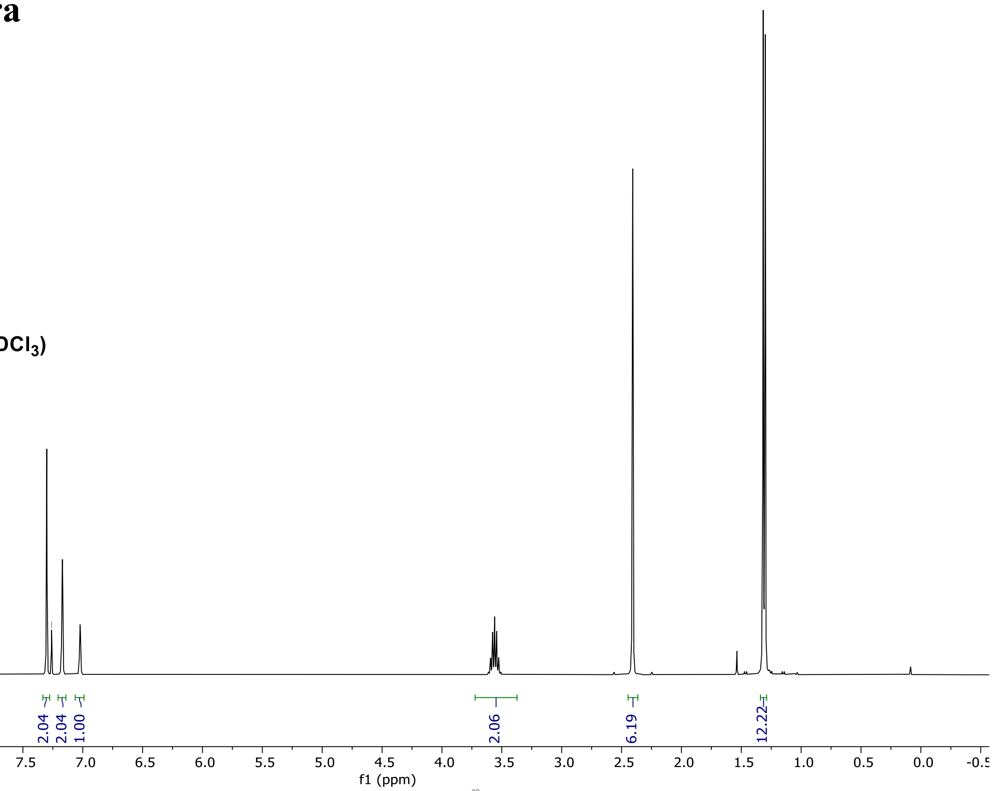

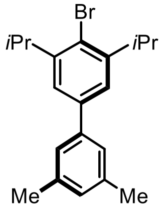

S1
(^{13}C NMR, 101 MHz, $CDCl_3$)

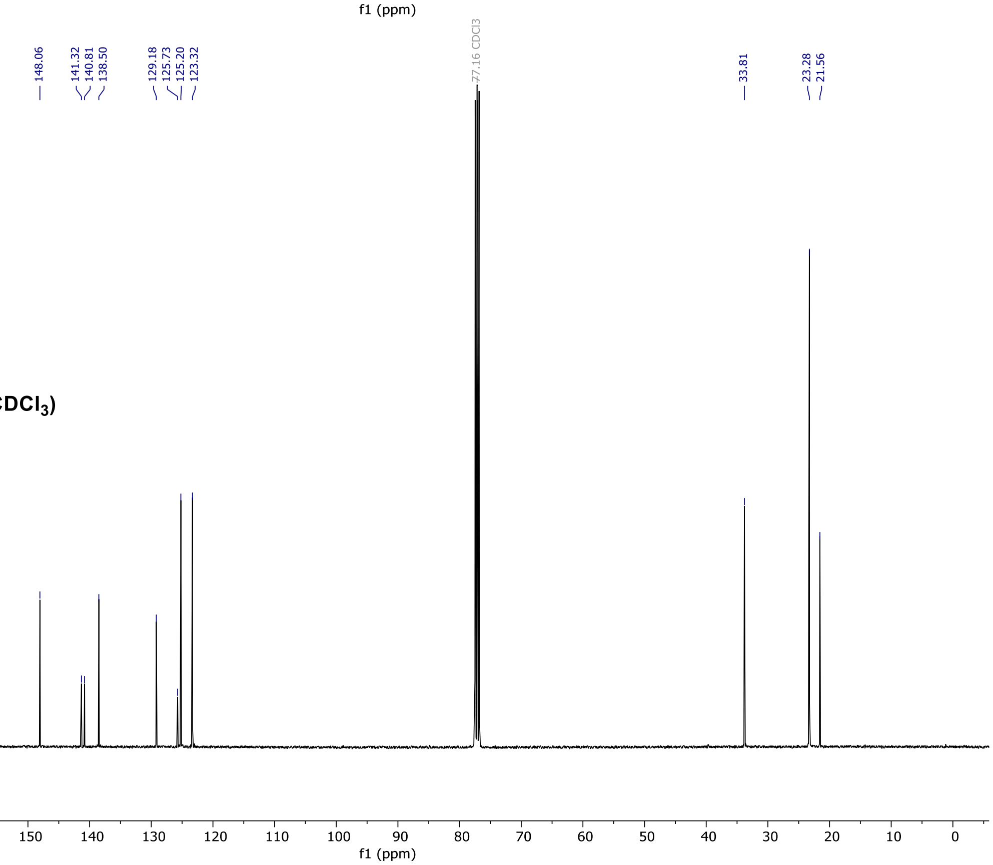

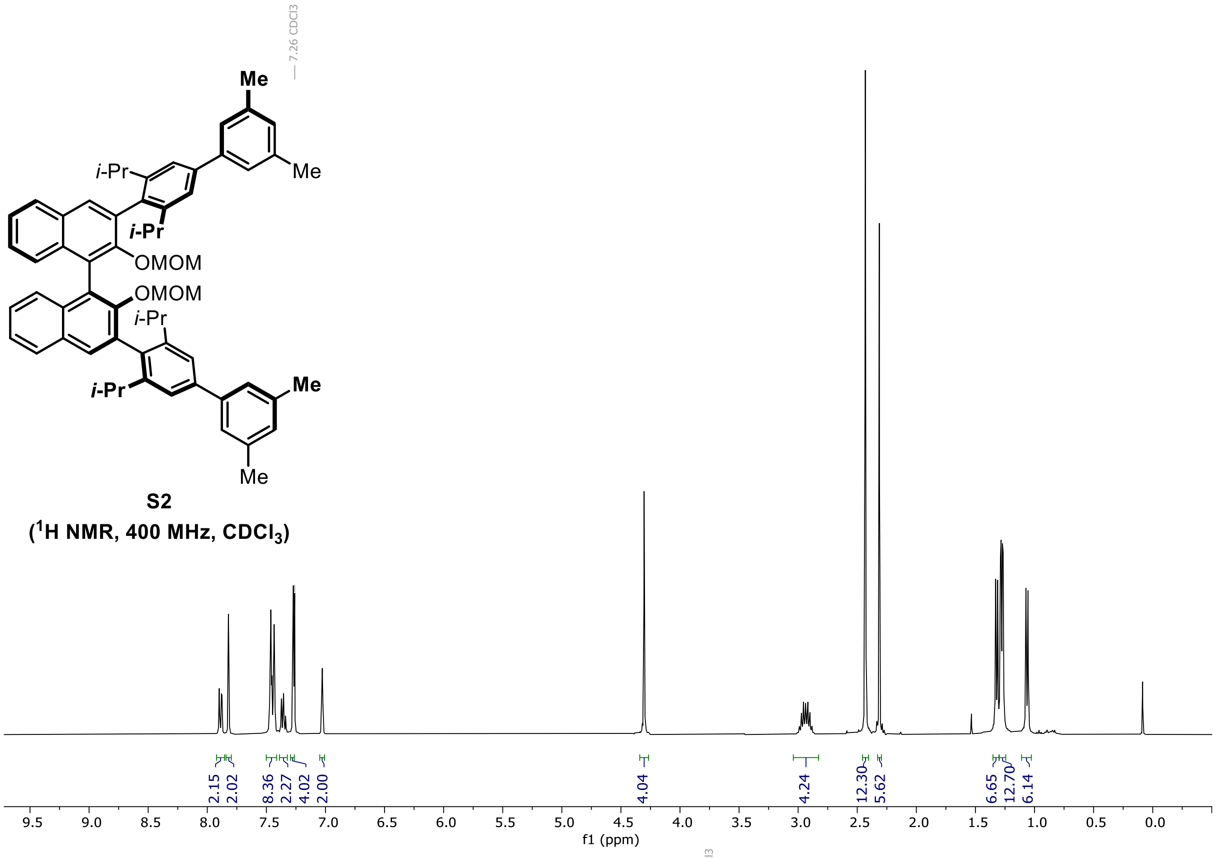
7.26 CDCl3
Me
i-Pr
Me
i-Pr
OMOM
OMOM
i-Pr
i-Pr
Me
Me
S2
(1H NMR, 400 MHz, CDCl3)
2.15
2.02
8.36
2.27
4.02
2.00
4.04
4.24
12.30
5.62
6.65
12.70
6.14
9.5 9.0 8.5 8.0 7.5 7.0 6.5 6.0 5.5 5.0 4.5 4.0 3.5 3.0 2.5 2.0 1.5 1.0 0.5 0.0
f1 (ppm)

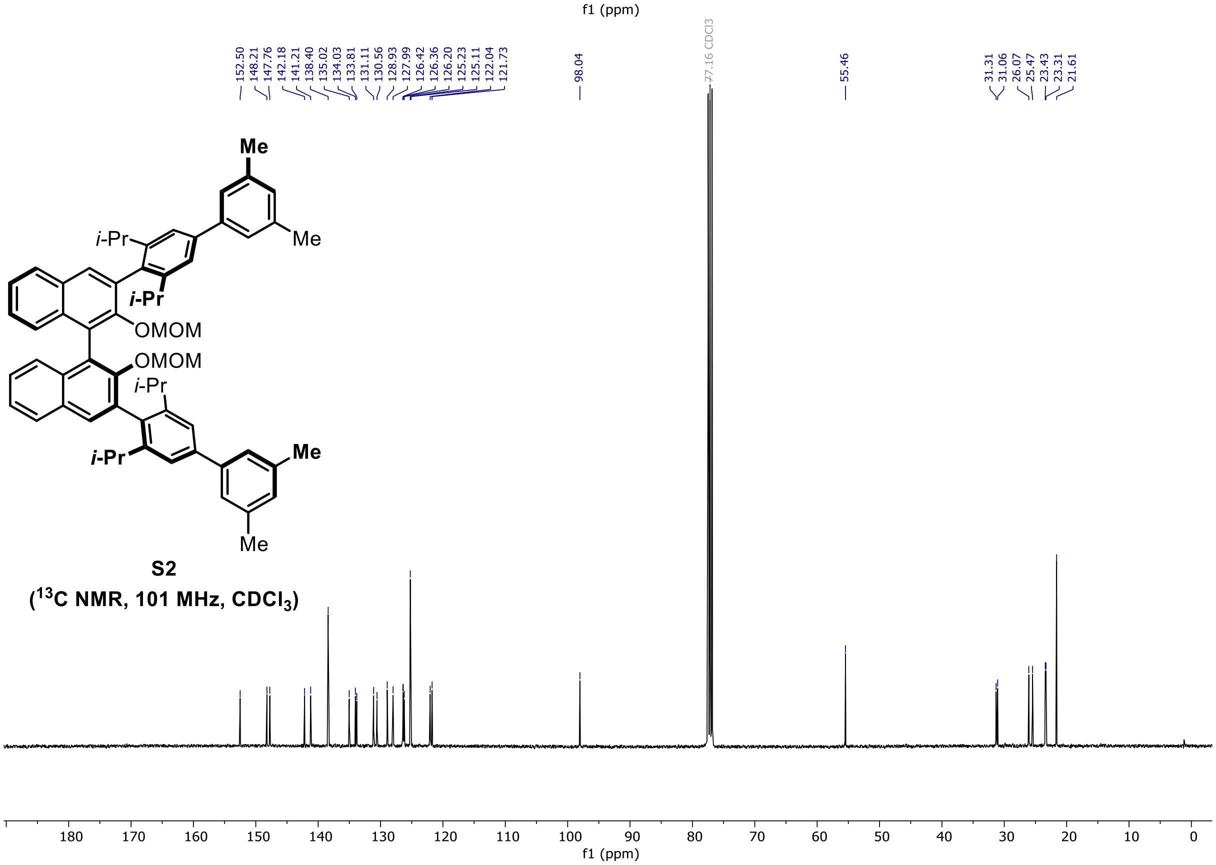
152.50
148.21
147.76
142.18
141.21
138.40
135.02
134.03
133.81
131.11
130.56
128.93
127.99
126.42
126.36
126.20
125.23
125.11
122.04
121.73
98.04
77.16 CDCl3
55.46
31.31
31.06
26.07
25.47
23.43
23.31
21.61
Me
i-Pr
Me
i-Pr
OMOM
OMOM
i-Pr
i-Pr
Me
Me
S2
(13C NMR, 101 MHz, CDCl3)
180 170 160 150 140 130 120 110 100 90 80 70 60 50 40 30 20 10 0
f1 (ppm)

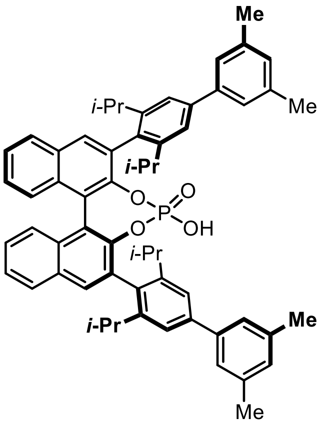

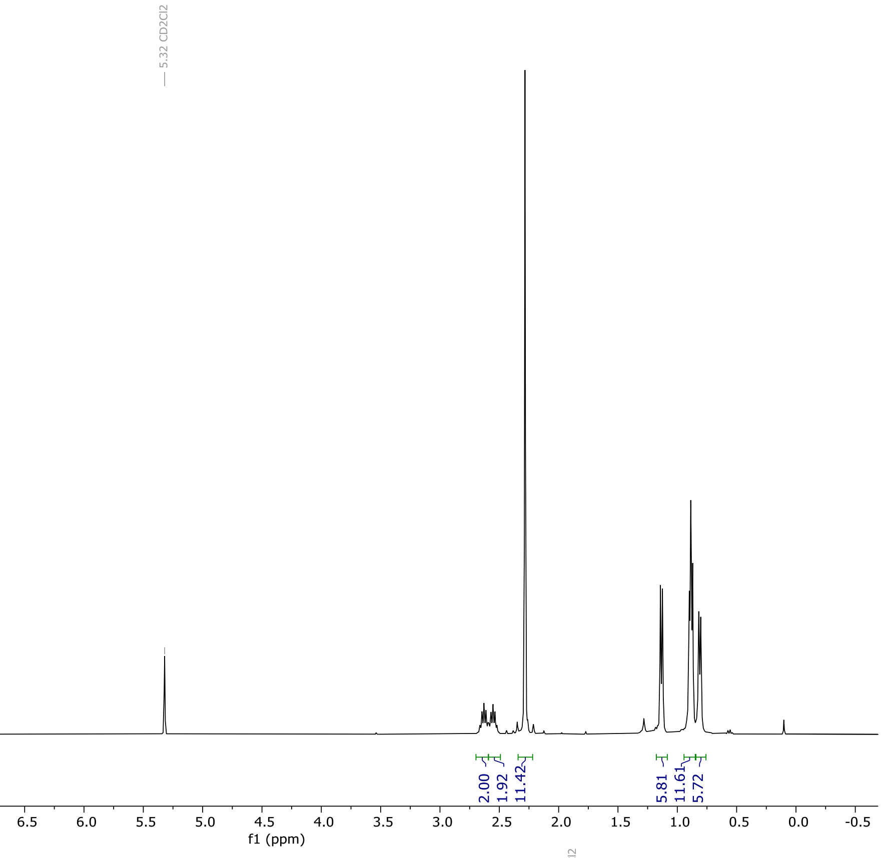

(*S*)-CPA-**5**
(^{1}H NMR, 400 MHz, CD_2Cl_2)

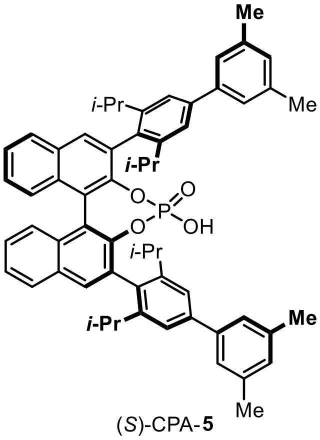

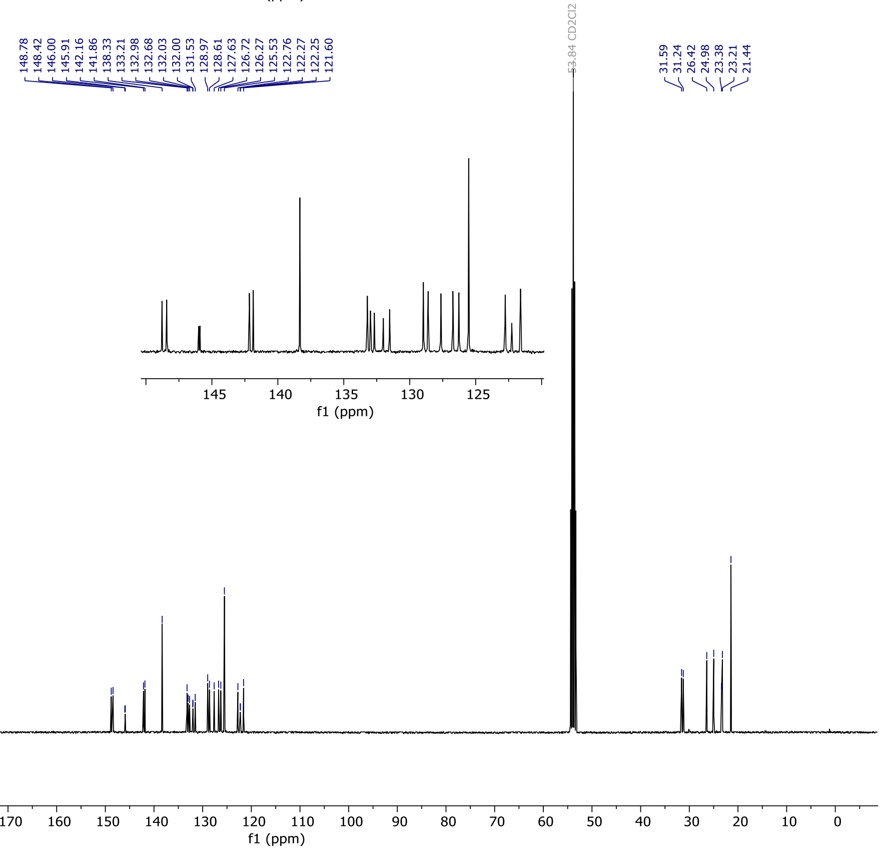

(*S*)-CPA-**5**
(^{13}C NMR, 101 MHz, CD_2Cl_2)

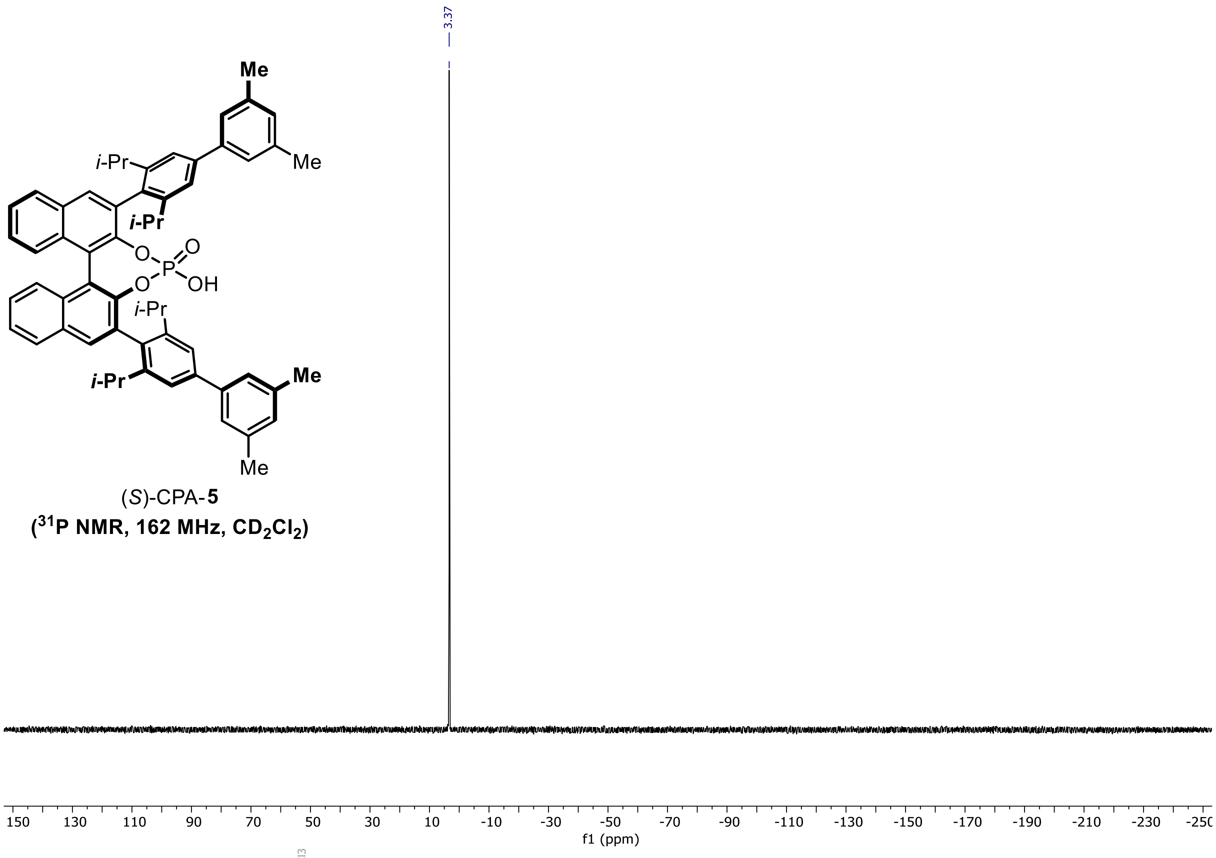

(*S*)-CPA-**5**
(**^{31}P NMR, 162 MHz, CD_2Cl_2**)

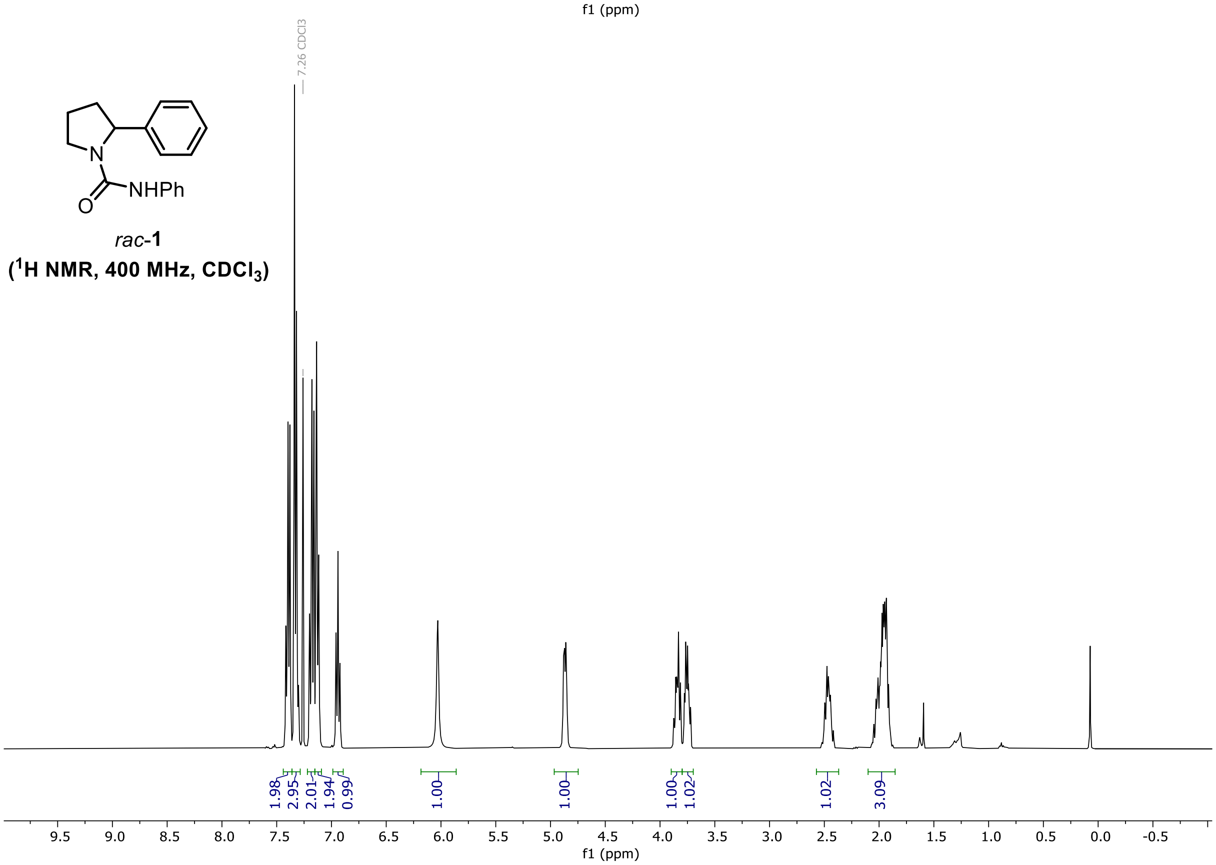

rac-**1**
(**^{1}H NMR, 400 MHz, $CDCl_3$**)

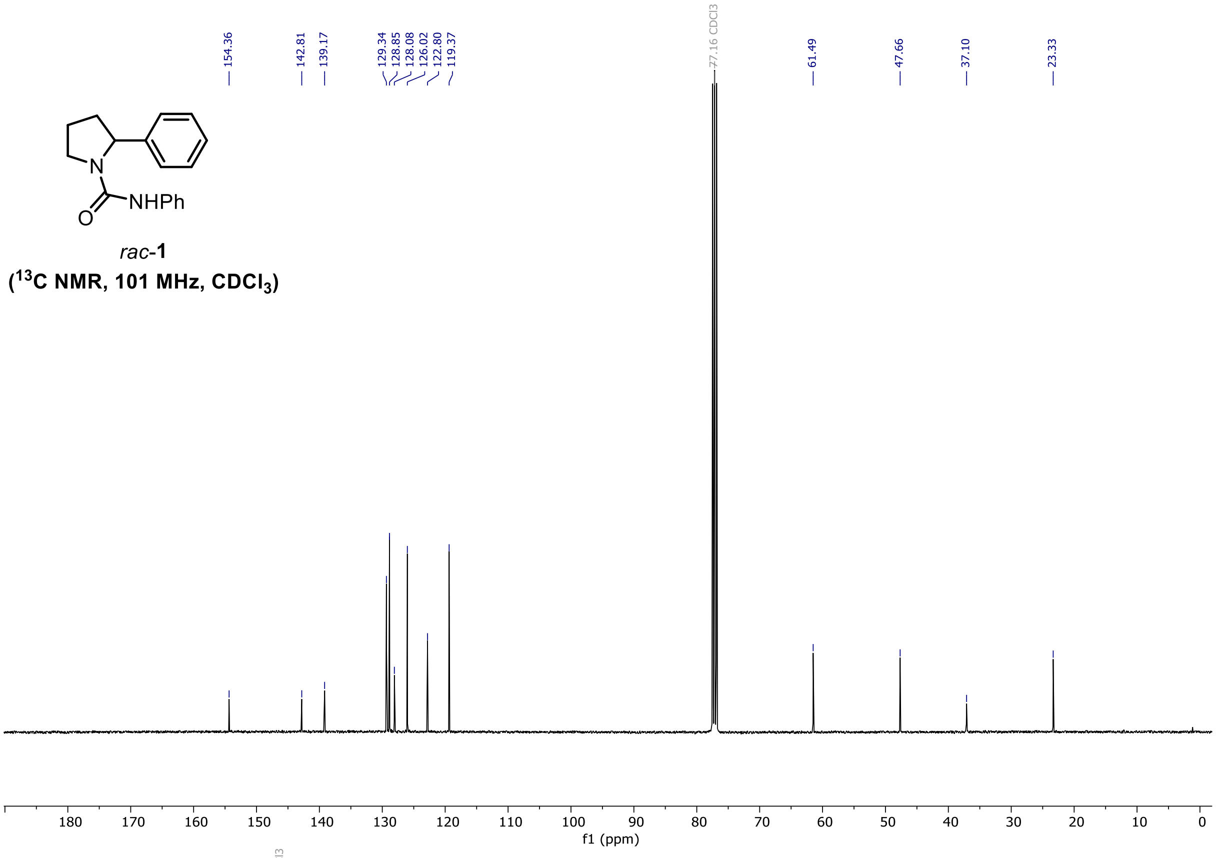
154.36
142.81
139.17
129.34
128.85
128.08
126.02
122.80
119.37
77.16 CDCl3
61.49
47.66
37.10
23.33
NHPh
N
O
rac-1
(13C NMR, 101 MHz, CDCl3)
f1 (ppm)

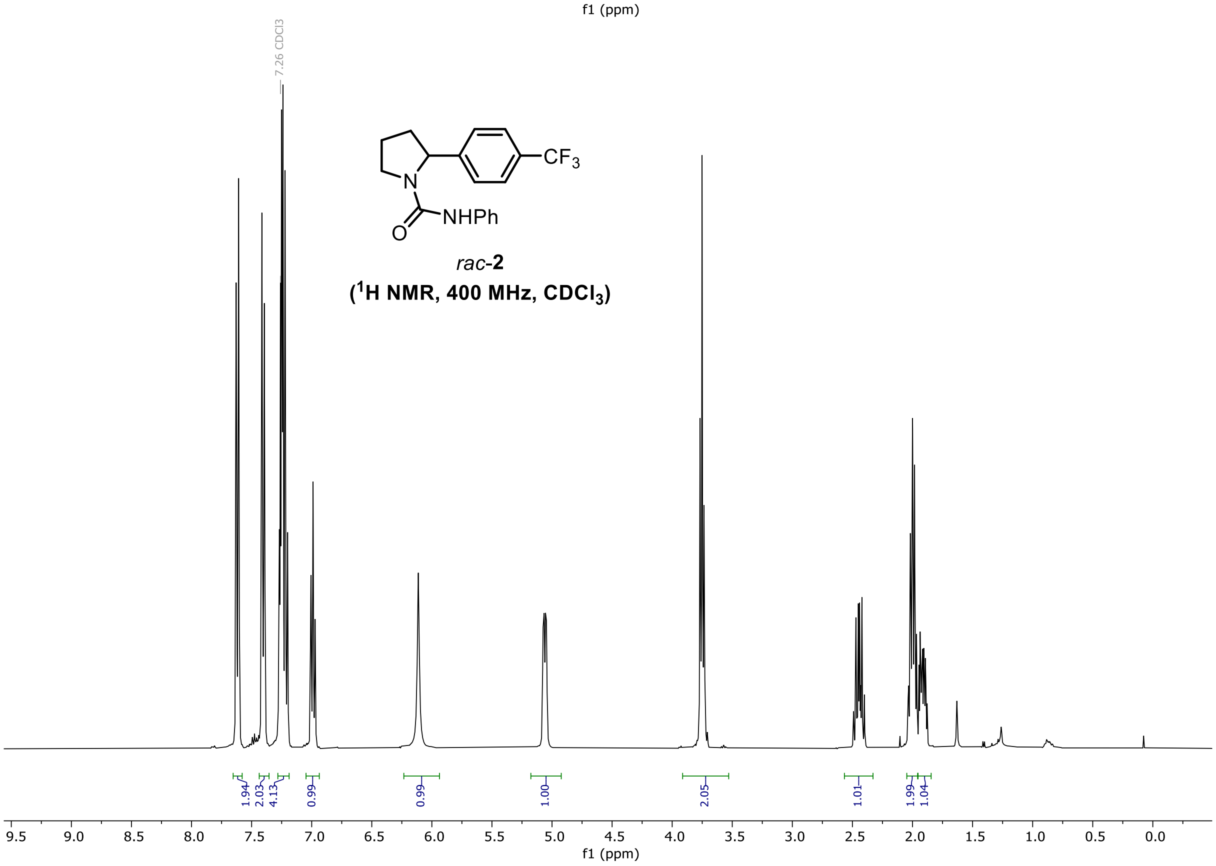
7.26 CDCl3
CF3
N
NHPh
O
rac-2
(1H NMR, 400 MHz, CDCl3)
1.94
2.03
4.13
0.99
0.99
1.00
2.05
1.01
1.99
1.04
f1 (ppm)

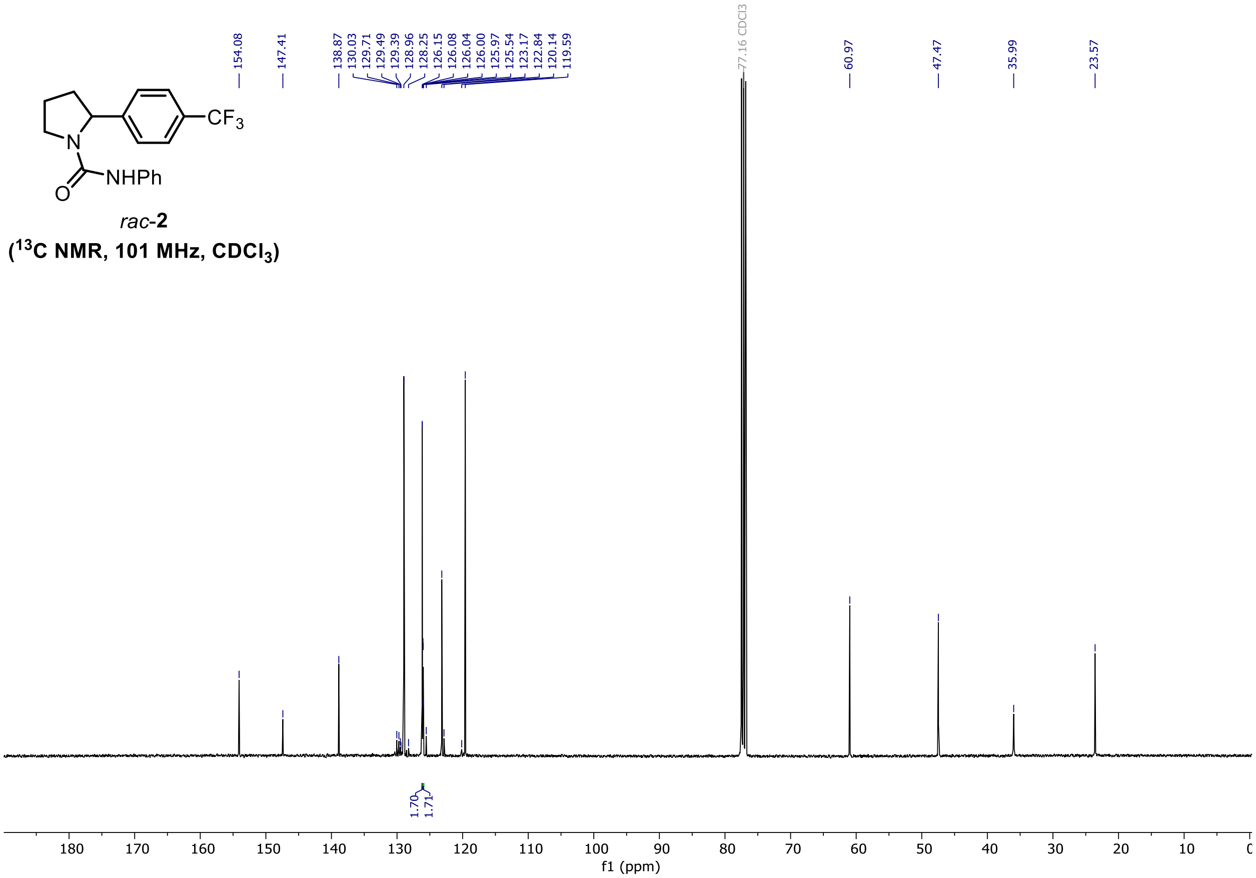

rac-**2**
(^{13}C NMR, 101 MHz, $CDCl_3$)

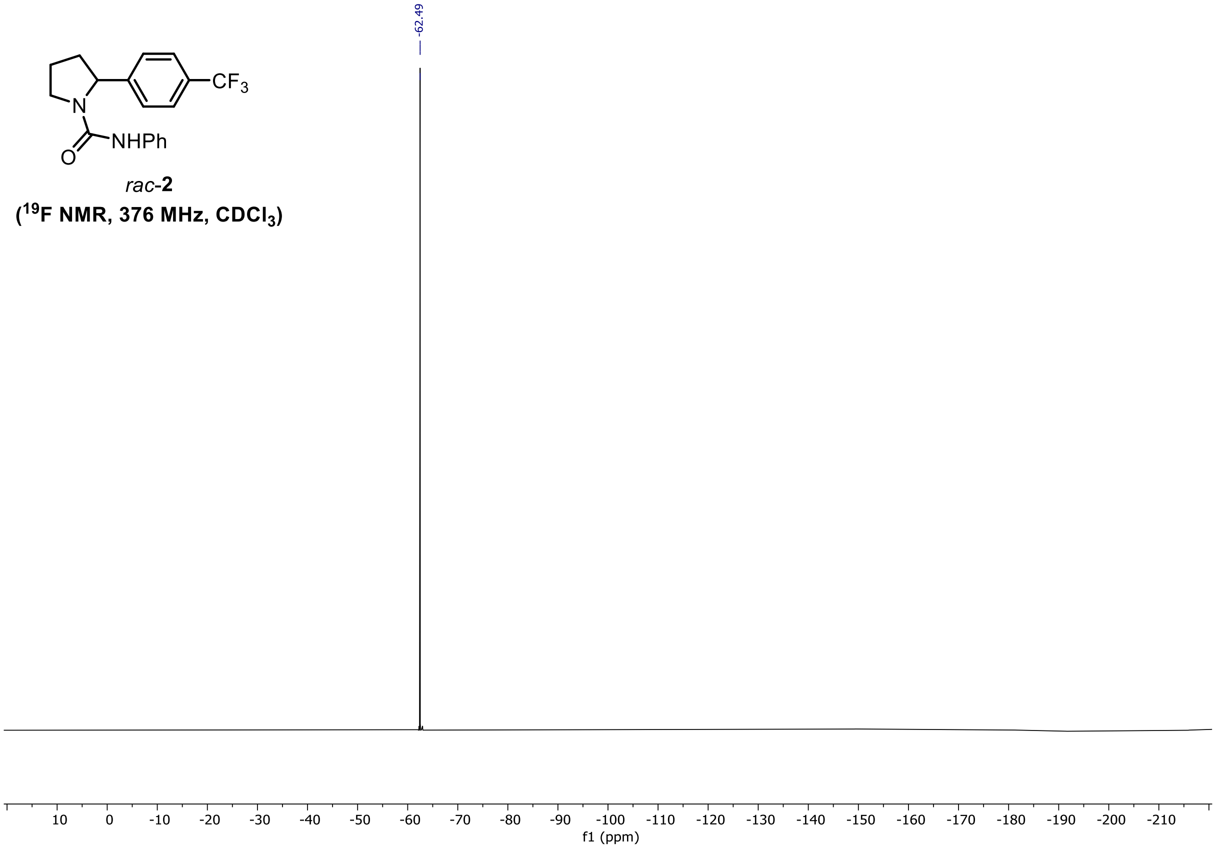

rac-**2**
(^{19}F NMR, 376 MHz, $CDCl_3$)

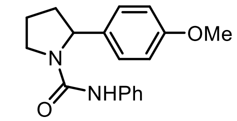

rac-**3**

(^{1}H NMR, 400 MHz, CDCl$_3$)

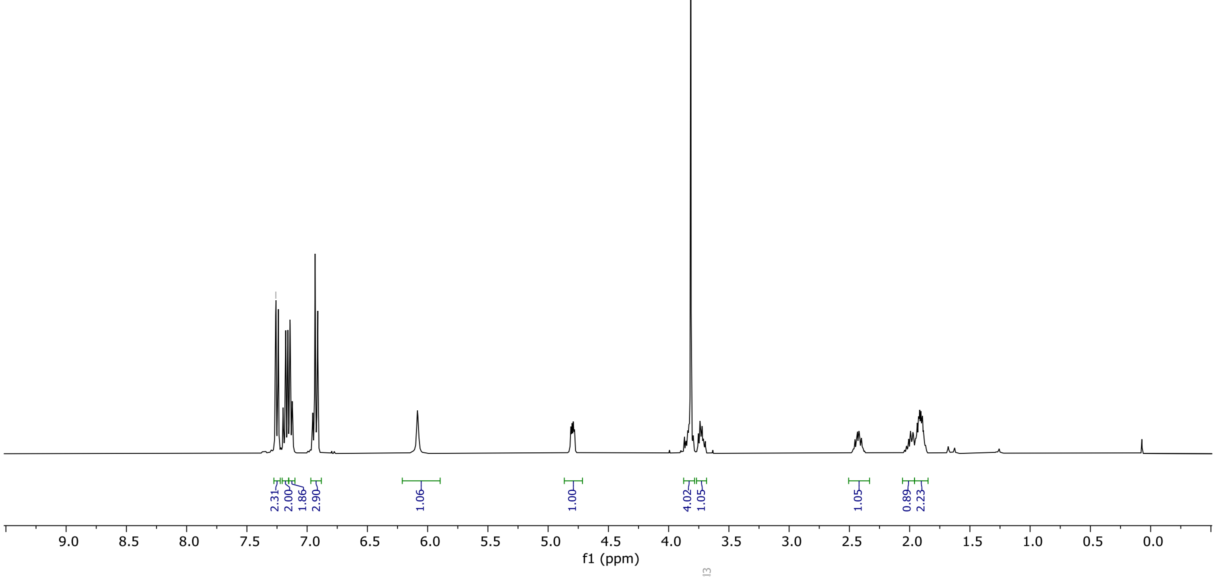

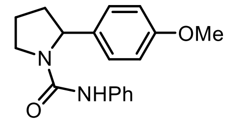

rac-**3**

(^{13}C NMR, 101 MHz, CDCl$_3$)

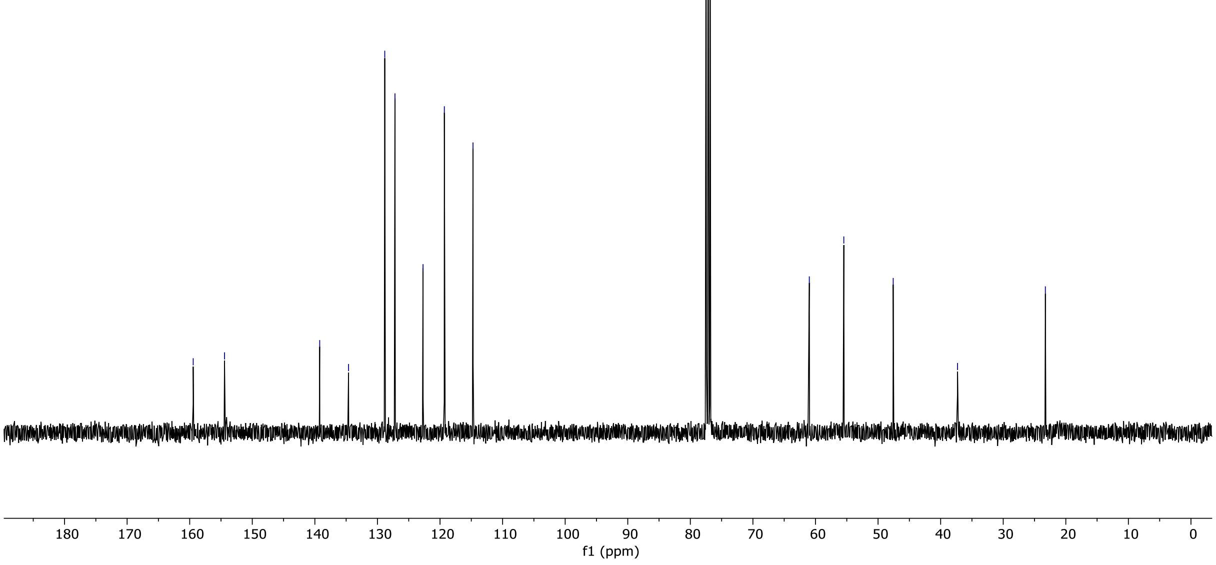

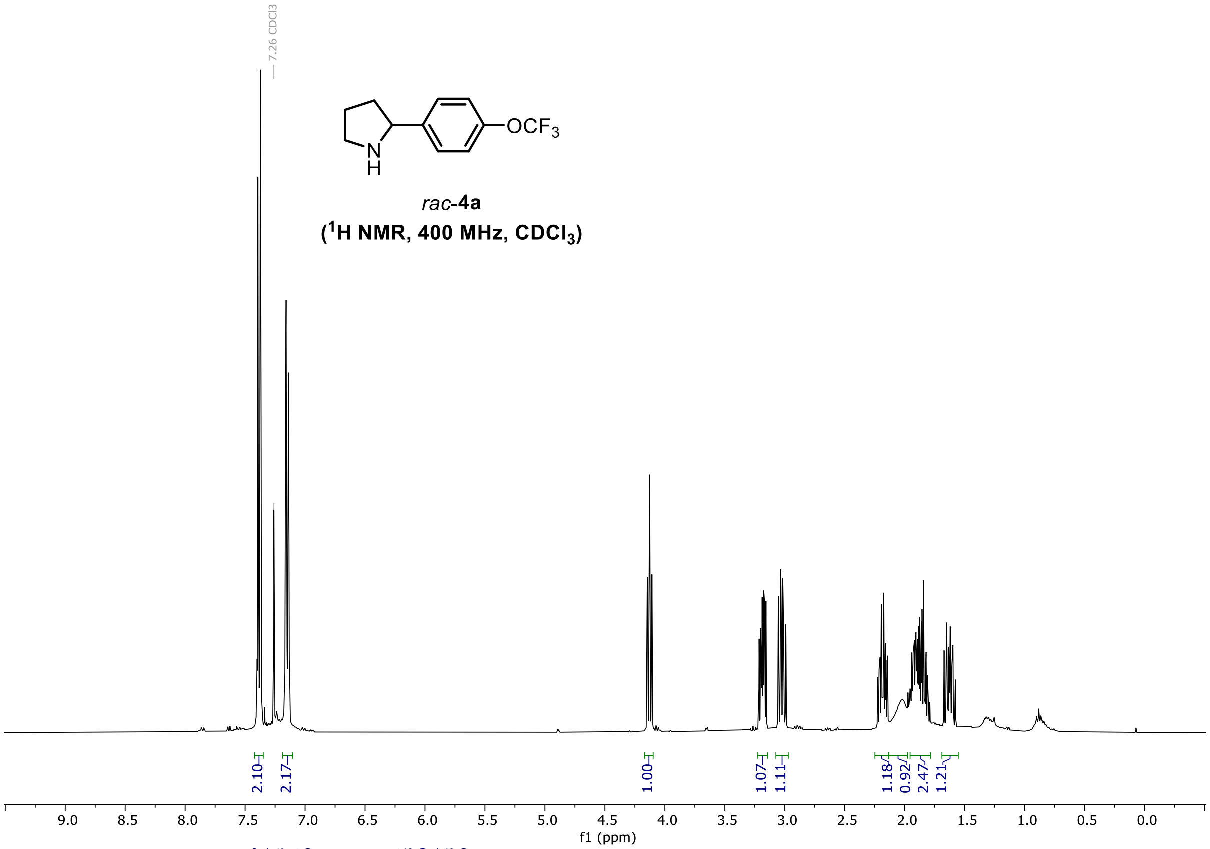

rac-**4a**
(^{1}H NMR, 400 MHz, $CDCl_3$)

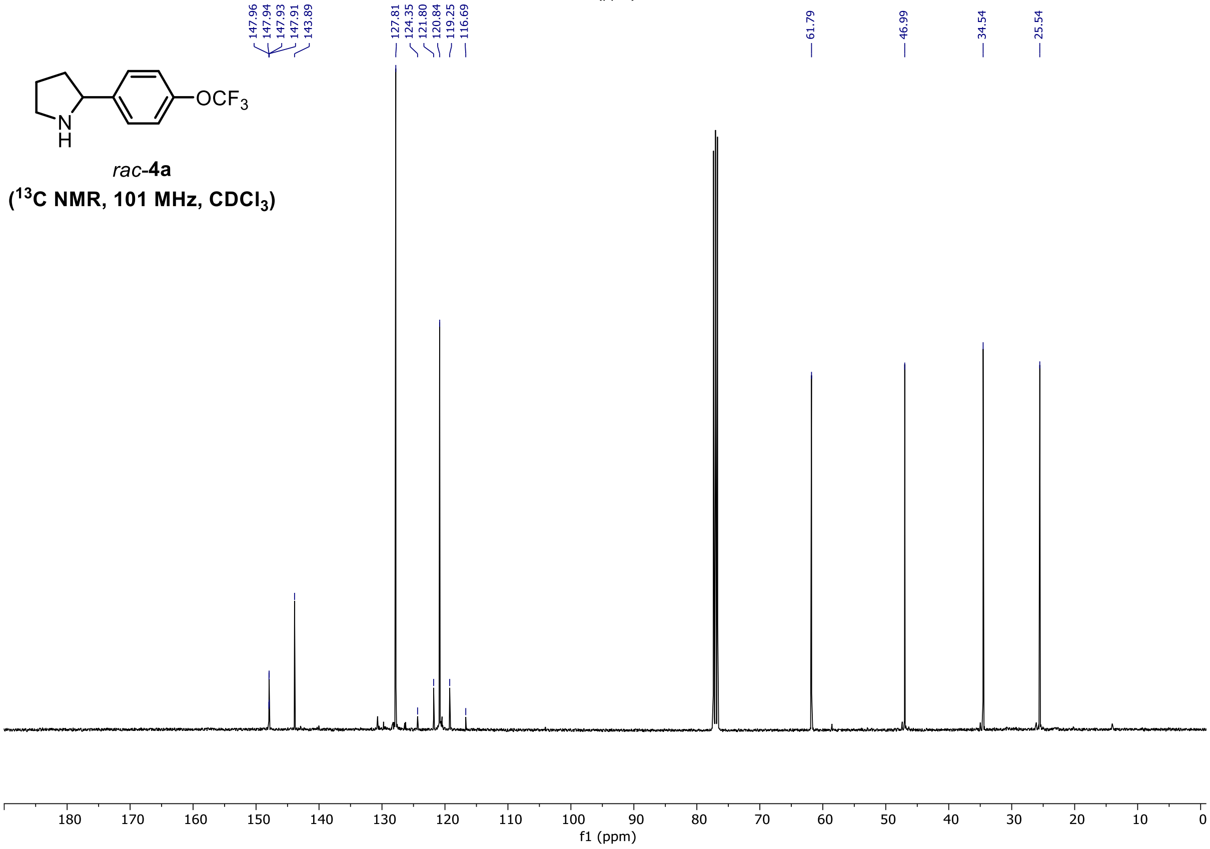

rac-**4a**
(^{13}C NMR, 101 MHz, $CDCl_3$)

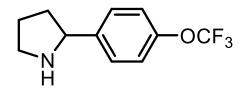

rac-**4a**
(^{19}F NMR, 376 MHz, CDCl$_3$)

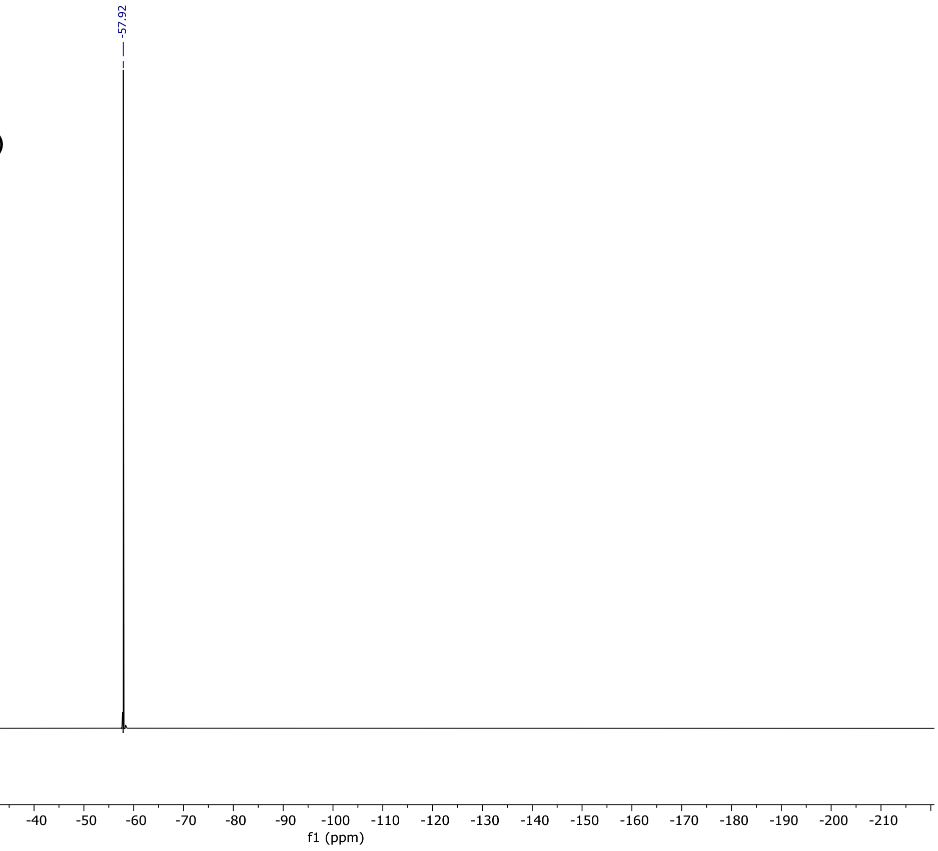

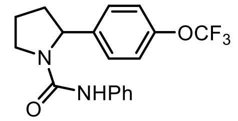

rac-**4**
(^{1}H NMR, 400 MHz, CDCl$_3$)

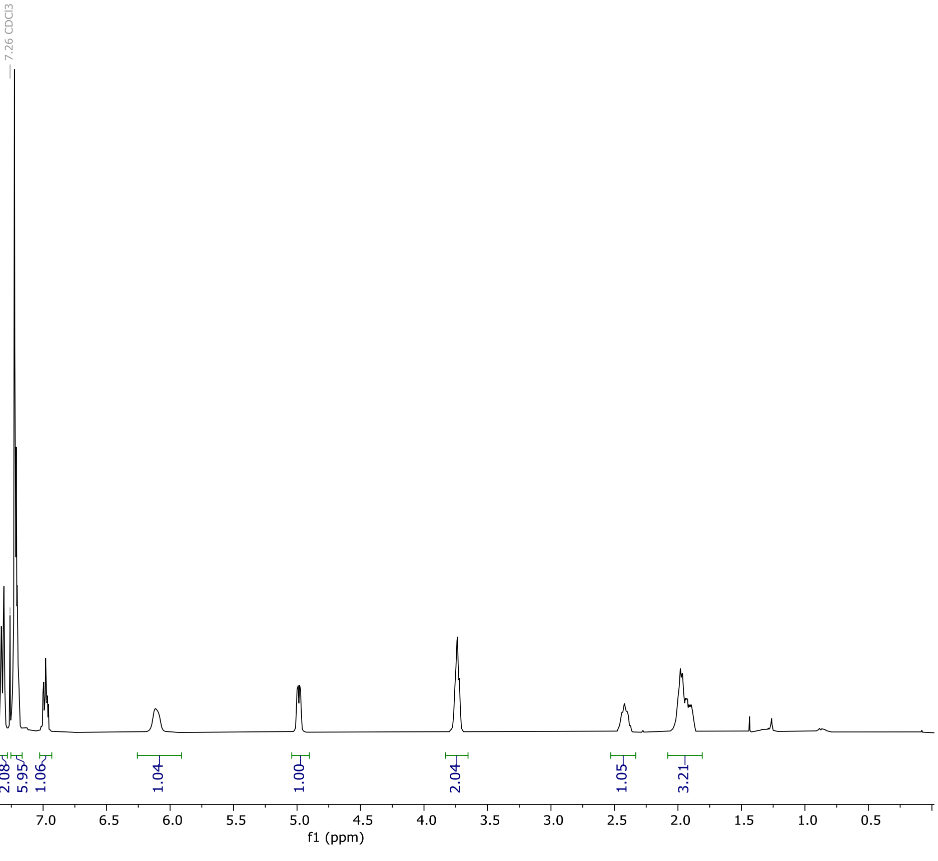

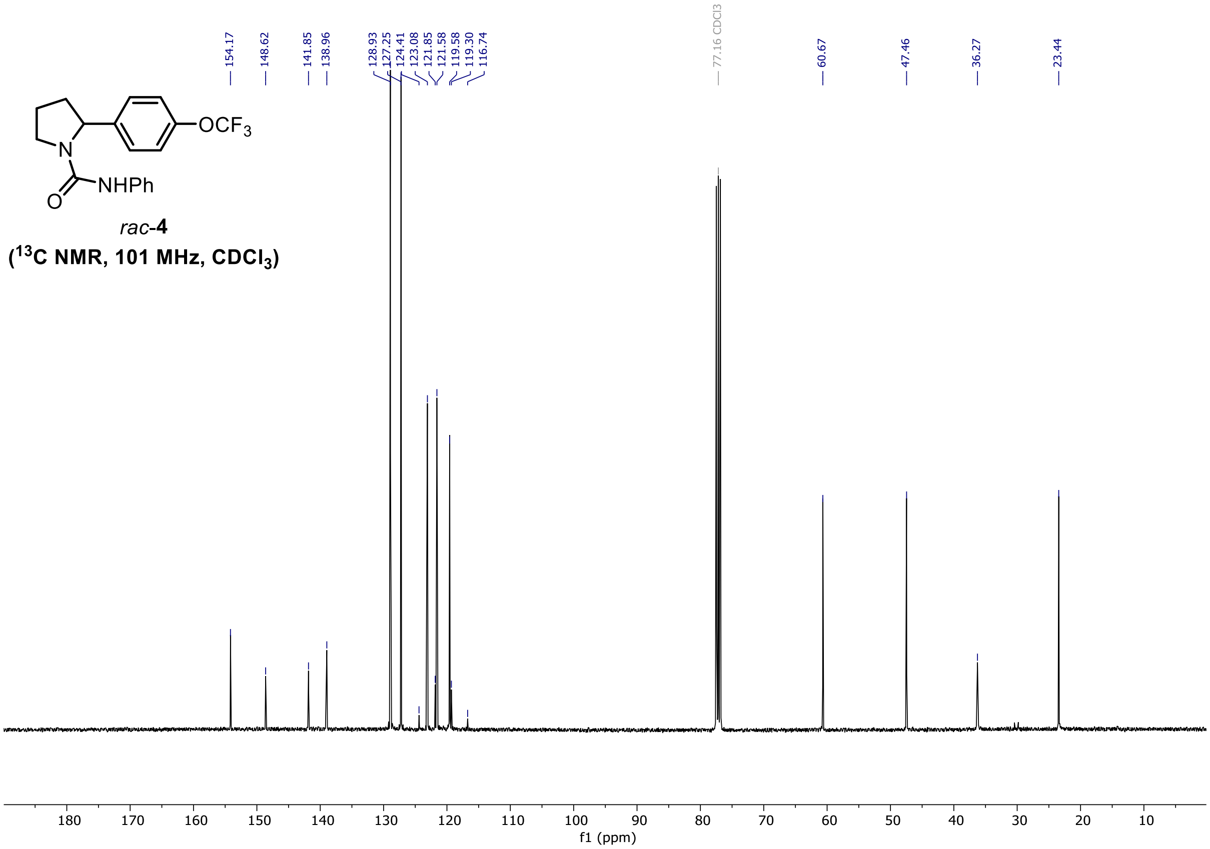

rac-**4**

(^{13}C NMR, 101 MHz, $CDCl_3$)

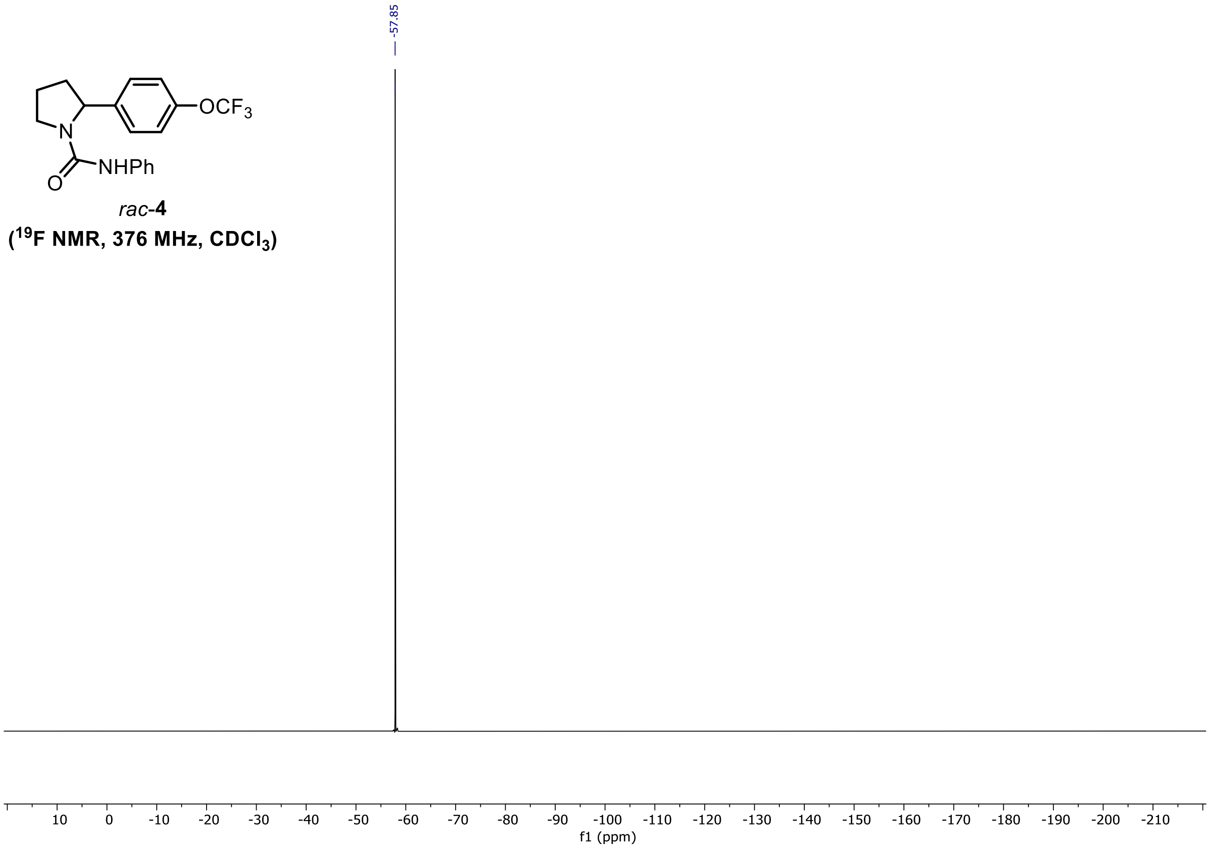

rac-**4**

(^{19}F NMR, 376 MHz, $CDCl_3$)

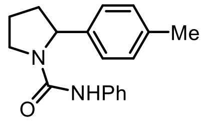

rac-**5**
(^{1}H NMR, 400 MHz, CDCl$_3$)

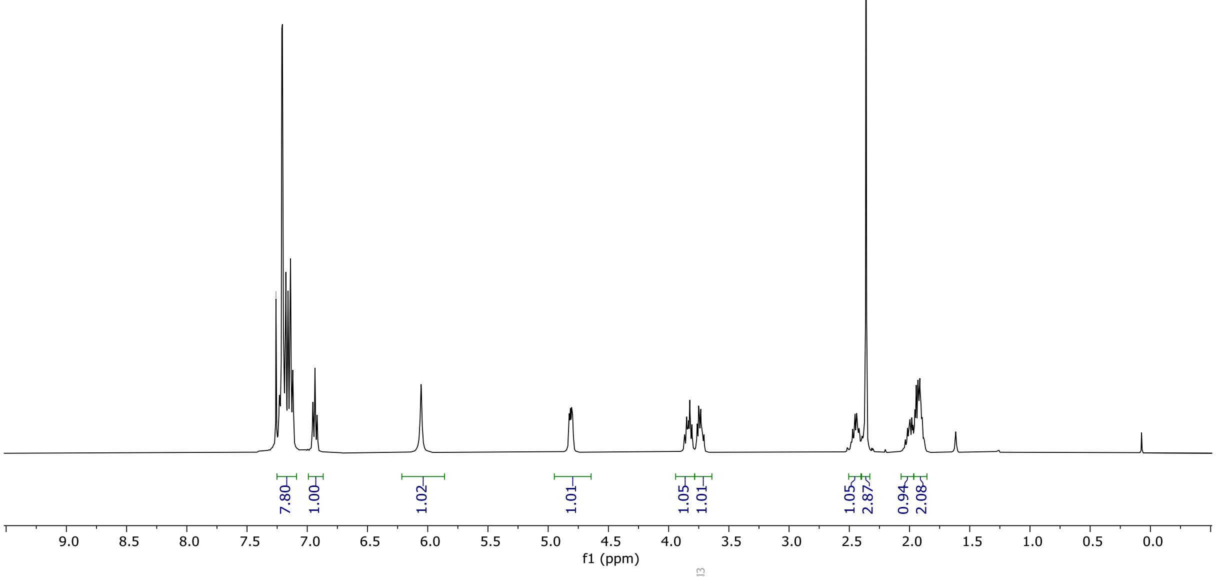

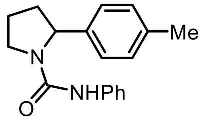

rac-**5**
(^{13}C NMR, 101 MHz, CDCl$_3$)

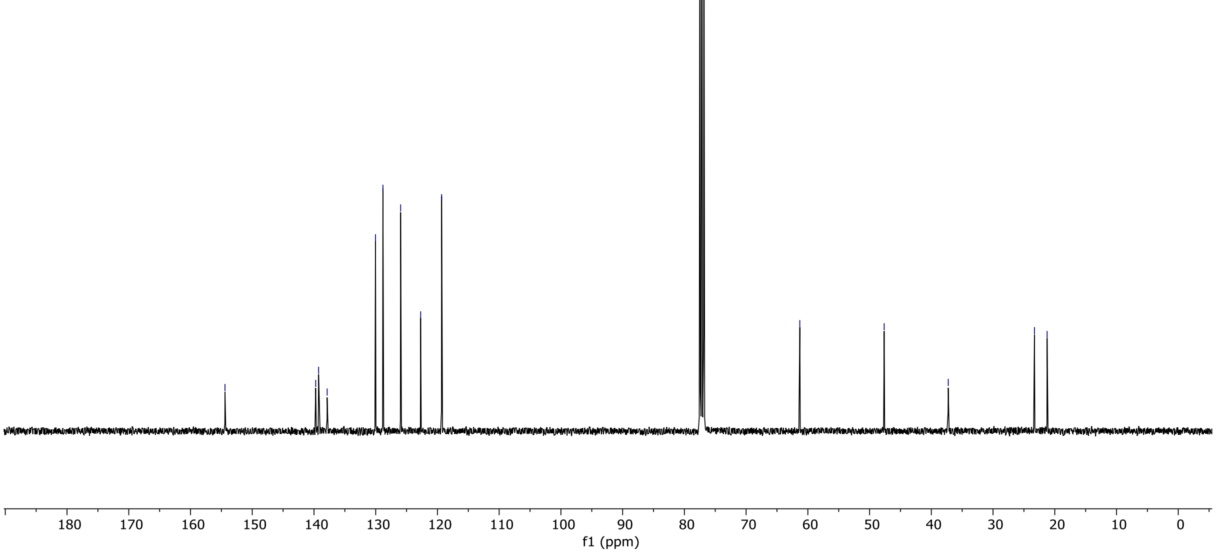

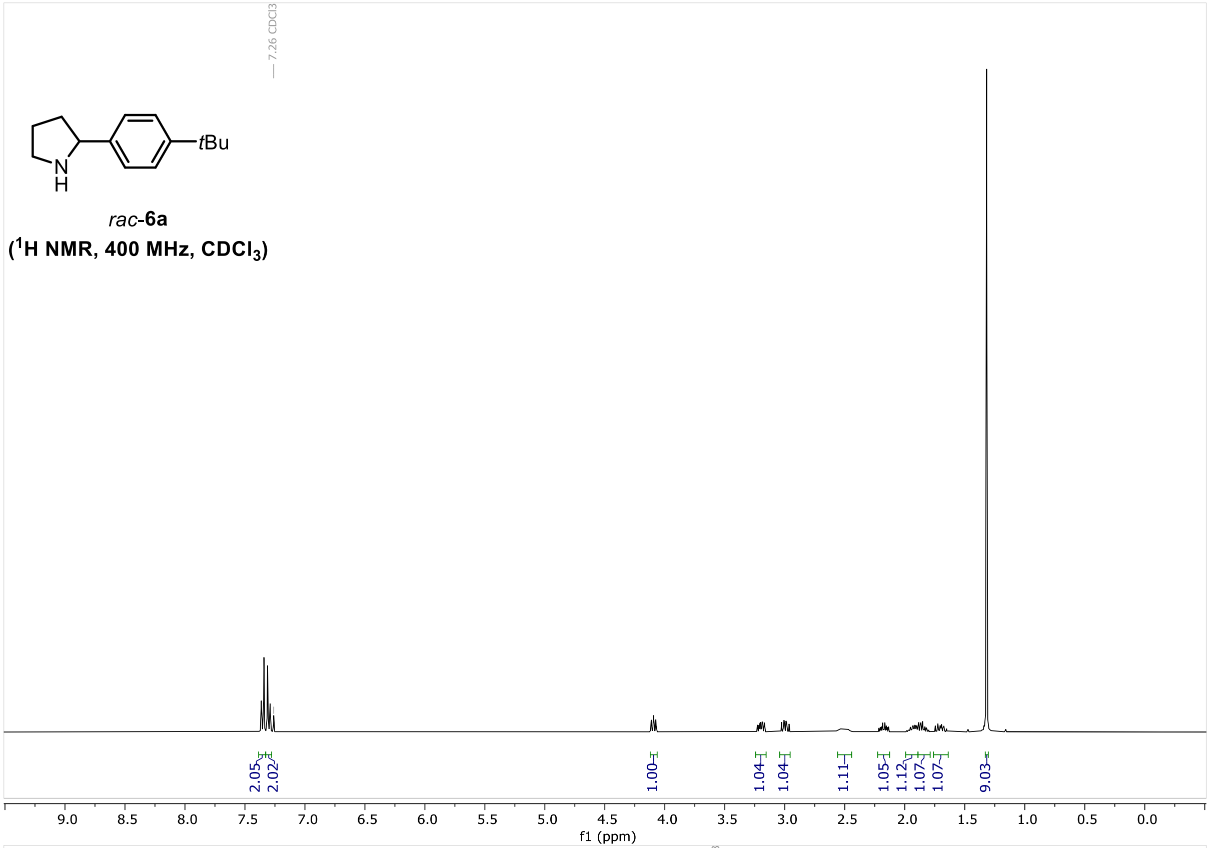

rac-**6a**
(^{1}H NMR, 400 MHz, CDCl$_3$)

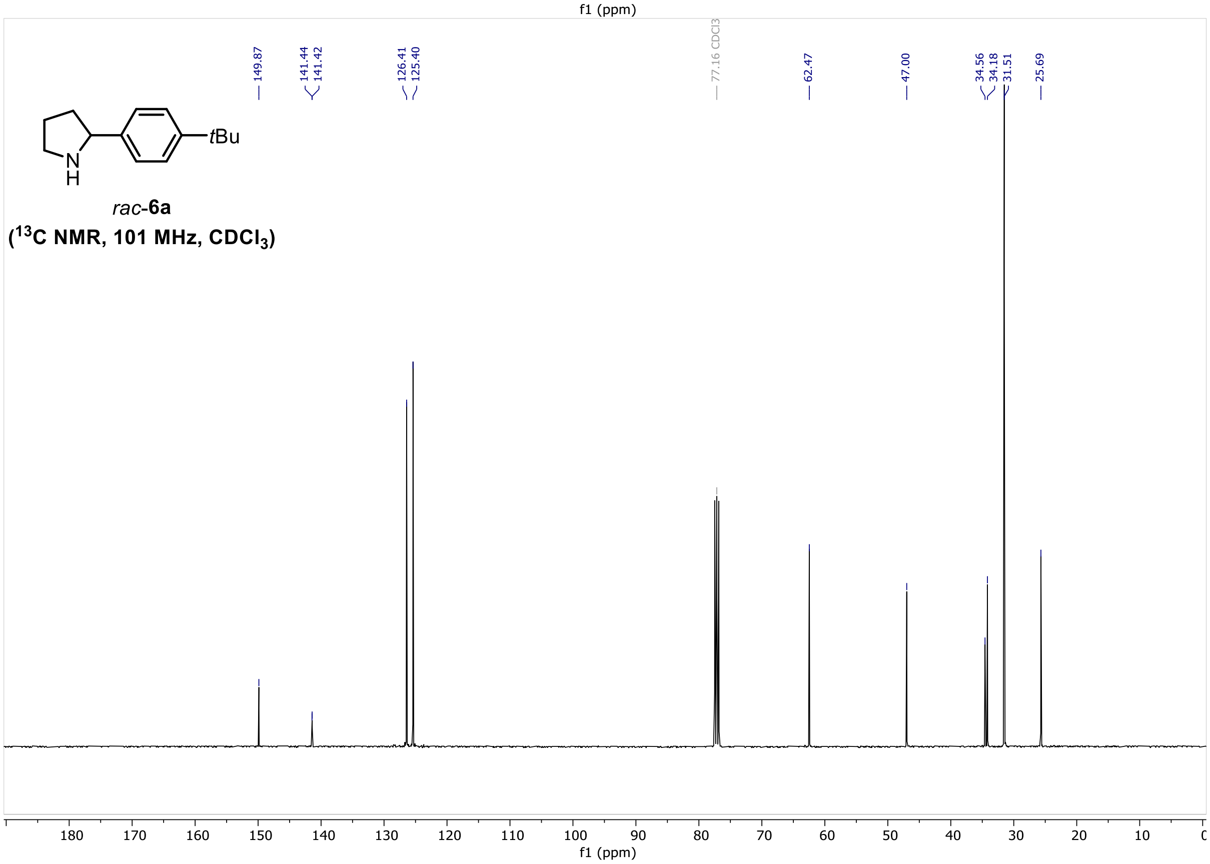

rac-**6a**
(^{13}C NMR, 101 MHz, CDCl$_3$)

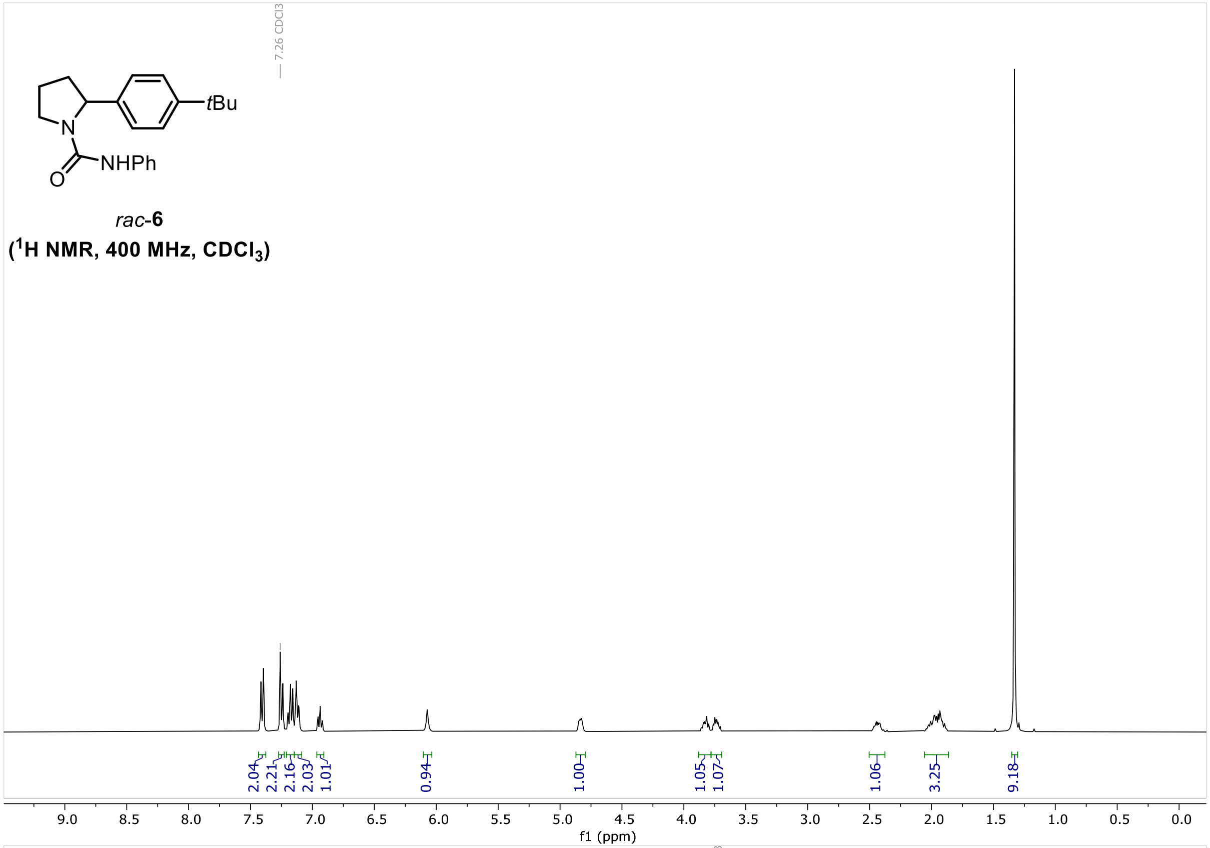

tBu
NHPh
rac-6
(1H NMR, 400 MHz, CDCl3)
7.26 CDCl3
2.04
2.21
2.16
2.03
1.01
0.94
1.00
1.05
1.07
1.06
3.25
9.18
f1 (ppm)

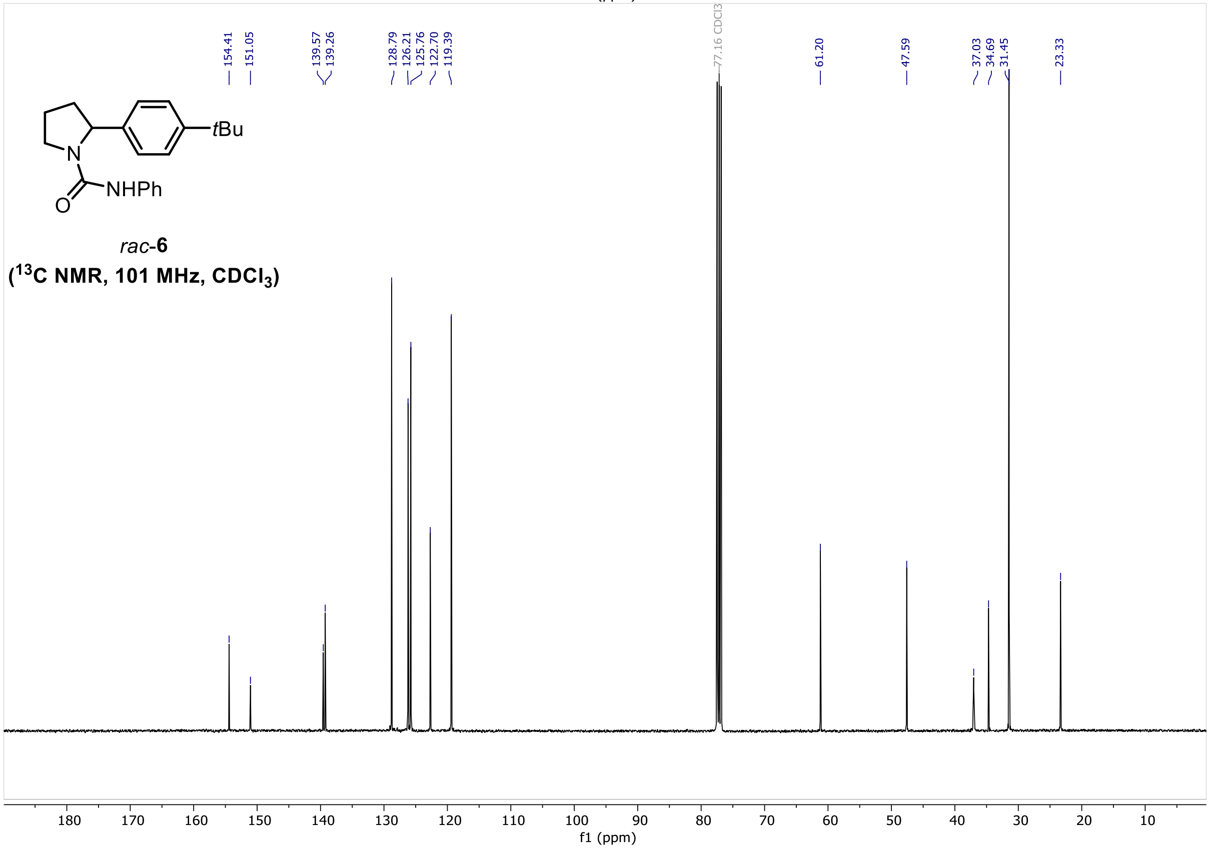

154.41
151.05
139.57
139.26
128.79
126.21
125.76
122.70
119.39
77.16 CDCl3
61.20
47.59
37.03
34.69
31.45
23.33
tBu
NHPh
rac-6
(13C NMR, 101 MHz, CDCl3)
f1 (ppm)

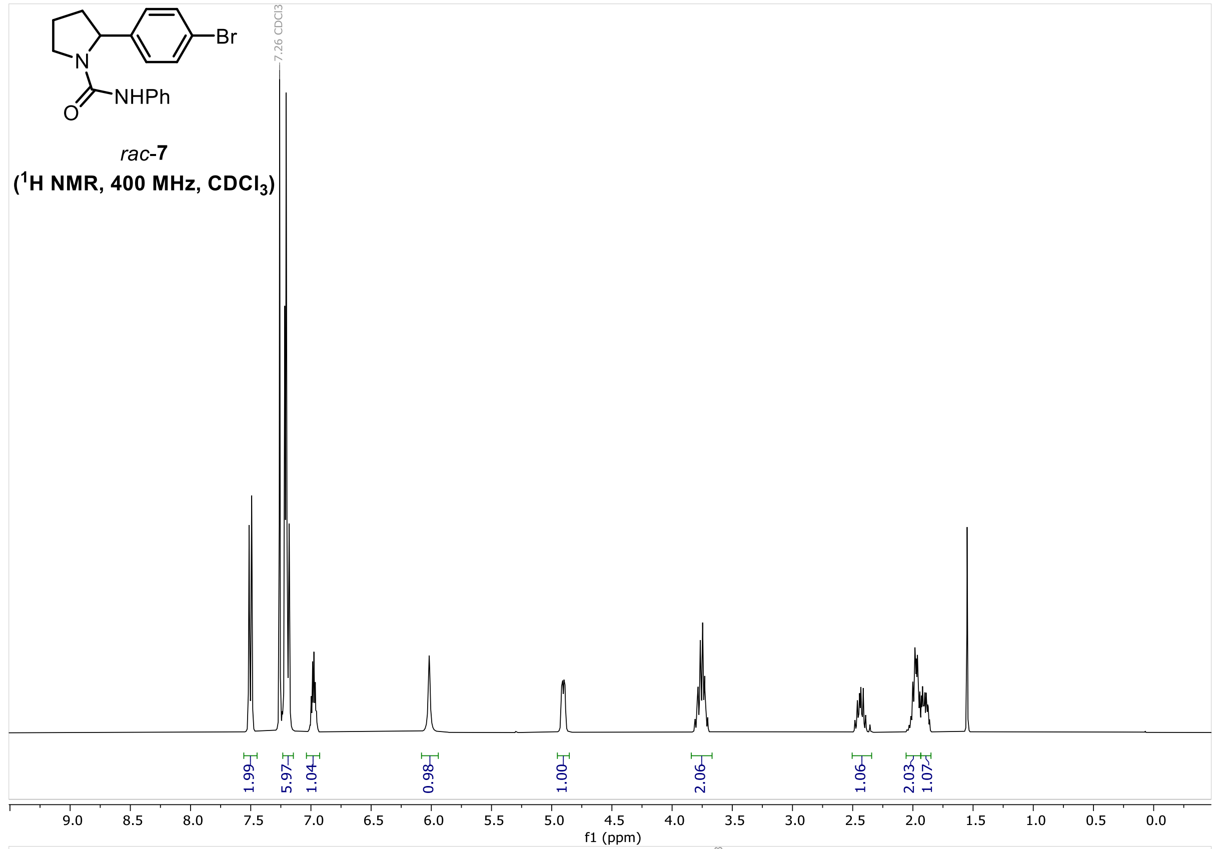
Br
N
O
NHPh
rac-7
(^{1}H NMR, 400 MHz, CDCl$_3$)
7.26 CDCl3
1.99
5.97
1.04
0.98
1.00
2.06
1.06
2.03
1.07
9.0
8.5
8.0
7.5
7.0
6.5
6.0
5.5
5.0
4.5
4.0
3.5
3.0
2.5
2.0
1.5
1.0
0.5
0.0
f1 (ppm)

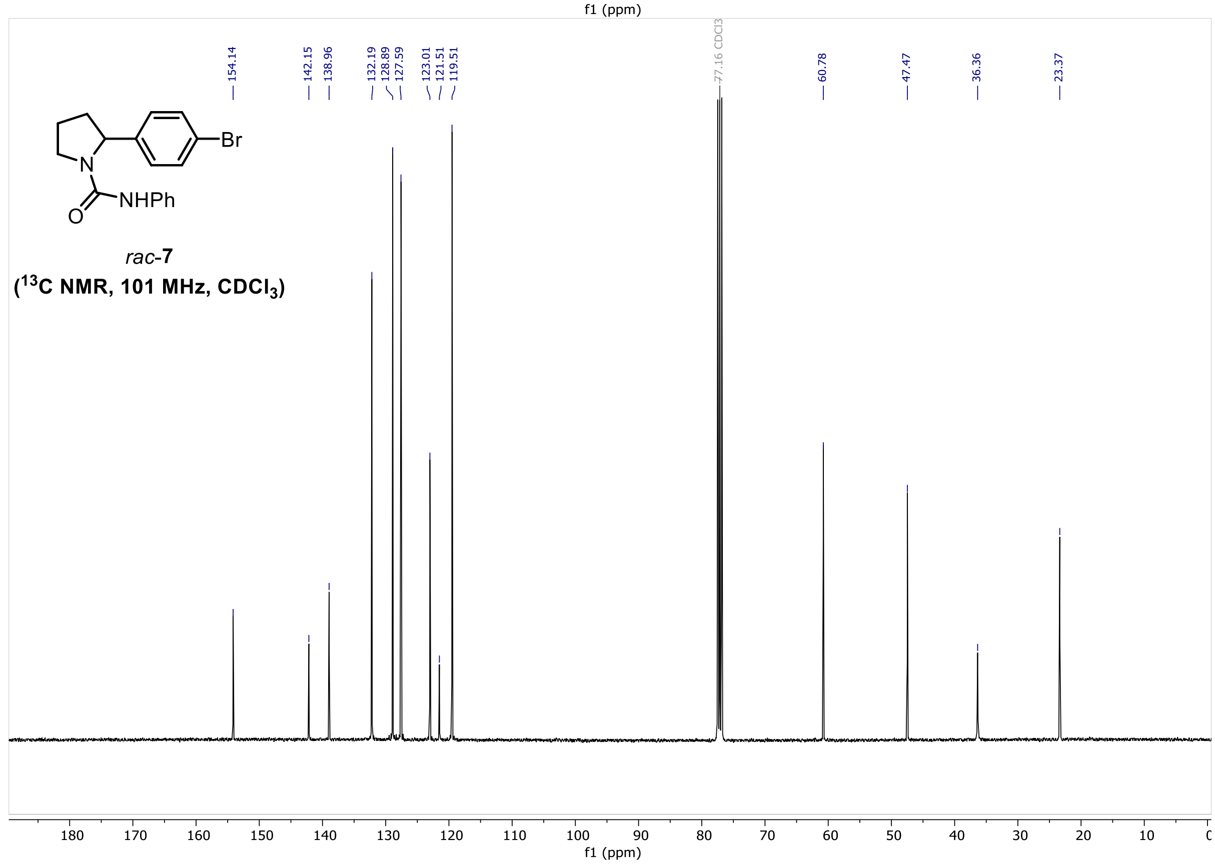
154.14
142.15
138.96
132.19
128.89
127.59
123.01
121.51
119.51
77.16 CDCl3
60.78
47.47
36.36
23.37
Br
N
O
NHPh
rac-7
(^{13}C NMR, 101 MHz, CDCl$_3$)
180
170
160
150
140
130
120
110
100
90
80
70
60
50
40
30
20
10
f1 (ppm)

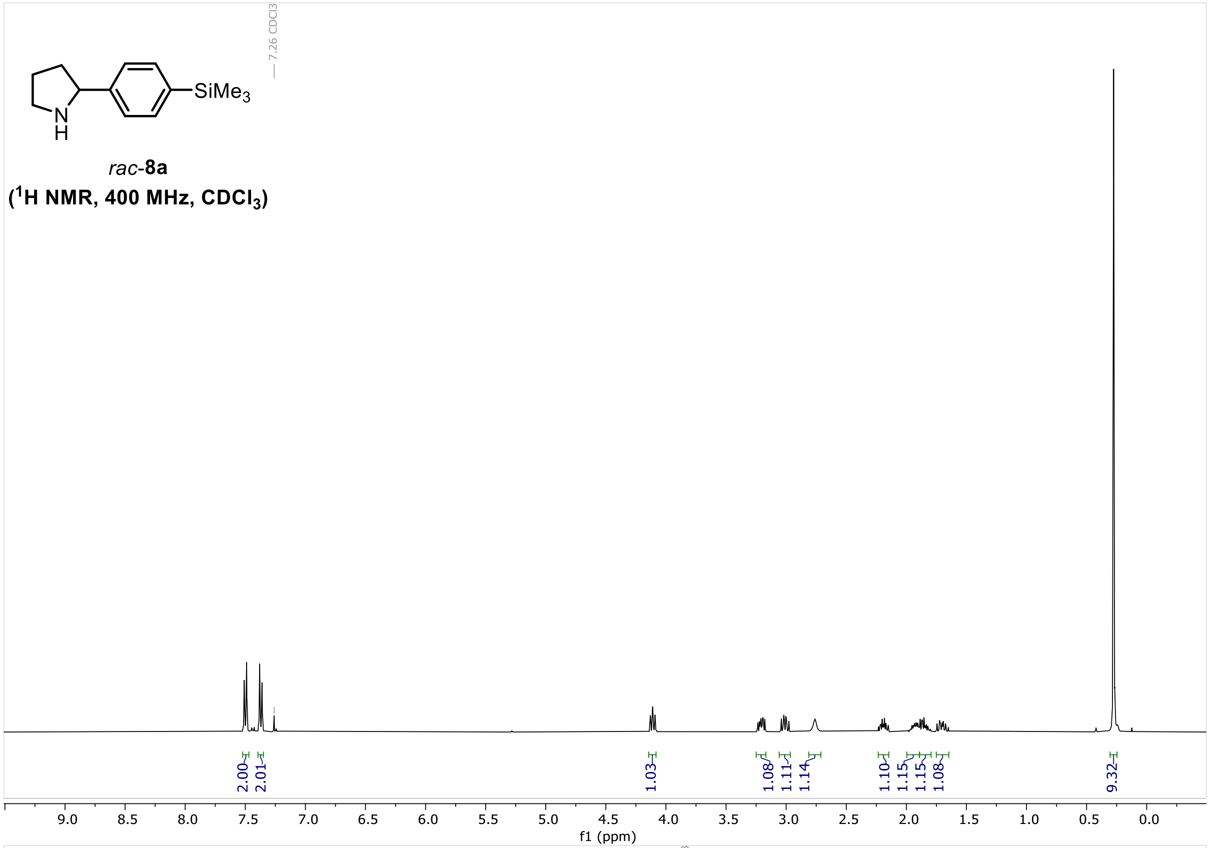

SiMe3
N
H
rac-8a
(1H NMR, 400 MHz, CDCl3)
7.26 CDCl3
2.00
2.01
1.03
1.08
1.11
1.14
1.10
1.15
1.15
1.08
9.32
f1 (ppm)

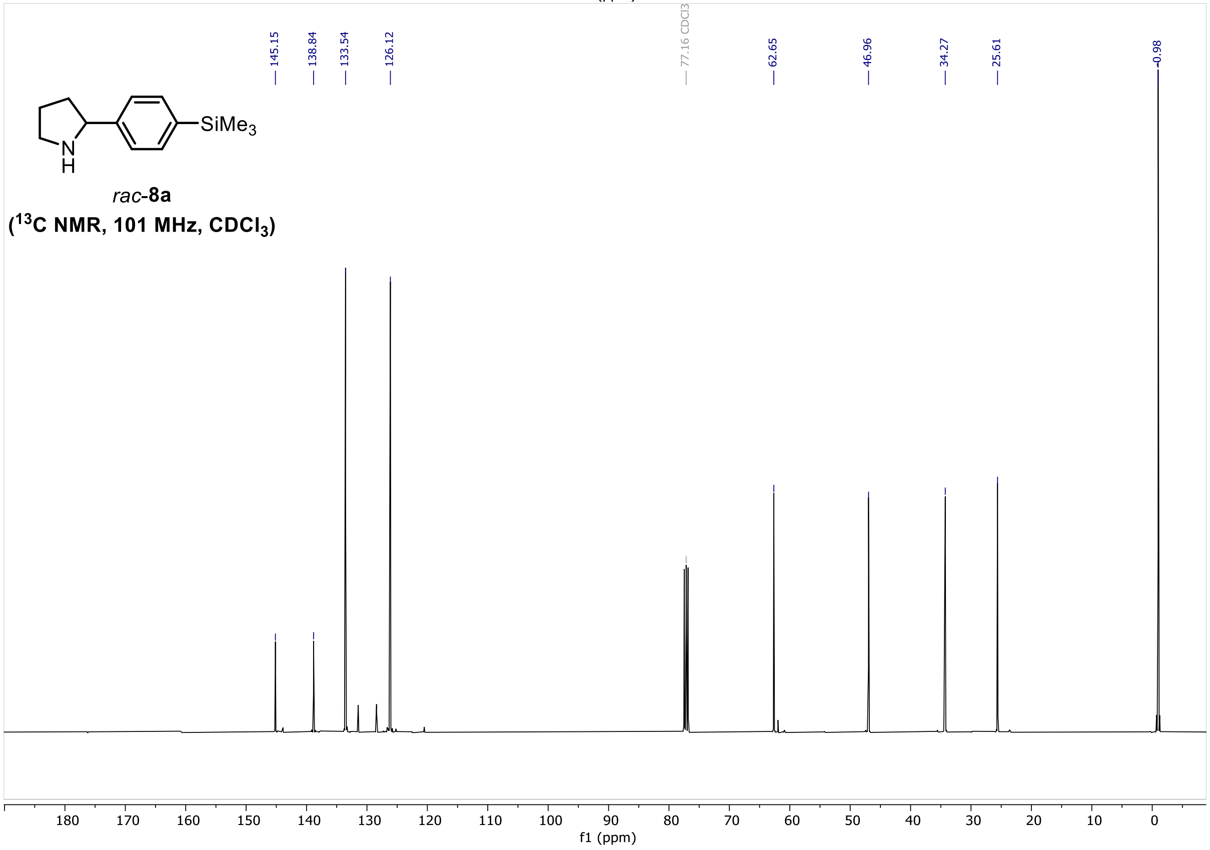

SiMe3
N
H
rac-8a
(13C NMR, 101 MHz, CDCl3)
145.15
138.84
133.54
126.12
77.16 CDCl3
62.65
46.96
34.27
25.61
-0.98
f1 (ppm)

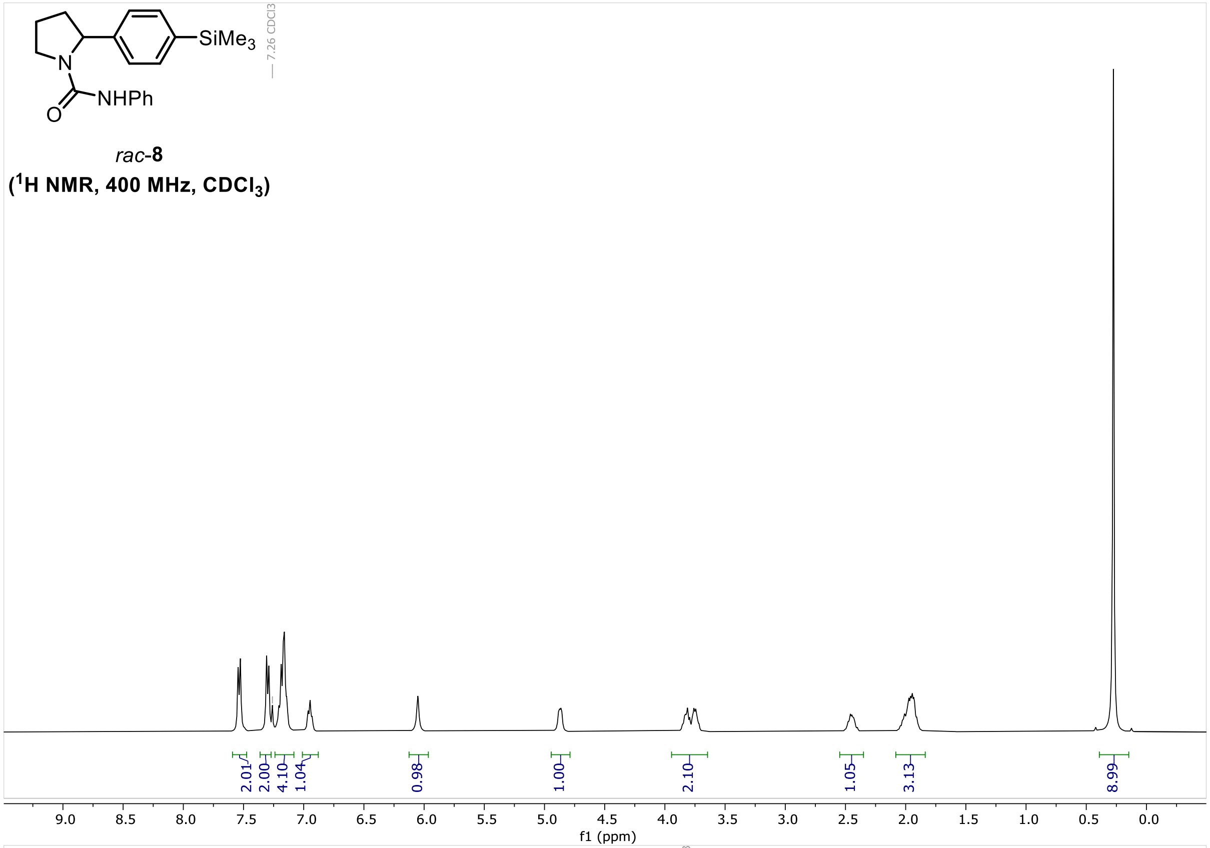

rac-**8**
(^{1}H NMR, 400 MHz, $CDCl_3$)

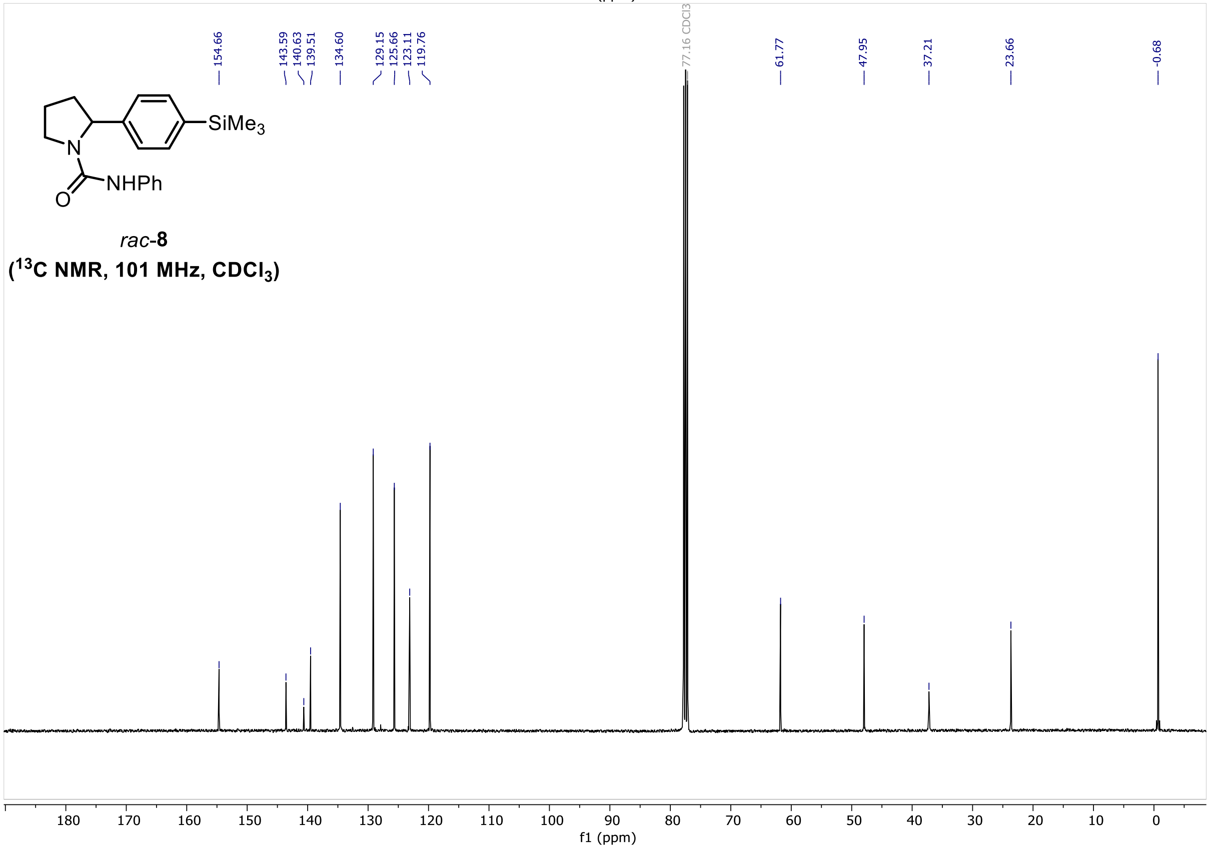

rac-**8**
(^{13}C NMR, 101 MHz, $CDCl_3$)

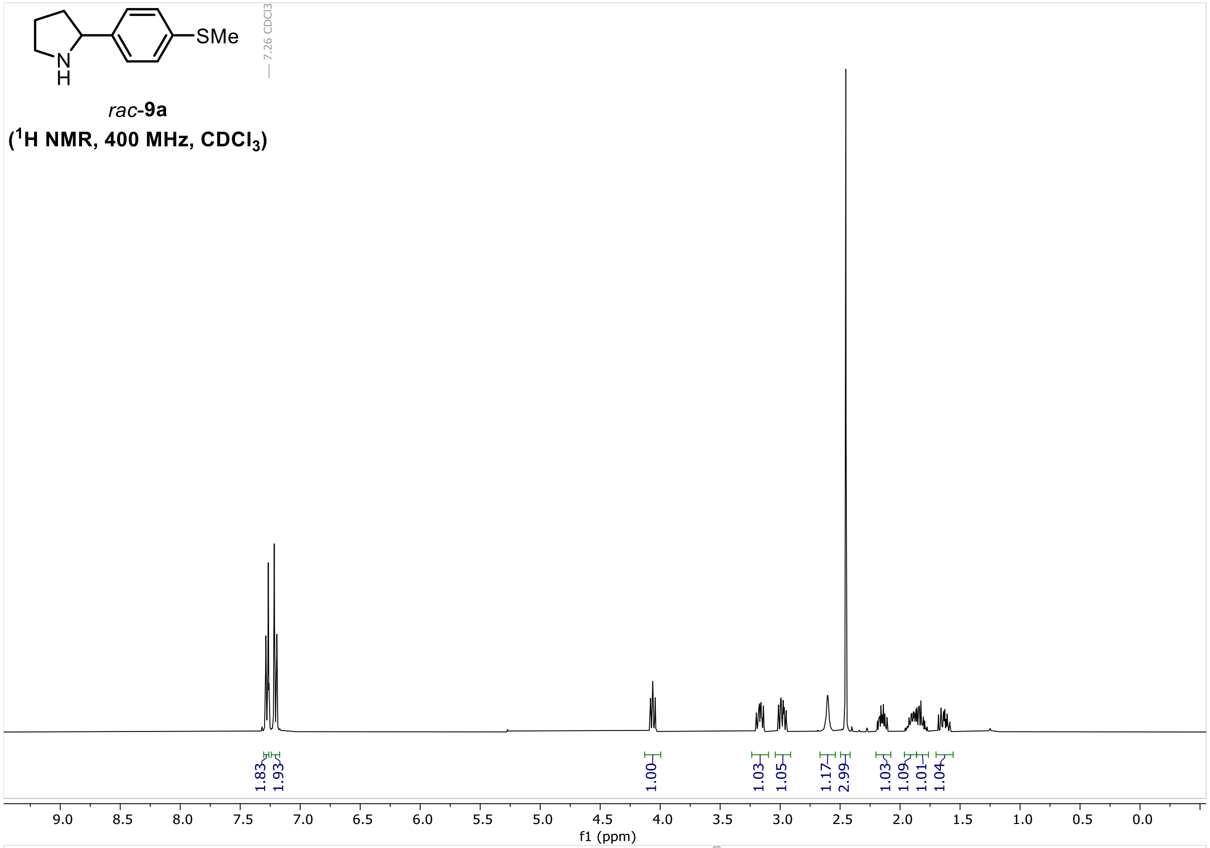
SMe
N
H
rac-9a
(1H NMR, 400 MHz, CDCl3)
7.26 CDCl3
1.83
1.93
1.00
1.03
1.05
1.17
2.99
1.03
1.09
1.01
1.04
9.0 8.5 8.0 7.5 7.0 6.5 6.0 5.5 5.0 4.5 4.0 3.5 3.0 2.5 2.0 1.5 1.0 0.5 0.0
f1 (ppm)

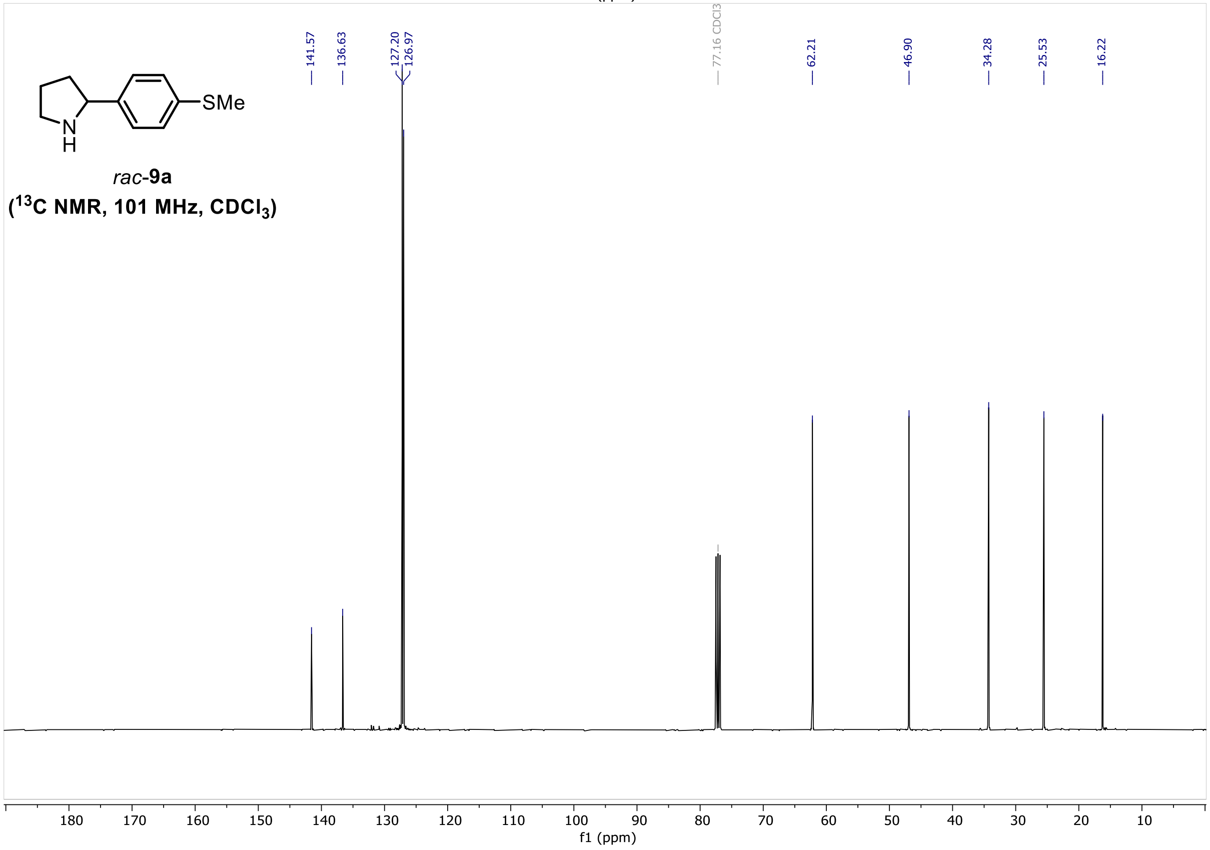
SMe
N
H
rac-9a
(13C NMR, 101 MHz, CDCl3)
141.57
136.63
127.20
126.97
77.16 CDCl3
62.21
46.90
34.28
25.53
16.22
180 170 160 150 140 130 120 110 100 90 80 70 60 50 40 30 20 10
f1 (ppm)

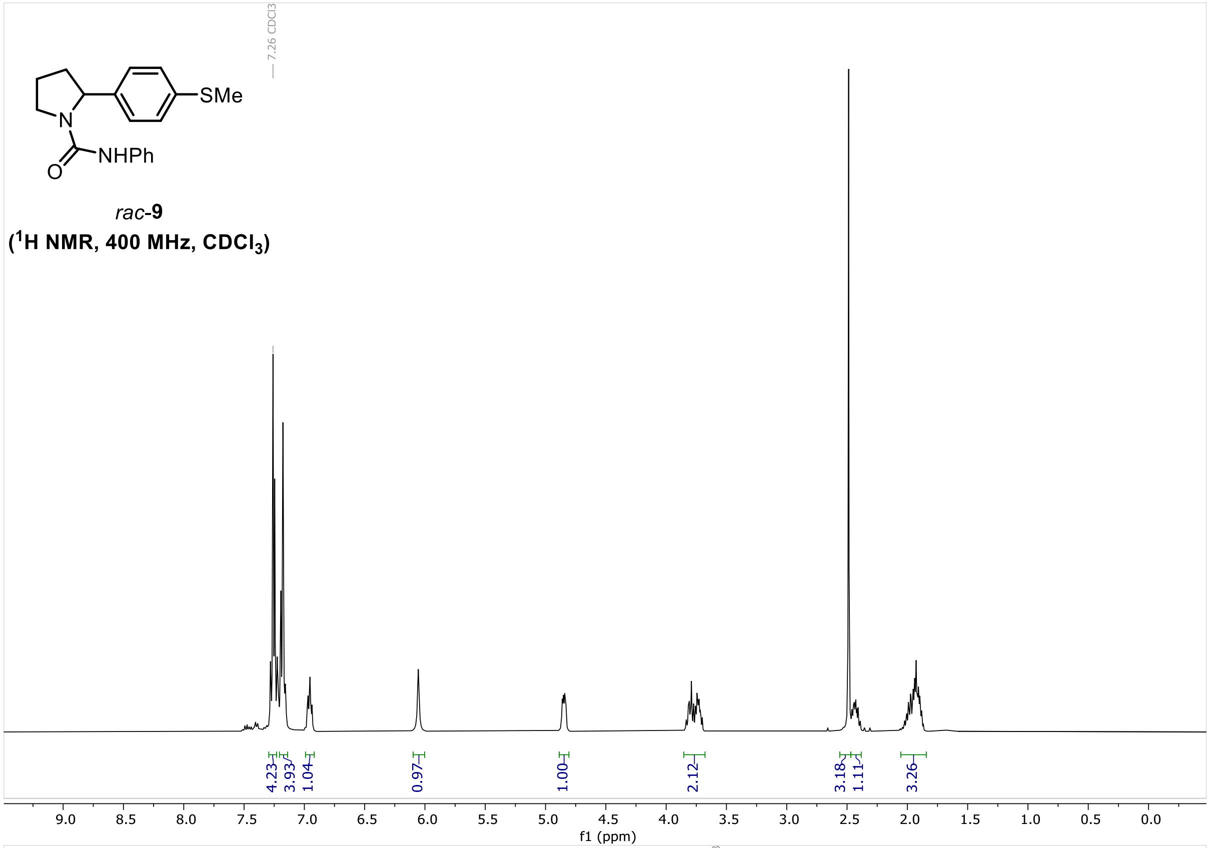

rac-**9**
(^{1}H NMR, 400 MHz, CDCl$_3$)

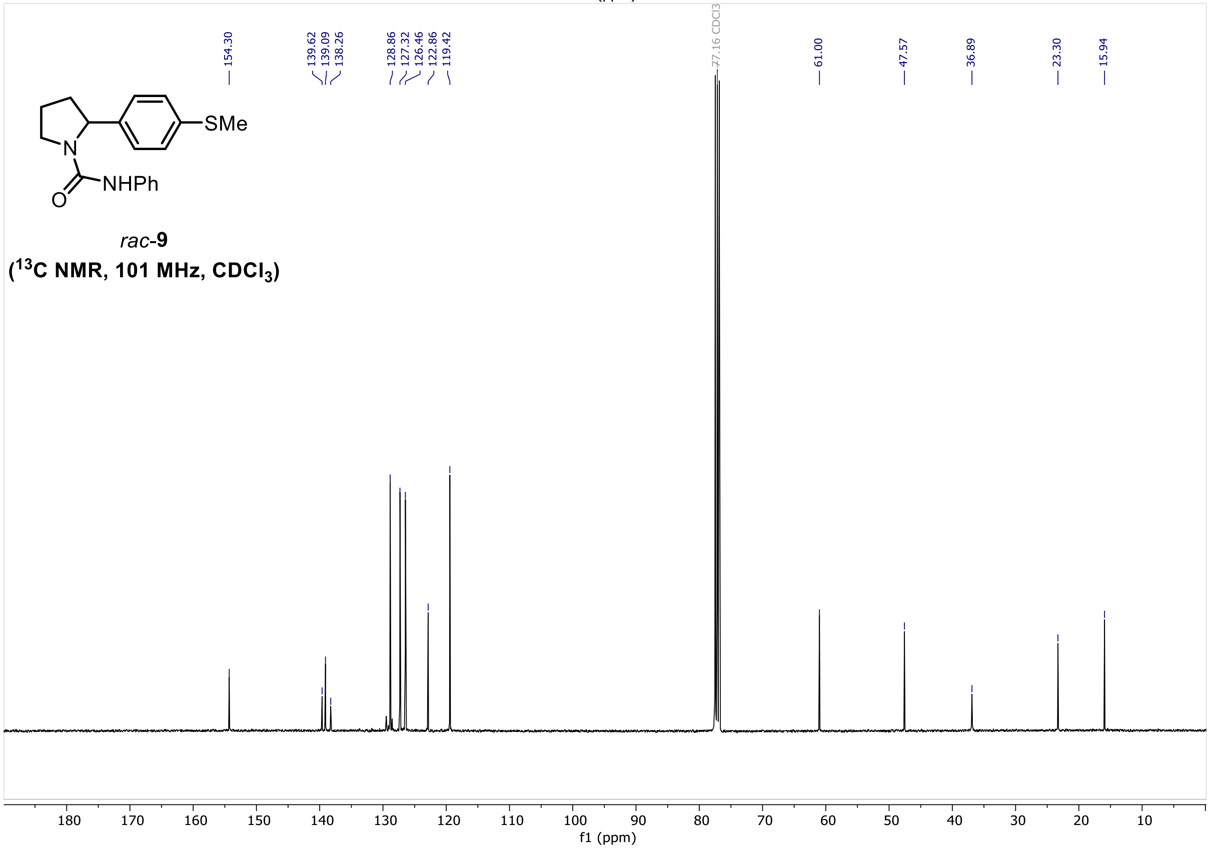

rac-**9**
(^{13}C NMR, 101 MHz, CDCl$_3$)

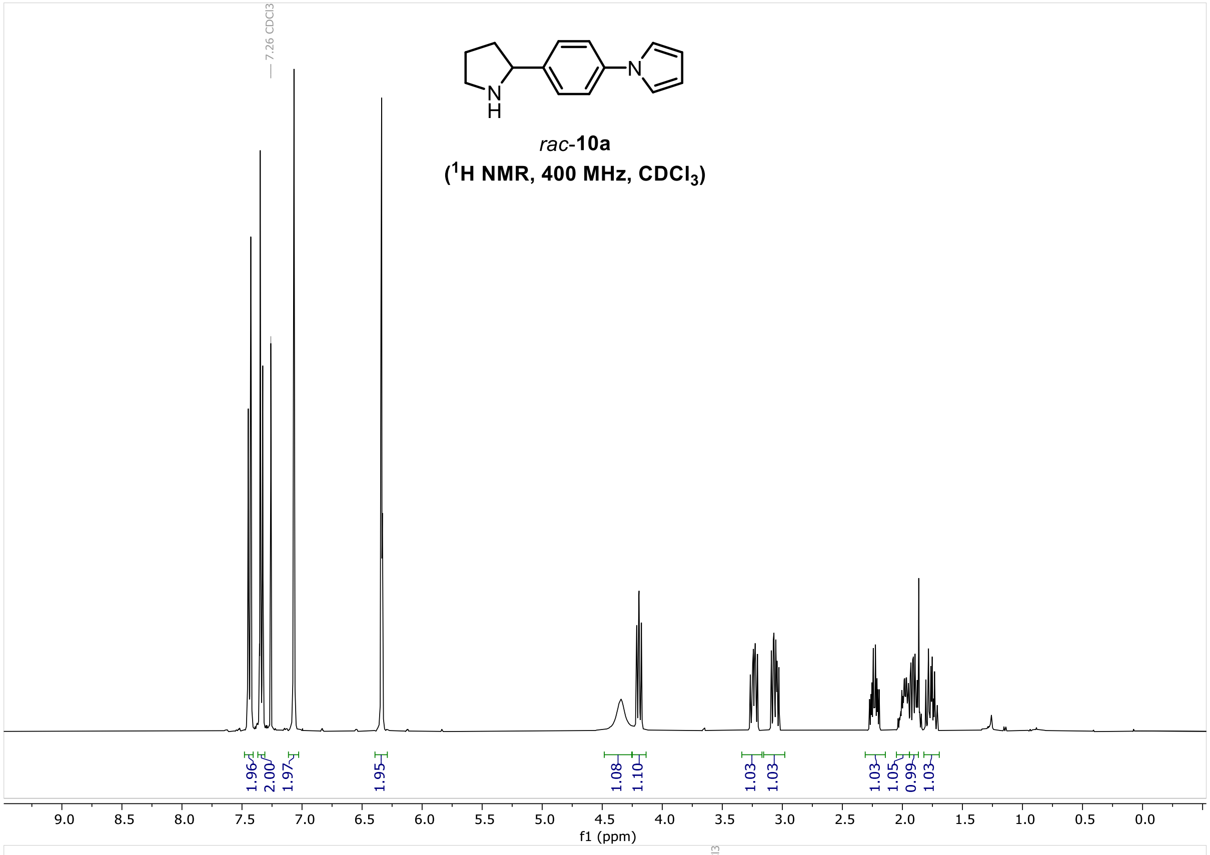

rac-10a
(1H NMR, 400 MHz, CDCl3)
7.26 CDCl3
1.96
2.00
1.97
1.95
1.08
1.10
1.03
1.03
1.03
1.05
0.99
1.03
f1 (ppm)

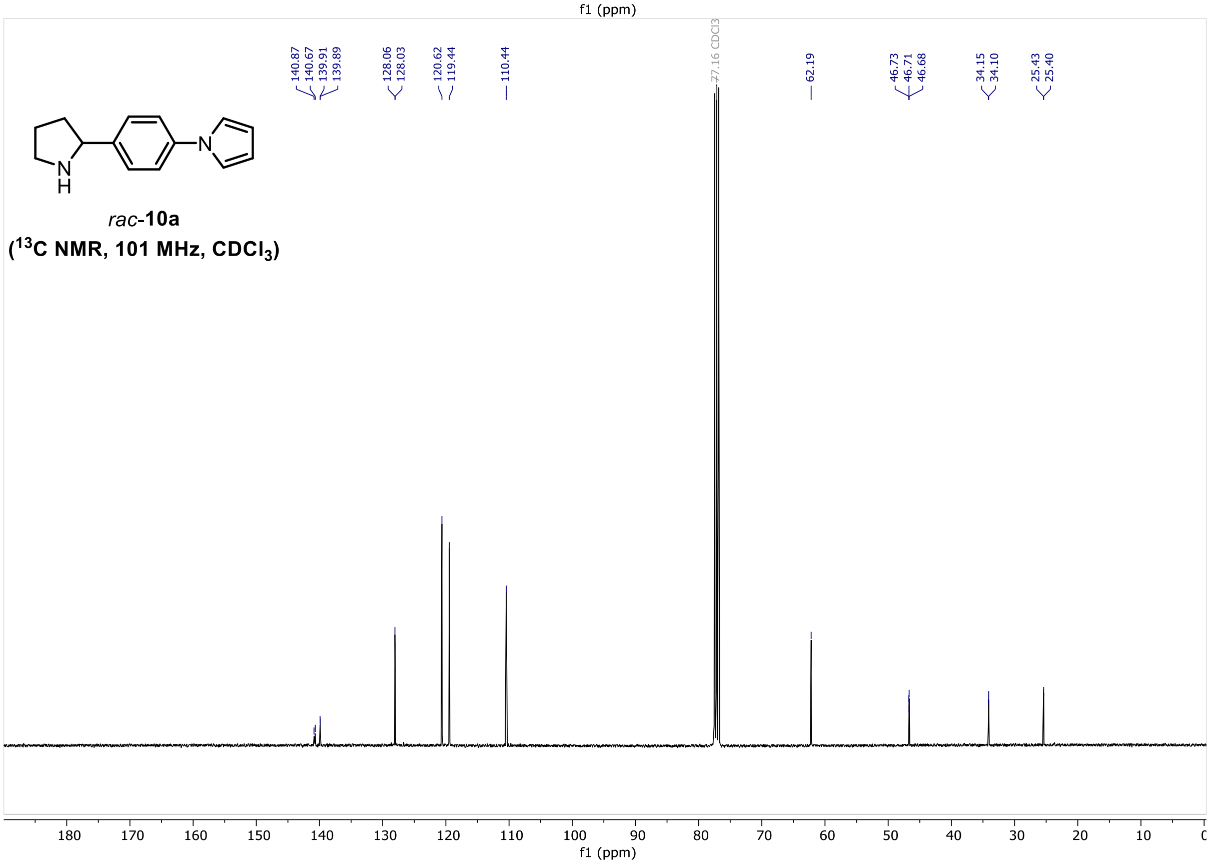

rac-10a
(13C NMR, 101 MHz, CDCl3)
140.87
140.67
139.91
139.89
128.06
128.03
120.62
119.44
110.44
77.16 CDCl3
62.19
46.73
46.71
46.68
34.15
34.10
25.43
25.40
f1 (ppm)

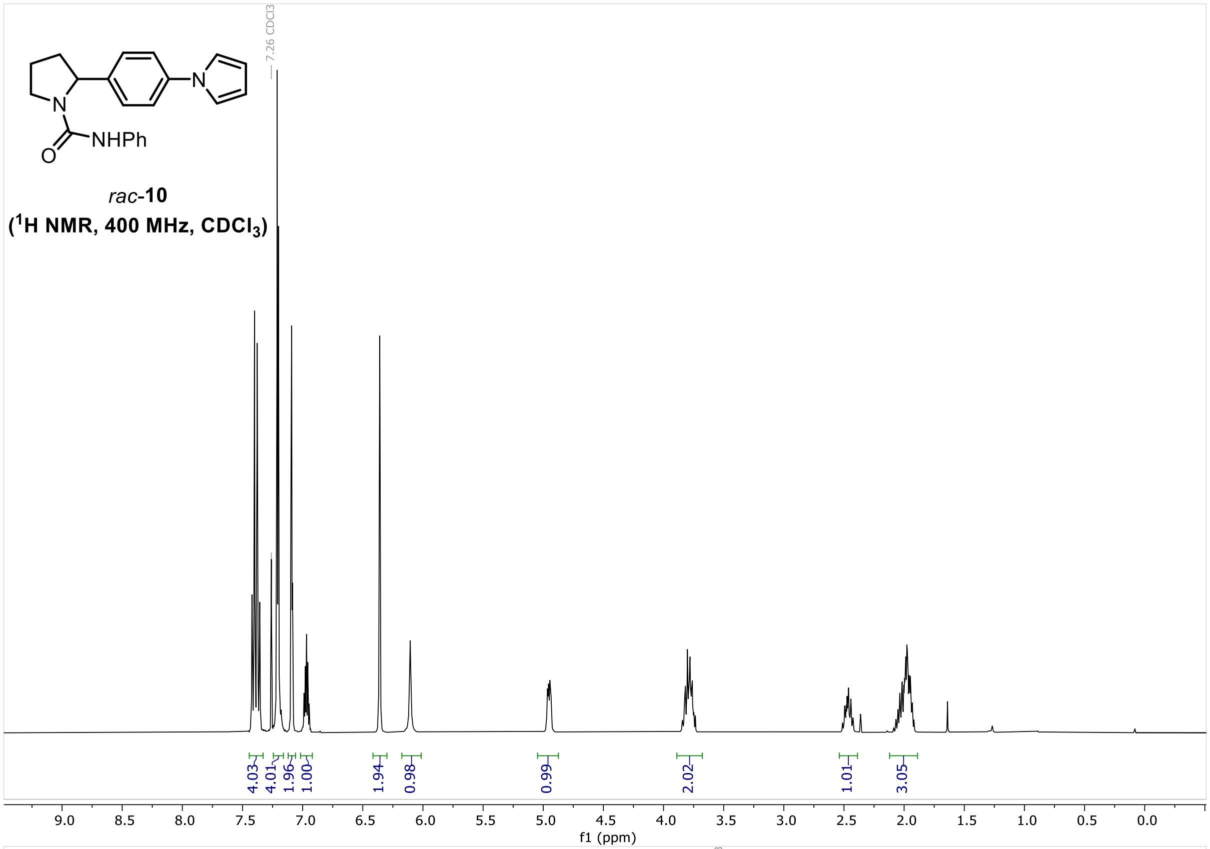
NHPh
O
N
rac-10
(1H NMR, 400 MHz, CDCl3)
7.26 CDCl3
4.03
4.01
1.96
1.00
1.94
0.98
0.99
2.02
1.01
3.05
9.0
8.5
8.0
7.5
7.0
6.5
6.0
5.5
5.0
4.5
4.0
3.5
3.0
2.5
2.0
1.5
1.0
0.5
0.0
f1 (ppm)

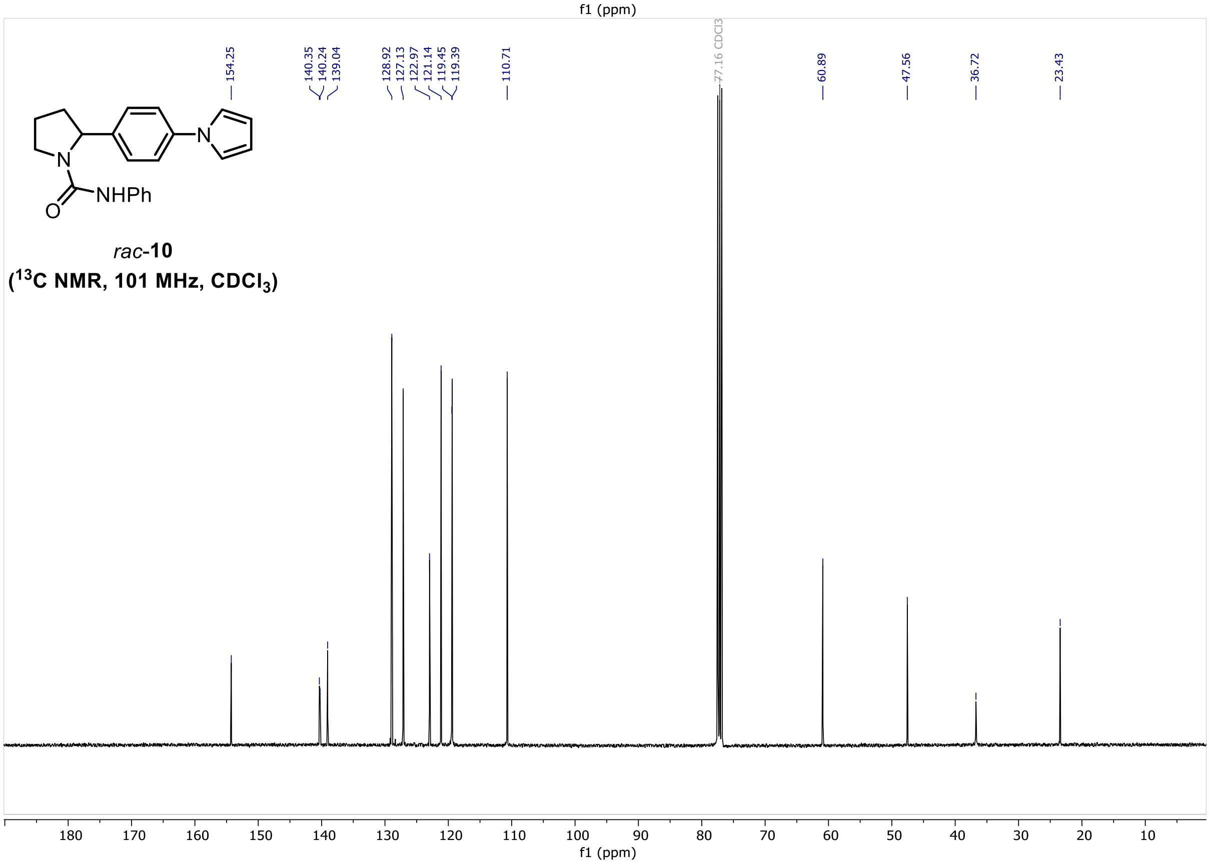
NHPh
O
N
rac-10
(13C NMR, 101 MHz, CDCl3)
154.25
140.35
140.24
139.04
128.92
127.13
122.97
121.14
119.45
119.39
110.71
77.16 CDCl3
60.89
47.56
36.72
23.43
180
170
160
150
140
130
120
110
100
90
80
70
60
50
40
30
20
10
f1 (ppm)

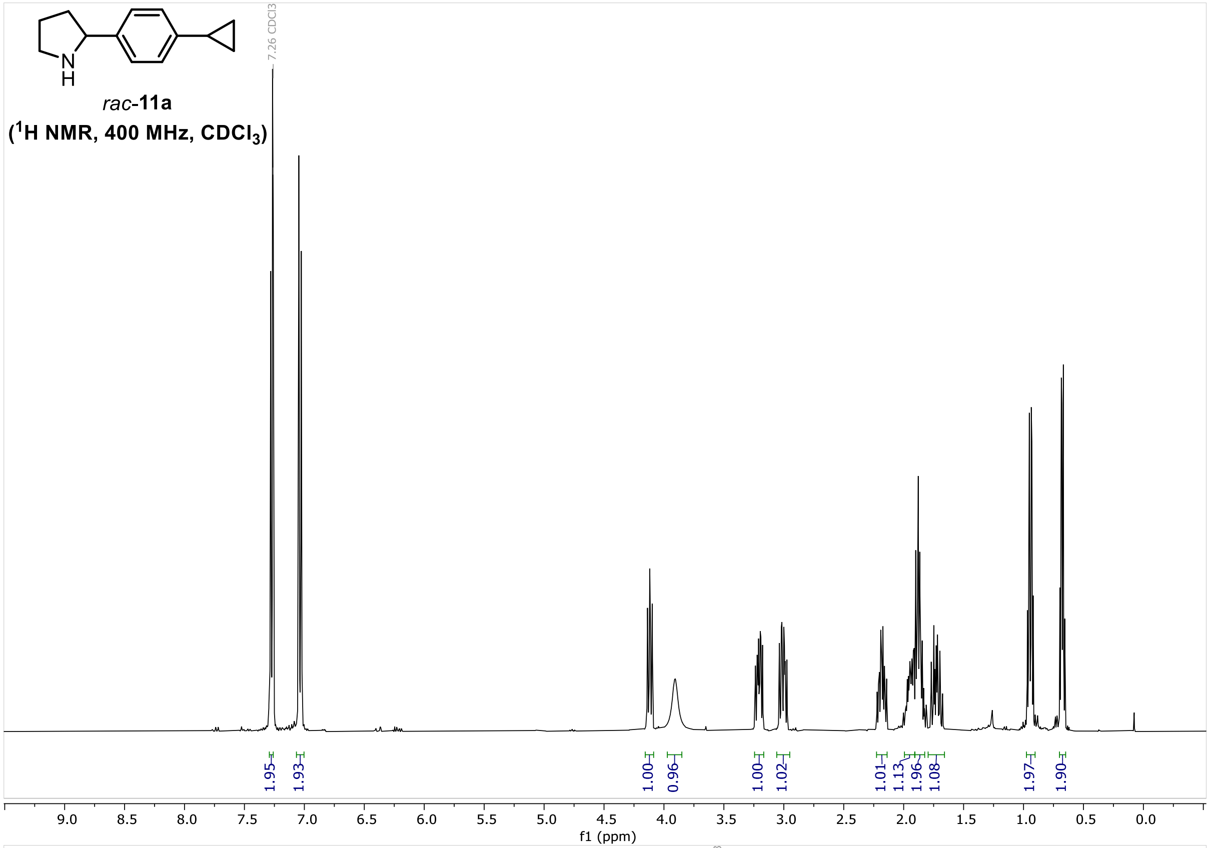

rac-11a
(¹H NMR, 400 MHz, CDCl₃)
7.26 CDCl3
1.95
1.93
1.00
0.96
1.00
1.02
1.01
1.13
1.96
1.08
1.97
1.90
9.0 8.5 8.0 7.5 7.0 6.5 6.0 5.5 5.0 4.5 4.0 3.5 3.0 2.5 2.0 1.5 1.0 0.5 0.0
f1 (ppm)

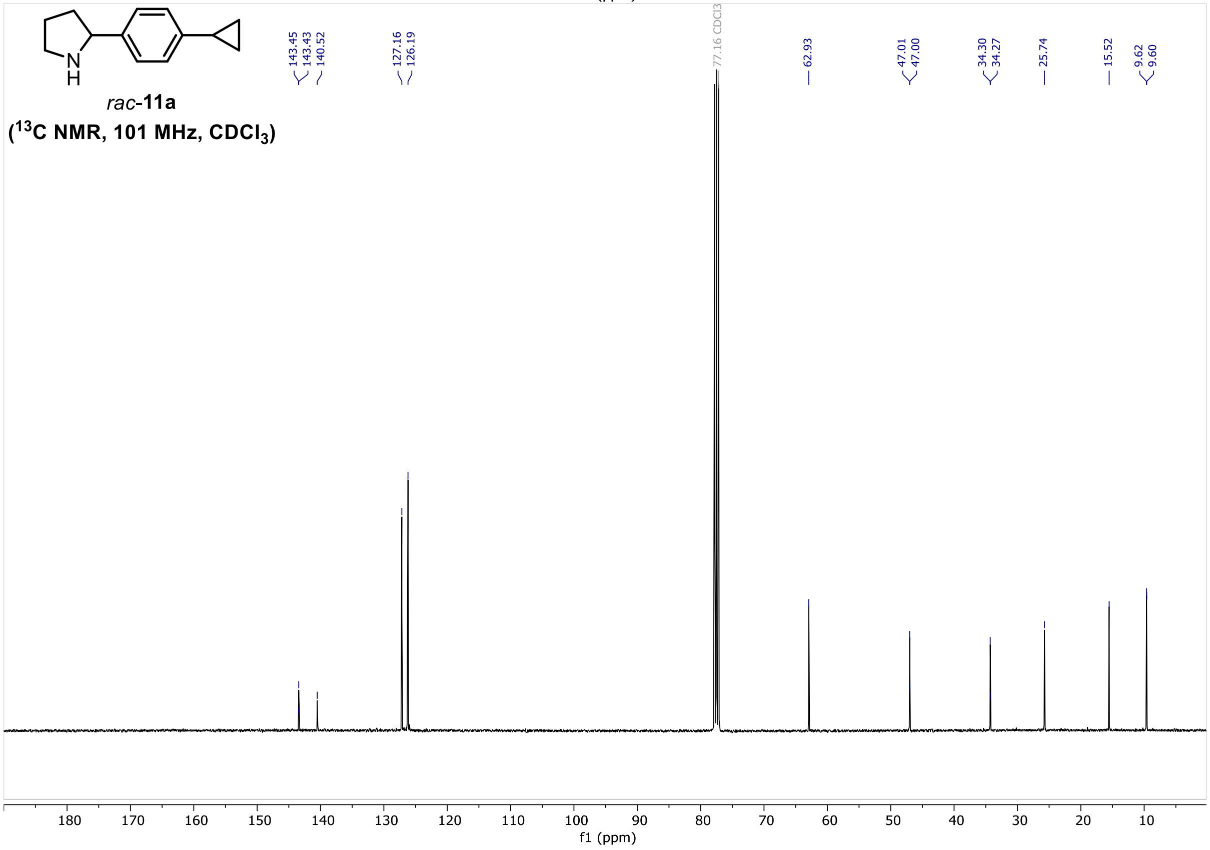

rac-11a
(¹³C NMR, 101 MHz, CDCl₃)
143.45
143.43
140.52
127.16
126.19
77.16 CDCl3
62.93
47.01
47.00
34.30
34.27
25.74
15.52
9.62
9.60
180 170 160 150 140 130 120 110 100 90 80 70 60 50 40 30 20 10
f1 (ppm)

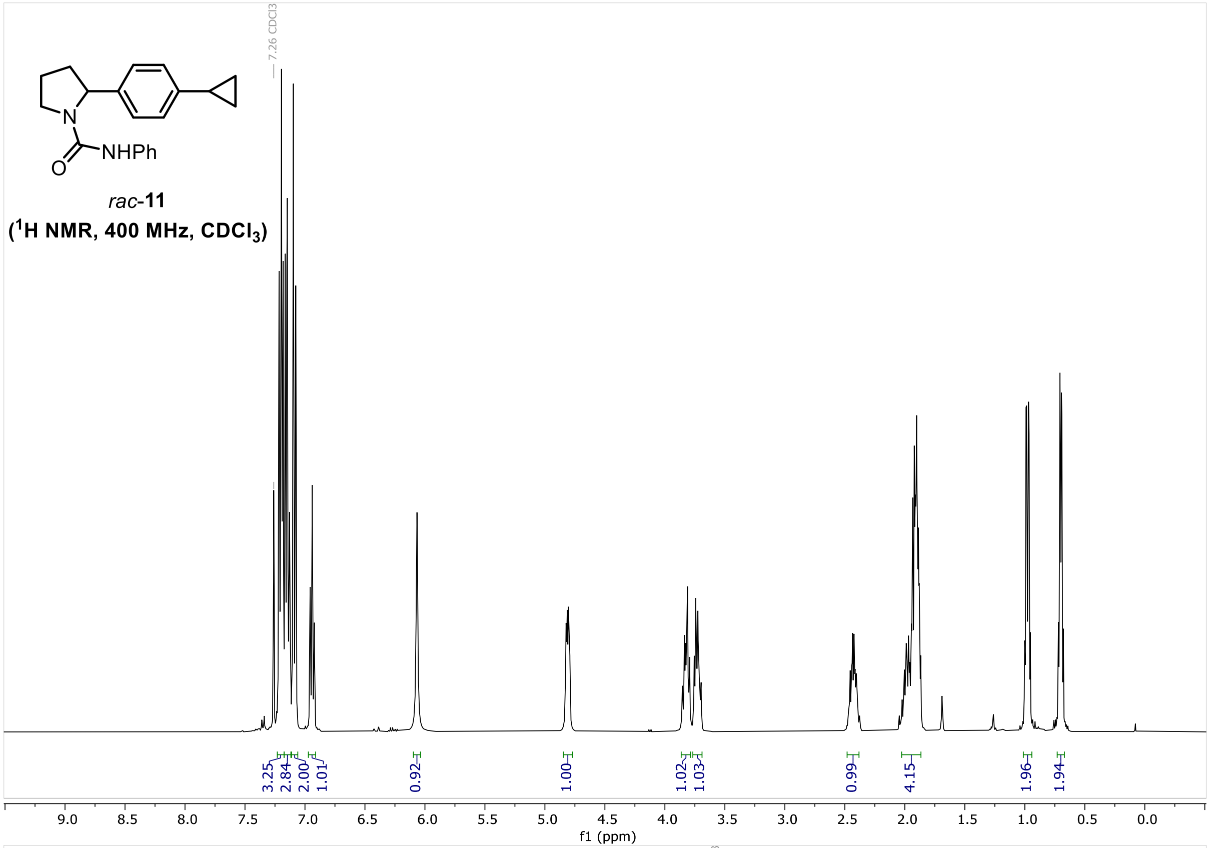

rac-**11**
(^{1}H NMR, 400 MHz, $CDCl_3$)

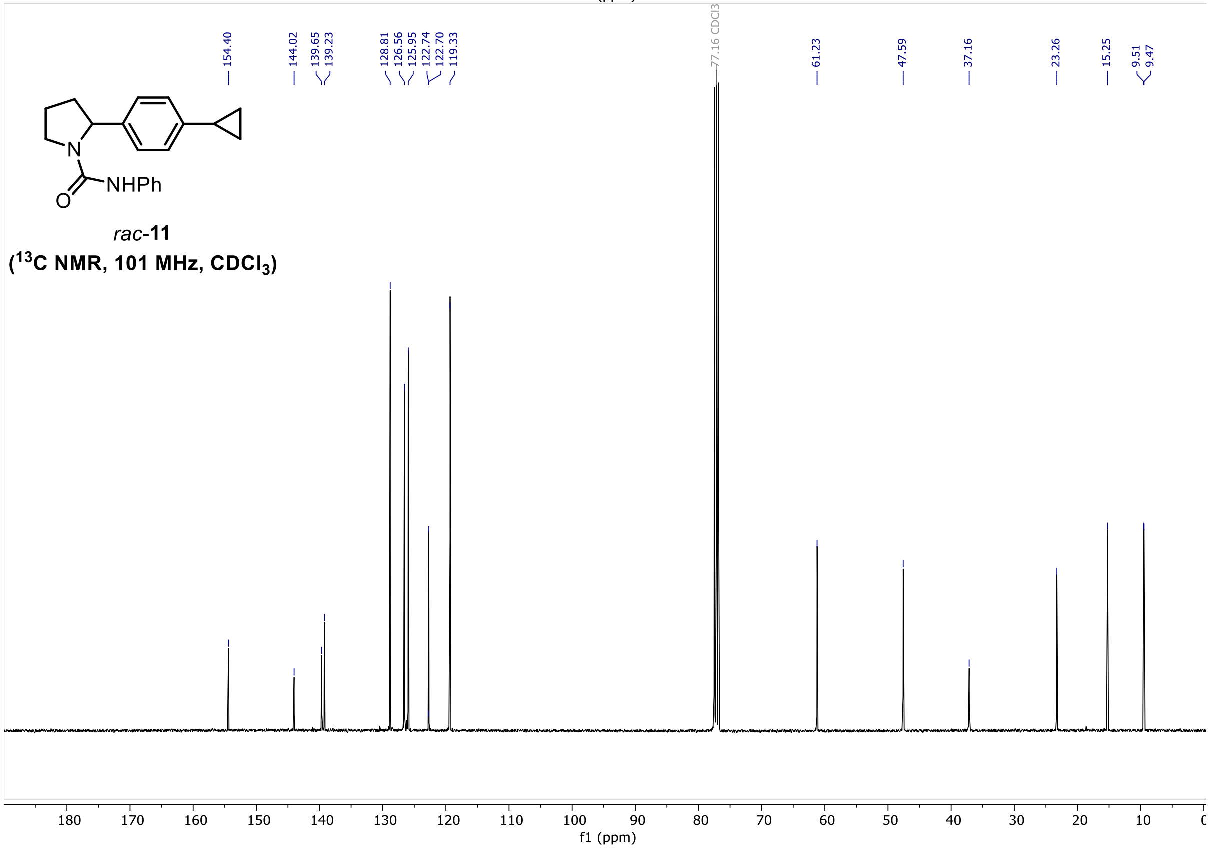

rac-**11**
(^{13}C NMR, 101 MHz, $CDCl_3$)

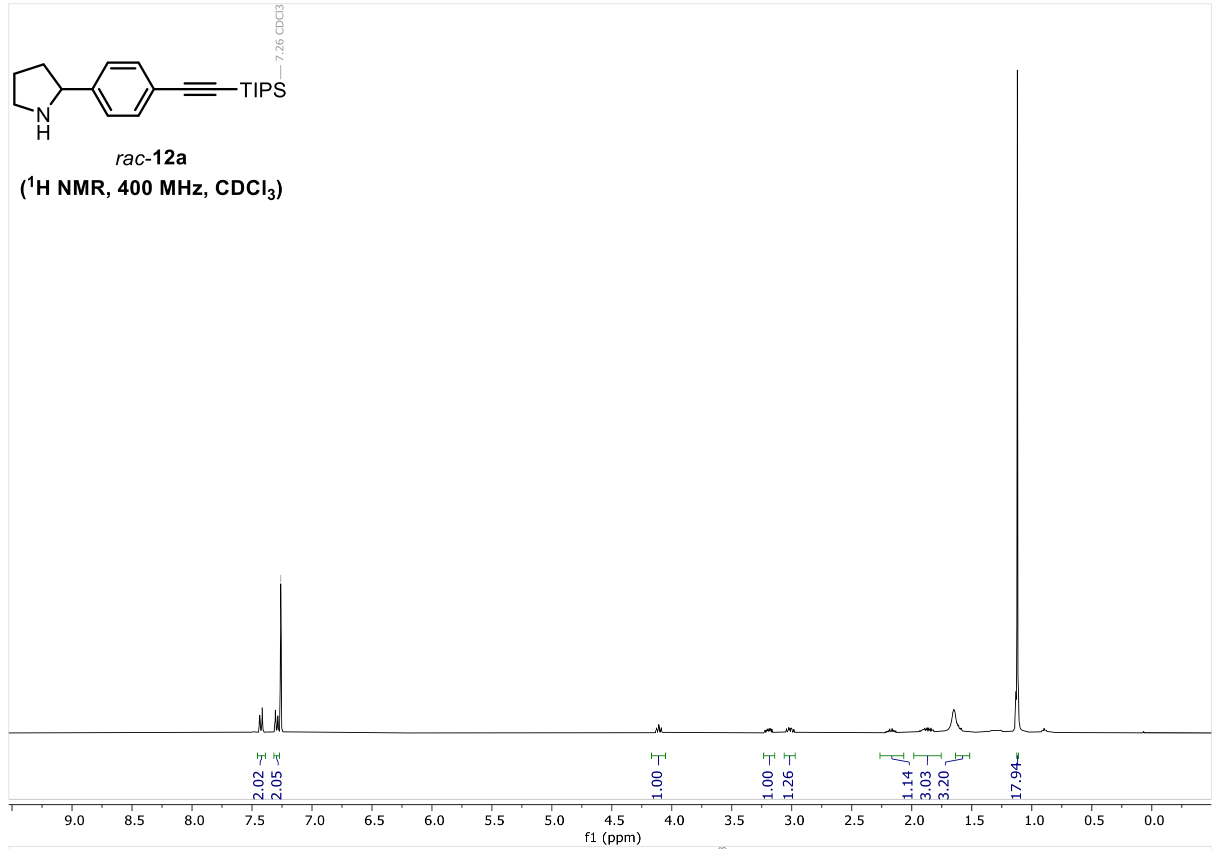

TIPS
N
H
rac-12a
(1H NMR, 400 MHz, CDCl3)
7.26 CDCl3
2.02
2.05
1.00
1.00
1.26
1.14
3.03
3.20
17.94
9.0 8.5 8.0 7.5 7.0 6.5 6.0 5.5 5.0 4.5 4.0 3.5 3.0 2.5 2.0 1.5 1.0 0.5 0.0
f1 (ppm)

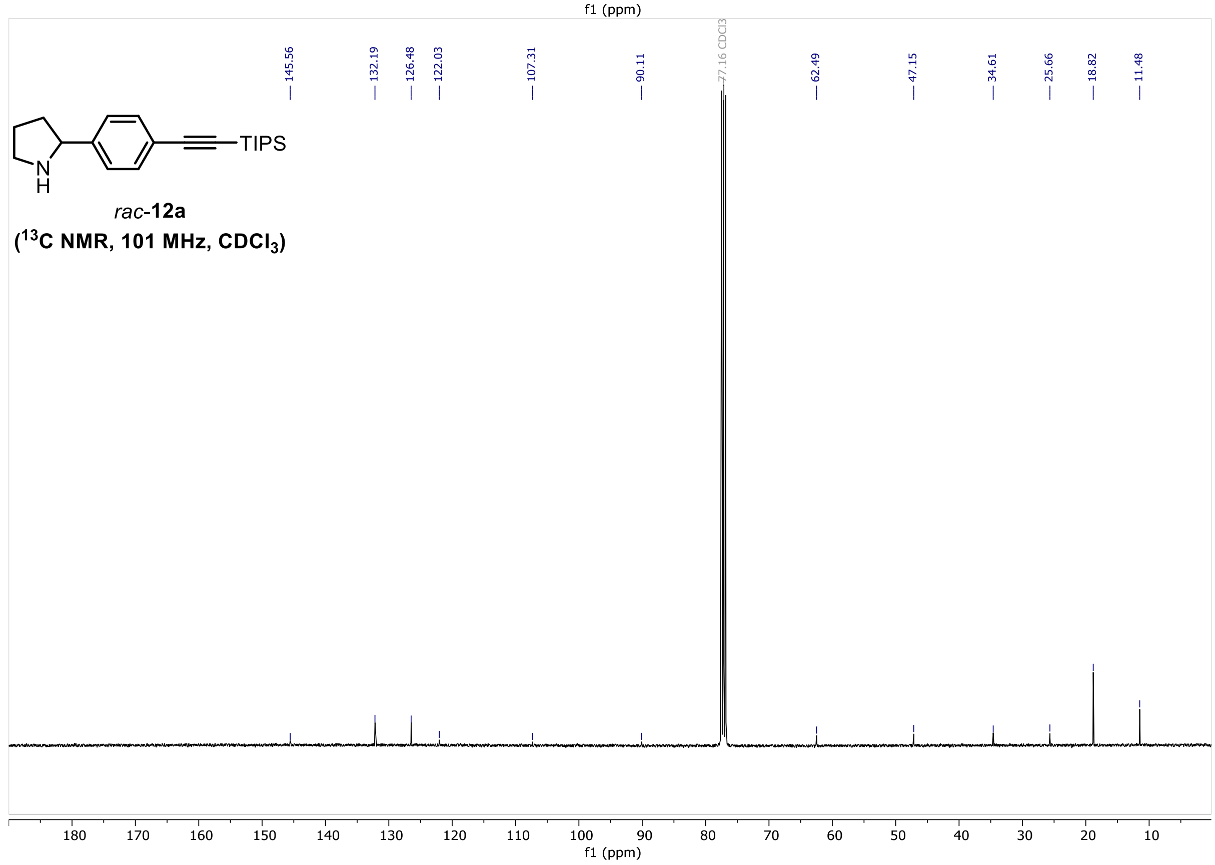

TIPS
N
H
rac-12a
(13C NMR, 101 MHz, CDCl3)
145.56
132.19
126.48
122.03
107.31
90.11
77.16 CDCl3
62.49
47.15
34.61
25.66
18.82
11.48
180 170 160 150 140 130 120 110 100 90 80 70 60 50 40 30 20 10
f1 (ppm)

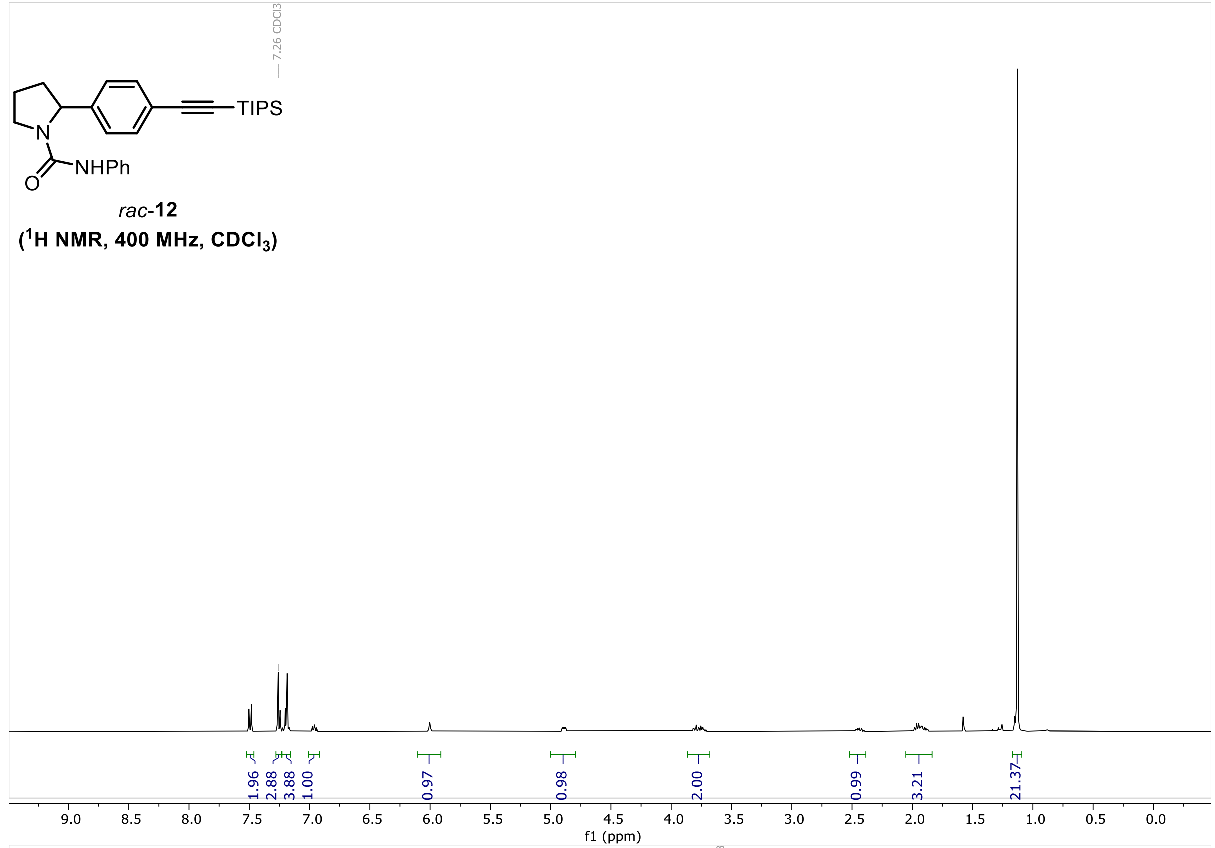

rac-**12**
(^{1}H NMR, 400 MHz, CDCl$_3$)

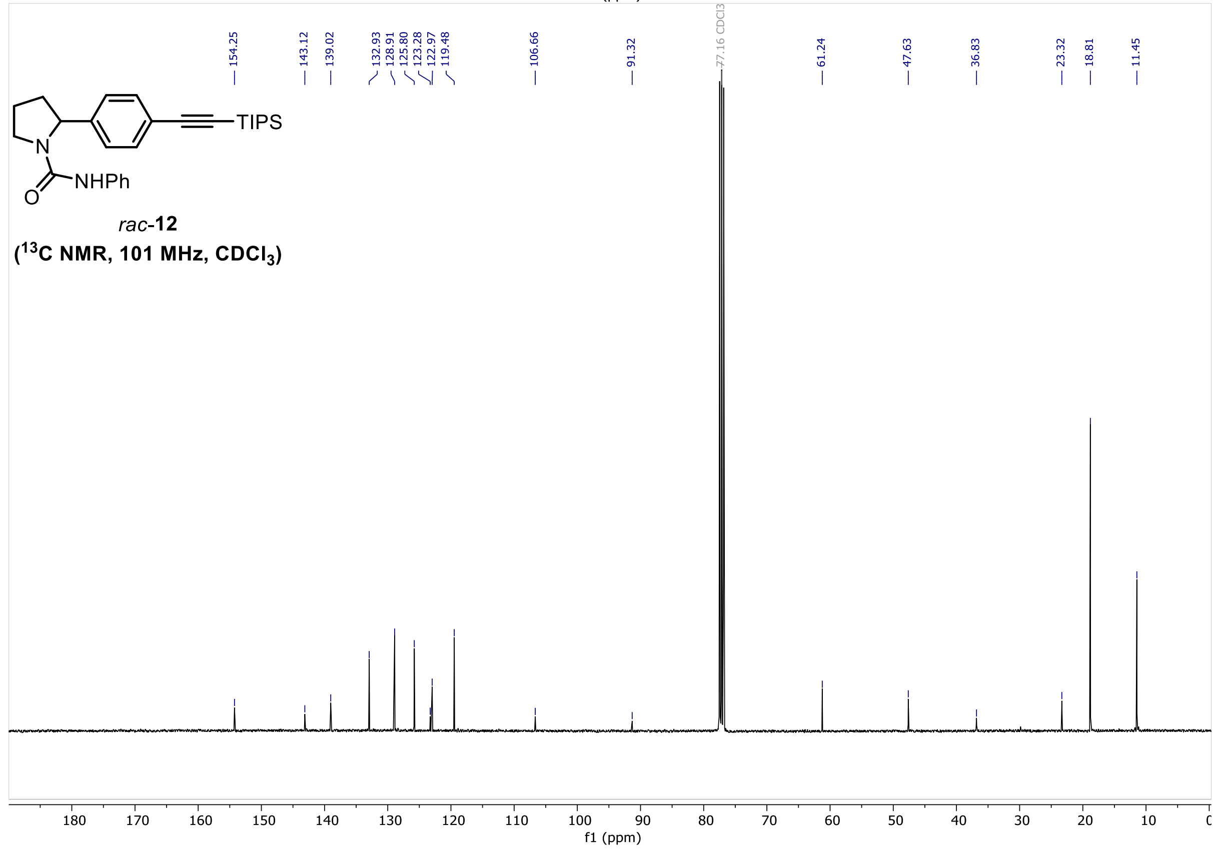

rac-**12**
(^{13}C NMR, 101 MHz, CDCl$_3$)

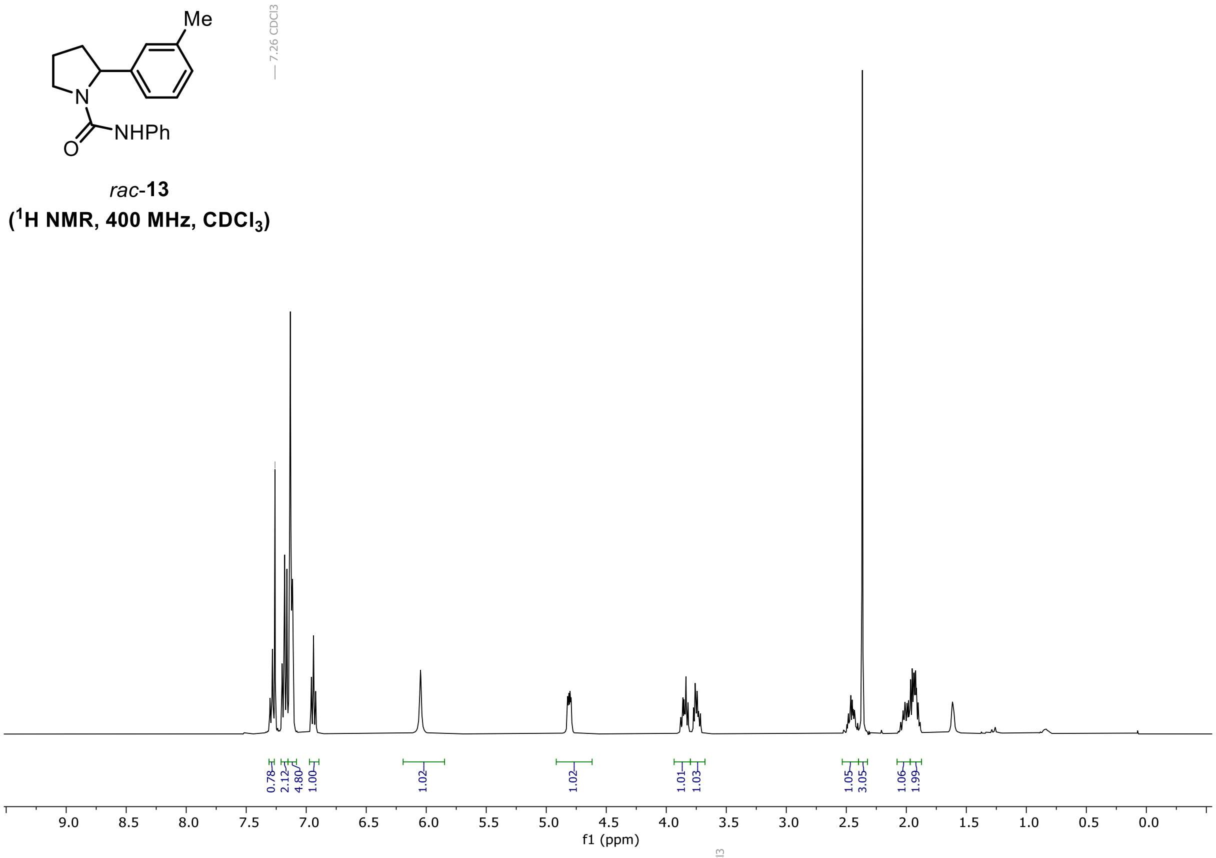

rac-**13**
(^{1}H NMR, 400 MHz, CDCl$_3$)

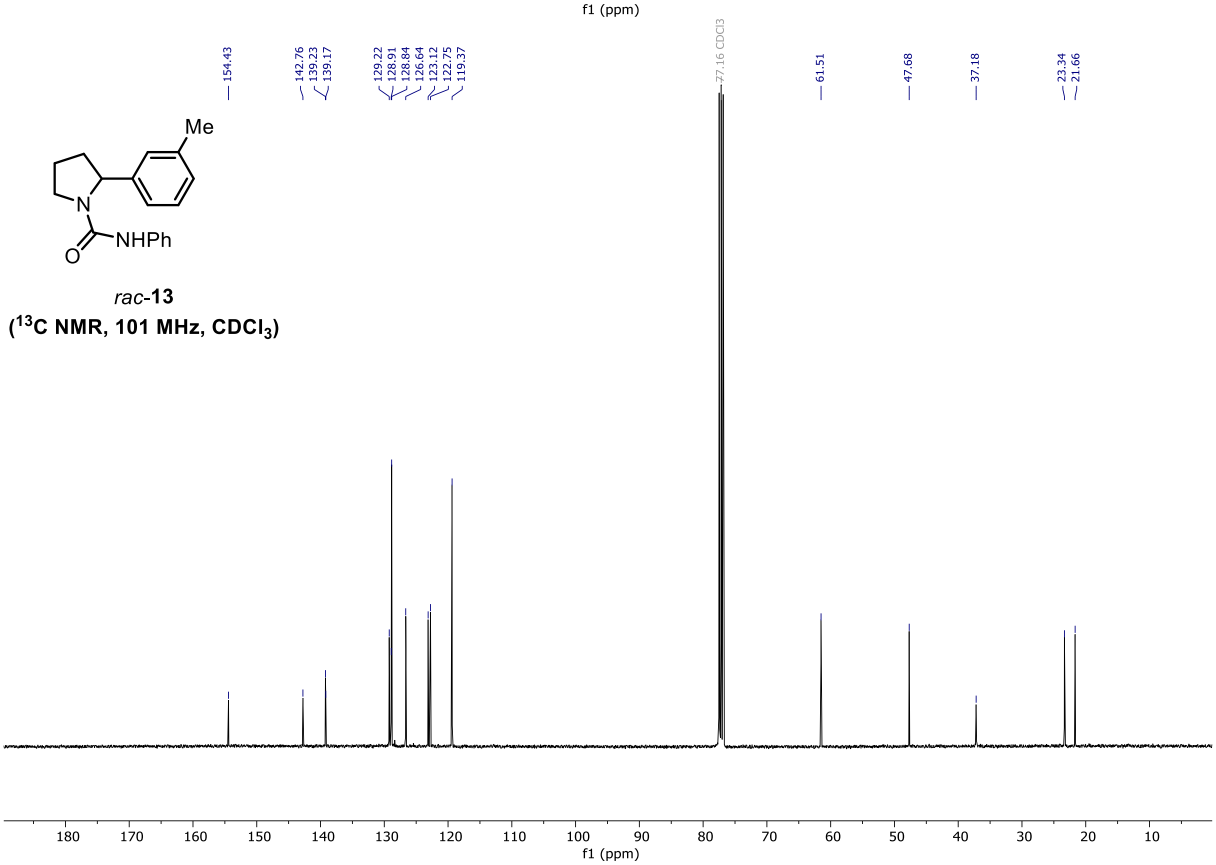

rac-**13**
(^{13}C NMR, 101 MHz, CDCl$_3$)

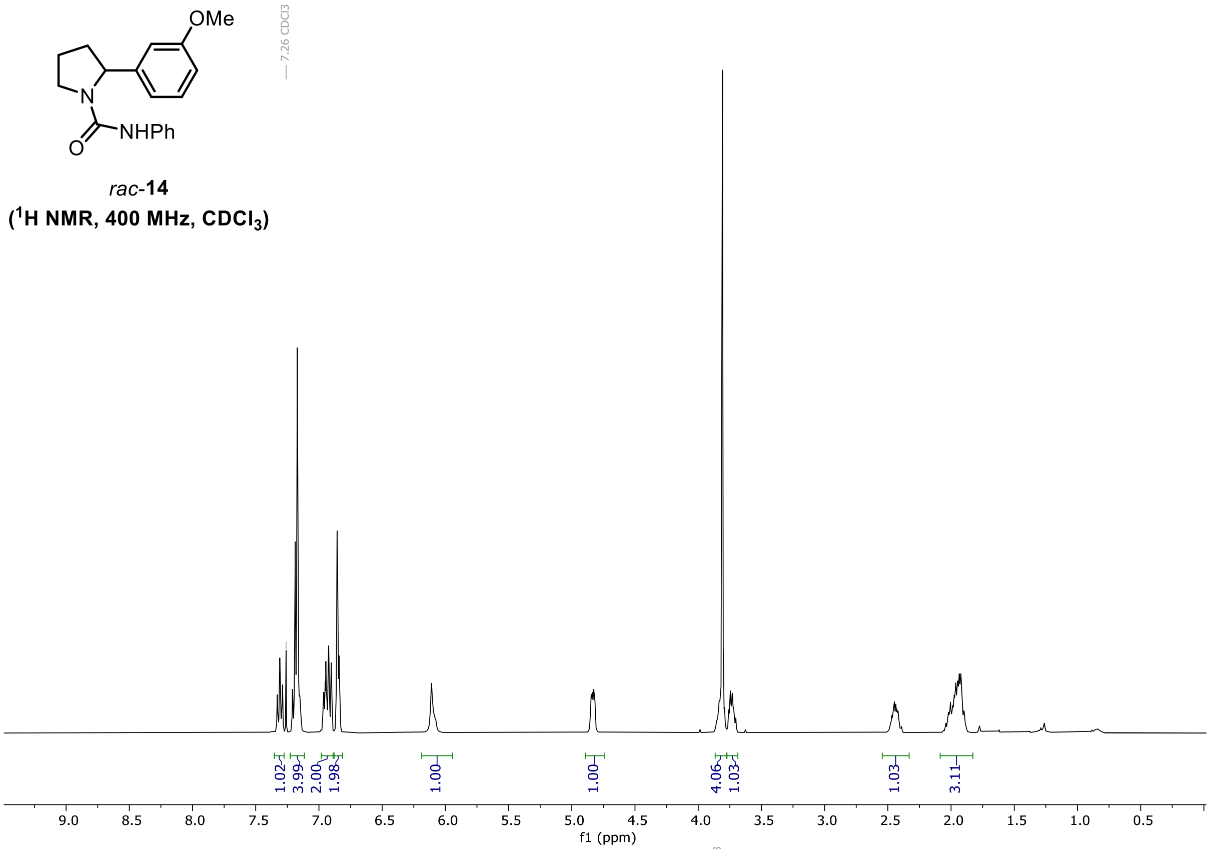
OMe
NHPh
O
N
rac-14
(1H NMR, 400 MHz, CDCl3)
7.26 CDCl3
1.02
3.99
2.00
1.98
1.00
1.00
4.06
1.03
1.03
3.11
f1 (ppm)

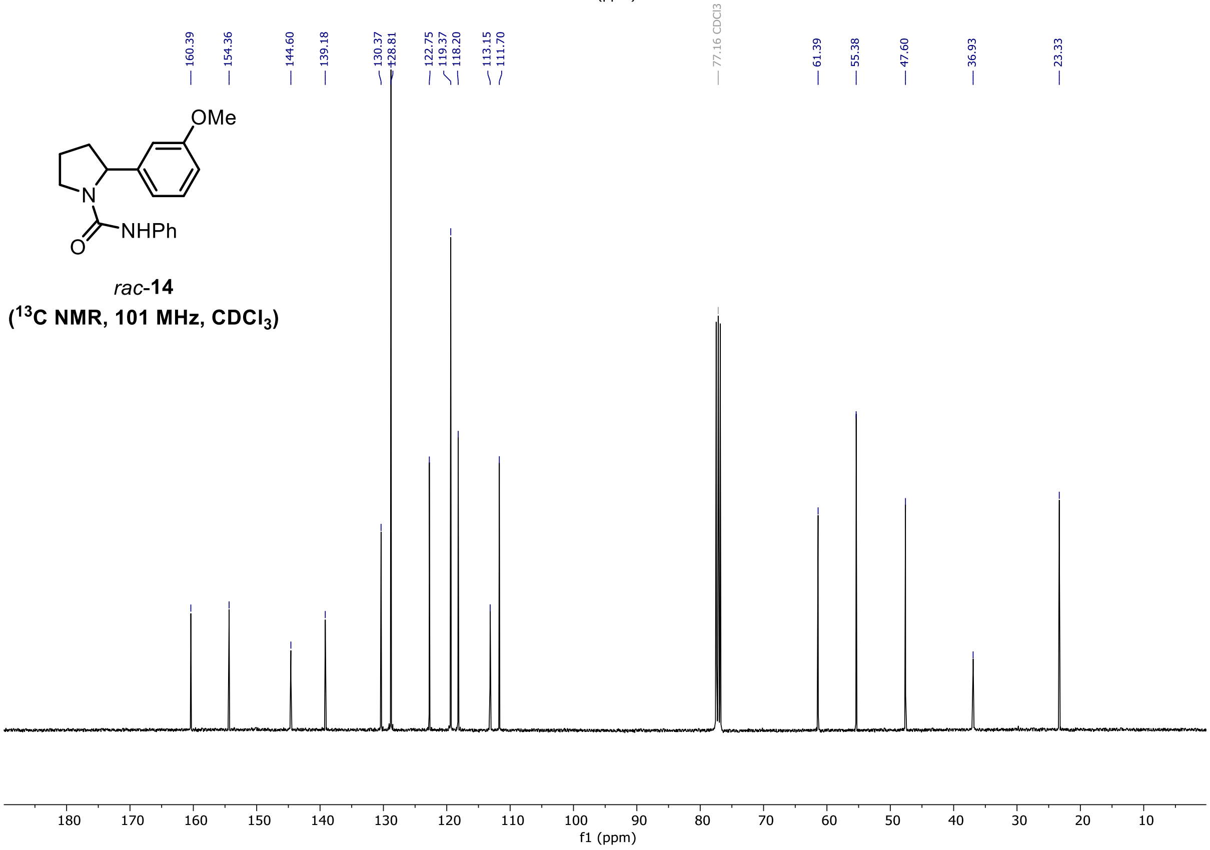
OMe
NHPh
O
N
rac-14
(13C NMR, 101 MHz, CDCl3)
160.39
154.36
144.60
139.18
130.37
128.81
122.75
119.37
118.20
113.15
111.70
77.16 CDCl3
61.39
55.38
47.60
36.93
23.33
f1 (ppm)

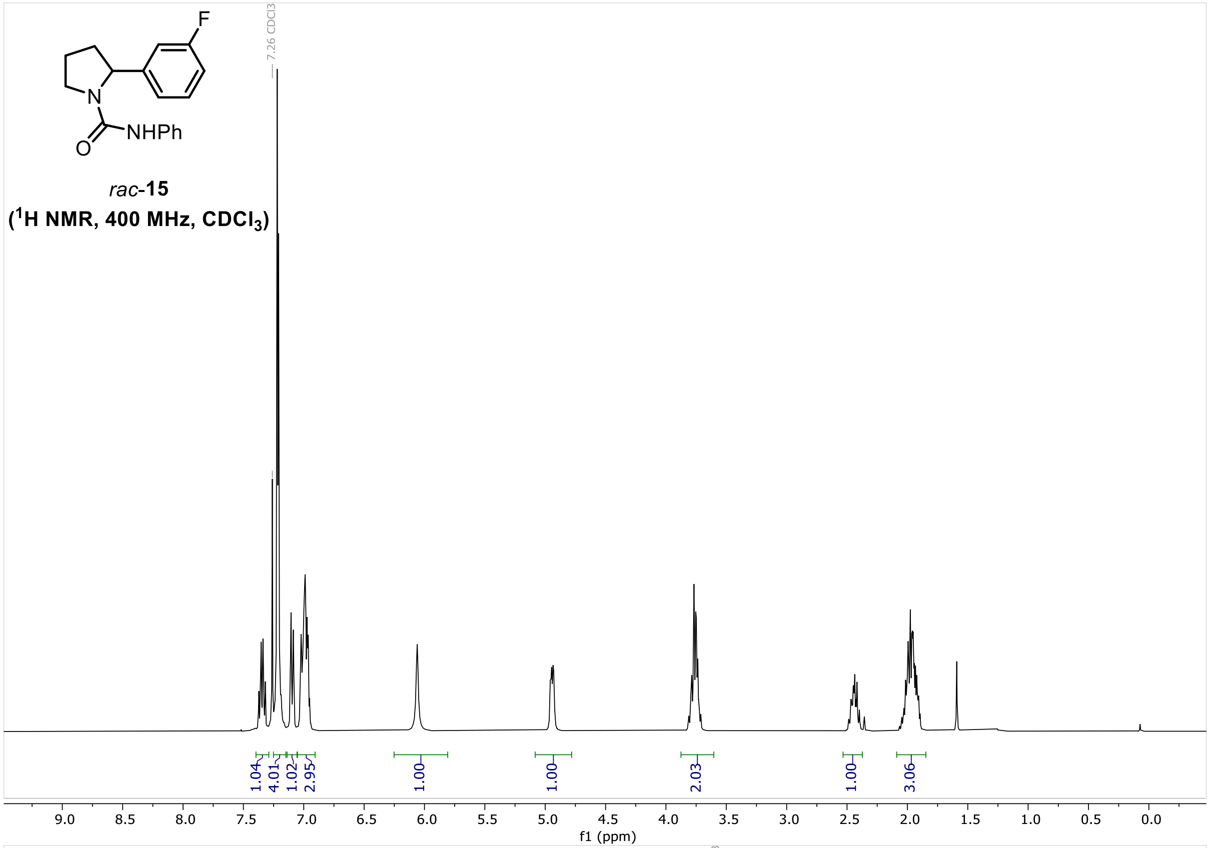

F
N
O
NHPh
rac-15
(1H NMR, 400 MHz, CDCl3)
7.26 CDCl3
1.04
4.01
1.02
2.95
1.00
1.00
2.03
1.00
3.06
f1 (ppm)

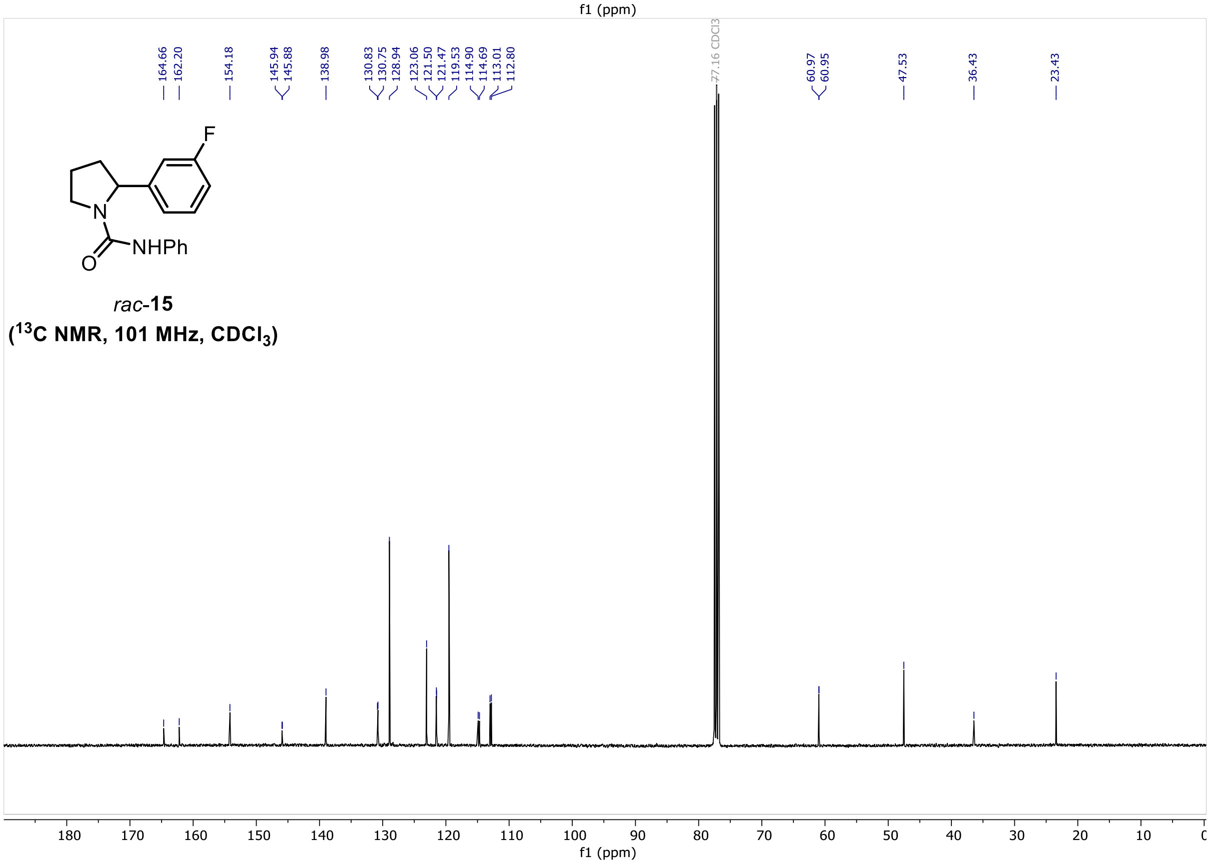

164.66
162.20
154.18
145.94
145.88
138.98
130.83
130.75
128.94
123.06
121.50
121.47
119.53
114.90
114.69
113.01
112.80
77.16 CDCl3
60.97
60.95
47.53
36.43
23.43
F
N
O
NHPh
rac-15
(13C NMR, 101 MHz, CDCl3)
f1 (ppm)

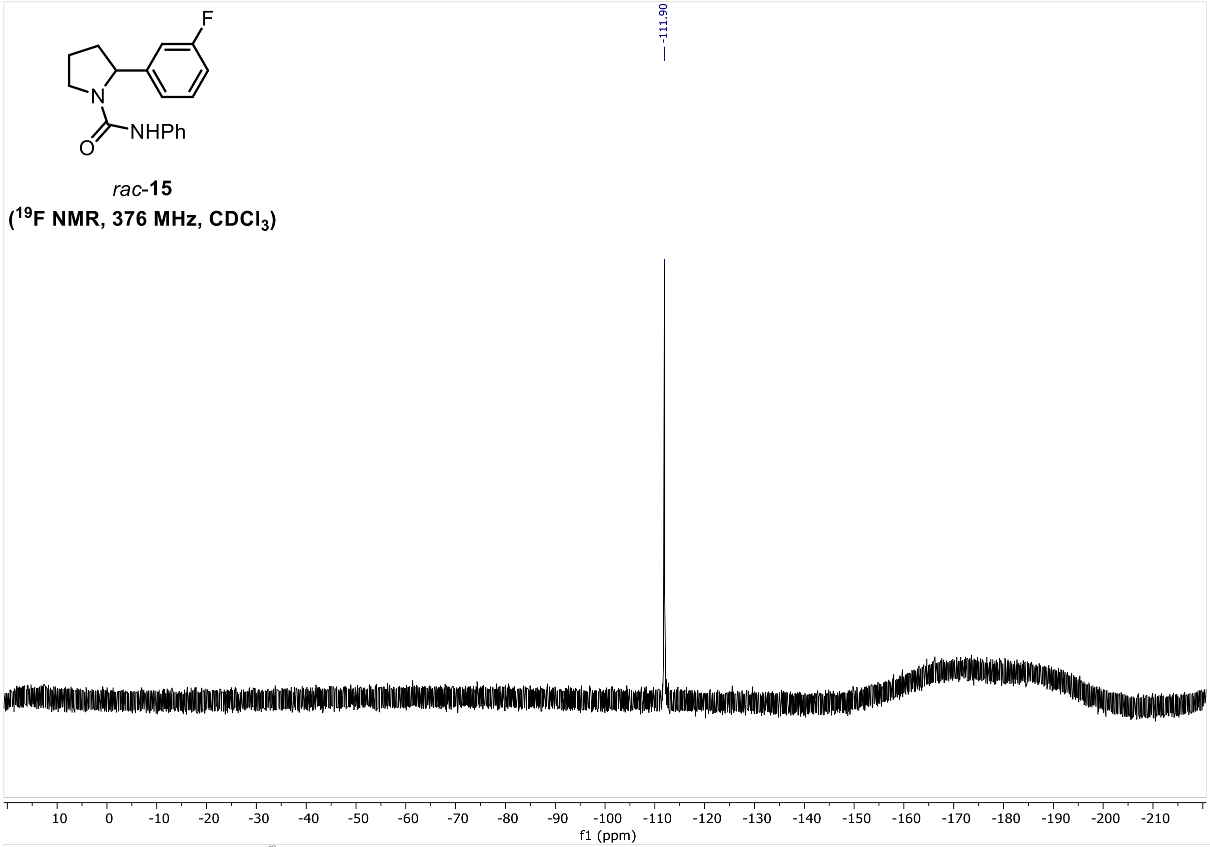
-111.90
F
NHPh
N
O
rac-15
(^{19}F NMR, 376 MHz, CDCl$_3$)
10 0 -10 -20 -30 -40 -50 -60 -70 -80 -90 -100 -110 -120 -130 -140 -150 -160 -170 -180 -190 -200 -210
f1 (ppm)

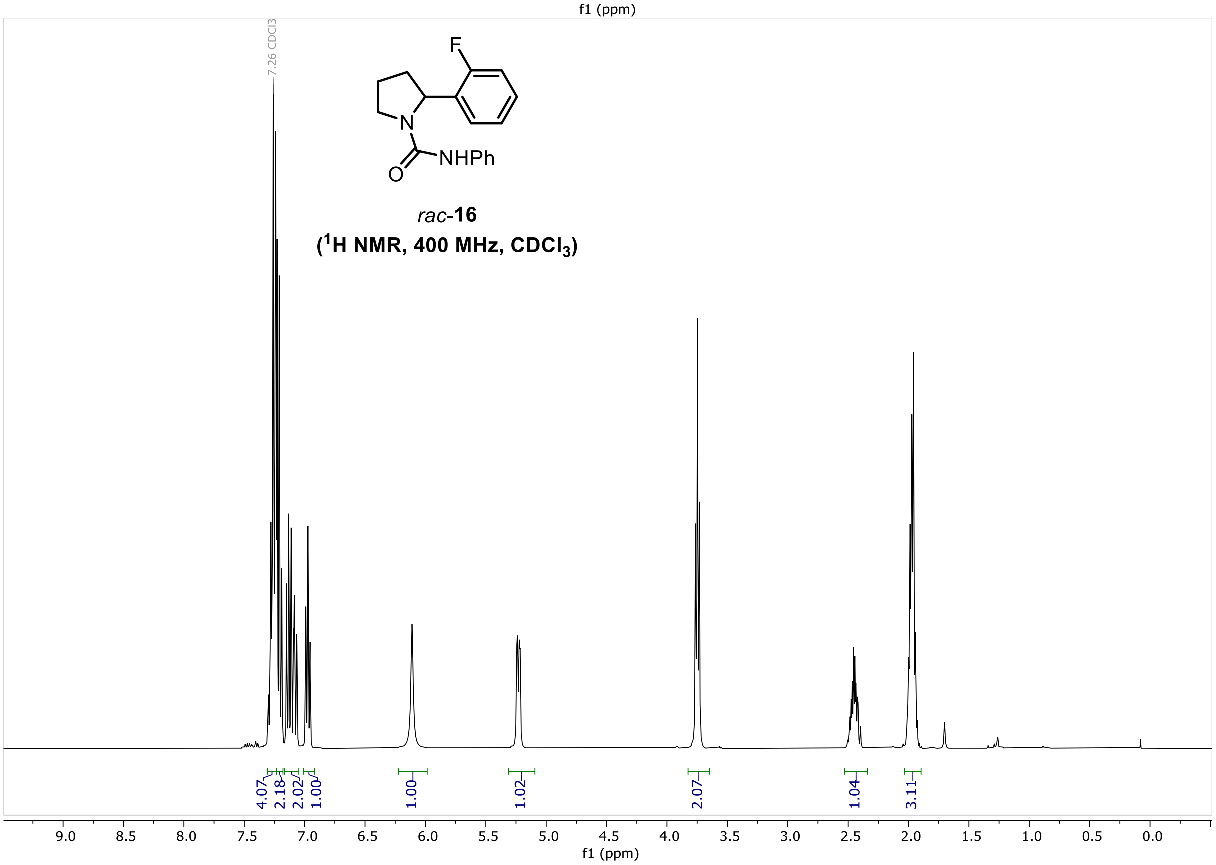
7.26 CDCl3
F
N
NHPh
O
rac-16
(^{1}H NMR, 400 MHz, CDCl$_3$)
4.07
2.18
2.02
1.00
1.00
1.02
2.07
1.04
3.11
9.0 8.5 8.0 7.5 7.0 6.5 6.0 5.5 5.0 4.5 4.0 3.5 3.0 2.5 2.0 1.5 1.0 0.5 0.0
f1 (ppm)

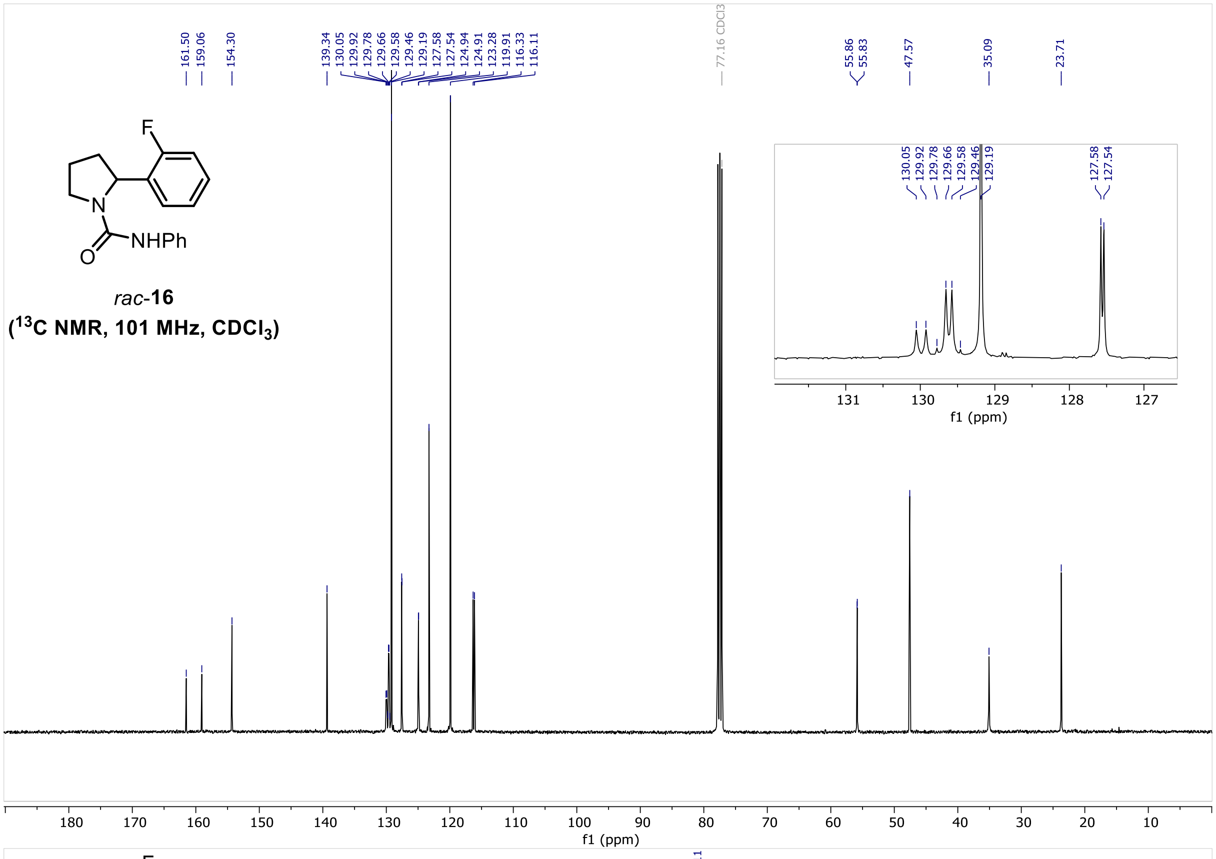

rac-**16**
(^{13}C NMR, 101 MHz, CDCl$_3$)

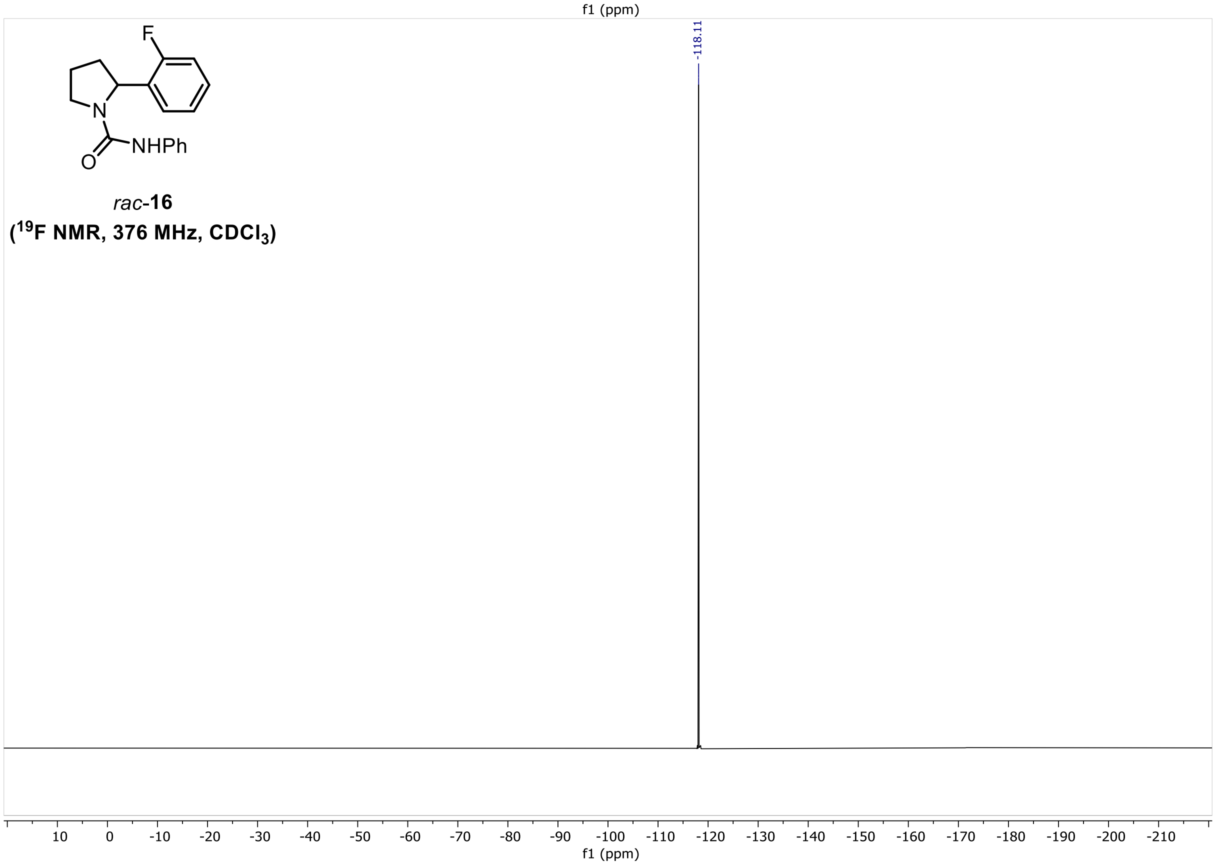

rac-**16**
(^{19}F NMR, 376 MHz, CDCl$_3$)

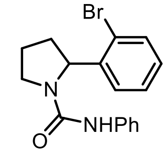

rac-**17**
(^{1}H NMR, 400 MHz, $CDCl_3$)

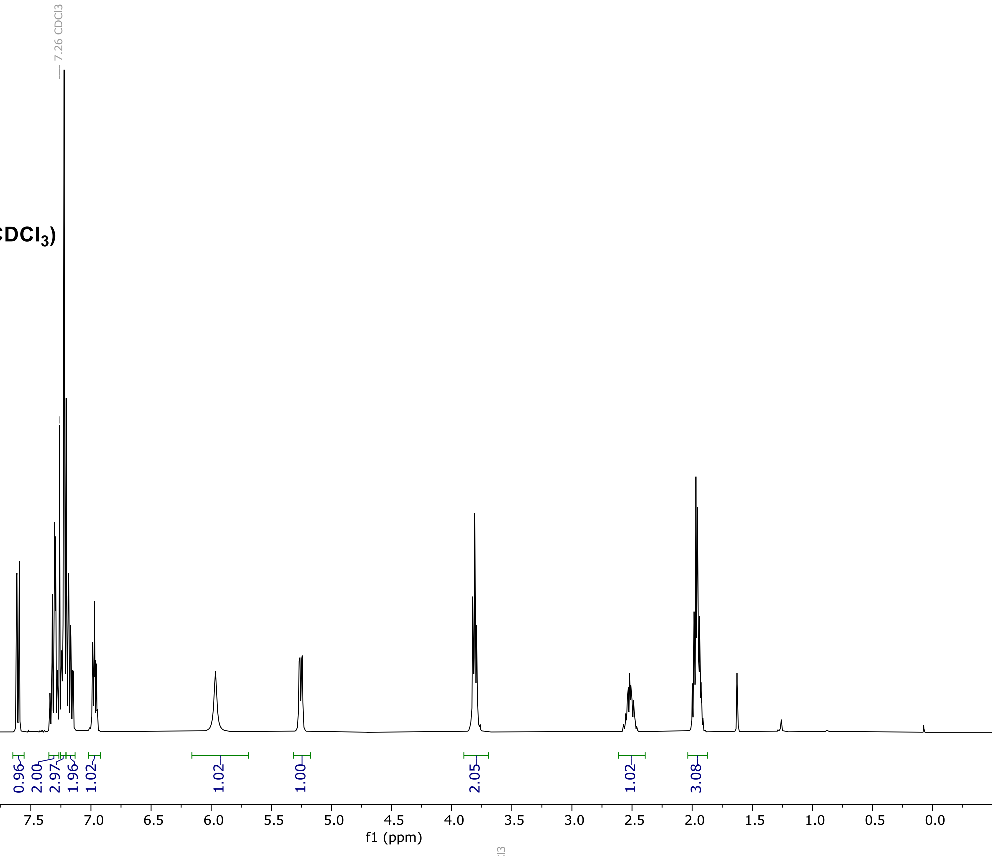

rac-**17**
(^{13}C NMR, 101 MHz, $CDCl_3$)

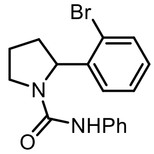

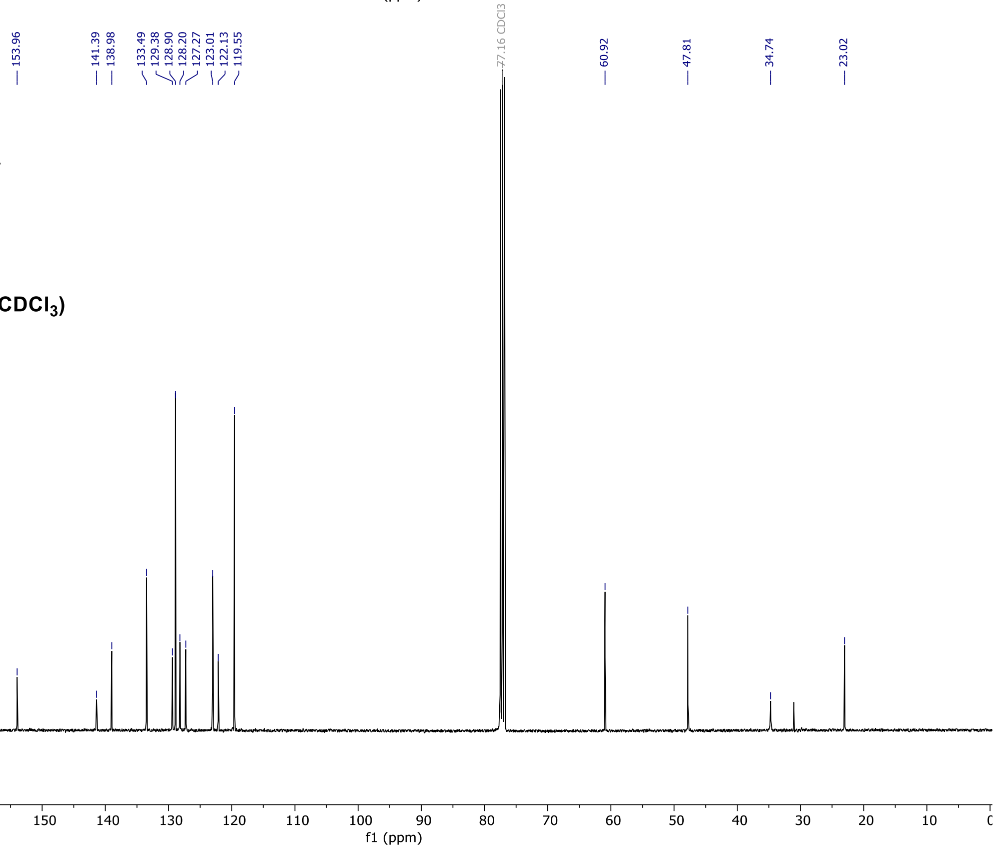

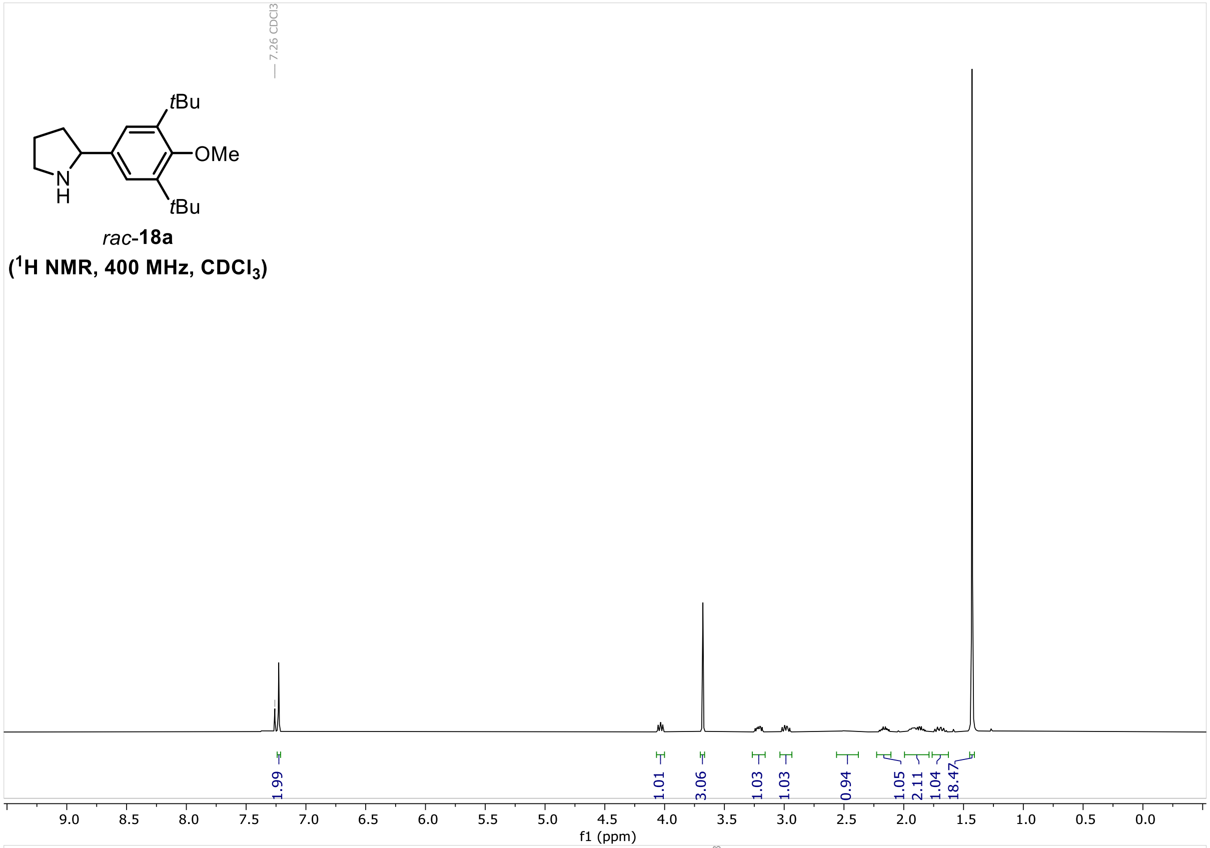
7.26 CDCl3
tBu
OMe
N
H
tBu
rac-18a
(1H NMR, 400 MHz, CDCl3)
1.99
1.01
3.06
1.03
1.03
0.94
1.05
2.11
1.04
18.47
9.0 8.5 8.0 7.5 7.0 6.5 6.0 5.5 5.0 4.5 4.0 3.5 3.0 2.5 2.0 1.5 1.0 0.5 0.0
f1 (ppm)

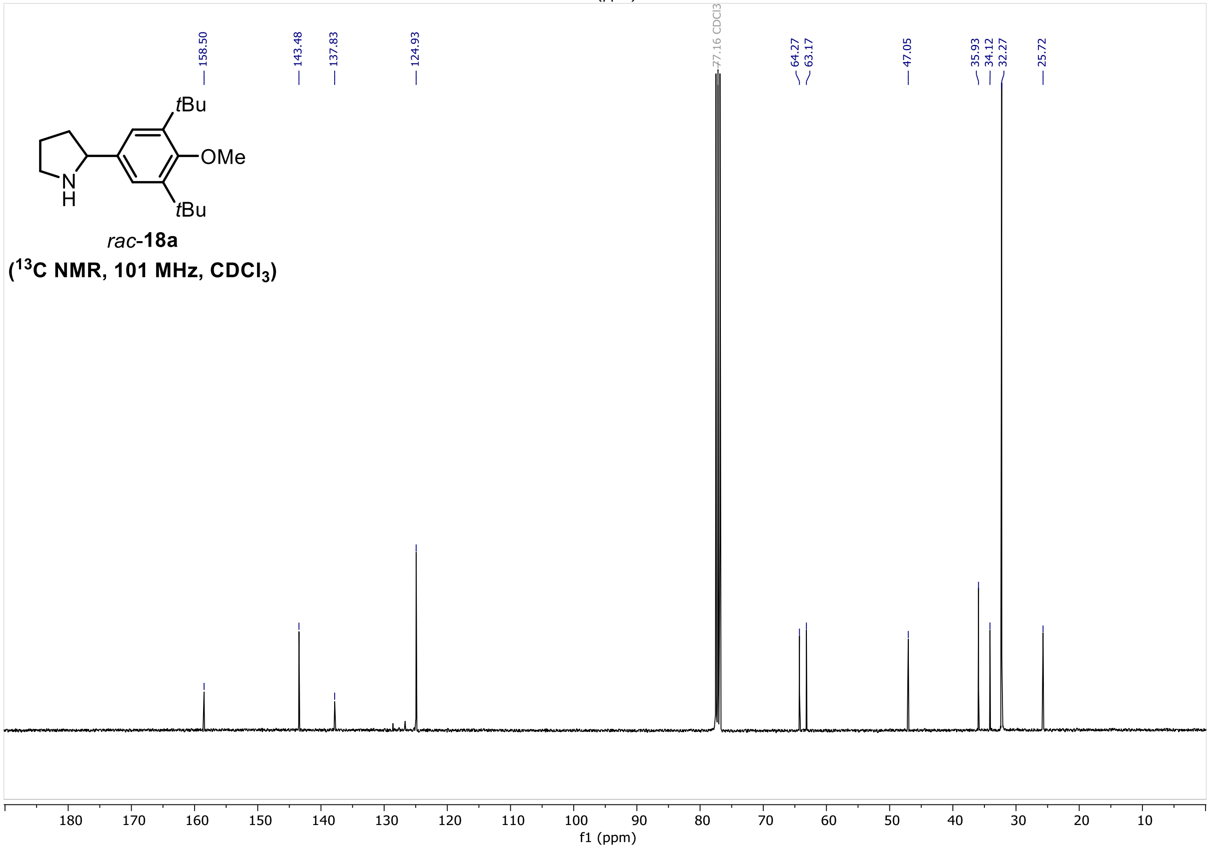
158.50
143.48
137.83
124.93
77.16 CDCl3
64.27
63.17
47.05
35.93
34.12
32.27
25.72
tBu
OMe
N
H
tBu
rac-18a
(13C NMR, 101 MHz, CDCl3)
180 170 160 150 140 130 120 110 100 90 80 70 60 50 40 30 20 10
f1 (ppm)

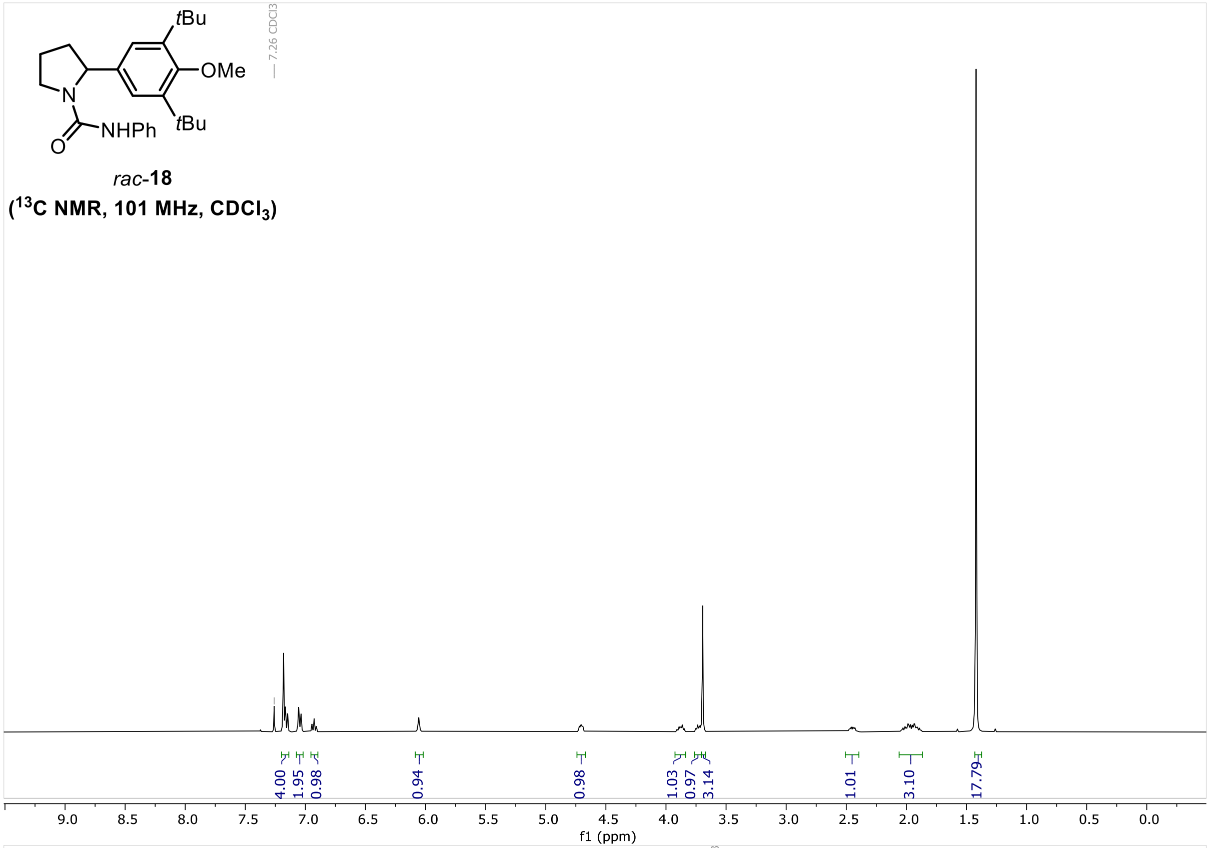

rac-**18**
(^{13}C NMR, 101 MHz, CDCl$_3$)

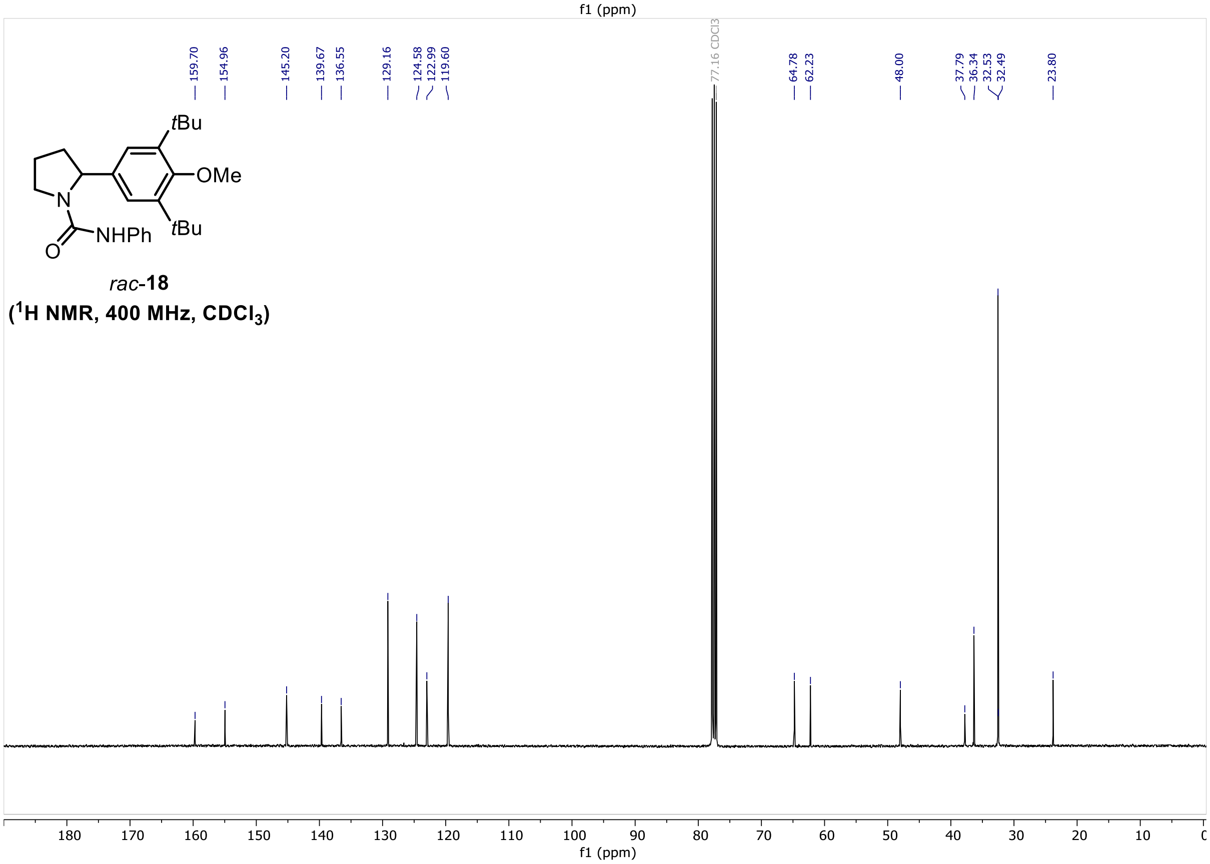

rac-**18**
(^{1}H NMR, 400 MHz, CDCl$_3$)

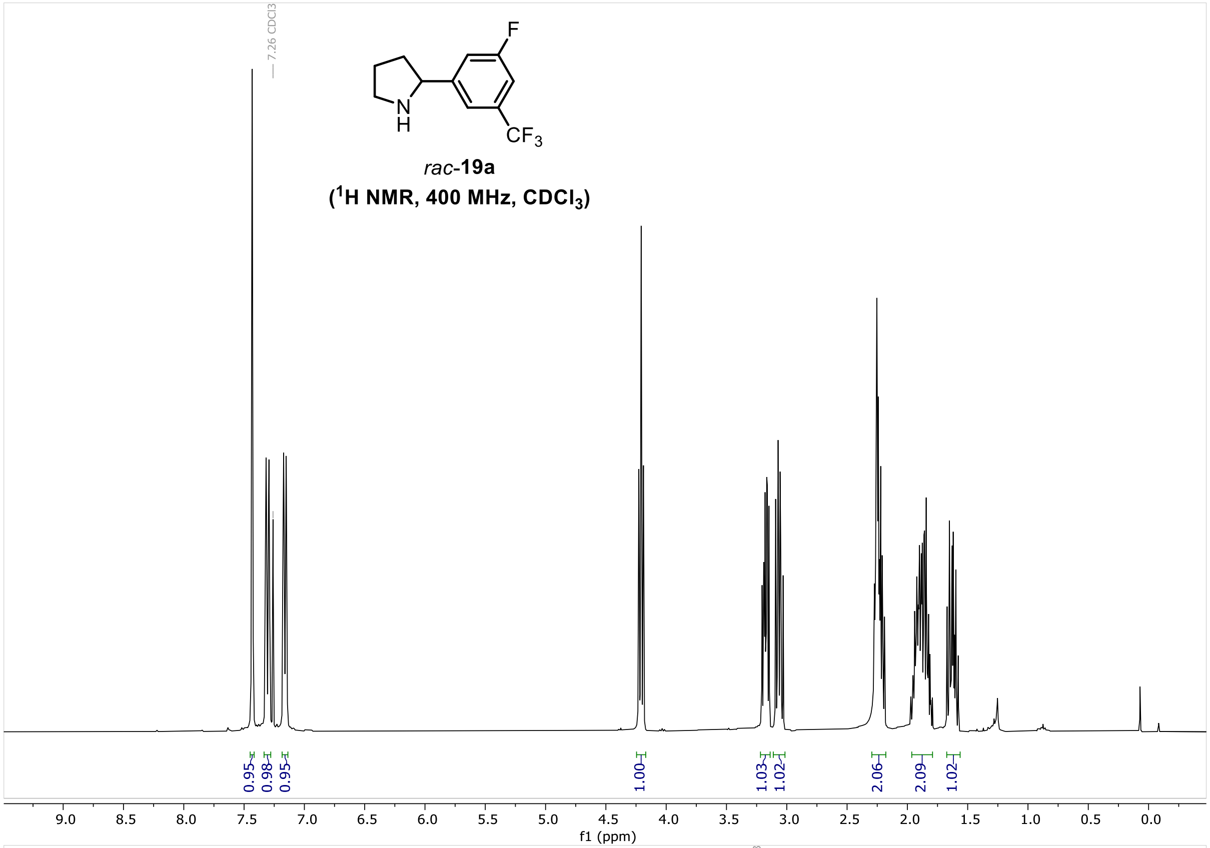
7.26 CDCl3
rac-19a
(1H NMR, 400 MHz, CDCl3)
0.95
0.98
0.95
1.00
1.03
1.02
2.06
2.09
1.02
9.0 8.5 8.0 7.5 7.0 6.5 6.0 5.5 5.0 4.5 4.0 3.5 3.0 2.5 2.0 1.5 1.0 0.5 0.0
f1 (ppm)

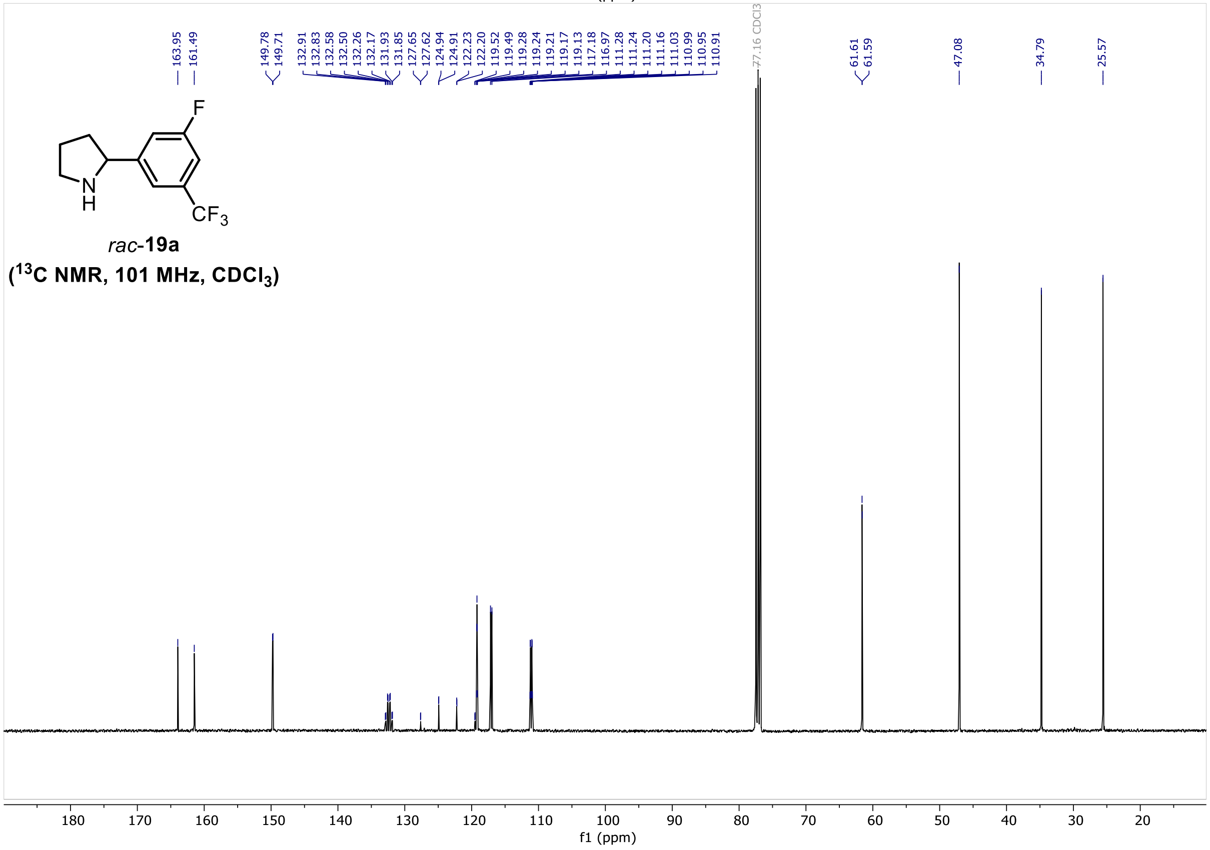
163.95
161.49
149.78
149.71
132.91
132.83
132.50
132.58
132.26
132.17
131.93
131.85
127.65
127.62
124.94
124.91
122.23
122.20
119.52
119.49
119.28
119.24
119.21
119.17
119.13
117.18
116.97
111.28
111.24
111.20
111.16
111.03
110.99
110.95
110.91
77.16 CDCl3
61.61
61.59
47.08
34.79
25.57
rac-19a
(13C NMR, 101 MHz, CDCl3)
180 170 160 150 140 130 120 110 100 90 80 70 60 50 40 30 20
f1 (ppm)

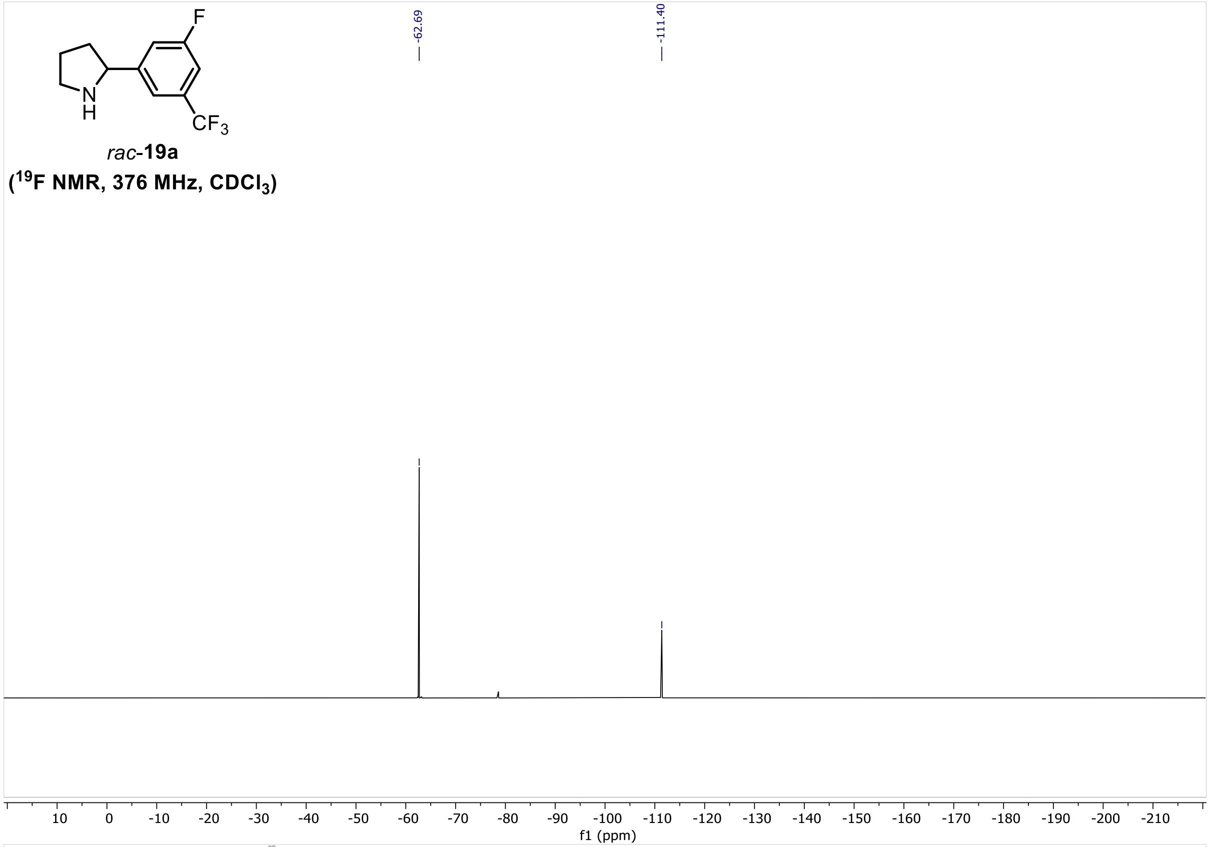

F
N
H
CF3
rac-19a
(19F NMR, 376 MHz, CDCl3)
-62.69
-111.40
10
0
-10
-20
-30
-40
-50
-60
-70
-80
-90
-100
-110
-120
-130
-140
-150
-160
-170
-180
-190
-200
-210
f1 (ppm)

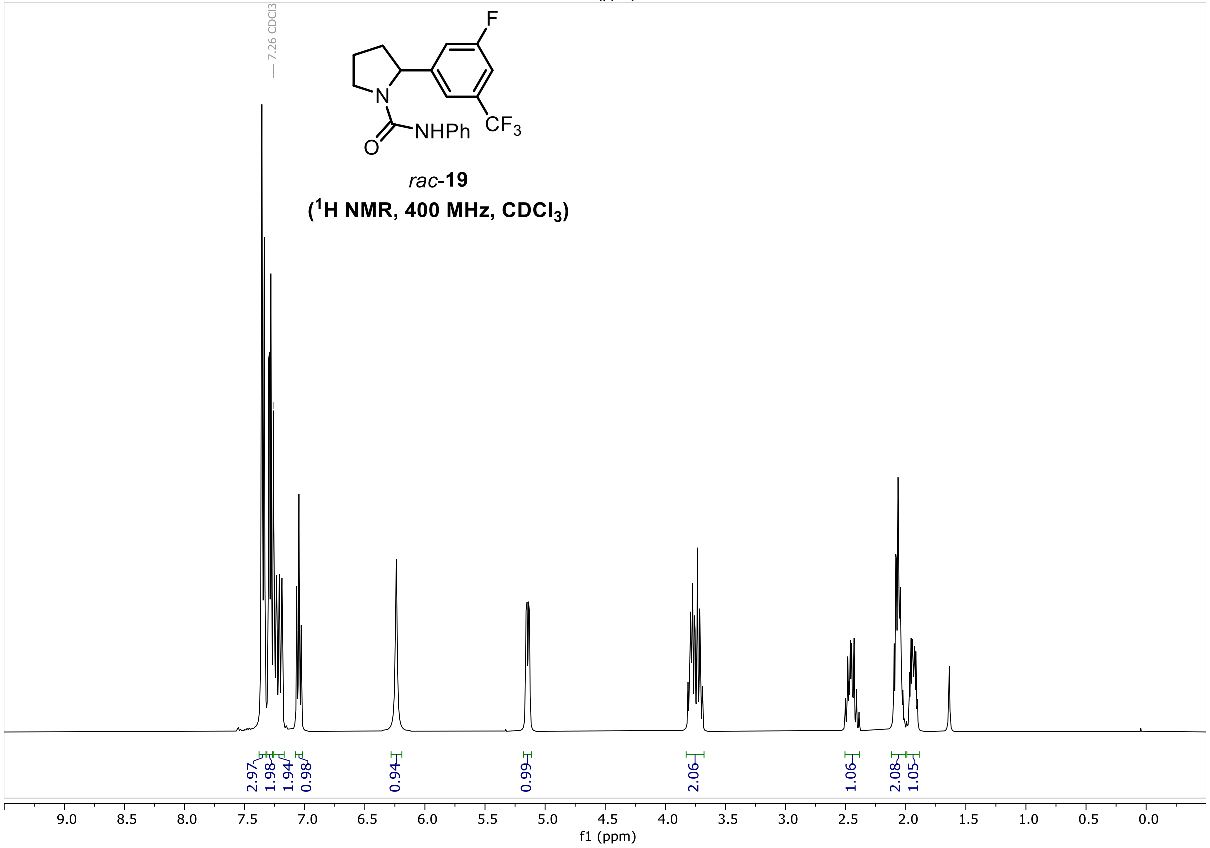

7.26 CDCl3
F
N
O
NHPh
CF3
rac-19
(1H NMR, 400 MHz, CDCl3)
2.97
1.98
1.94
0.98
0.94
0.99
2.06
1.06
2.08
1.05
9.0
8.5
8.0
7.5
7.0
6.5
6.0
5.5
5.0
4.5
4.0
3.5
3.0
2.5
2.0
1.5
1.0
0.5
0.0
f1 (ppm)

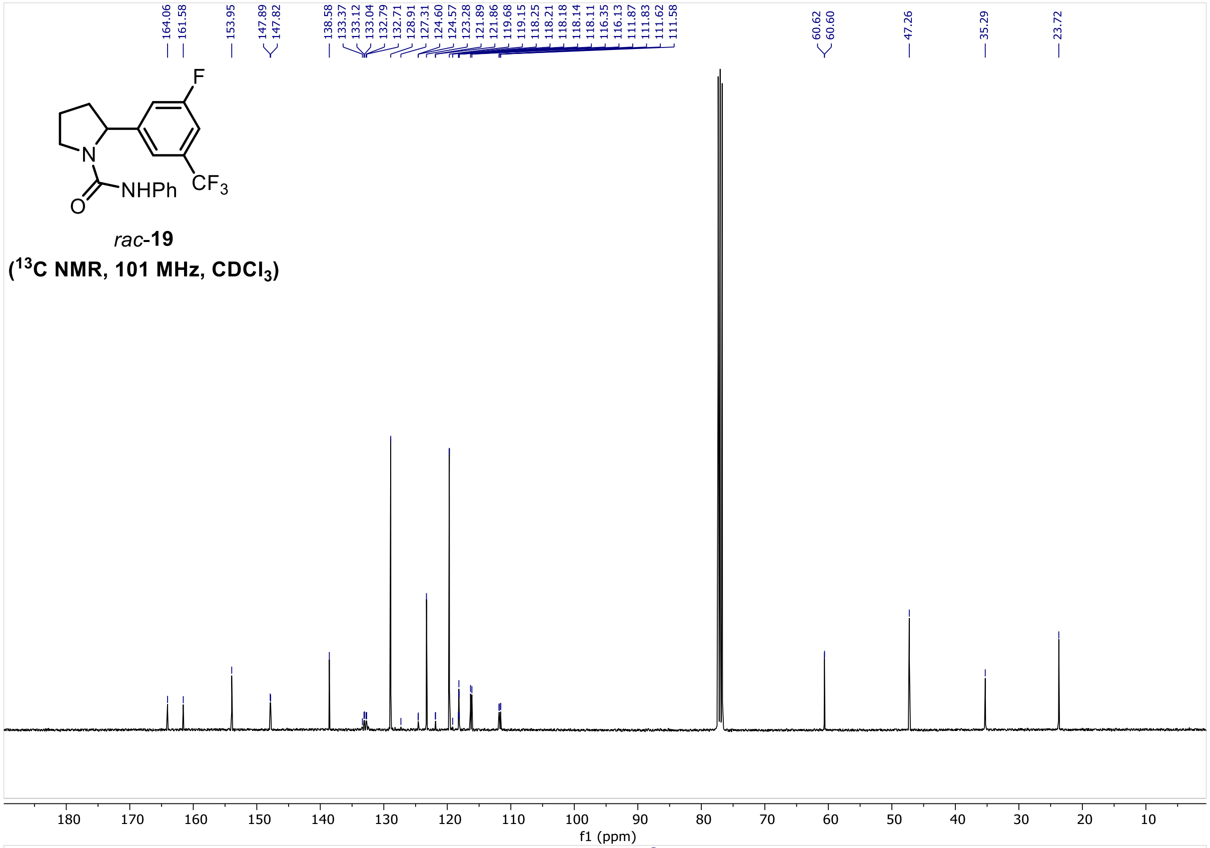

164.06
161.58
153.95
147.89
147.82
138.58
133.37
133.12
133.04
132.79
132.71
128.91
127.31
124.60
124.57
123.28
121.89
121.86
119.68
119.15
118.25
118.21
118.18
118.14
118.11
116.35
116.13
111.87
111.83
111.62
111.58
60.62
60.60
47.26
35.29
23.72
F
N
NHPh
CF3
O
rac-19
(13C NMR, 101 MHz, CDCl3)
180 170 160 150 140 130 120 110 100 90 80 70 60 50 40 30 20 10
f1 (ppm)

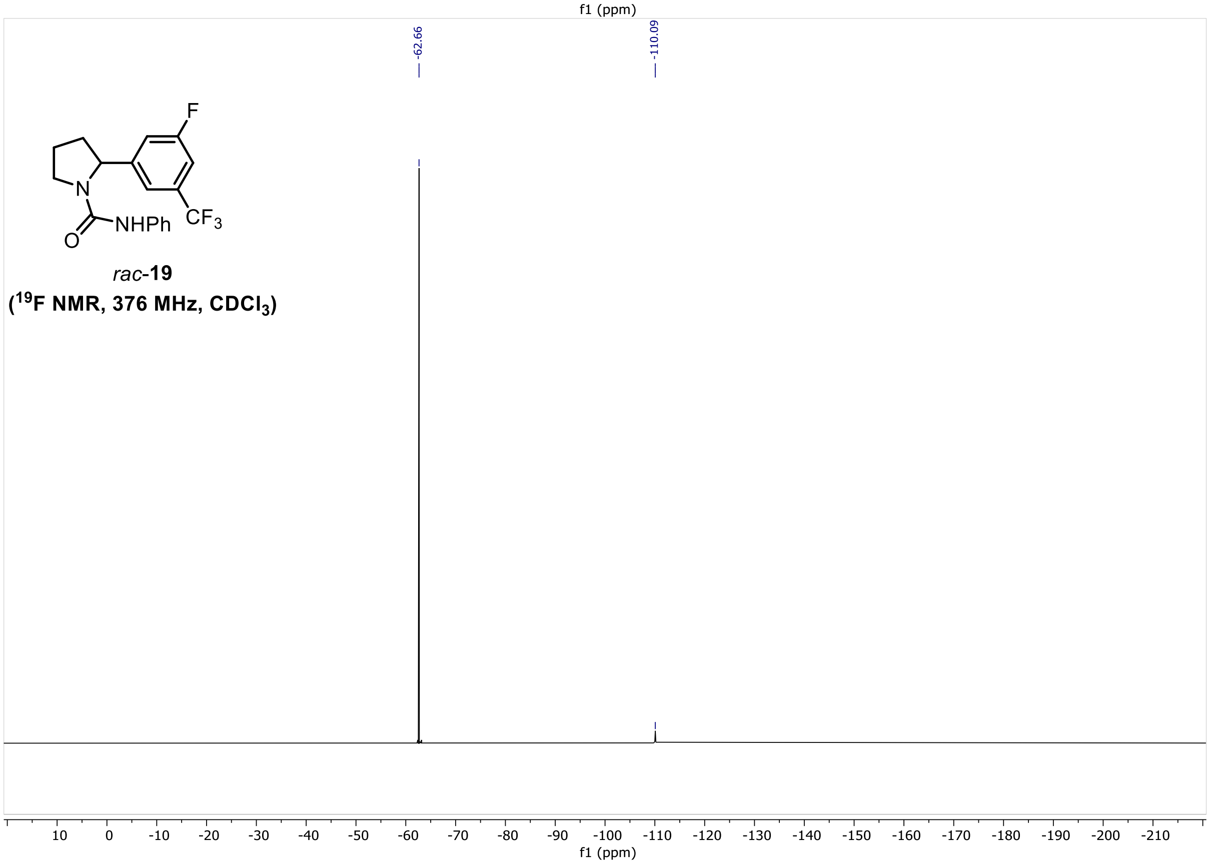

-62.66
-110.09
F
N
NHPh
CF3
O
rac-19
(19F NMR, 376 MHz, CDCl3)
10 0 -10 -20 -30 -40 -50 -60 -70 -80 -90 -100 -110 -120 -130 -140 -150 -160 -170 -180 -190 -200 -210
f1 (ppm)

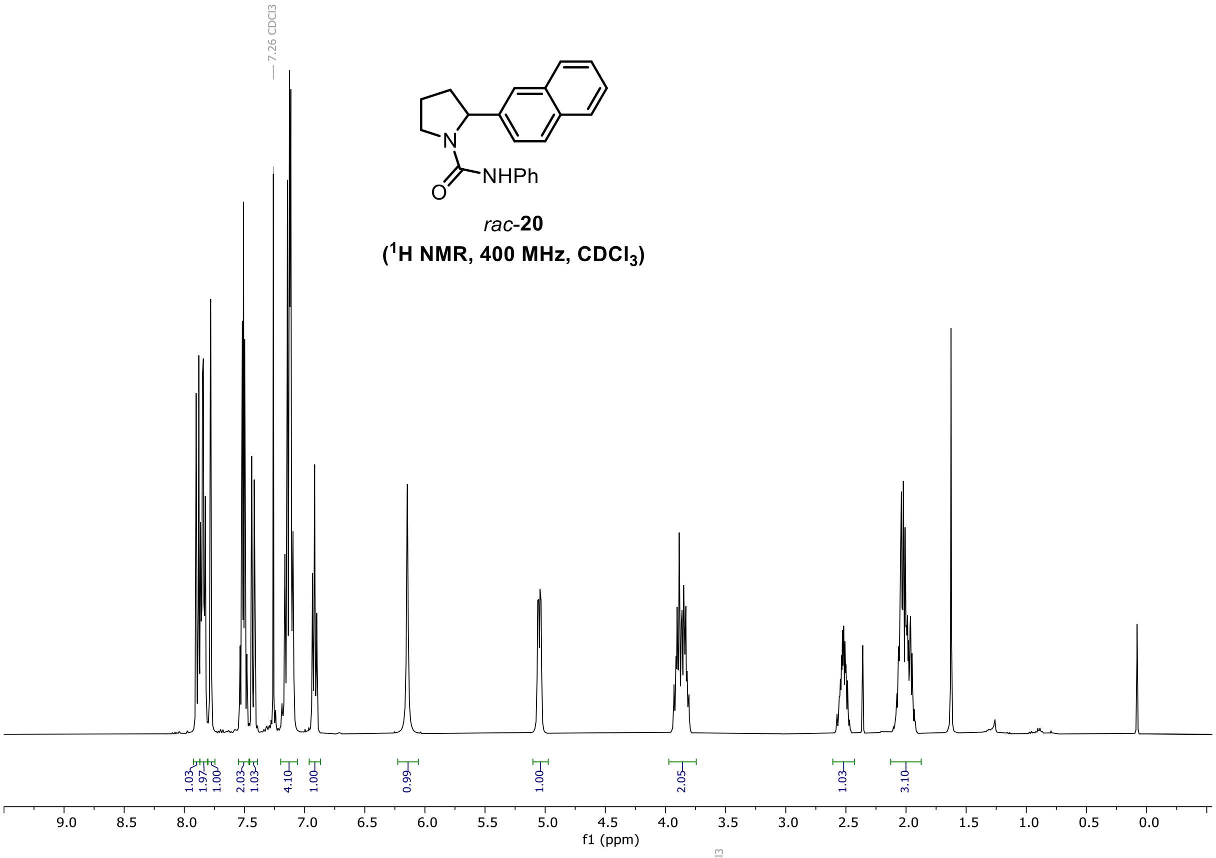
7.26 CDCl3
NHPh
N
O
rac-20
(1H NMR, 400 MHz, CDCl3)
1.03
1.97
1.00
2.03
1.03
4.10
1.00
0.99
1.00
2.05
1.03
3.10
9.0 8.5 8.0 7.5 7.0 6.5 6.0 5.5 5.0 4.5 4.0 3.5 3.0 2.5 2.0 1.5 1.0 0.5 0.0
f1 (ppm)

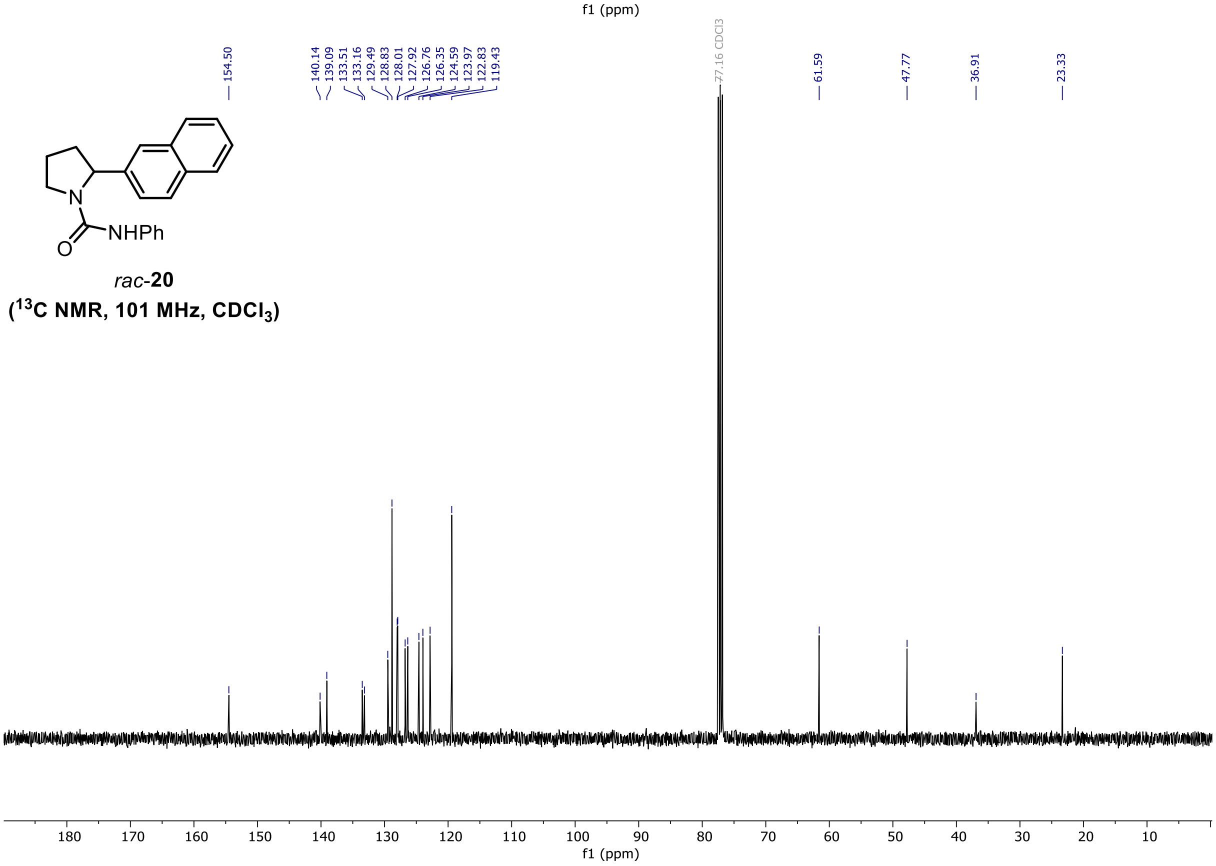
154.50
140.14
139.09
133.51
133.16
129.49
128.83
128.01
127.92
126.76
126.35
124.59
123.97
122.83
119.43
77.16 CDCl3
61.59
47.77
36.91
23.33
N
NHPh
O
rac-20
(13C NMR, 101 MHz, CDCl3)
180 170 160 150 140 130 120 110 100 90 80 70 60 50 40 30 20 10
f1 (ppm)

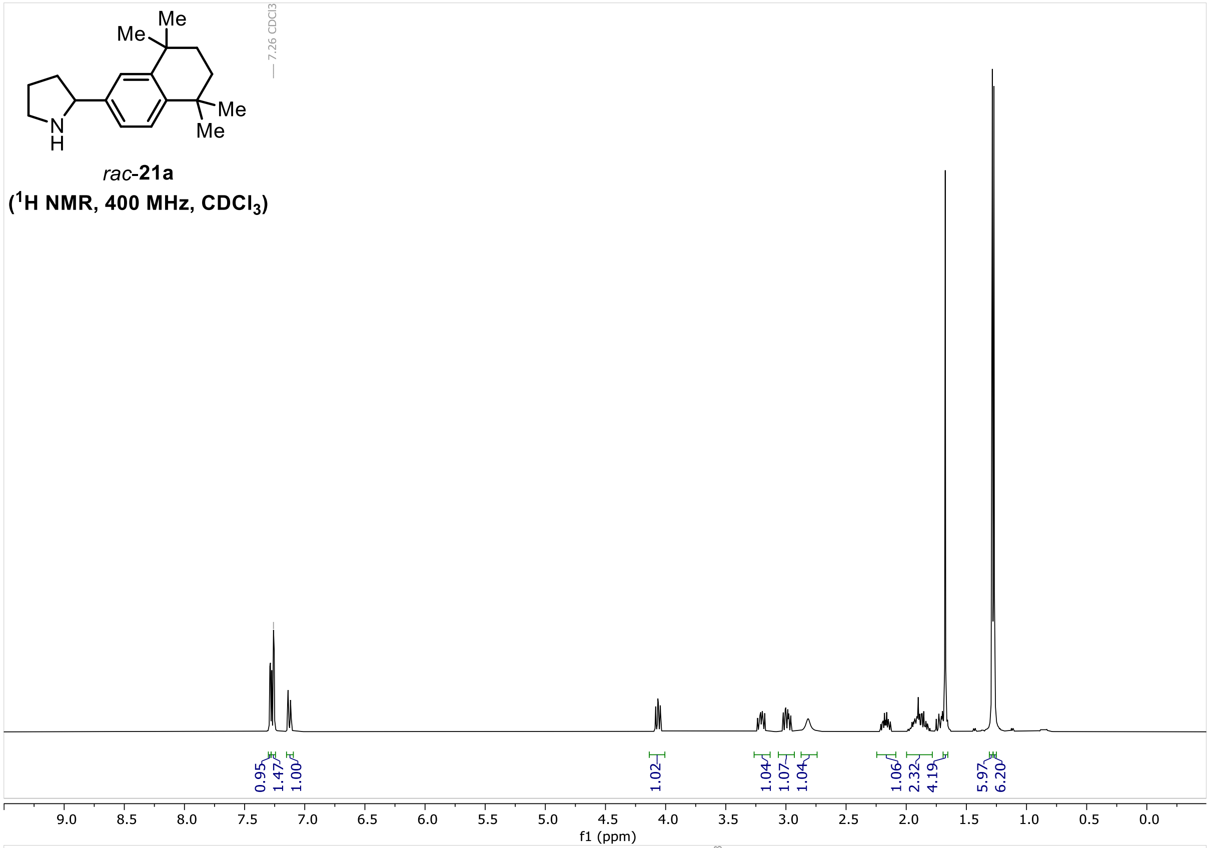

Me
Me
Me
Me
N
H
rac-21a
(1H NMR, 400 MHz, CDCl3)
7.26 CDCl3
0.95
1.47
1.00
1.02
1.04
1.07
1.04
1.06
2.32
4.19
5.97
6.20
f1 (ppm)

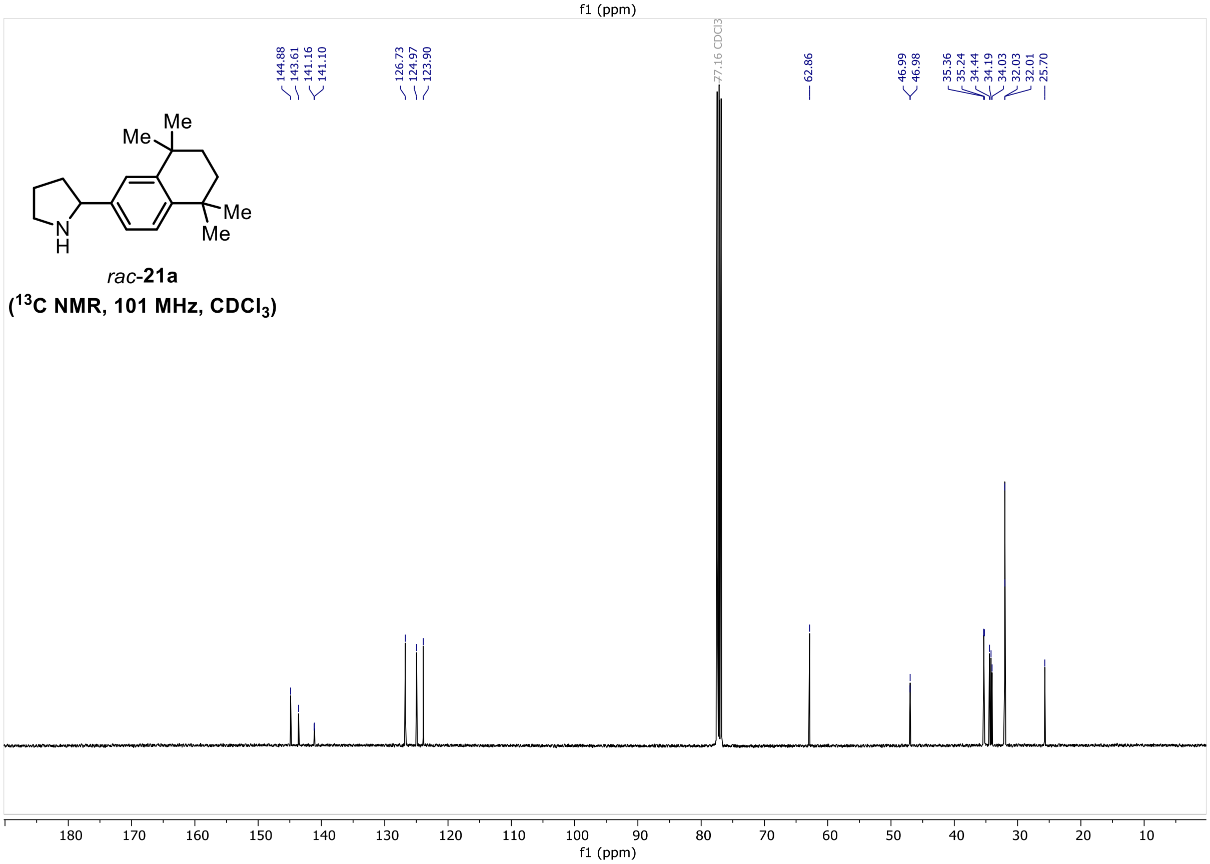

Me
Me
Me
Me
N
H
rac-21a
(13C NMR, 101 MHz, CDCl3)
144.88
143.61
141.16
141.10
126.73
124.97
123.90
77.16 CDCl3
62.86
46.99
46.98
35.36
35.24
34.44
34.19
34.03
32.03
32.01
25.70
f1 (ppm)

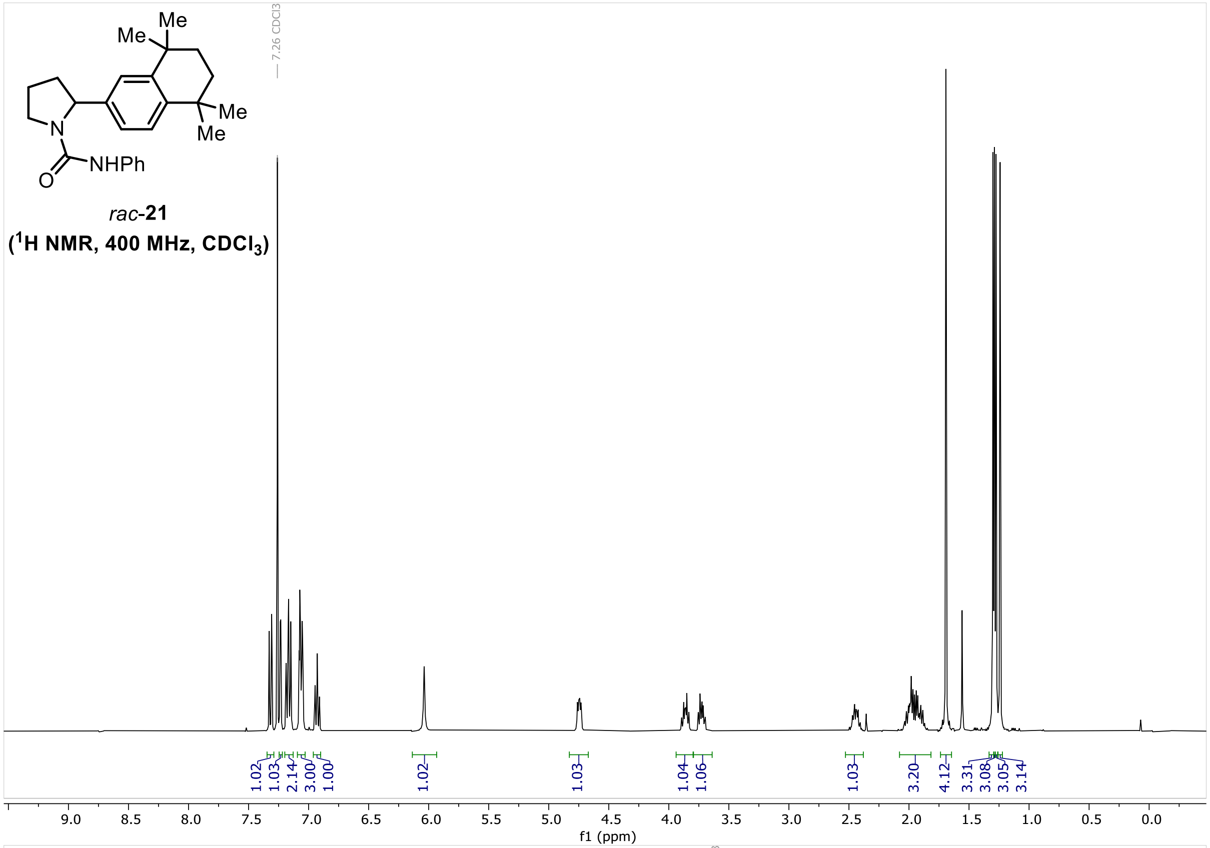

rac-**21**
(**^{1}H NMR, 400 MHz, CDCl$_3$**)

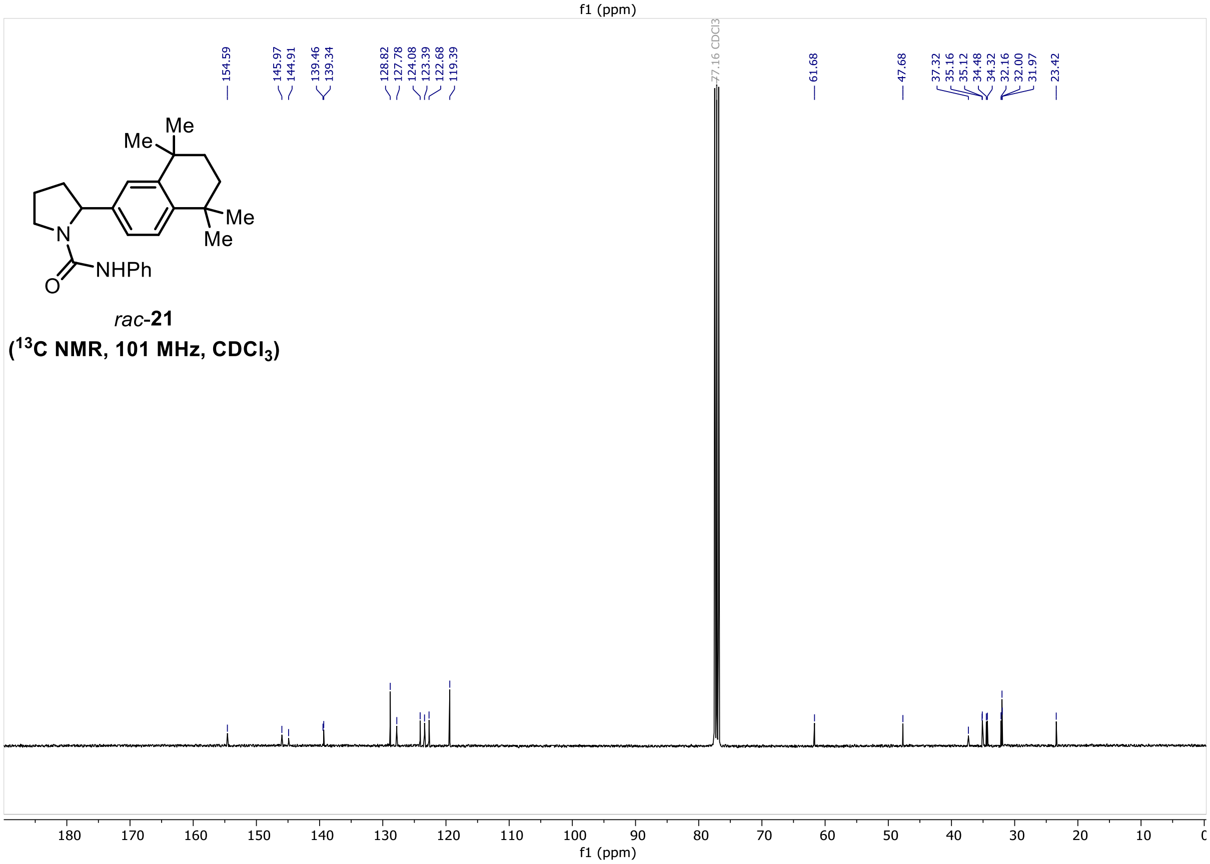

rac-**21**
(**^{13}C NMR, 101 MHz, CDCl$_3$**)

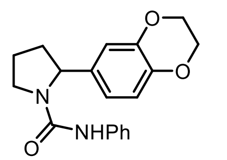

rac-**22**
(^{1}H NMR, 400 MHz, CDCl$_3$)

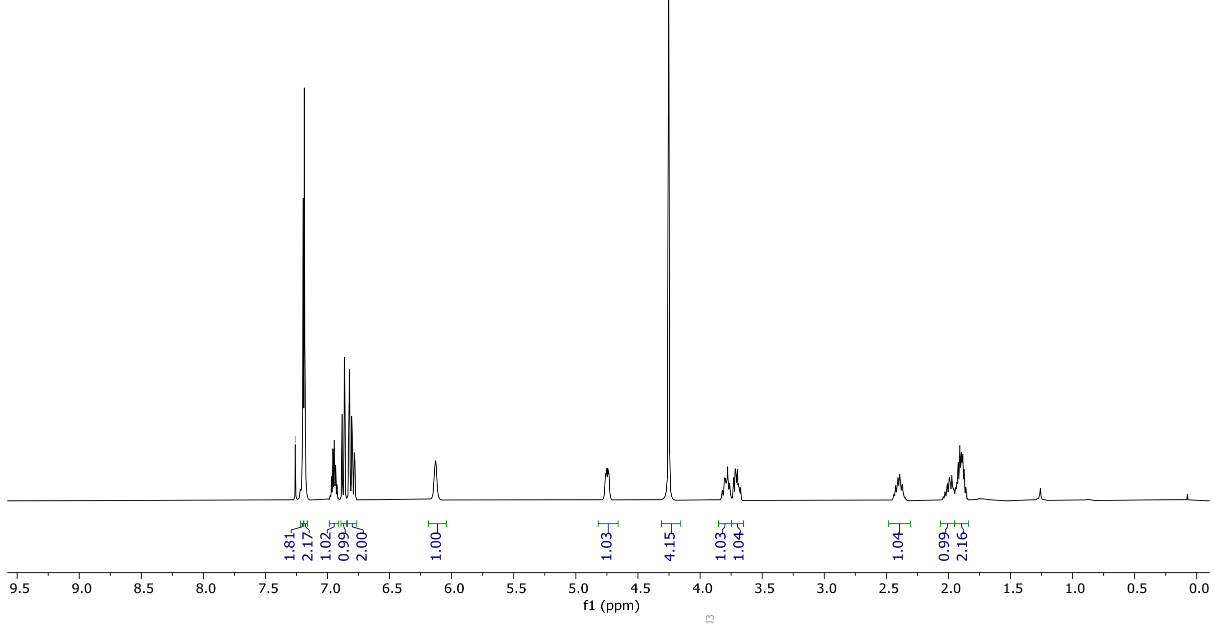

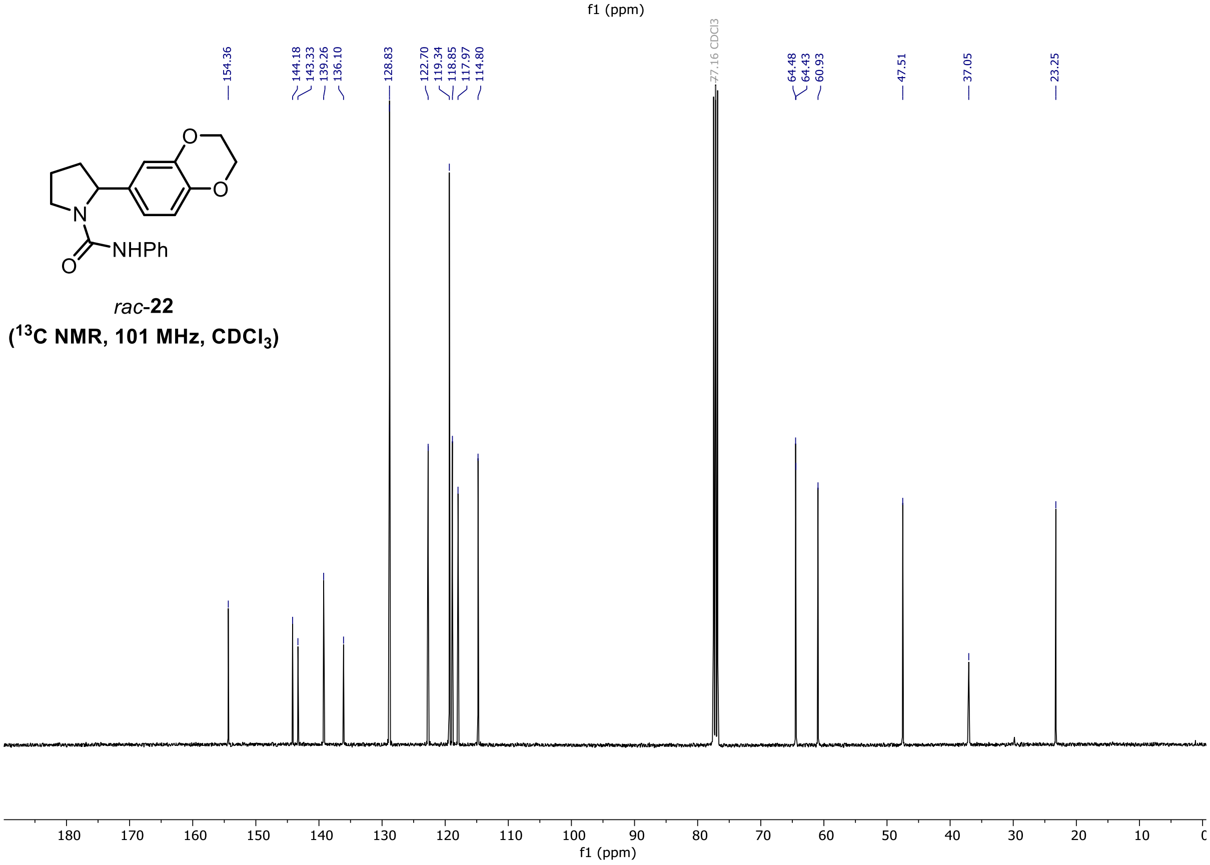

rac-**22**
(^{13}C NMR, 101 MHz, CDCl$_3$)

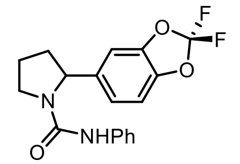

rac-**23**
(^{1}H NMR, 400 MHz, CDCl$_3$)

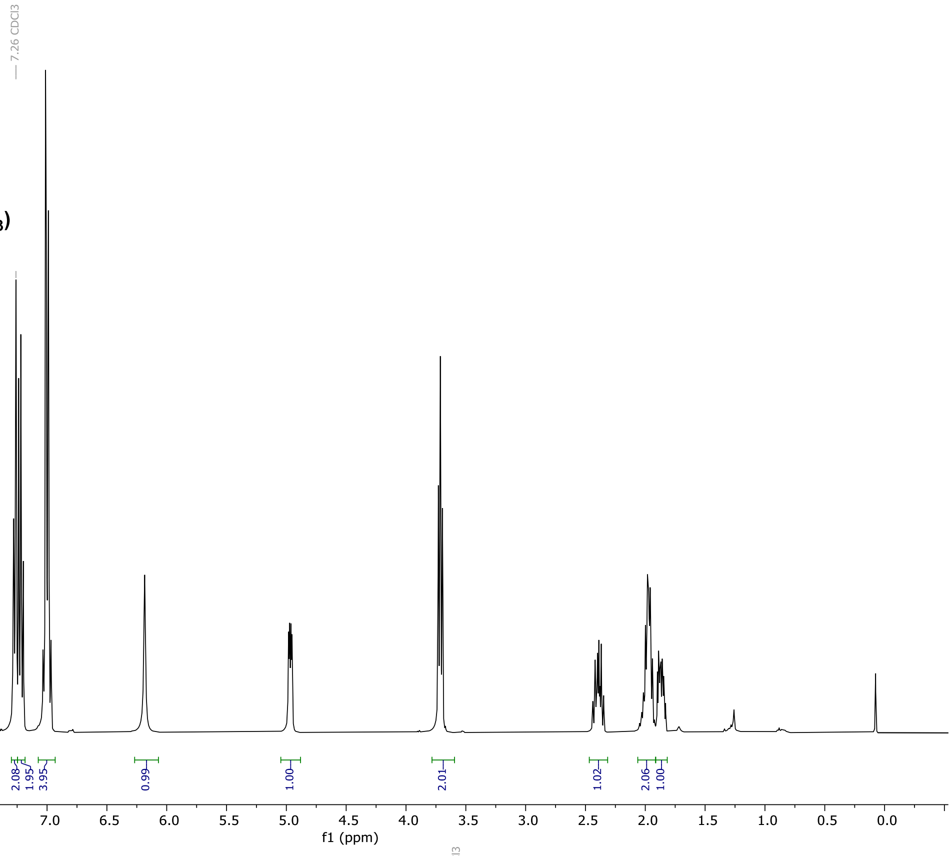

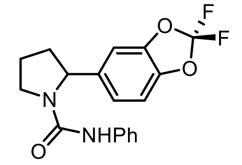

rac-**23**
(^{13}C NMR, 101 MHz, CDCl$_3$)

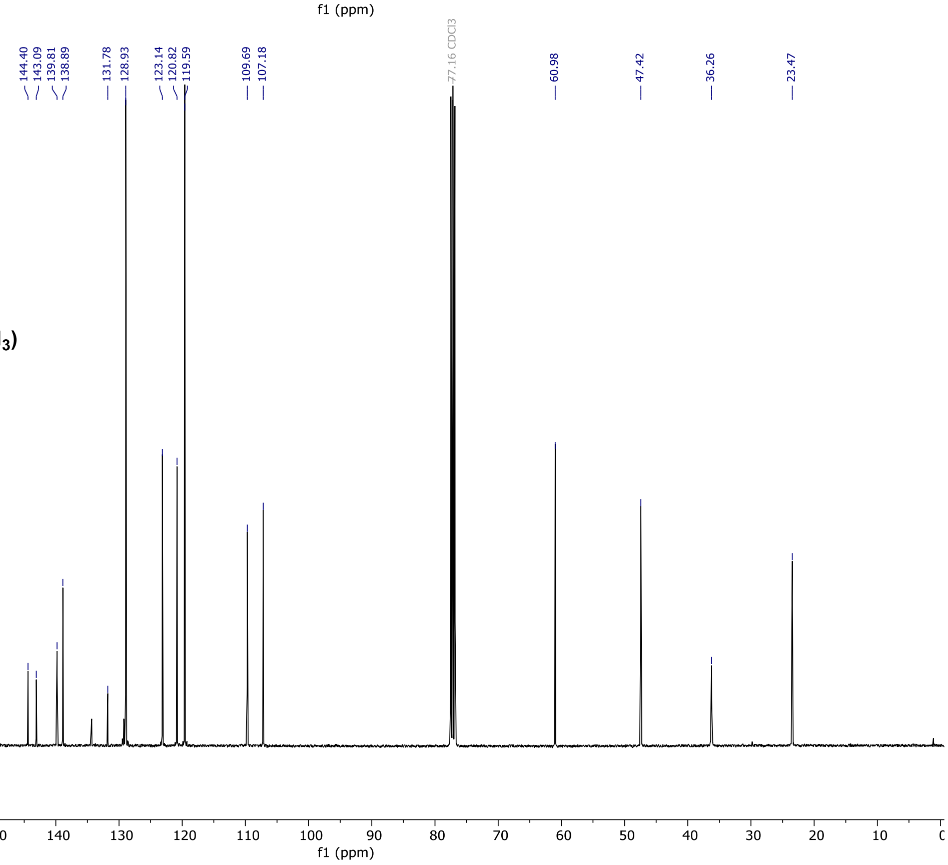

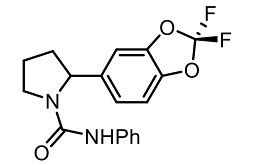

(¹⁹F NMR, 376 MHz, CDCl$_3$)

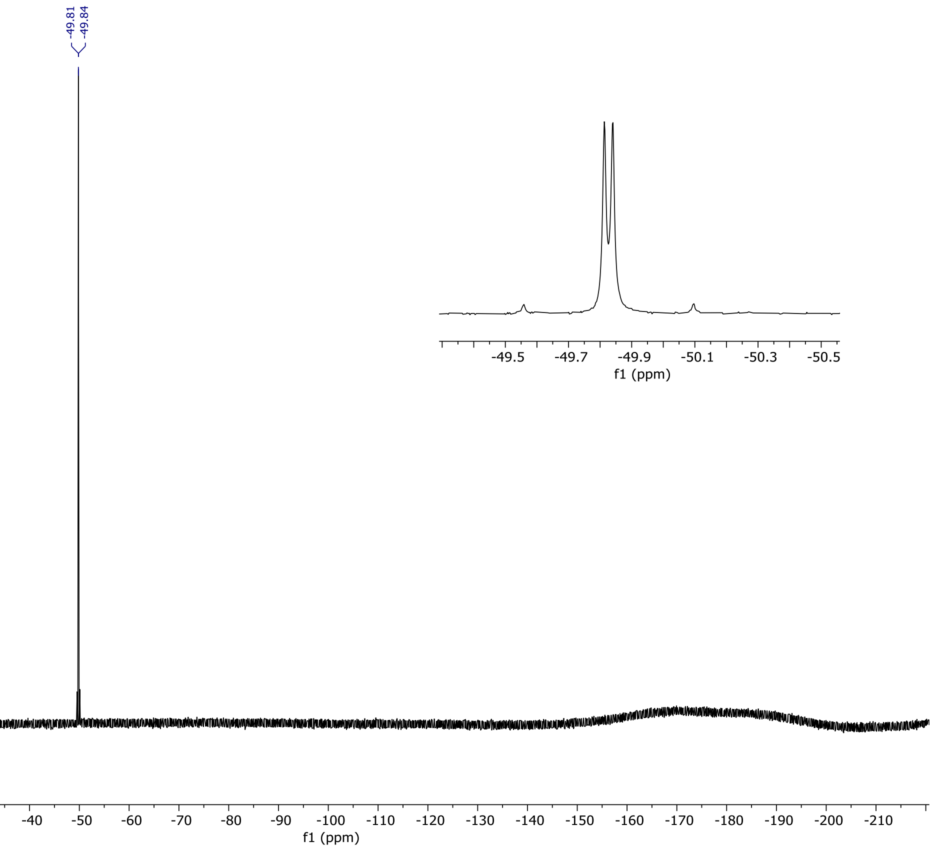

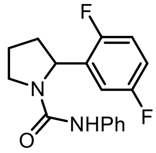

rac-**24**
(¹H NMR, 400 MHz, CDCl$_3$)

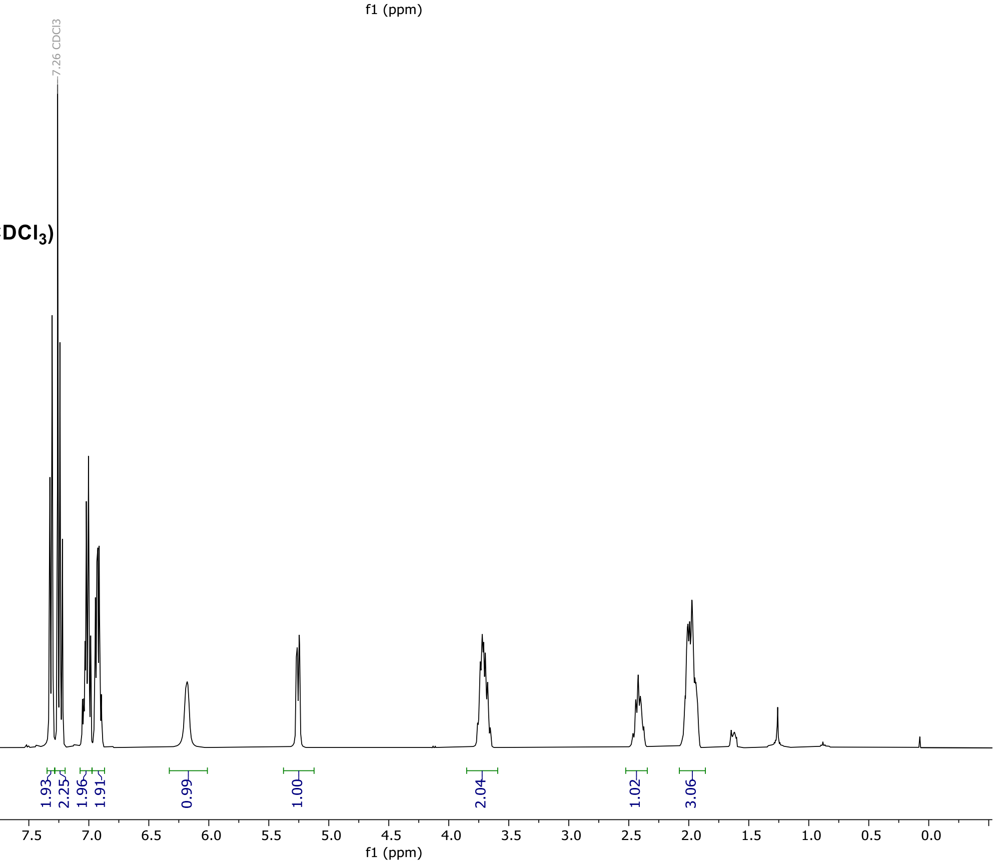

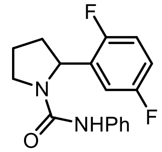

rac-**24**
(^{13}C NMR, 101 MHz, CDCl$_3$)

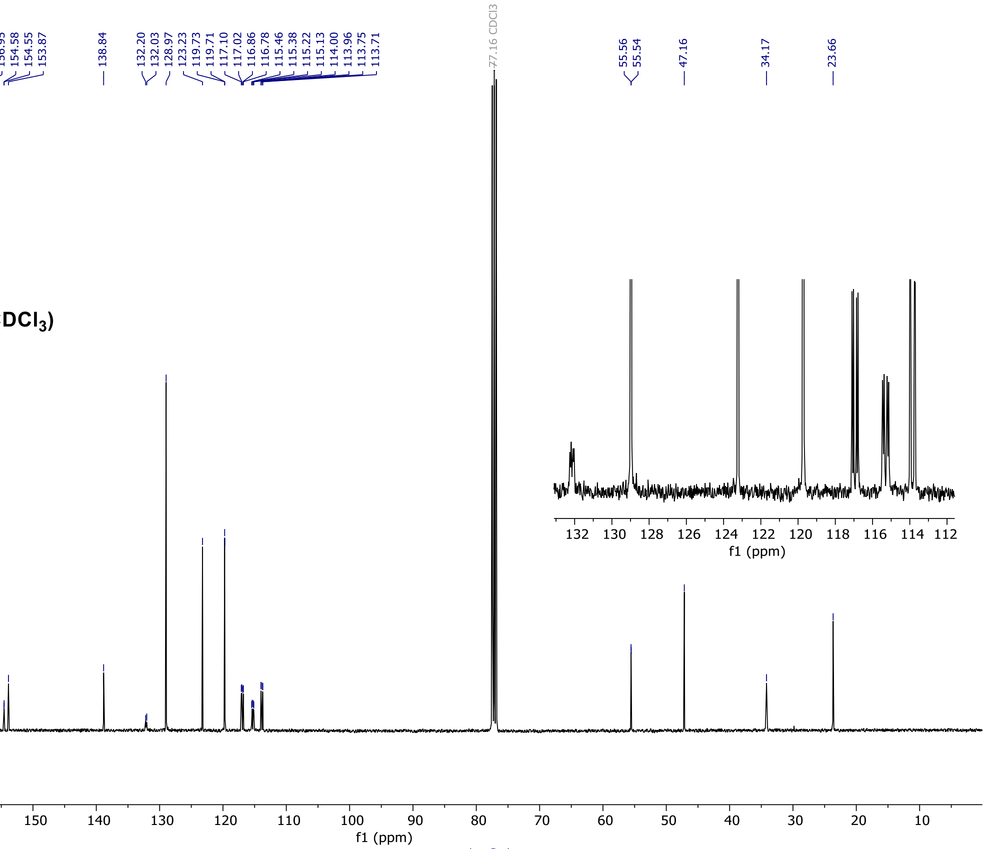

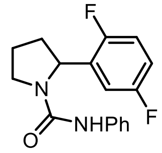

rac-**24**
(^{19}F NMR, 376 MHz, CDCl$_3$)

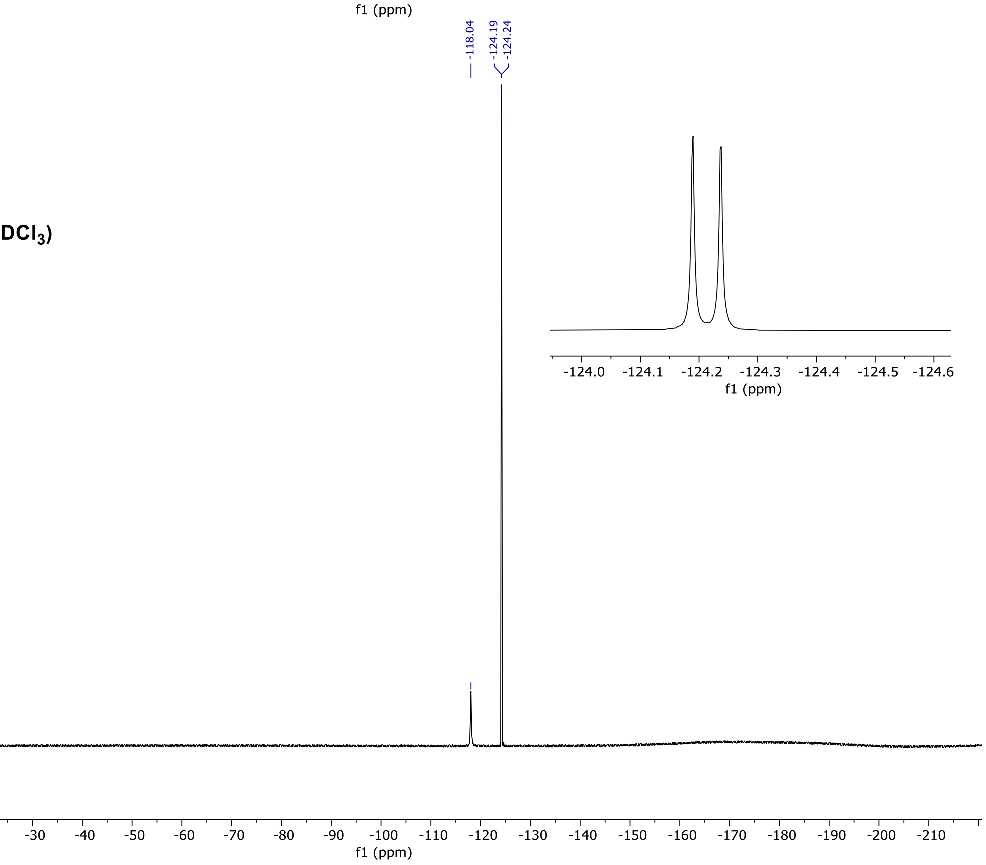

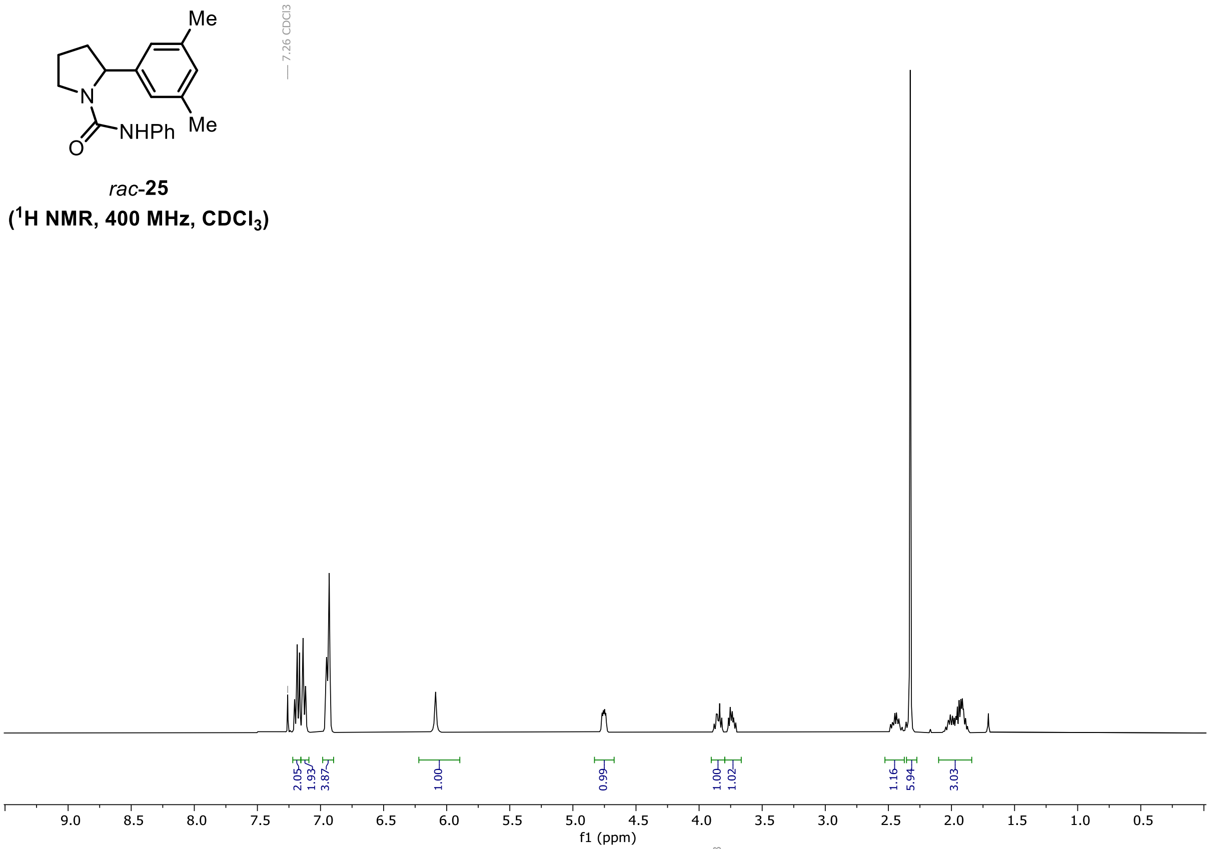

rac-**25**
(^{1}H NMR, 400 MHz, CDCl$_3$)

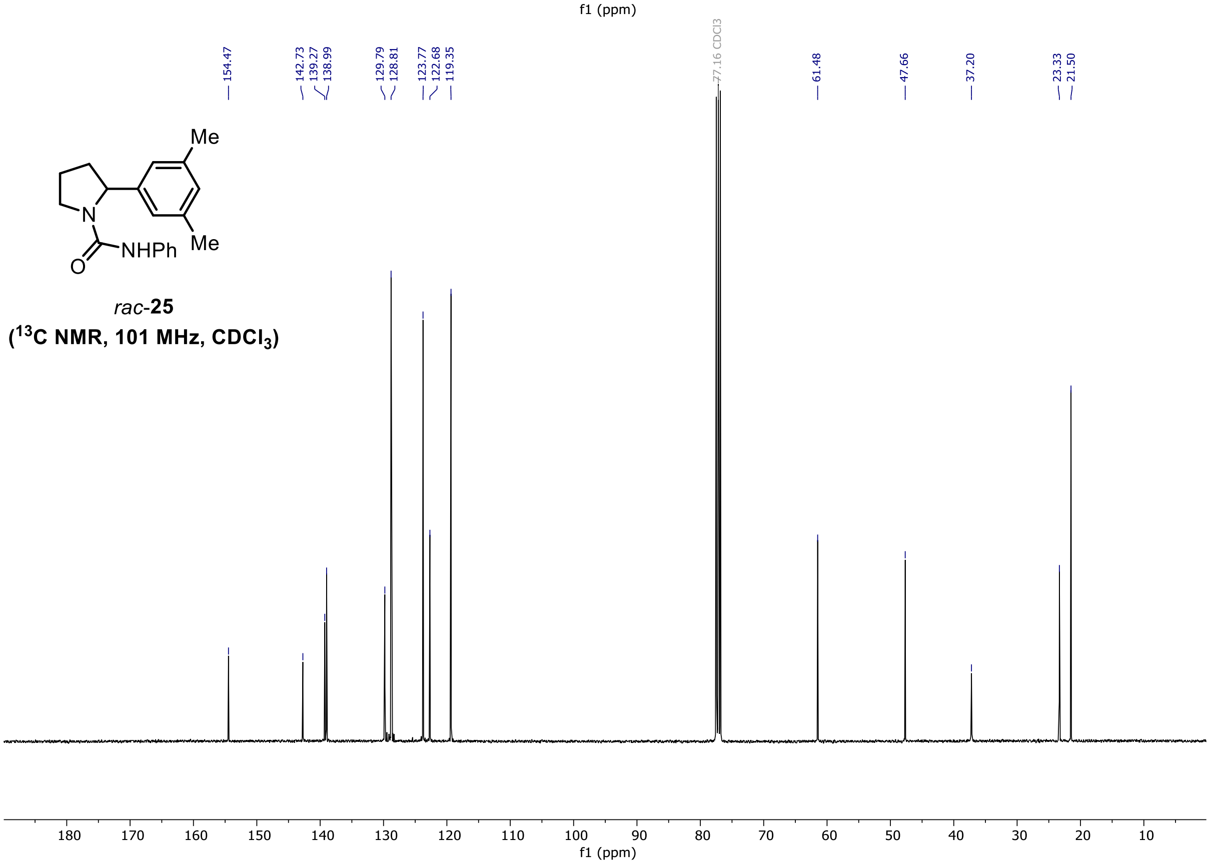

rac-**25**
(^{13}C NMR, 101 MHz, CDCl$_3$)

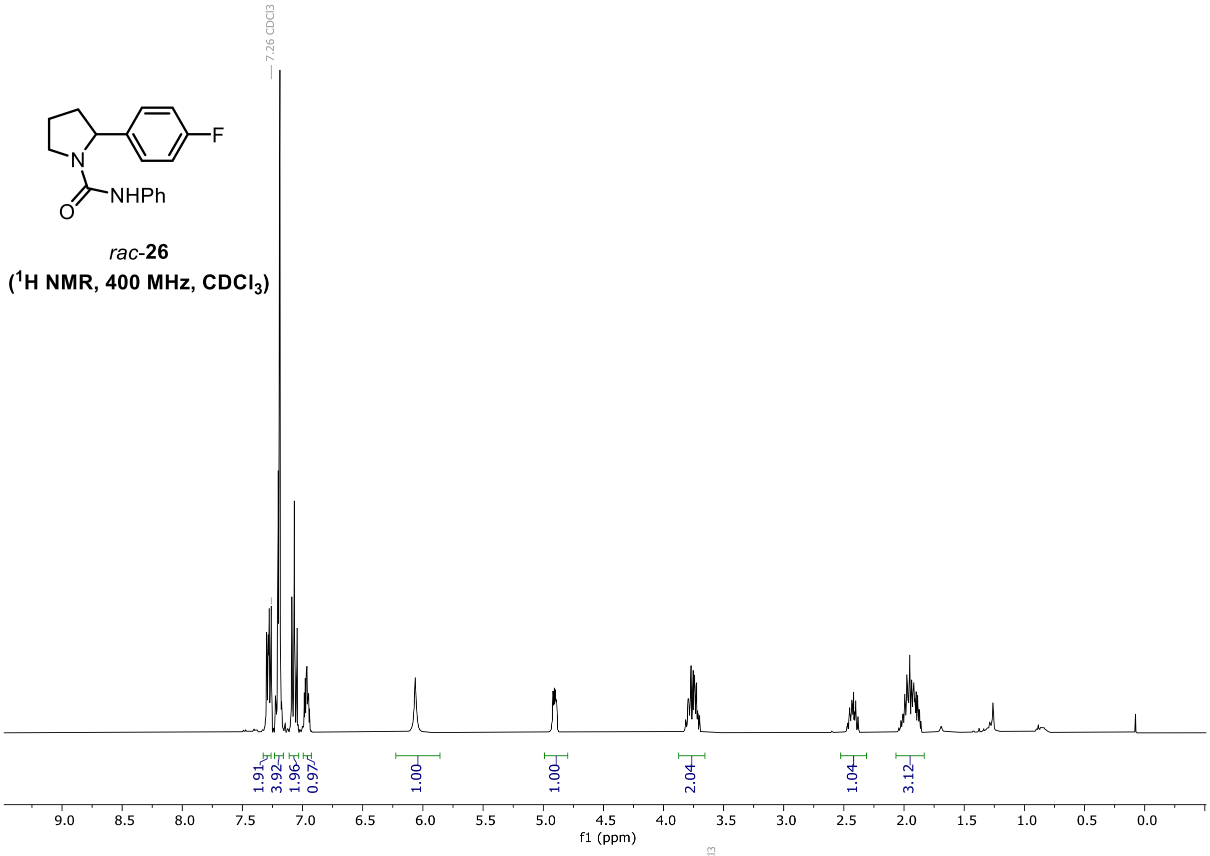

rac-**26**
(^{1}H NMR, 400 MHz, $CDCl_3$)

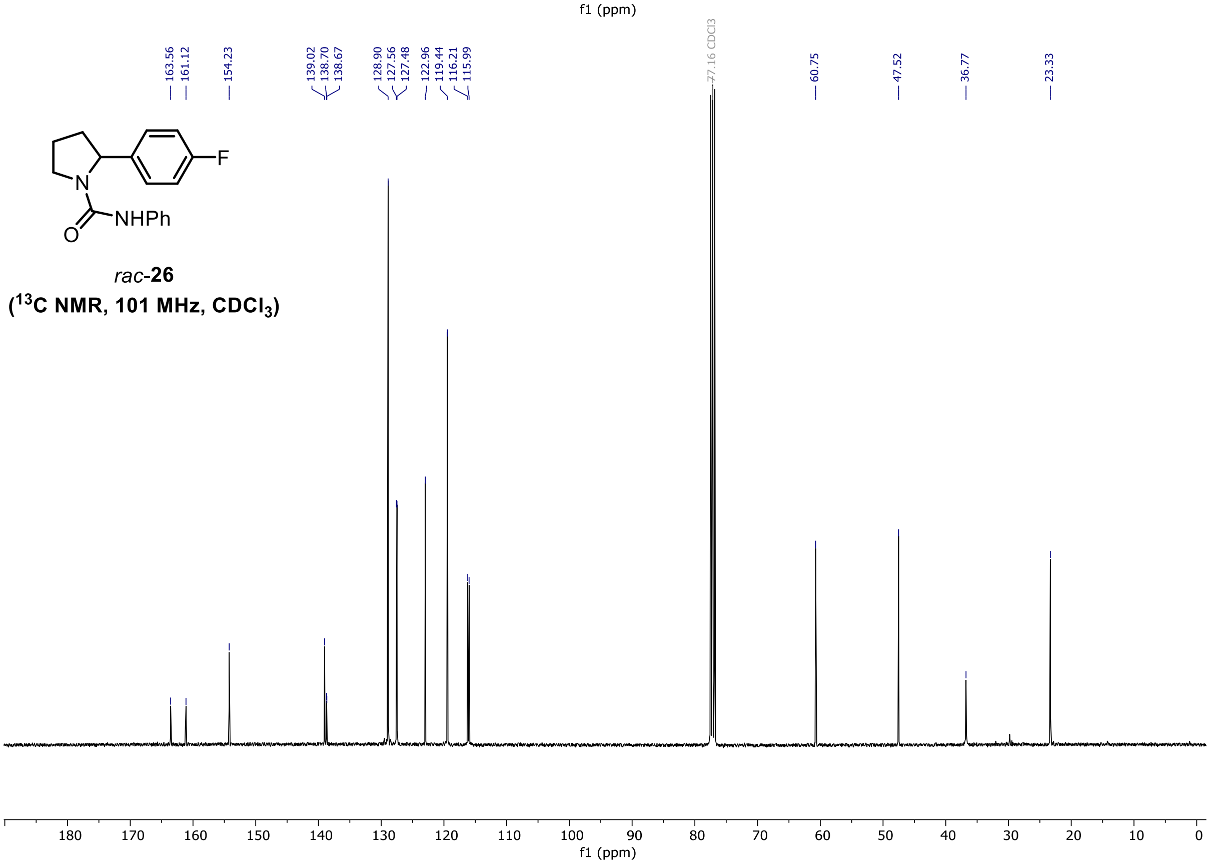

rac-**26**
(^{13}C NMR, 101 MHz, $CDCl_3$)

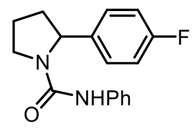

rac-**26**
(^{19}F NMR, 376 MHz, CDCl$_3$)

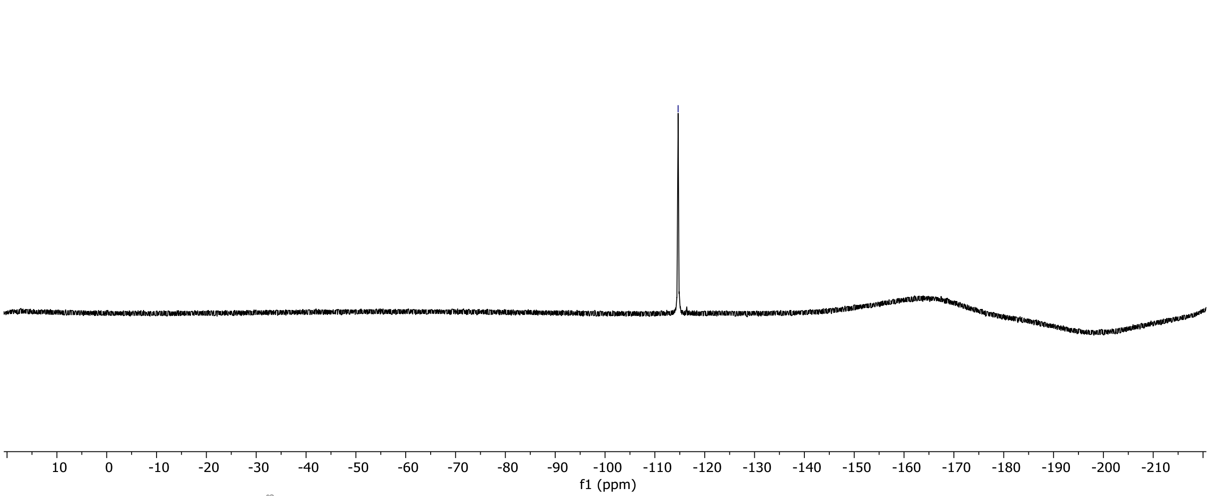

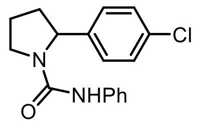

rac-**27**
(^{1}H NMR, 400 MHz, CDCl$_3$)

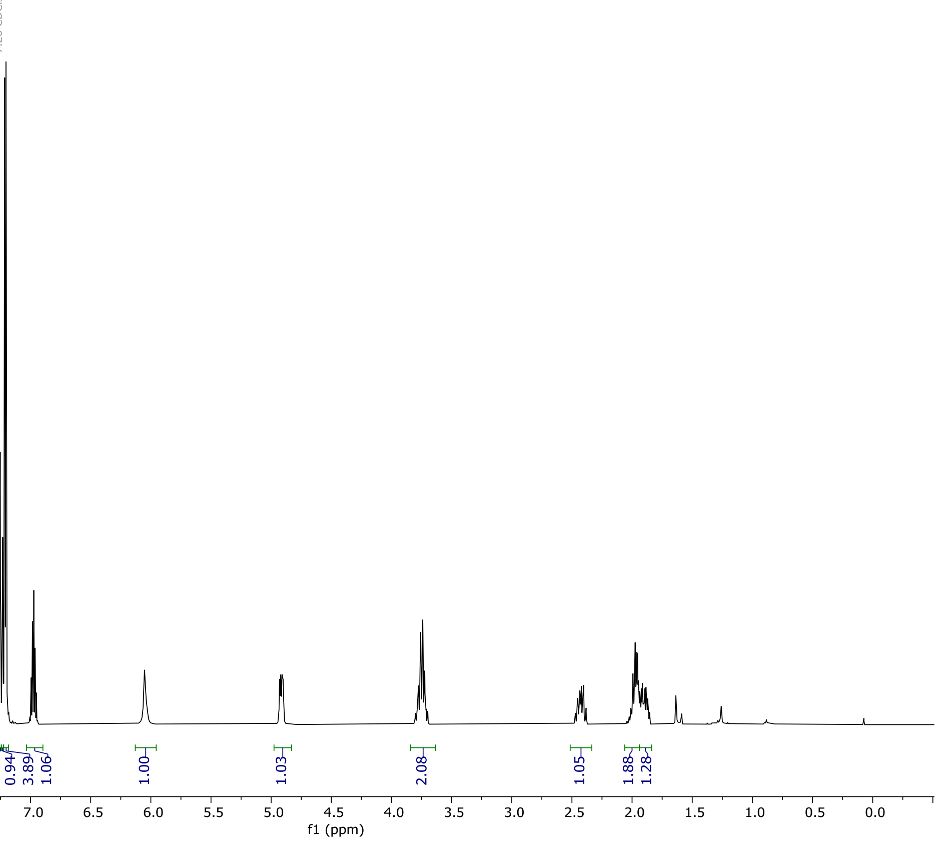

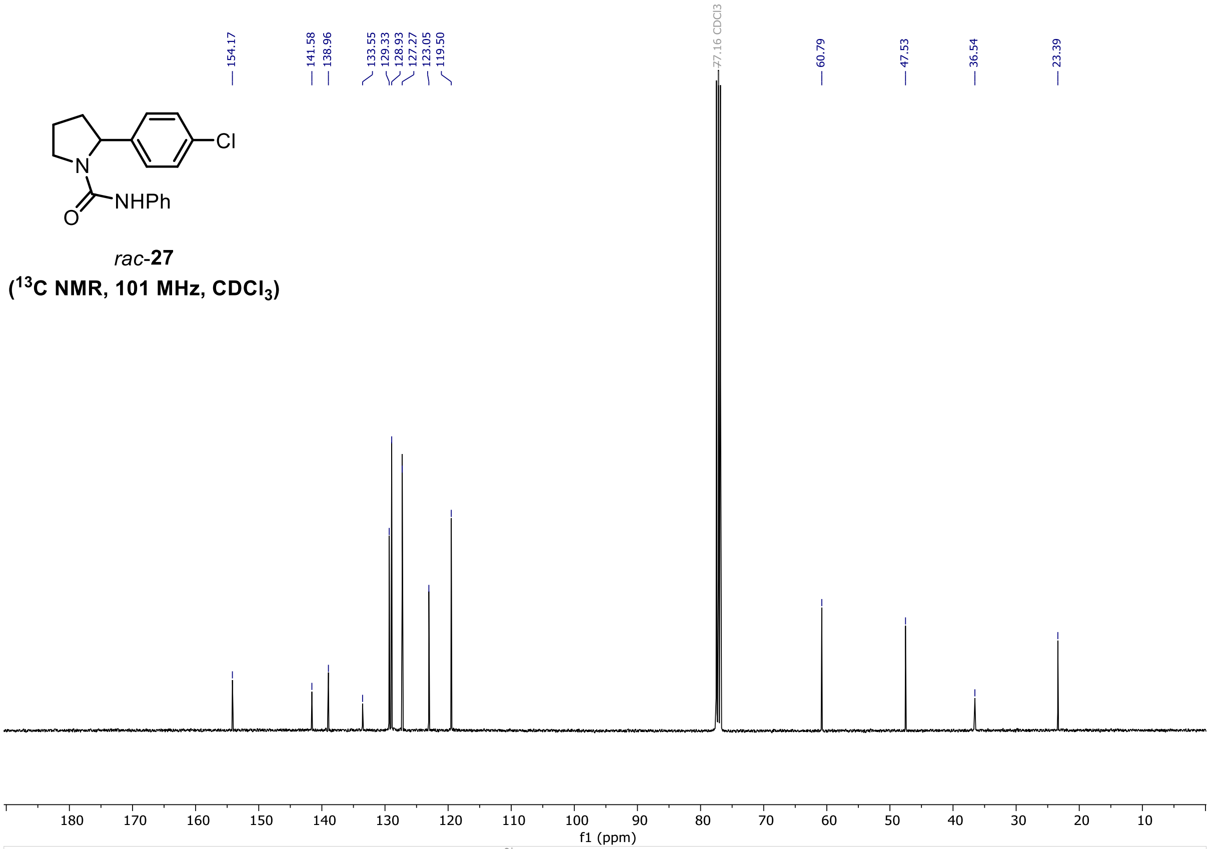

rac-**27**
(^{13}C NMR, 101 MHz, CDCl$_3$)

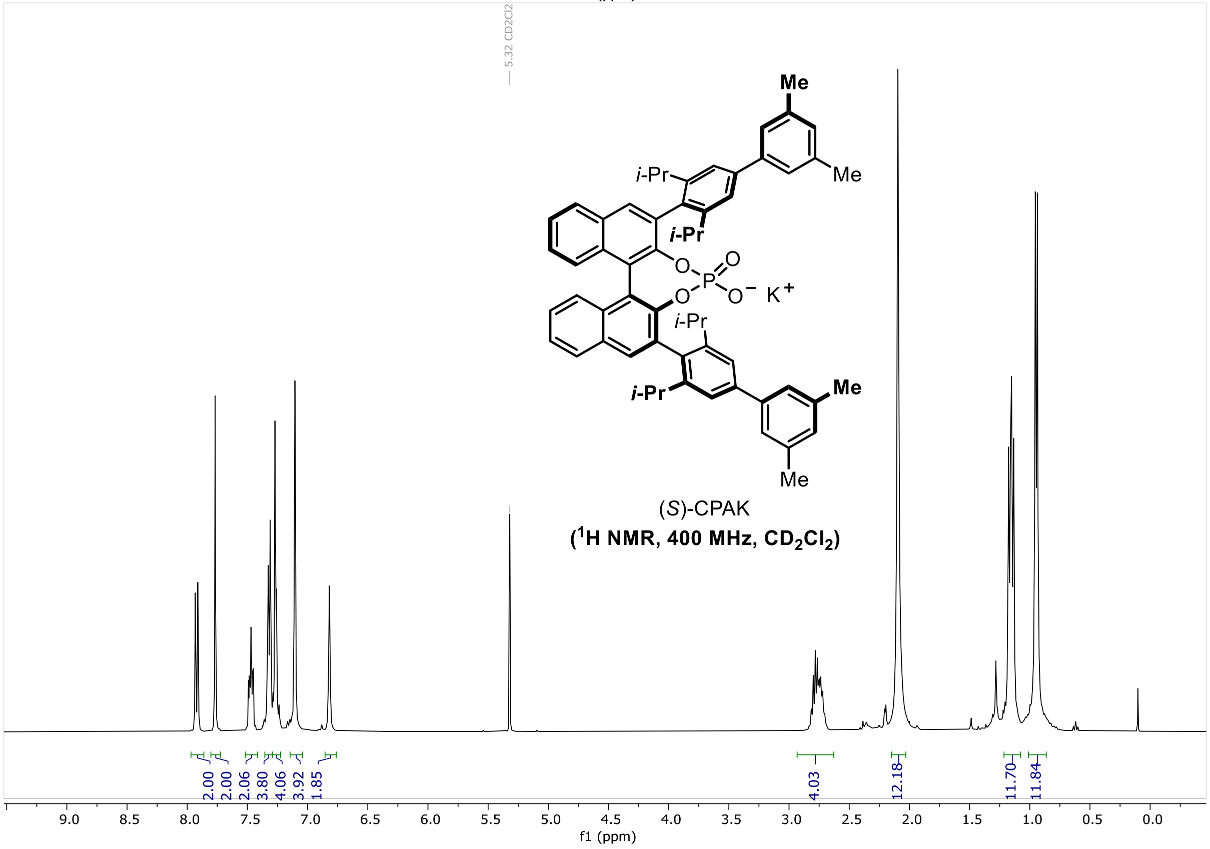

(*S*)-CPAK
(^{1}H NMR, 400 MHz, CD$_2$Cl$_2$)

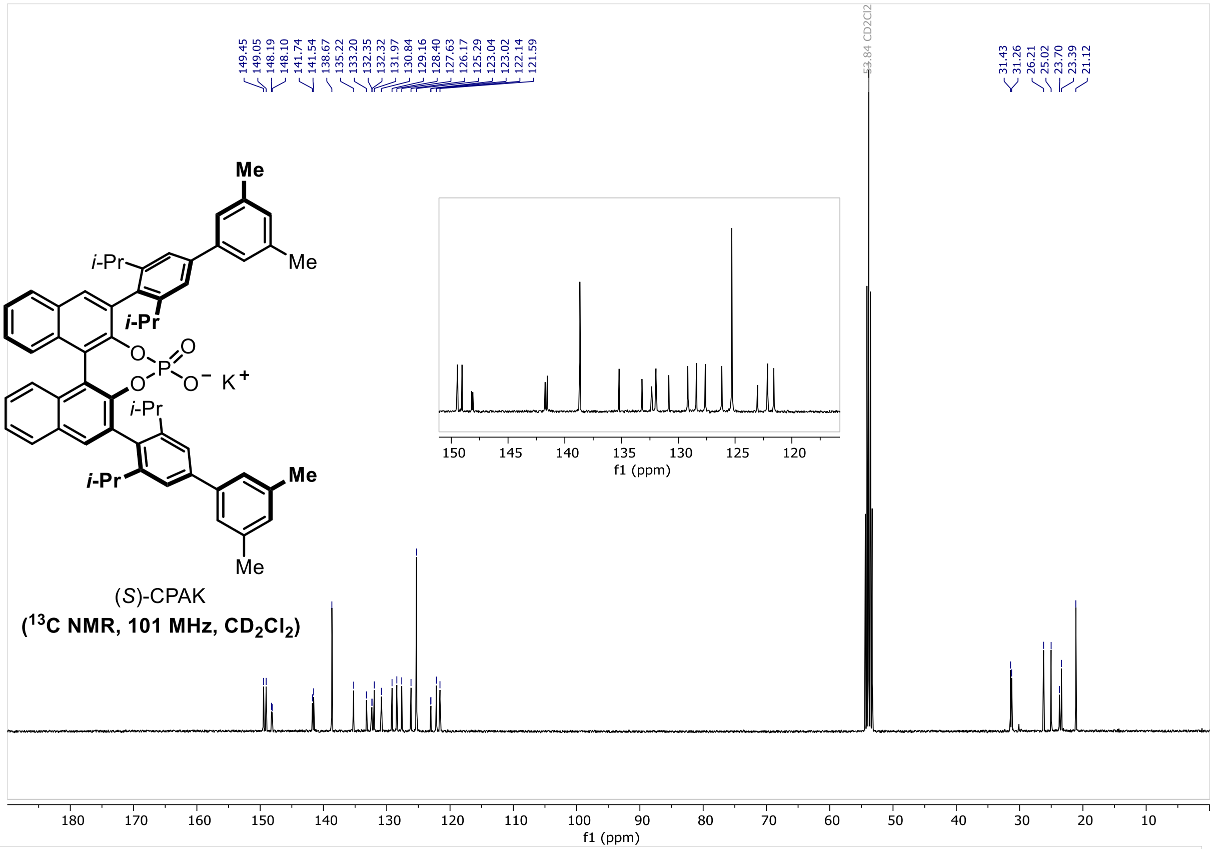
149.45
149.05
148.19
148.10
141.74
141.54
138.67
135.22
133.20
132.35
132.32
131.97
130.84
129.16
128.40
127.63
126.17
125.29
123.04
123.02
122.14
121.59
53.84 CD2Cl2
31.43
31.26
26.21
25.02
23.70
23.39
21.12
(S)-CPAK
(^{13}C NMR, 101 MHz, CD_2Cl_2)
f1 (ppm)

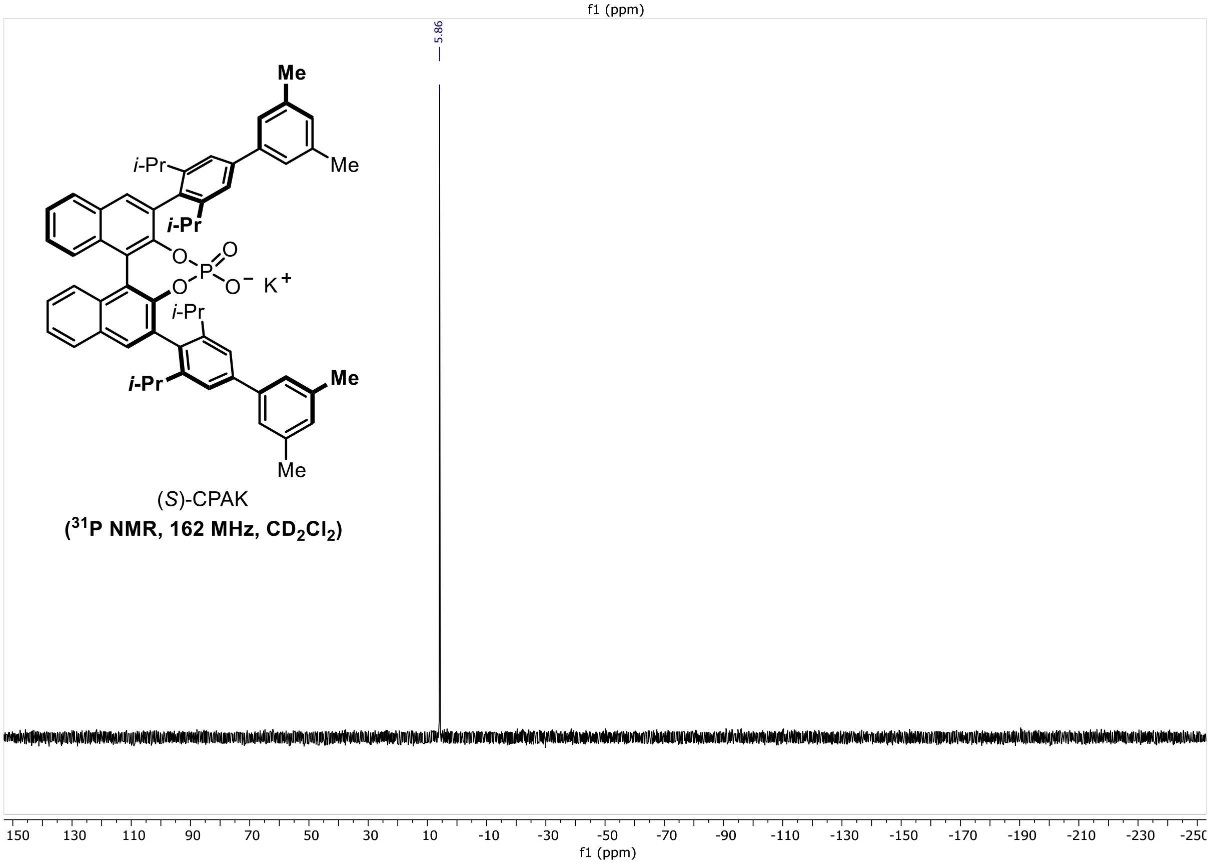
5.86
(S)-CPAK
(^{31}P NMR, 162 MHz, CD_2Cl_2)
f1 (ppm)

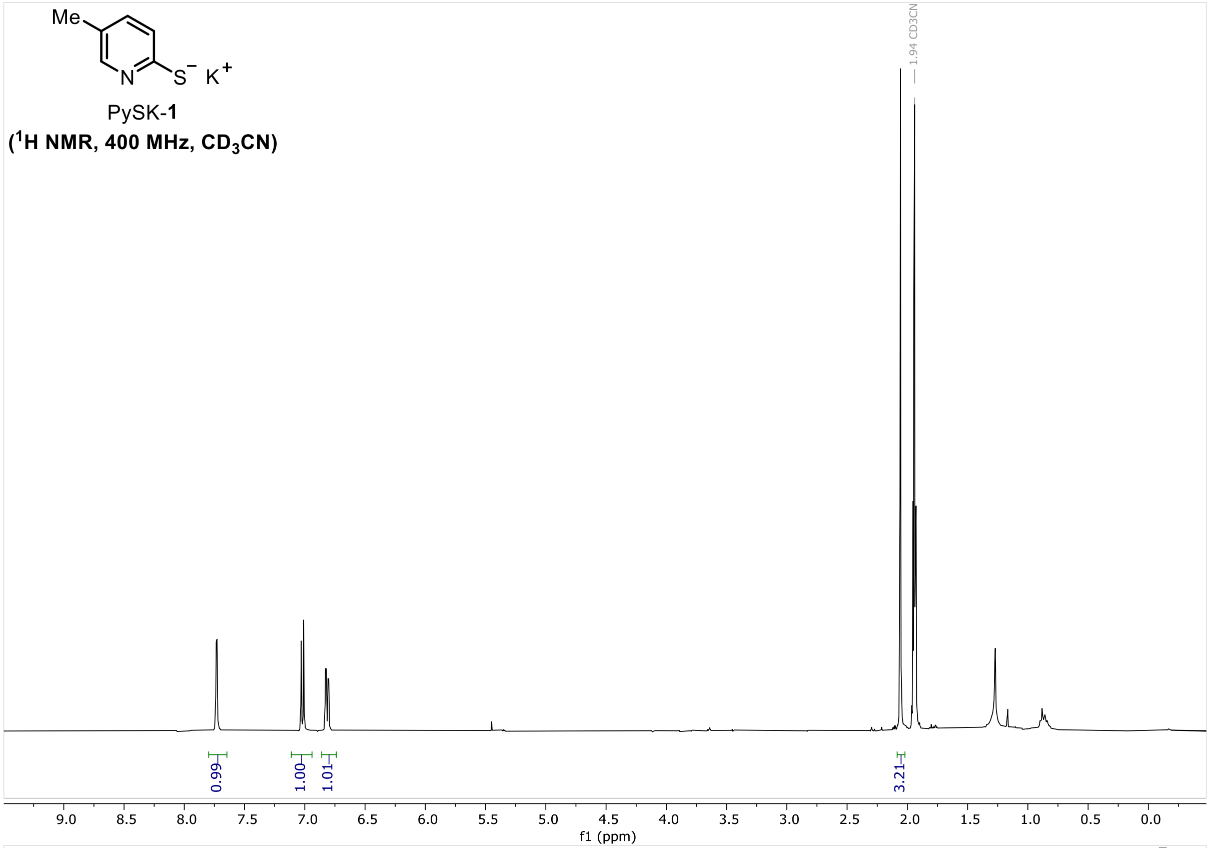
Me
N
S⁻ K⁺
PySK-1
(1H NMR, 400 MHz, CD3CN)
1.94 CD3CN
0.99
1.00
1.01
3.21
9.0 8.5 8.0 7.5 7.0 6.5 6.0 5.5 5.0 4.5 4.0 3.5 3.0 2.5 2.0 1.5 1.0 0.5 0.0
f1 (ppm)

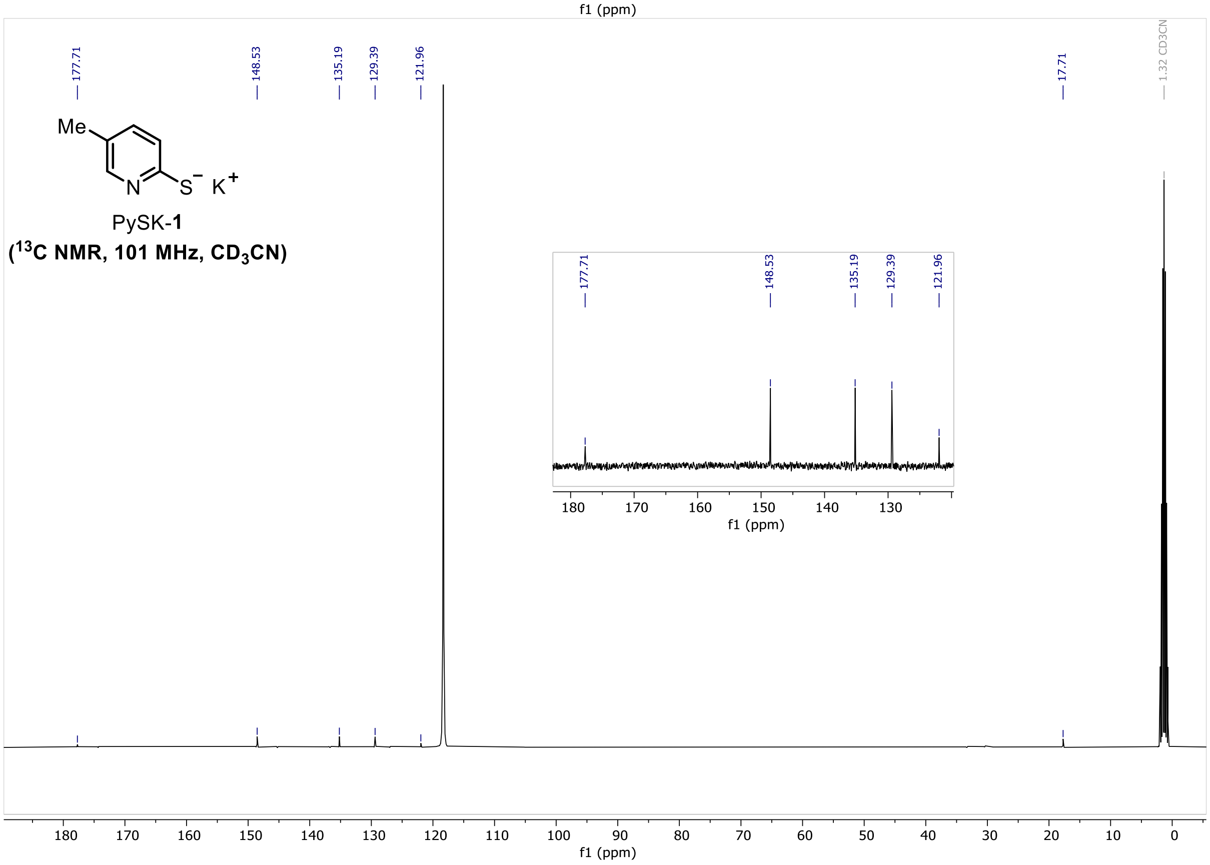
177.71
148.53
135.19
129.39
121.96
17.71
1.32 CD3CN
Me
N
S⁻ K⁺
PySK-1
(13C NMR, 101 MHz, CD3CN)
177.71
148.53
135.19
129.39
121.96
180 170 160 150 140 130
f1 (ppm)
180 170 160 150 140 130 120 110 100 90 80 70 60 50 40 30 20 10 0
f1 (ppm)

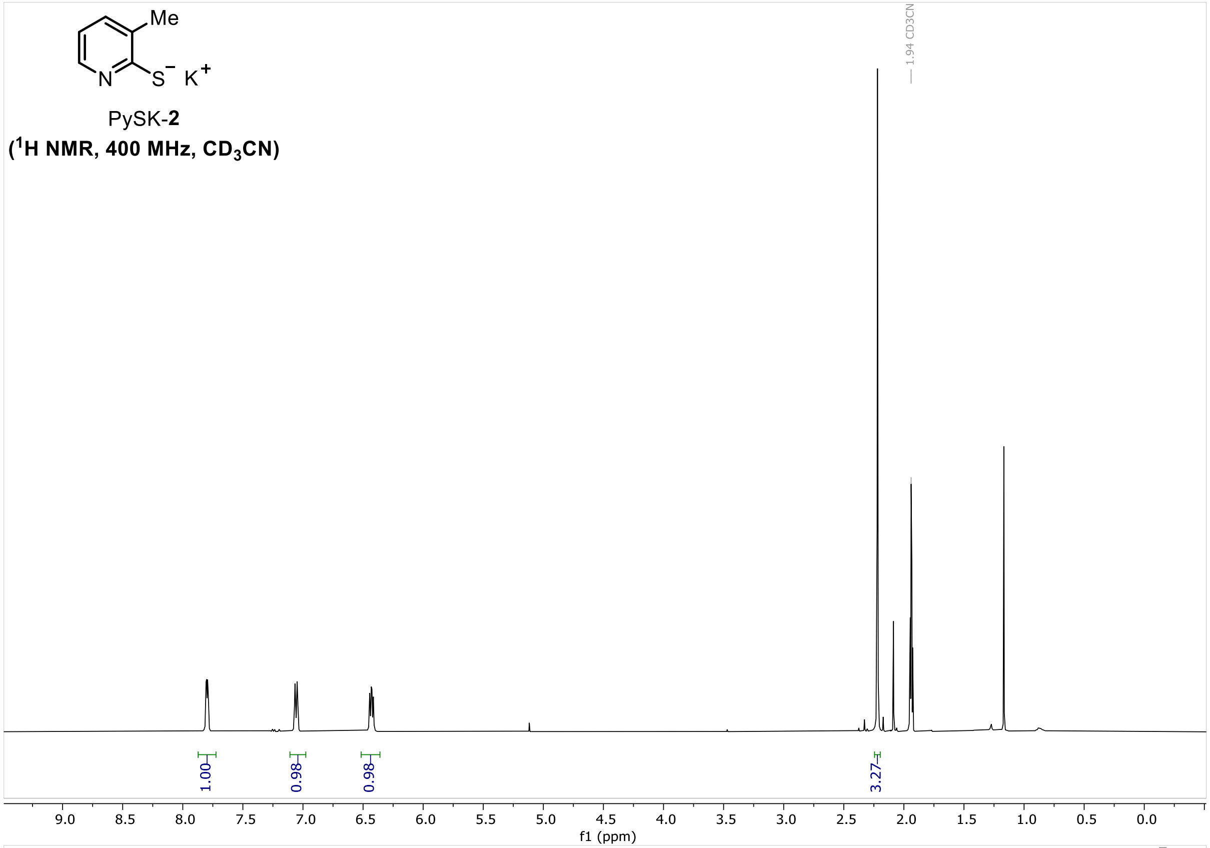
Me
N
S⁻ K⁺
PySK-2
(1H NMR, 400 MHz, CD3CN)
1.94 CD3CN
1.00
0.98
0.98
3.27
9.0 8.5 8.0 7.5 7.0 6.5 6.0 5.5 5.0 4.5 4.0 3.5 3.0 2.5 2.0 1.5 1.0 0.5 0.0
f1 (ppm)

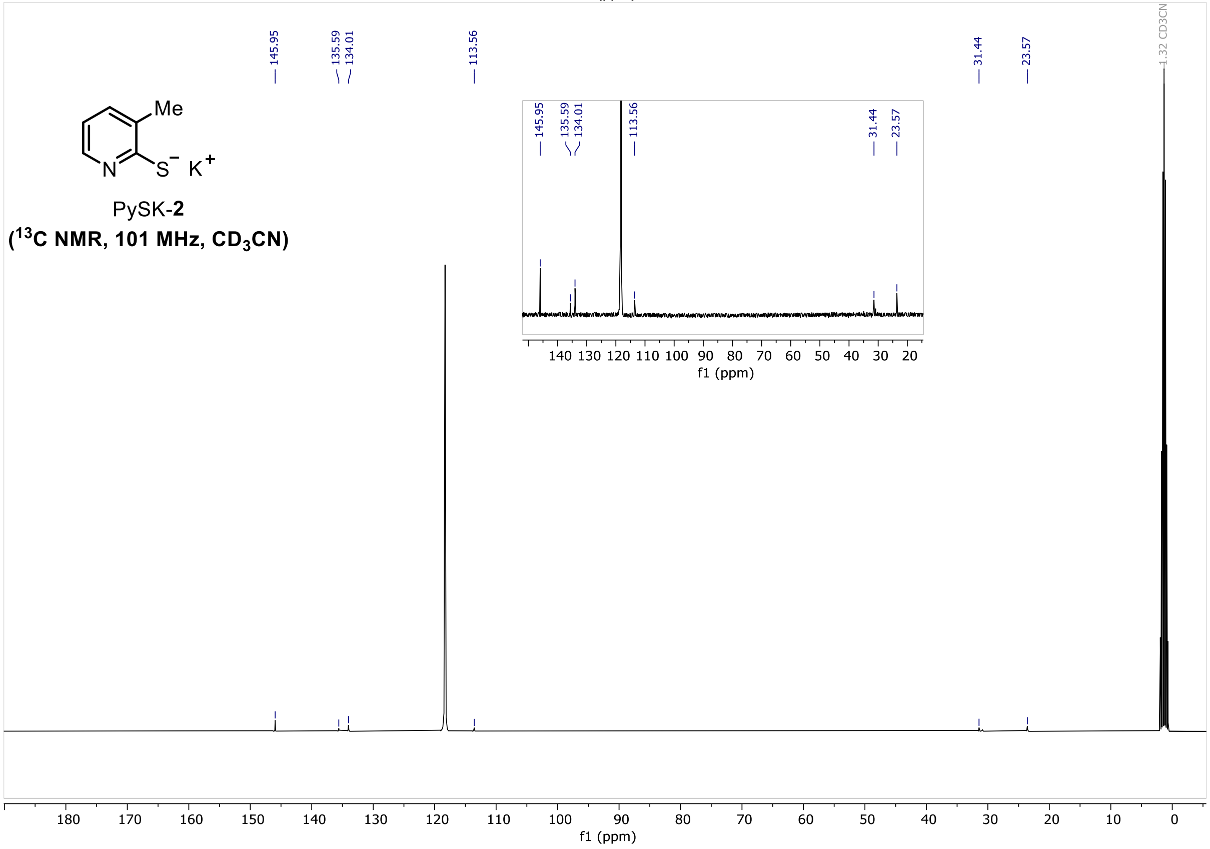
145.95
135.59
134.01
113.56
31.44
23.57
1.32 CD3CN
Me
N
S⁻ K⁺
PySK-2
(13C NMR, 101 MHz, CD3CN)
140 130 120 110 100 90 80 70 60 50 40 30 20
f1 (ppm)
180 170 160 150 140 130 120 110 100 90 80 70 60 50 40 30 20 10 0
f1 (ppm)

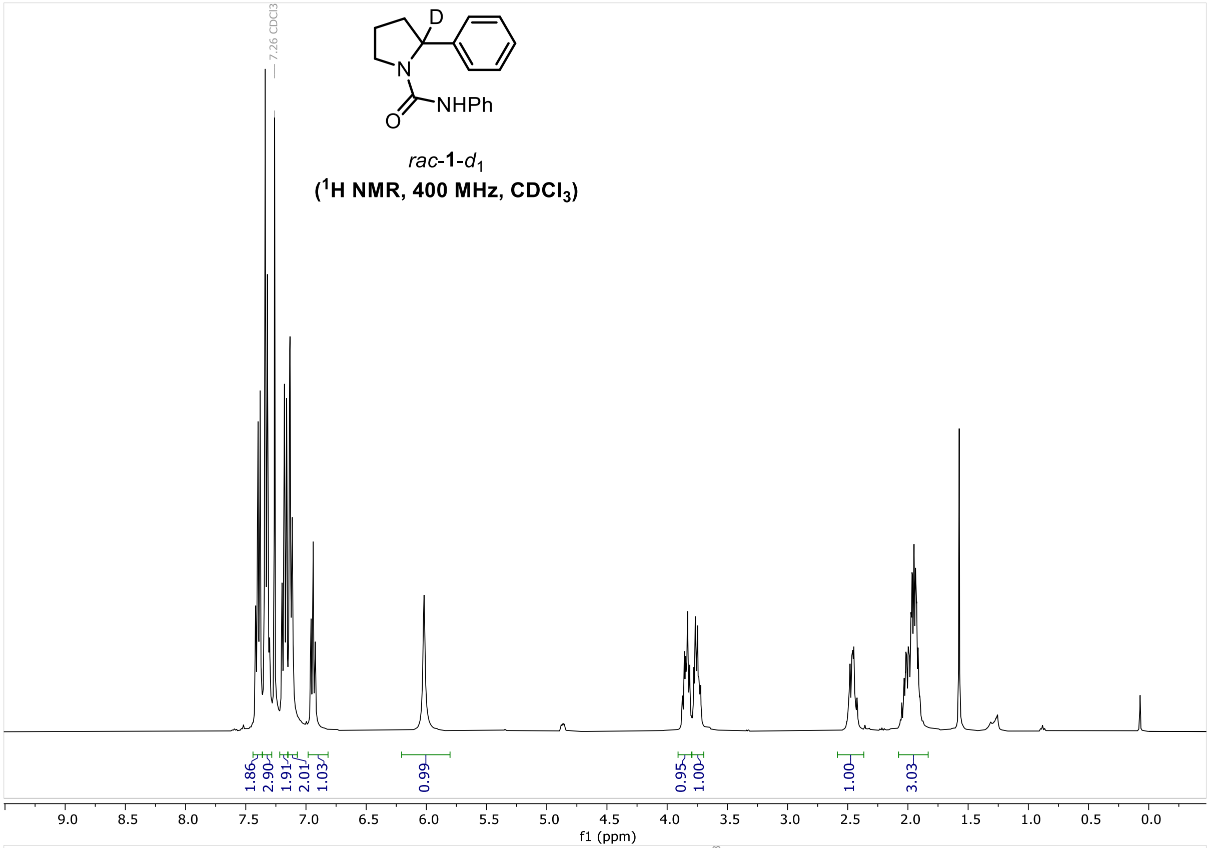

rac-**1**-d_1
(^{1}H NMR, 400 MHz, $CDCl_3$)

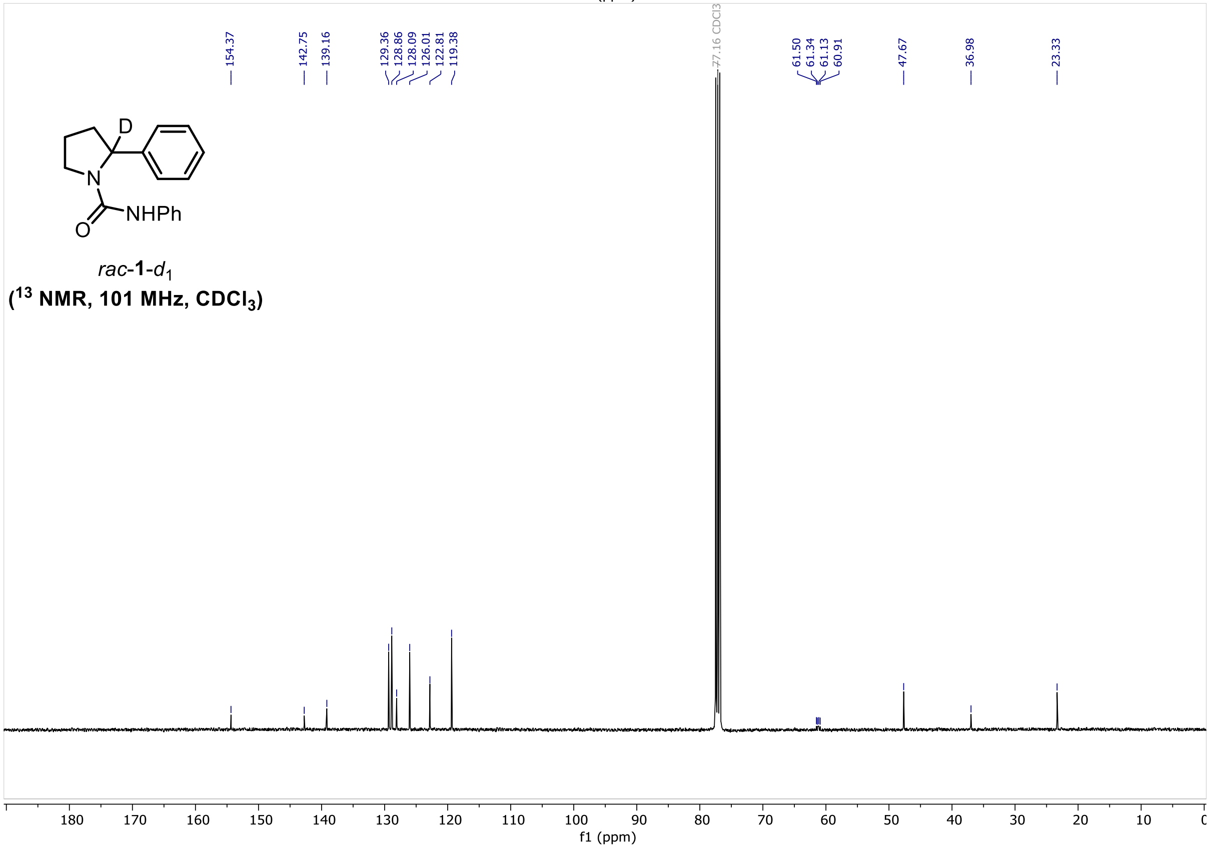

rac-**1**-d_1
(^{13} NMR, 101 MHz, $CDCl_3$)
