# Supplementary material for: Enantioselective hydrogen atom relay via non-covalent catalyst assembly
Source: Nature. 2026 Jun 1;655(8122):381–8. doi: 10.1038/s41586-026-10692-4 (PMC13345913; doi:10.1038/s41586-026-10692-4)
Supplement: Supplementary file 2 — Peer Review File [file 41586_2026_10692_MOESM2_ESM.pdf]

nature portfolio

Peer Review File

Enantioselective hydrogen atom relay via non-covalent catalyst assembly

Corresponding Author: Dr Giuseppe Zuccarello

Any redactions in this file are there to maintain patient confidentiality, the confidentiality of unpublished data, or to remove third-party material.

This file contains all reviewer reports in order by version, followed by all author rebuttals in order by version.

Attachments originally included by the reviewers as part of their assessment can be found at the end of this file.

Version 0:

Reviewer comments:

Referee #1

(Remarks to the Author)
In this manuscript, Zuccarello and coworkers describe the discovery of a creative and robust, photocatalytic strategy for enantioselective deracemization of 2-aryl pyrrolidines (with urea protecting groups). The ready access to a single enantiomer of these common drug motifs - and by such a simple, catalytic process - is truly impressive.

Indeed, the concept is quite well explored, optimized, and evaluated with a range of interesting, drug like pyrrolidines being deracemized or even interconverted. The selectivity is consistently high across these examples - illustrating high robustness and utility.

The major critique is the likely flawed understanding of the mechanism. Conceptually, in the Intro (in Fig 1C) and later again in the proposed mechanism (Fig 5H), a proposal is put forth that would violate the principle of microscopic reversibility. Specifically, if a chiral catalyst favors HAA of one enantiomer (e.g. R over S) than the microscopic reverse (HAD) between that same catalyst and substrate pair would dictate the same enantiomer should be formed (returning again to R over S). The very few examples of deracemizations have dealt with this paradox by separating the mechanisms of radical generation versus trapping. For example, instead of HAA by the chiral catalyst, the Ir could directly oxidize the amine (followed by rapid loss of the alpha proton to afford the net HAA product). Based on the Stern-Volmer quenching studies, the authors ruled out such a pathway (despite its satisfactory avoidance of the microscopic reversibility paradox). However, I urge them to consider testing other quenching combinations in order to find an alternate proposal that does not have the same catalyst performing both HAA and HAD (since only one diastereomeric transition state can be lower, and not alternating between R and S). Possibilities may simply include solubilization by CPAK ion pair and an achiral oxidation by Ir (then deprotonation by PyS) - for an overall racemic HAA, followed by enantioselective HAD. Or if HAA is selective, than HAD could be racemic, but this would likely take longer to deracemize.

Overall, the authors have done a thorough job describing the reaction development, mechanistic elucidation, excellent scope of reactivity, and synthetic utility. It is a highly scholarly work, including a thoughtful discussion and well-done SI, which is exceptionally detailed and the authors should be commended for this thorough effort.

Publication is highly recommended upon addressing the major critique described above, as well as the following minor comments.

Minor Revisions:

Intro - Please fix this text: “Despite seminal work by … through reversible C(sp3)–H cleavage have only been realized with highly specialized substrates.” This critique is a little too broad, and could easily be applied to this work too. Perhaps merely mentioning that those seminal works exist might be a better strategy.

Figure 3 - Use arrowheads to indicate progression of the optimization. The end(?) in Box 3 is particularly confusing.

Figure 4 - These scope studies are very well done. Excellent choices of widely ranging and useful molecules.

Figure 5 - Great mechanistic section. Minor additions:

Fig 5a: Indicate which catalyst is used

Fig 5b: >1:99 should be <1:99 (or >99:1 enantiomer)

Fig 5c: Quenching:
Repeat with the matched vs mismatched pairs to probe HAA vs HAD.
Include a solubilizing agent for the substrate probe (e.g. rac-CPAK or an achiral biphenyl phosphate).
Consider a triple combination (1:1:1) to probe the proposed B in the mechanism.
The structure of PySK-2 is out of place and confusing.

Fig 5e: only R = Me is 29, H = 1

Fig 5f: what rates are being measured? Isomerization? Are these parallel experiments? Please indicate.
On the right side of this box (D scrambling), where is the H from? Perhaps the urea NH? Pleased indicate.

Fig 5h: The progression of B > C > D violates the principle of microscopic reversibility. If B > C favors R consumption, then C > D would favor R re-generation, since it must be the same diastereomeric transition states (pro-R less energy than pro-S).

Referee #2

(Remarks to the Author)
The work by Zuccarello and co-workers reports an enantioselective photochemical deracemization of 2-aryl pyrrolidines enabled by a non-covalent, in situ “assembled” chiral HAT catalyst formed from a chiral phosphoric acid and 2-mercaptopyridine. In contrast to previous studies, the central claim of the work relies on controlling both enantioselective hydrogen atom abstraction (HAA) and enantioselective hydrogen atom delivery (HAD). Conceptually, the combinatorial catalyst idea – assembled from commercially available precursors via non-covalent interactions – for this type of transformation is appealing, and the simplicity of the reaction design is elegant. Although currently limited to the 2-aryl pyrrolidines, the scope is fairly broad within the selected substrate class, with generally high yields and good to excellent er.

The mechanistic experiments are broadly consistent with the work's central claims but not fully conclusive. The kinetic isotope effect, together with Stern-Volmer studies, indicates that the reaction occurs via HAT. Deuterium scrambling experiments (including crossover experiments), especially with different enantiomers, are consistent with an in-cage HAA. However, the current evidence for the proposed non-covalent assembly is rather thin and indirect. To further substantiate the mechanistic proposal, the existence of the assembly should be supported by at least NMR experiments (e.g., titration/DOSY). Furthermore, the authors should attempt to deconvolve the respective contributions of the claimed enantioselective HAA and HAD.

Overall, the work describes a valuable transformation utilizing an appealing strategy. The manuscript currently relies heavily on a conceptual framework (non-covalently assembled chiral HAT catalyst, enantioselective control of HAA and HAD) that is not yet well supported. Addressing the points above – particularly direct evidence for the proposed assembly and deconvolution of HAA vs HAD – would substantially strengthen the manuscript. I therefore recommend revisions prior to publication.

Referee #3

(Remarks to the Author)
I co-reviewed this manuscript with one of the reviewers who provided the listed reports.

Referee #4

(Remarks to the Author)
In this manuscript, Zuccarello and co-workers report the photochemical deracemization of 2-aryl pyrrolidines using a combination of a 2-mercaptopyridine and a chiral phosphoric acid. The authors proposed that this combination forms a “chiral thiol” in situ via non-covalent interactions, which then effects enantioselective HAA to break the stereogenic C–H bonds followed by enantioselective HAD to reform the C–H bonds. A series of mechanistic studies have been conducted. While the concept of forming a “chiral thiol” in situ is interesting, the proposed deracemization mechanism is fundamentally incorrect as it violates the principle of microscopic reversibility, which dictates that using one chiral catalyst to effect asymmetric induction in both bond-breaking and bond-making events via the same mechanistic pathway will always result in an overall equilibrium of racemate because of the shared energy surface (for reviews and highlights, see: Angew. Chem. Int. Ed. 2009, 48, 2648; Angew. Chem. Int. Ed. 2023, 62, e202308241; Science 2019, 366, 304; Science 2023, 382, 373). In other words, if the HAA step consumes R faster, then the HAD step catalyzed by the same thiol will reform R faster, therefore, it is impossible to achieve enantioenrichment via the proposed mechanism. Accordingly, the entire manuscript,

including the related figures, should be rewritten/redesigned based on the correct mechanism. In addition, the similarity to known deracemization systems and the limited scope (detailed below) also make the manuscript unsuitable for publication in a top journal like Nature.

Mechanistic aspects
1. As mentioned above, the proposed mechanism (Fig. 5H) will result in a racemic mixture. The fact that enantioenrichment was achieved suggests alternative mechanisms. A recent work utilized a very similar system (an achiral thiol and a chiral phosphoric acid) for deracemization of α-Aryl Ketones (CCS Chem. 2026, doi: 10.31635/ccschem.025.202506718). In their work, a non-selective HAA by an arylthiyl radical followed by enantioselective protonation by the phosphoric acid was proposed, circumventing the principle of microscopic reversibility. The authors should add this work in Fig. 1 and examine in detail to see if the current system follow a similar pathway.
2. In addition to the aforementioned work, chiral phosphate anions have recently been used in several photochemical deracemization reactions, however, these closely related works were not discussed or cited: J. Am. Chem. Soc. 2025, 14625; J. Am. Chem. Soc. 2025, 15307.
3. In Fig. 5G, the observation that added PhSH erodes enantioselectivity does not prove enantioselective HAD. An alternative explanation is that PhSH generates an achiral thiyl radical that performs non-selective HAA, thereby lowering ee. This possibility should be evaluated.
4. In Fig. 5F, the low D/H exchange in the crossover experiment is interpreted as evidence for an in-cage HAA/HAD process. However, an alternative kinetic explanation exists: if non-deuterated rac-3 reacts much faster than rac-1-d1, the former could reach the photostationary state (95:5 er) before significant deuterium incorporation occurs. This alternative should be addressed.

Substrate scope:
1. The reaction requires substrates bearing a urea protecting group with a free N–H moiety, presumably to engage the chiral catalyst via hydrogen bonding. Furthermore, the stereocenter must be aryl-substituted. The generality of the method is therefore limited. To strengthen the study, the authors should explore other amine derivatives, including those with different ring sizes, and examine alkyl-substituted stereocenters.
2. The claim on page 5 (lines 85–89) that “successful photochemical deracemizations through reversible $C(sp^3)$–H cleavage have only been realized with highly specialized substrates” is an overstatement and overlooks several pertinent examples, for example, pyridylketones (J. Am. Chem. Soc. 2021, 13393), δ-and γ- lactams (J. Am. Chem. Soc. 2025, 1186), α-, β-, and γ-arylated ketones and esters (J. Am. Chem. Soc. 2025, 14625; J. Am. Chem. Soc. 2025, 15307; CCS Chem. 2026, doi: 10.31635/ccschem.025.202506718). These works should be cited and discussed to provide a balanced view of the field. The authors should also clarify what defines a “highly specialized substrate” and explain why their own substrates, which possess a specific hydrogen-bond donor, are not considered as such.

Overall, the manuscript presents an interesting catalytic system for photochemical deracemization, but the proposed mechanism is not supported by fundamental principles and requires substantial revision. Furthermore, the substrate scope is limited, and the discussion lacks appropriate context from recent literature. Once these issues are properly addressed, this work should be suitable for a more specialized journal whose readers could better appreciate the subtle mechanistic difference to the known deracemization systems.

Version 1:

Reviewer comments:

Referee #1

(Remarks to the Author)
I remain strongly in favor of publication of this important synthetic contribution, and I commend the authors for thoroughly addressing all the reviewers’ comments to the best of their ability.

However, I also remain skeptical that the new mechanism addresses the key issue concerning the principle of microscopic reversibility.

As drawn now, B to D are still the intermediates that would contribute to the same transition state. The proposed thione-thiol photo-tautomerization (drawn in between) is a reasonable complementary mechanism, but seems irrelevant to the key, shared transition state. The issue is that this T.S. is shared, not that there is a separate tautomerization possible. (To be fair, I’m not totally convinced that the radicals would wait around for another photon to excite them and mediate this isomerization, but that is beside the point).

Overall, I empathize with the authors that it is hard to rationalize the Stern-Volmer quenching studies, which caused them to rule out a more “typical” Ir oxidation of the amine (followed by alpha proton loss to afford the “net” HAA product) that conforms with the principle of microscopic reversibility. However, I think it is simply an issue of solubility (conveniently provided by the self-assembled catalyst complex), which I hoped they would find a solution to via the new triple combination S-V experiments. Thus, while they didn’t figure out this solubility issue (heterogeneity is a well-known issue in the study of kinetics), I am not sure that the overly complex (and still seemingly not quite right) explanation provided is needed.

Anyways, this is merely my strong opinion, but the authors are free to choose to pose whichever mechanistic hypothesis they believe the data is suggesting. Either way, I do NOT believe it negates from the synthetic achievement of this privileged

(in med chem especially) pyrrolidine deracemization (see their response to Reviewer 4 about the difference in substrate classes now possible versus previous carbonyl-based deracemizations). And I think the claims and analysis of the experiments are all sound, and they may very well contribute to a healthy discourse in the future scientific literature.

Referee #2

(Remarks to the Author)
The authors have satisfactorily addressed most of the concerns, including those of the other reviewers. The Job´s plot provided cannot be used to derive a stoichiometry; it rather indicates that there is a multiple step equilibrium and not the formation of a 1:1 assembly.
Despite some remaining doubts on the mechanism I consider the manuscript suitable for publication.

Referee #3

(Remarks to the Author)
I co-reviewed this manuscript with one of the reviewers who provided the listed reports.

Referee #4

(Remarks to the Author)
In the revised manuscript, the authors corrected their mechanism based on the photo-tautomerization of 2-mercaptopyridines, proposing that a nitrogen radical undergoes HAA while the S-H bond is responsible for HAD. This proposal indeed circumvents the principle of macroscopic reversibility, however, I could not find any experimental or computational evidence that supports the existence of the nitrogen radical, as well as its capability to undergo HAA. Given that this mechanism is the foundation of the work’s novelty and claim to an “unprecedented” strategy, it is imperative that the authors provide direct evidence for its validity. Notably, all the seminar work cited by the authors (ref. 14, 18, 48-52) have experimental and/or computational evidences for the key intermediates involved. Substantial additional work is required to address the concerns below.

Detailed comments:
1) There is no experimental or computational evidence to support the proposed mechanism. The authors should conduct spin population analysis to check if nitrogen radical exists or not. If so, what are the BDEs of the N-H bond and the C-H bond of the substrate? These data could indicate whether the proposed HAA by the nitrogen radical is thermodynamically favorable. Furthermore, low-temperature EPR experiments should be attempted to experimentally detect the nitrogen radical in the absence of substrate.

2) As noted by the author, HAA by the thiyl radical is also possible. However, the authors excluded this possibility based on the relative stability of the two tautomers. This argument is not supported by the Hammond postulate as HAA reactions are generally exergonic processes that involve early transition states, that is, the stability of the products has little influence on the selectivity of HAA reactions. The authors should provide more solid evidences to prove/disprove the alternative pathway —HAA by the thiyl radical and HAD by the N-H bond.

3) The authors claim that both HAA and HAD are enantioselective and the overall enantioselectivity is the synergistic effect of the two steps. However, neither selectivity is experimentally determined. Therefore, it's unknown which step is more important in stereocontrol. In the first round of review, Reviewer 2 also mentioned that the authors should “deconvolve the respective contributions of the claimed enantioselective HAA and HAD”. Notably, most of the seminal works cited by the authors have experimentally determined at least one of the two selectivities, providing valuable insights for future catalyst development. This is particularly important for an unprecedented deracemization system.

4) The interpretation of the experiments using excess PySH-6 or thiophenol is still confusing (Fig. 5G and SI, P74). The authors proposed an in-cage HAA/HAD process, , yet addition of 20 mol% of PySH-6 significantly influence the er of the product. How does PySH-6 influence the in-cage, quasi-intramolecular HAD step?

5) In the first round of review, this reviewer suggested that several closely related works should be cited. However, none of them was cited in the revised manuscript, even the very related work where a chiral phosphate and a non-chiral aryl thiol was utilized for photochemical deracemization (CCS Chem. 2026, doi: 10.31635/ccschem.025.202506718). I understand that the authors did some experiments and claimed that their mechanism is different, but that does not mean this reference should be excluded. I am aware that this journal has some guidelines on the number of citations, but I don't think the editor would ask the authors to exclude closely related references merely to keep the citation count within a certain limit. If the authors are confident about their novelty, they should cite and discuss this work in the introduction/Fig. 2, and then highlight their own novelty via subsequent mechanistic evidences. Otherwise, the readers would be confused as to why the current work deserves publication in Nature when a very similar system has already been published. For the rest suggested references, the authors tried very hard to find various reasons to exclude them. They also explained why their 5-membered, N-CONHPh, 2-aryl pyrrolidine substrates are not limited--because electron-rich and electron-deficient as well as bulky aryl substituents are tolerated. Notably, even the supportive “Reviewer 1” mentioned that “This critique is a little too broad, and could easily be applied to this work too”, indicating that the current work is also limited in scope.

6) To better define the scope and limitations of the current approach, the results of 2-alkyl/4/6-membered substrates should be added in the text and the SI. Currently, I only see these results in the authors' response letter.

In summary, this reviewer remains unconvinced that the current manuscript meets the high standard expected for Nature. However, if the authors can fully address the above issues, I may reconsider my position.

Open Access This Peer Review File is licensed under a Creative Commons Attribution 4.0 International License, which permits use, sharing, adaptation, distribution and reproduction in any medium or format, as long as you give appropriate credit to the original author(s) and the source, provide a link to the Creative Commons license, and indicate if changes were made.
In cases where reviewers are anonymous, credit should be given to 'Anonymous Referee' and the source.
The images or other third party material in this Peer Review File are included in the article's Creative Commons license, unless indicated otherwise in a credit line to the material. If material is not included in the article's Creative Commons license and your intended use is not permitted by statutory regulation or exceeds the permitted use, you will need to obtain permission directly from the copyright holder.
To view a copy of this license, visit https://creativecommons.org/licenses/by/4.0/

Point-to-Point response : Reviewer #1

Referee #1 (Remarks to the Author):

Comment 1: *In this manuscript, Zuccarello and coworkers describe the discovery of a creative and robust, photocatalytic strategy for enantioselective deracemization of 2-aryl pyrrolidines (with urea protecting groups). The ready access to a single enantiomer of these common drug motifs - and by such a simple, catalytic process - is truly impressive.*
Indeed, the concept is quite well explored, optimized, and evaluated with a range of interesting,

drug like pyrrolidines being deracemized or even interconverted. The selectivity is consistently high across these examples - illustrating high robustness and utility.

Response 1: We would like to express our gratitude to the referee for the generous evaluation of our work. Following the referee´s suggestions, we have conducted detailed experimental investigations to complement the study with a deeper mechanistic foundation. We sincerely hope that these address the points raised by the referee (please see below). We fully agree that this elevates the impact of the work.

Comment 2: *The major critique is the likely flawed understanding of the mechanism. Conceptually, in the Intro (in Fig 1C) and later again in the proposed mechanism (Fig 5H), a proposal is put forth that would violate the principle of microscopic reversibility. Specifically, if a chiral catalyst favors HAA of one enantiomer (e.g. R over S) than the microscopic reverse (HAD) between that same catalyst and substrate pair would dictate the same enantiomer should be formed (returning again to R over S). The very few examples of deracemizations have dealt with this paradox by separating the mechanisms of radical generation versus trapping. For example, instead of HAA by the chiral catalyst, the Ir could directly oxidize the amine (followed by rapid loss of the alpha proton to afford the net HAA product). Based on the Stern-Volmer quenching studies, the authors ruled out such a pathway (despite its satisfactory avoidance of the microscopic reversibility paradox). However, I urge them to consider testing other quenching combinations in order to find an alternate proposal that does not have the same catalyst performing both HAA and HAD (since only one diastereomeric transition state can be lower, and not alternating between R and S). Possibilities may simply include solubilization by CPAK ion pair and an achiral oxidation by Ir (then deprotonation by PyS) - for an overall racemic HAA, followed by enantioselective HAD. Or if HAA is selective, than HAD could be racemic, but this would likely take longer to deracemize.*

Response 2:

We thank the reviewer for raising this important question. We conducted additional experiments to investigate this mechanism. To confirm that the chiral HAT assembly formed from CPAs and PySH in the presence of base operates via enantioselective HAA we have performed further Stern-Volmer luminescence quenching studies, analyzing a 1:1:1 mixture of a triple combination of (*S*)-CPAK, PySK-**2** and matched (*S*)-**1**, and a 1:1:1 mixture of a triple combination of (*S*)-CPAK, PySK-**2** and *mis*matched (*R*)-**1**. (see **response 12** for details). We found that the matched combination quenches the excited state of the Iridium photocatalyst 50% faster than the mismatched combination (data added in SI). This is in line with the deuterium erosion studies with enantiopure deuterated substrates (Fig 5F). Adding a solubilizing agent (racemic CPAK) to the quenching study does not alter the quenching activity of rac-**1** (see **response 11** for details).

We have also performed deeper mechanistic studies to assess whether HAD (back-HAT) occurs in- *or* out-of-cage. The cross-over experiment in Fig. 5F suggests that HAD occurs in-cage as no deuterium incorporation was observed in the non-deuterated product **3**. This contrasts with previous reports by Bach (*J. Am. Chem. Soc.* **2021**, *143*, 21241.). We validated our cross-over experiment with time-course experiments showing that rac-**3** undergoes optical enrichment on a comparable timescale to the standard substrate, indicating that enantioenrichment is not instantaneous (see **response 6** of reviewer #4). Thus, a certain degree of deuterium incorporation is expected in a potential out-of-cage HAD over the given timescale. Therefore, HAD occurs prior to substrate-catalyst dissociation inside the chiral microenvironment provided by the catalyst.

Overall, our additional mechanistic studies have reinforced our previous scenario in which a single chiral HAT assembly orchestrates enantioselective HAA and HAD. However, as correctly pointed out by reviewer #1 this would lead to a racemic equilibrium, unless the assembly realizes these microscopically reverse elemental steps via *distinct pathways*.

We have therefore revisited our deuterium erosion experiment with *rac*-**1**-d_1 (Fig. 5F) and analyzed the reaction via high resolution mass spectrometry (HRMS). We observed mono- and di-incorporation of deuterium in PySK-**1** (see **response 16**). When performing the same experiment in benzene instead of toluene, the same degree of deuterium scrambling is observed excluding solvent-mediated H/D-exchange (See supplementary information). This finding confirms that D/H exchange occurs with PySK-**1**. Thus, upon deuterium atom abstraction from *rac*-**1**-d_1 deuterium incorporation in PySK-**1** must proceed via tautomerization prior to back-HAT which accounts for the observed deuterium erosion. In literature, the thione-thiol tautomerism of 2-mercaptopyridine is well-documented and in non-polar solvents the equilibrium favors the thione tautomer (*J. Org. Chem.* **2002**, *67*, 9061).

We hypothesized that the thione-thiol tautomerism may open to a scenario in which HAA and HAD are performed by the two distinct tautomeric forms. Thus, HAA can occur at two sites of the delocalized chiral radical **B** (N vs. S) (Fig 5H) (*Inorg. Chem.* **2003**, *42*, 8494). This selectivity is dictated by the formation of the more stable tautomer after the abstraction, being the thione in non-polar solvents such as toluene (*J. Org. Chem.* **2002**, *67*, 9061). The resulting thione tautomerizes to the thiol allowing enantioselective HAD. Therefore, the two microscopic reverse reactions can occur via distinct diastereomeric transition states.

We found in literature that the thione-thiol tautomerization can be induced by light irradiation via excited state proton transfer (ESPT). Although this is a well-documented process in the realm of physical chemistry occurring for 2-mercaptopyridine (*Struct. Dyn.* **2017**, *4*, 044021; *J. Phys. Chem. B* **2011**,*115*, 8266; *Chem. Eur. J.* **2019**, *25*, 1733.; J. Phys. Chem. 1990, 94, 7406; *Photochem. Photobiol.* **1994**, *60*, 442.; *J. Chem. Phys.* **2025**, *163*, 234313; *International Reviews in Physical Chemistry*, **2022**, *41*, 1.) at the time of our submission we were not aware of it.

[Redaction]
Figure Includes
Third Party Material

To corroborate whether such photo-tautomerization is possible under our reaction conditions we recorded UV-Vis spectra of PySH-**6** (present as the thione tautomer) and a 1:1 mixture of (*S*)-CPAK and PySH-**6**. Both analyses showed absorption in the range of light source we irradiated for our reaction. Thus, the thione-thiol photo-tautomerization occurs under our reaction conditions and this is consistent with the observed deuterium incorporation in PySK-**1**.

UV-Vis of 5-methylpyridine thiol (PySH-6) and (*S*)-CPAK

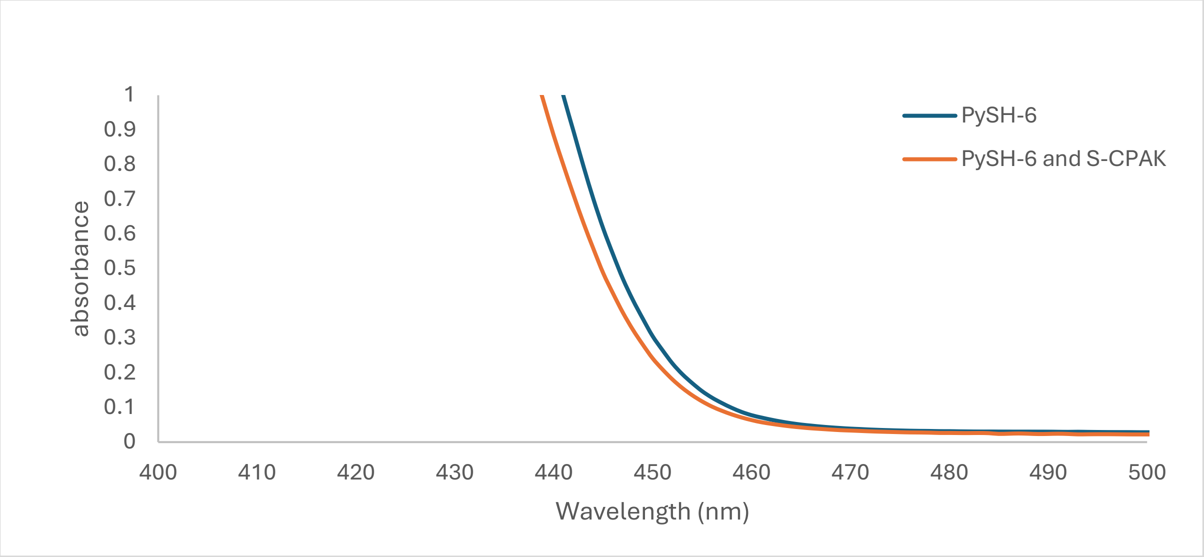

Based on this new mechanistic evidence and literature support we propose a revised mechanism.

The resubmitted manuscript now states:

*"Our experimental evidence suggests that a single HAT assembly formed in situ from CPAs and PySH achieves photochemical deracemization of 2-aryl pyrrolidines inducing enantioselectivity in both HAA and the microscopic reverse HAD. This scenario however would result in a racemic mixture,[47] unless the two elemental steps occur via distinct diastereomeric transition states. A plausible mechanistic proposal is depicted in Fig. **5H**. Under light-irradiation, the excited state of the iridium photocatalyst engages in electron transfer with in situ-generated chiral HAT assembly **A** and generates delocalized chiral radical in **B**, that undergoes HAA at one enantiomer of the racemic mixture to give a carbon-centered radical. Importantly, HAA can occur at two sites of the delocalized chiral radical **B** (N vs. S).[60] This selectivity is dictated by the formation of the more stable tautomer after the abstraction, being the thione in non-polar solvents[61]. Under photoirradiation, thione-phosphate complex **C** converts into thiol tautomer **D** via rapid excited state proton transfer.[62,63] The deuterium incorporation in PySK-**1**[58] previously observed (See Fig. 5F and the supplementary information) indicates that such photo-tautomerization takes place under the standard reaction conditions. Thus, thiol tautomer **D** promotes stereoselective HAD to the carbon-centered radical affording enantioenriched product and thiyl radical **E**. A subsequent electron transfer event with the reduced photocatalyst regenerates chiral assembly **A**. The two-component assembly essentially acts as a single chiral HAT catalyst inducing enantioselectivity in both independent steps (HAA and HAD) proceeding through two distinct reactive sites of 2-mercaptopyridine (N vs. S). The thione-thiol photo-tautomerization thus enables the two elemental steps to proceed through two distinct diastereomeric transition states."*

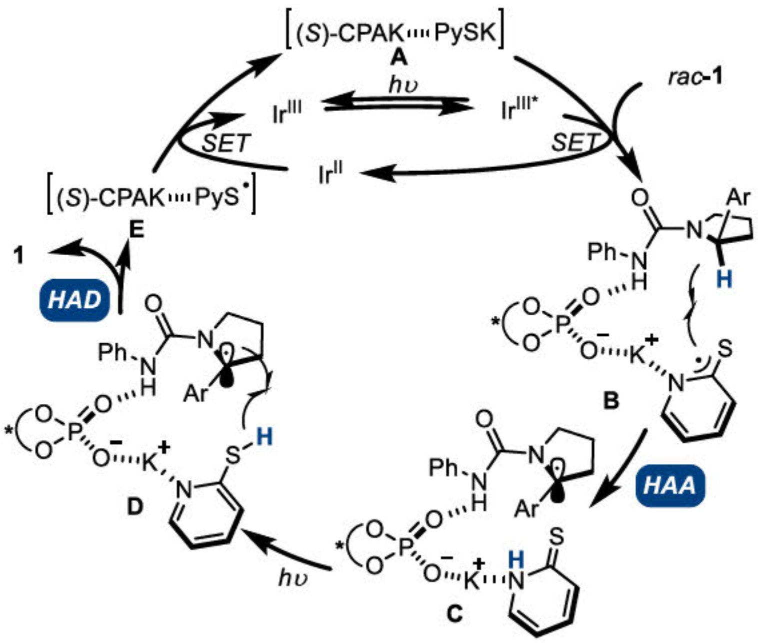

Irradiation for 1 h followed by prolonged stirring for 19 h in the dark gave an enantiomeric ratio of 52:48 er which is in line with our time-course experiment (Fig. 5A). The deracemization therefore proceeds under non-equilibrium photochemical conditions. We believe that the revised manuscript provides a mechanistically rigorous and conceptually clarified scenario of enantioselective hydrogen atom relay catalysis.

Comment 3: *Overall, the authors have done a thorough job describing the reaction development, mechanistic elucidation, excellent scope of reactivity, and synthetic utility. It is a highly scholarly work, including a thoughtful discussion and well-done SI, which is exceptionally detailed and the authors should be commended for this thorough effort.*

Response 3: We thank reviewer #1 for the appreciation of our work and acknowledge the positive evaluation of the Supplementary Material.

Comment 4: *Publication is highly recommended upon addressing the major critique described above, as well as the following minor comments.*

Response 4: We are very grateful for the recommendation for publication in Nature and the compliments. The constructive review has improved the quality of our study.

Minor Revisions:

Comment 5: *Intro - Please fix this text: "Despite seminal work by … through reversible C(sp3)–H cleavage have only been realized with highly specialized substrates." This critique is a little too broad, and could easily be applied to this work too. Perhaps merely mentioning that those seminal works exist might be a better strategy.*

Response 5: We thank the referee for the suggestion to change the indicated sentence in the introduction of our manuscript. We agree that it may be a better strategy to mention the seminal works and we have done so. The revised manuscript now states: *"Photochemical deracemization is emerging as a powerful strategy to convert a racemic mixture into enantioenriched material. Seminal work has been reported by Bach, Knowles and Miller, Luo, Gilmour, Zuo, and Fu and Liu."*

Comment 6: *Figure 3 - Use arrowheads to indicate progression of the optimization. The end(?) in Box 3 is particularly confusing.*

Response 6: *We are grateful for this suggestion to better highlight the progression of the optimization using arrowheads. We have adopted this change. At the end of Box 3 we summarized* the final optimized reaction conditions and have noted the yield and enantioselectivity of the deracemization with standard substrate.

Comment 7: *Figure 4 - These scope studies are very well done. Excellent choices of widely ranging and useful molecules.*

Response 7: We very much appreciate the compliments by the referee and are motivated to keep the quality of our studies constantly high in the future. Thank you very much.

Comment 8: *Figure 5 - Great mechanistic section. Minor additions:*

Response 8: We thank the referee for the encouraging comments and thorough review.

Comment 9: *Fig 5a: Indicate which catalyst is used*

Response 9: For the time course experiments, we have used the standard conditions in Fig. 4A. We have added this information to the caption of Fig. 5A and detailed the procedure in the SI.

Comment 10: *Fig 5b: >1:99 should be <1:99 (or >99:1 enantiomer)*

Response 10: Thank you for highlighting this mistake. We have corrected it according to the suggestion.

Comment 11: *Fig 5c: Quenching: Include a solubilizing agent for the substrate probe (e.g. rac-CPAK or an achiral biphenyl phosphate).*

Response 11: Thank you for the suggestion. We have conducted luminescence quenching experiments with a prestirred, 1:1 solution of *rac*-CPAK and *rac*-**1** These studies have been included in the SI.

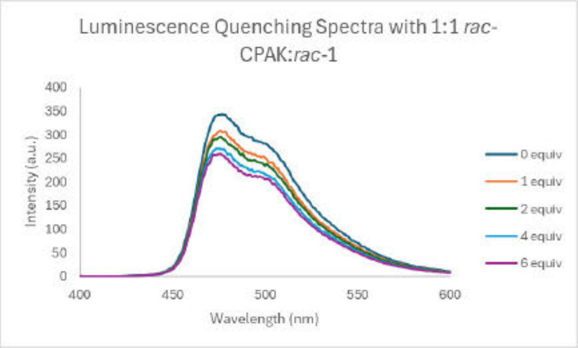

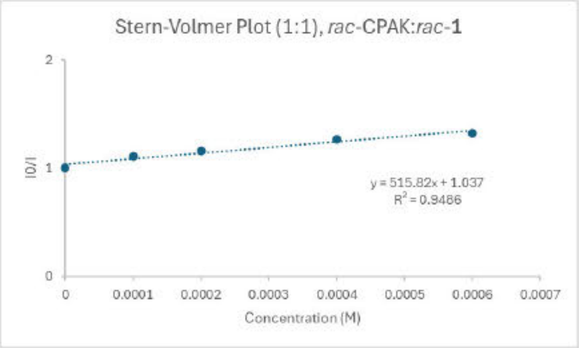

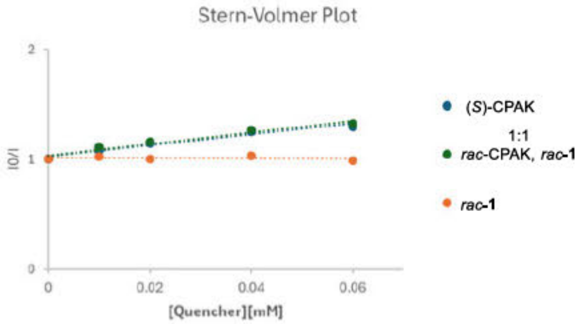

As stated in the supplementary information: "Stern-Volmer Luminescence analysis of a 1:1 combination of *rac*-CPAK and *rac*-**1** revealed the same quenching activity as (*S*)-CPAK. Thus, the addition of *rac*-**1** does not alter the quenching activity of (*S*)-CPAK. This is consistent with our previous observation that the substrate does not show any quenching activity."

Comment 12: *Repeat with the matched vs mismatched pairs to probe HAA vs HAD. Consider a triple combination (1:1:1) to probe the proposed B in the mechanism.*

Response 12: Thank you for the suggestion. We have conducted luminescence quenching with a prestirred, 1:1:1 solution of (*S*)-CPAK, PySK-**2** and matched (*S*)-**1** 1:1:1 solution of (*S*)-CPAK, PySK-**2** and mismatched (*R*)-**1**. These studies have been included in the SI.

Luminescence quenching with a prestirred, 1:1:1 solution of (*S*)-CPAK, PySK-**2** and (*S*)-**1**

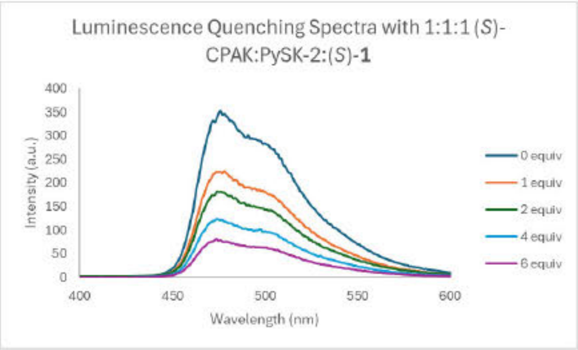

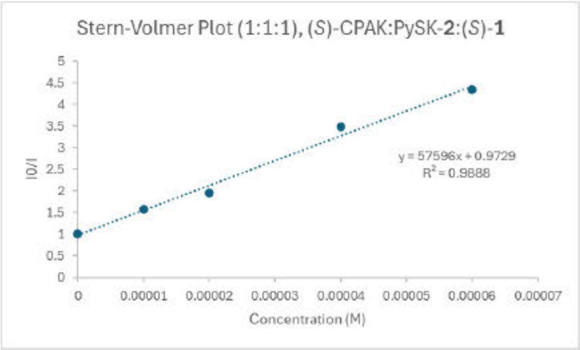

Luminescence quenching with a prestirred, 1:1:1 solution of (*S*)-CPAK, PySK-**2** and (*R*)-**1**

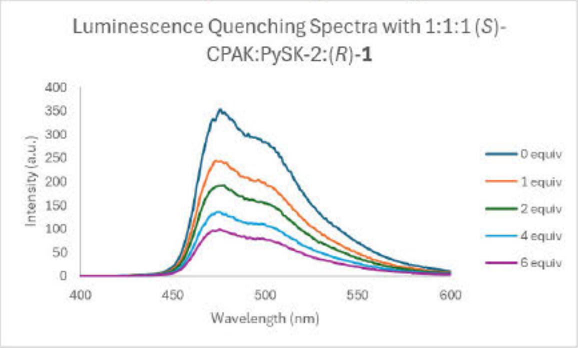

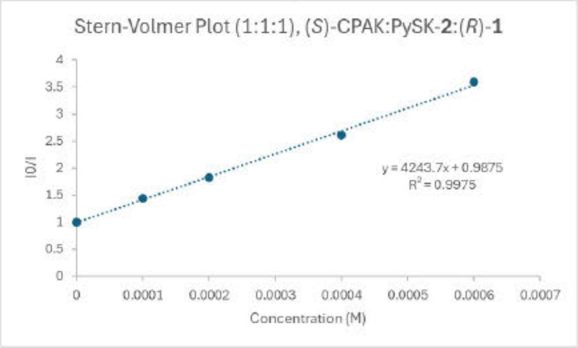

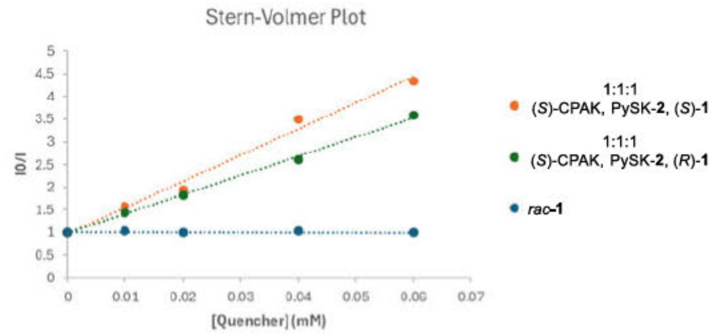

Stern-Volmer luminescence analysis of a triple combination (*S*)-CPAK, PySK-**2** and (*S*)- or (*R*)-**1** revealed a faster quenching activity for the matched (*S*)-**1** substrate. This is consistent with a selective HAA at one enantiomeric substrate.

Comment 13: *The structure of PySK-2 is out of place and confusing.*

Response 13: We moved PySK-**2** to Fig. 5B.

Comment 14: *Fig 5e: only R = Me is 29, H = 1*

Response 14: Thank you for highlighting this mistake. We have corrected it accordingly.

Comment 15: *Fig 5f: what rates are being measured? Isomerization? Are these parallel experiments? Please indicate.*

Response 15: We apologize for not clearly communicating that we have measured rates of racemization of enantiopure compounds in parallel experiments. We have added a comment in the caption of Fig. 5F. We have also clarified it in the main text which now states: "*A primary kinetic isotope effect (k_H/k_D) of 1.86 was found by measuring the initial rates of racemization of (S)-**1** and (S)-**1**-d_1 in parallel experiments (data added in SI).*"

Comment 16: *On the right side of this box (D scrambling), where is the H from? Perhaps the urea NH? Pleased indicate.*

Response 16: We acknowledge the reviewer for this comment. The D/H exchange can take place with the N-H proton of the urea. We think that the exchange is also occurring with PySK-**1**. The literature supports this hypothesis (*Can. J. Chem.* **1989**, *67*, 5.). We have mentioned this

hypothesis in the main text: "*We observed that ca. 20% deuterium scrambling occurred when rac-**1**-d_1 was subjected to photochemical deracemization and **1**-d_1 is recovered with good enantiomeric ratio, indicating that reversible deuterium atom transfer is operative upon deuterium incorporation in PySK-**1**.*"

We have revisited our deuterium erosion experiment with *rac*-**1**-d_1 (Fig. 5F) and analyzed the reaction via high resolution mass spectrometry (HRMS). We observed mono- and di-incorporation of deuterium in PySK-**1**.

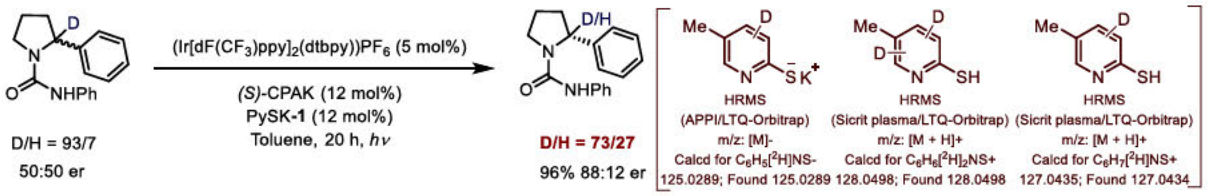

Comment 17: *Fig 5h: The progression of B > C > D violates the principle of microscopic reversibility. If B > C favors R consumption, then C > D would favor R re-generation, since it must be the same diastereomeric transition states (pro-R less energy than pro-S).*

Response 17: We thank reviewer #1 for raising concerns about our mechanistic proposal. This has encouraged us to take a closer look at our experimental data. We acknowledge that the previously proposed mechanism violates the principle of microscopic reversibility. We have addressed this concern (see **response 2**) with more in-depth mechanistic investigations and have provided a revised mechanism (see Fig. 5H). We believe that the new proposal is consistent with our experimental observations.

- Fig. 5H has been redrawn.
- In the revised mechanism, HAA and HAD are proposed to occur via two distinct diastereomeric transition states which is enabled by the photo-tautomerization of 2-mercaptopyridine.

<u>Point-to-Point response : Reviewer #2</u>

Referee #2 (Remarks to the Author):

Comment 1*: The work by Zuccarello and co-workers reports an enantioselective photochemical deracemization of 2-aryl pyrrolidines enabled by a non-covalent, in situ "assembled" chiral HAT catalyst formed from a chiral phosphoric acid and 2-mercaptopyridine. In contrast to previous studies, the central claim of the work relies on controlling both enantioselective hydrogen atom abstraction (HAA) and enantioselective hydrogen atom delivery (HAD). Conceptually, the combinatorial catalyst idea – assembled from commercially available precursors via non-covalent interactions – for this type of transformation is appealing, and the simplicity of the reaction design is elegant. Although currently limited to the 2-aryl pyrrolidines, the scope is fairly broad within the selected substrate class, with generally high yields and good to excellent er.*

Response 1: We thank reviewer #2 for their time to evaluate this manuscript and for their generally positive feedback. Their helpful comments and suggestions were thoroughly reviewed and addressed. Point-to-point responses to their comments are presented below.

Comment 2*: The mechanistic experiments are broadly consistent with the work's central claims but not fully conclusive. The kinetic isotope effect, together with Stern-Volmer studies, indicates that the reaction occurs via HAT. Deuterium scrambling experiments (including crossover experiments), especially with different enantiomers, are consistent with an in-cage HAA. However, the current evidence for the proposed non-covalent assembly is rather thin and indirect. To further substantiate the mechanistic proposal, the existence of the assembly should be supported by at least NMR experiments (e.g., titration/DOSY).*

Response 2: We thank reviewer #2 for this very valuable recommendation. We have performed a Job Plot analysis (data added in SI) with (*S*)-CPAK and PySK-**2** revealing that the two components assemble in solution as a 1:1 complex. This further strengthens our previous observation that a distinct chiral species is formed in catalysis as no racemization of (*S*)-**1** (matched enantiomer) is observed in the absence of either of the salts (Fig.5B).

Uv-Vis absorption curve for Job plot analysis solutions

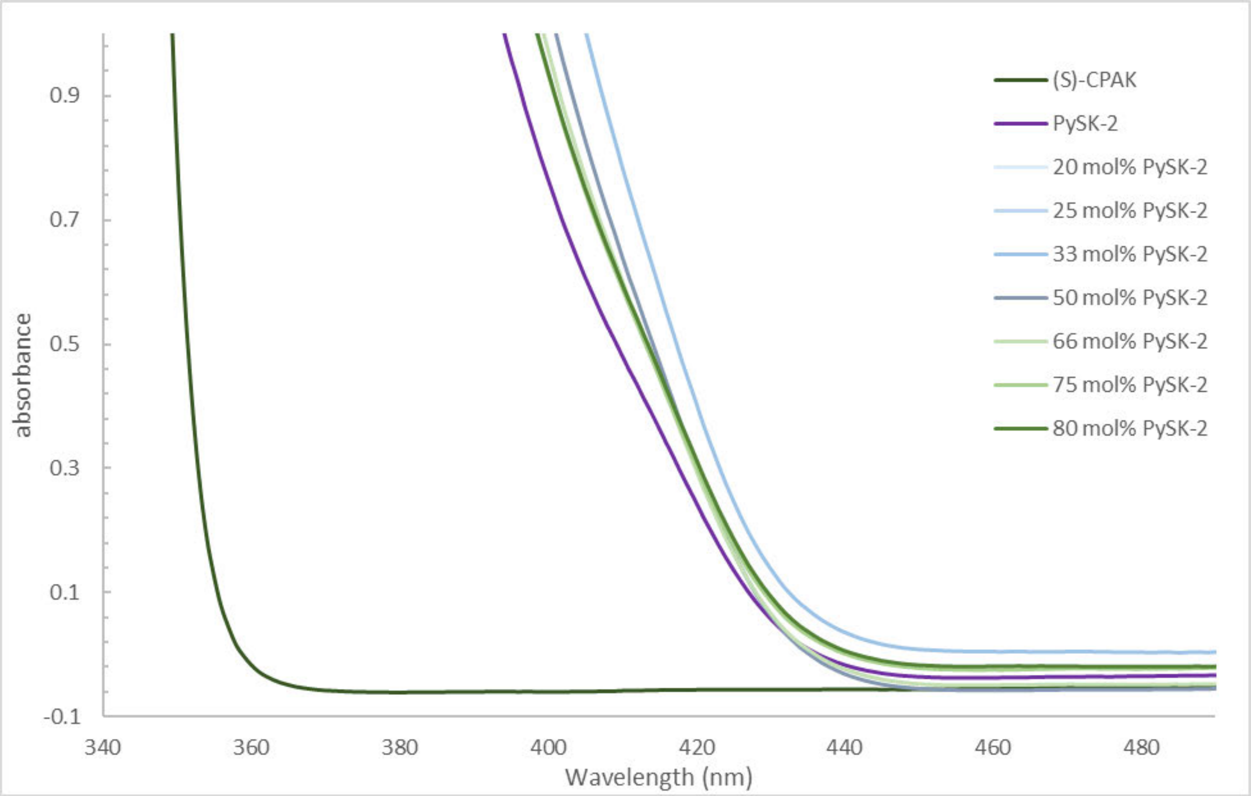

Job plot of $\Delta A_{obs}^{410\ nm}$•χ versus mole fraction of thiol (χ)

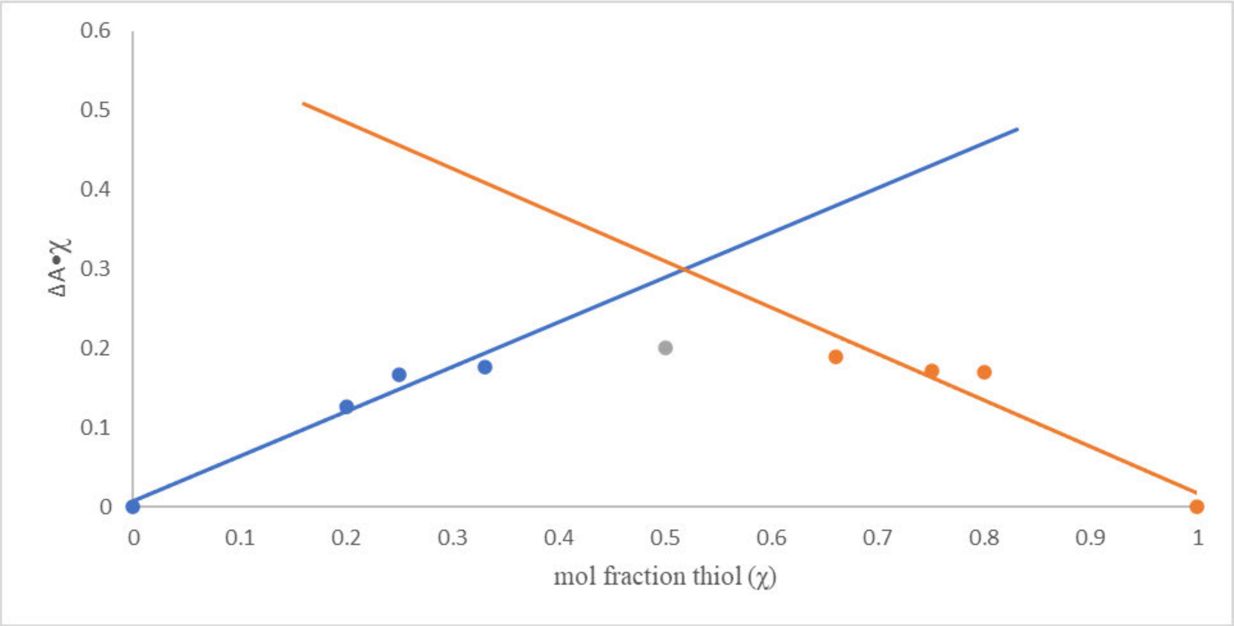

Comment 3: *Furthermore, the authors should attempt to deconvolve the respective contributions of the claimed enantioselective HAA and HAD.*

Response 3: We have attempted to capture the putative radical with acceptors to obtain an estimate of enantioselectivity arising from individual HAA and HAD. Because the photogenerated radical intermediates are short-lived, and the cross-over experiment (Fig 5F) suggests that HAD occurs prior to substrate-catalyst dissociation, intercepting the radical intermediate proved challenging. Attempting to intercept the radical intermediate with different acceptors did not yield detectable trapping products. In the case of two acceptors (vinyl sulphone and vinyl acetate) we observed substantial deracemization. This further reinforces an in-cage HAA/HAD process and the *intra*molecular HAD is faster than the addition reactions *inter*molecularly.

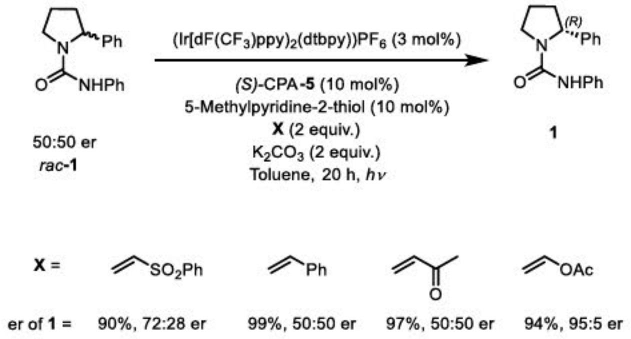

Comment 4: *Overall, the work describes a valuable transformation utilizing an appealing strategy. The manuscript currently relies heavily on a conceptual framework (non-covalently assembled chiral HAT catalyst, enantioselective control of HAA and HAD) that is not yet well supported. Addressing the points above – particularly direct evidence for the proposed assembly and deconvolution of HAA vs HAD – would substantially strengthen the manuscript. I therefore recommend revisions prior to publication.*

Response 4: We thank reviewer #2, for supporting publication of this work in *Nature*, for their constructive comments and suggestions. We hope we have addressed all the concerns.

Point-to-Point response : Reviewer #3

Referee #3 (Remarks to the Author):

Comment 1: *I co-reviewed this manuscript with one of the reviewers who provided the listed reports.*

Response 1: We thank reviewer #3 for evaluating our work.

<u>Point-to-Point response : Reviewer #4</u>

Referee #4 (Remarks to the Author):

Comment 1: *In this manuscript, Zuccarello and co-workers report the photochemical deracemization of 2-aryl pyrrolidines using a combination of a 2-mercaptopyridine and a chiral phosphoric acid. The authors proposed that this combination forms a "chiral thiol" in situ via non-covalent interactions, which then effects enantioselective HAA to break the stereogenic C–H bonds followed by enantioselective HAD to reform the C–H bonds. A series of mechanistic studies have been conducted. While the concept of forming a "chiral thiol" in situ is interesting, the proposed deracemization mechanism is fundamentally incorrect as it violates the principle of microscopic reversibility, which dictates that using one chiral catalyst to effect asymmetric induction in both bond-breaking and bond-making events via the same mechanistic pathway will always result in an overall equilibrium of racemate because of the shared energy surface (for reviews and highlights, see: Angew. Chem. Int. Ed. 2009, 48, 2648; Angew. Chem. Int. Ed. 2023, 62, e202308241; Science 2019, 366, 304; Science 2023, 382, 373). In other words, if the HAA step consumes R faster, then the HAD step catalyzed by the same thiol will reform R faster, therefore, it is impossible to achieve enantioenrichment via the proposed mechanism. Accordingly, the entire manuscript, including the related figures, should be rewritten/redesigned based on the correct mechanism.*

Response 1: We thank the reviewer for this important point. A new mechanistic proposal has been provided. We agree with the reviewer that "*using one chiral catalyst to effect asymmetric induction in both bond-breaking and bond-making events will result in an overall equilibrium of racemate,"* ***unless distinct reaction pathways can be realized by a single catalyst.*** In our updated mechanism HAA and HAD proceed through two distinct diastereomeric transition states. We have addressed this concern in full above. We respectfully refer reviewer #4 to **response 2** of **reviewer #1**.

Comment 2: *In addition, the similarity to known deracemization systems and the limited scope (detailed below) also make the manuscript unsuitable for publication in a top journal like Nature*.

Response 2: We believe the revised manuscript clarifies the conceptual distinction and broader significance of the present study. To our knowledge, this is the first example where: An achiral HAT catalyst (2-mercaptopyridine) is rendered effectively chiral via non-covalent interaction with a chiral phosphoric acid as a modular and interchangeable chiral element. This represents a distinct mechanistic paradigm with far-reaching implications. In this study chiral induction in radical HAT processes occur via *in situ* non-covalent catalyst assembly, rather than enantioselective protonation.

Mechanistic aspects

Comment 3: *As mentioned above, the proposed mechanism (Fig. 5H) will result in a racemic mixture. The fact that enantioenrichment was achieved suggests alternative mechanisms. A recent work utilized a very similar system (an achiral thiol and a chiral phosphoric acid) for deracemization of α-Aryl Ketones (CCS Chem. 2026, doi: 10.31635/ccschem.025.202506718). In their work, a non-selective HAA by an arylthiyl radical followed by enantioselective protonation by the phosphoric acid was proposed, circumventing the principle of microscopic reversibility. The*

authors should add this work in Fig. 1 and examine in detail to see if the current system follow a similar pathway.

Response 3: We appreciate the effort to propose an alternative mechanism for our transformation. Suggesting that an achiral thiol undergoes racemic HAA abstraction followed by enantioselective protonation such as in *CCS Chem.* 2026, doi: 10.31635/ccschem.025.202506718, is *inconsistent* with our mechanistic observations. When subjecting the matched enantiomer (*S*)-**1** to the reaction conditions in the absence of either (*S*)-CPAK or PySK-**1** leads to no loss in enantiopurity. Therefore, both salts are required for reactivity and enantioselectivity. This has been evidenced in Figure 5B. In addition, in Figure 3A we have shown that common HAD catalysts such as thiophenol and TRIP-SH do not provide enantioselectivity in our deracemization.

In the above-mentioned paper, α-aryl ketones are enolizable substrates and can therefore take advantage of enantioselective protonation such as in *J. Am. Chem. Soc.* **2025**, *147*, 15307. The 2-aryl pyrrolidine substrates are not enolizable and therefore fall under a completely different substrate category.

To directly evaluate the reviewer's alternative hypothesis (achiral thiol to thiyl radical that undergoes non-selective HAA), we performed control experiments using CPA + thiophenol with (*R*)-**1**, (*S*)-**1**, and *rac*-**1** under otherwise identical photochemical conditions. In all cases, we observe no conversion and no change in er. These experiments are now added to the SI. This demonstrates that thiophenol does not generate an operative achiral HAA pathway in our system, and therefore er erosion or deracemization cannot be rationalized by an "achiral thiyl HAA + CPA-controlled protonation" paradigm.

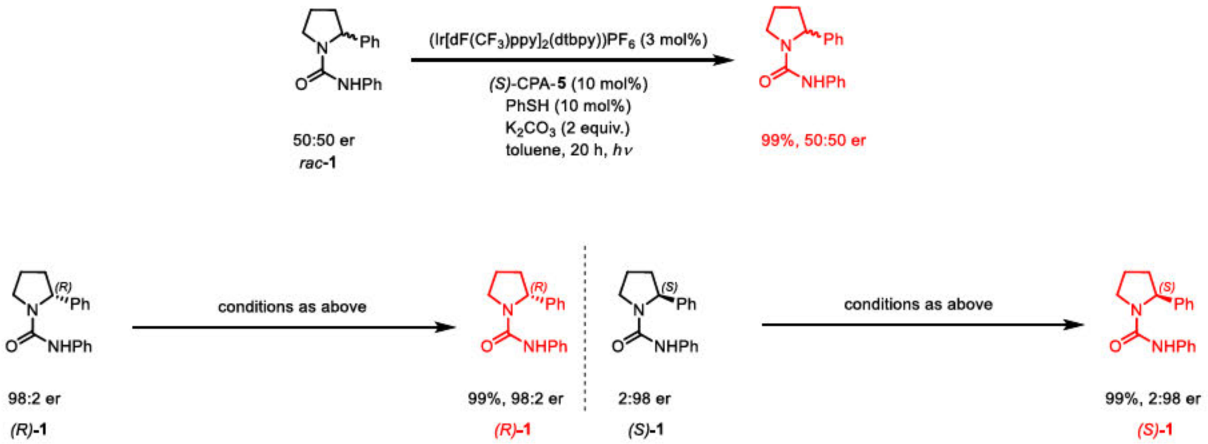

Comment 4: *2. In addition to the aforementioned work, chiral phosphate anions have recently been used in several photochemical deracemization reactions, however, these closely related works were not discussed or cited: J. Am. Chem. Soc. 2025, 14625; J. Am. Chem. Soc. 2025, 15307.*

Response 4: We appreciate the variety of strategies toward deracemization and also referenced relevant review articles, however, the constraints of the journal on the number of citations restricts the full discussion of this broader topic.

Chiral phosphoric acids have been used for enantioselective deprotonation/protonation and have been recently employed in photochemical deracemizations (*Chem. Commun.*, **2025**, *61*,

7737) relying on similar proton transfer mechanisms. These asymmetric deprotonation/protonation approaches are well established in the literature and have been extensively developed over the past two decades (see *Nature Chem* **2009**, *1*, 359). Importantly, however, the operative mechanism in those studies differs fundamentally from that in the present work. In view of our current mechanistic evidence the deracemization of 2-aryl pyrrolidines proceeds through HAT. In our study, the phosphate ion alone does not engage in the formation of the postulated intermediate radical such as in: *J. Am. Chem. Soc.* **2025**, 14625 and *J. Am. Chem. Soc.* **2025**, 15307. Our system does not involve enolate or carbanion intermediates. In our work, the chiral phosphate provides a modular interchangeable source of chirality to render the achiral HAT catalyst effectively chiral via non-covalent ion pair interaction. This is un unprecedent conceptual design of a chiral HAT catalyst.

Comment 5: *3. In Fig. 5G, the observation that added PhSH erodes enantioselectivity does not prove enantioselective HAD. An alternative explanation is that PhSH generates an achiral thiyl radical that performs non-selective HAA, thereby lowering ee. This possibility should be evaluated.*

Response 5: As mentioned above, when replacing PySH with PhSH, we observe no conversion and no change in er. Thus, the mechanism suggested by the referee is not plausible.

In addition, we have conducted experiments in the presence of excess PySH-**6**. Our observations were in line with the experiments performed with excess thiophenol (Fig. 5G). Importantly, replacing PySH-**6** with thiophenol under otherwise standard reaction conditions showed no erosion of enantiopurity with either (*S*)-**1** or (*R*)-**1**. Thus, thiophenol acts exclusively as an external HAD catalyst and does not interfere with the HAA step.

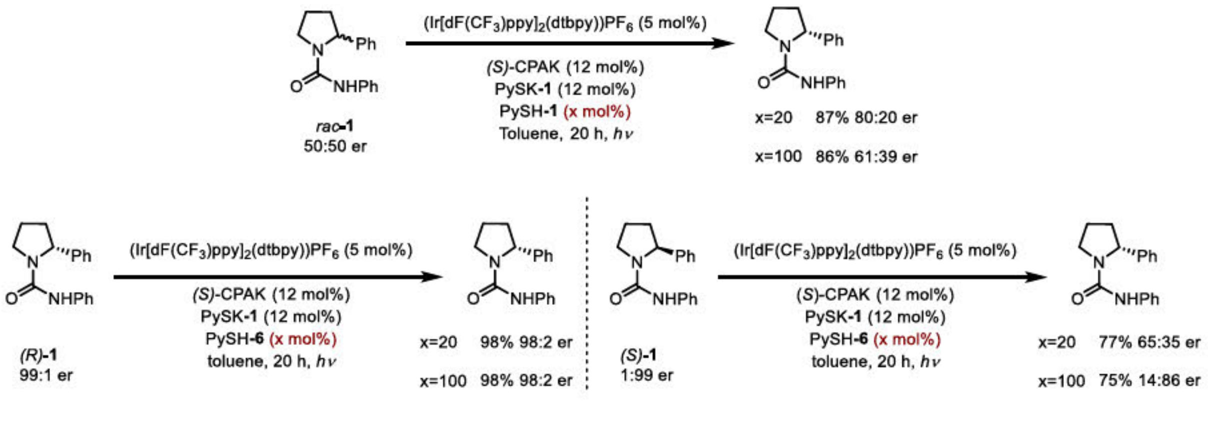

Comment 6: *In Fig. 5F, the low D/H exchange in the crossover experiment is interpreted as evidence for an in-cage HAA/HAD process. However, an alternative kinetic explanation exists: if non-deuterated rac-3 reacts much faster than rac-1-d1, the former could reach the photostationary state (95:5 er) before significant deuterium incorporation occurs. This alternative should be addressed.*

Response 6: We thank the reviewer for the thoroughness of the interpretation of the cross-over experiment. We have performed time-course experiments which show similar kinetic profiles

between *rac*-**3** and *rac*-**1**. Deracemization of *rac*-**3** is therefore not instantaneous. Consequently, a certain degree of deuterium incorporation is expected in a potential out-of-cage HAD over the given timescale. This contrasts with previous reports by Bach (*J. Am. Chem. Soc.* **2021**, *143*, 21241.). Therefore, HAD occurs prior to substrate-catalyst dissociation inside the chiral microenvironment provided by the catalyst.

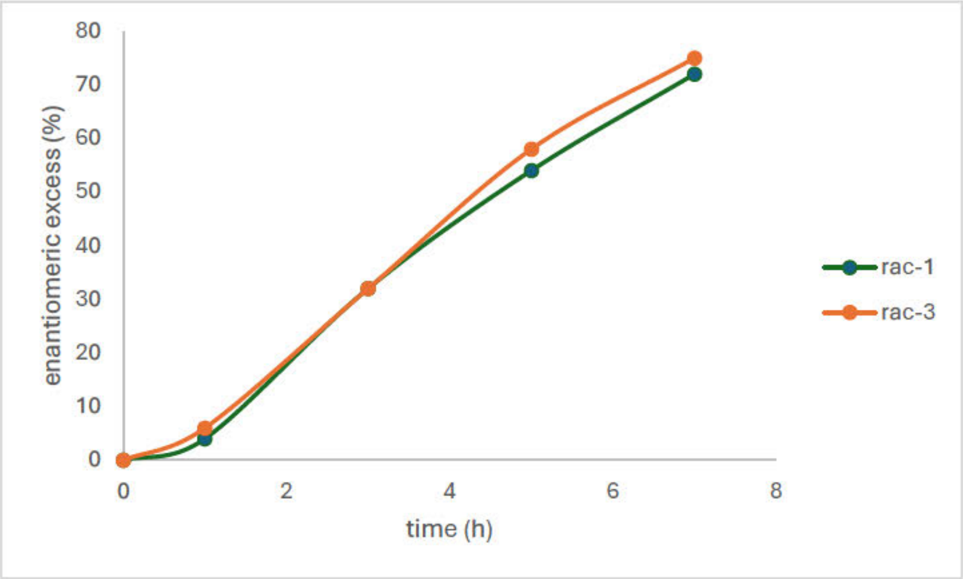

Substrate scope:

Comment 7: *1. The reaction requires substrates bearing a urea protecting group with a free N–H moiety, presumably to engage the chiral catalyst via hydrogen bonding. Furthermore, the stereocenter must be aryl-substituted. The generality of the method is therefore limited.*

Response 7: We thank the reviewer for this comment and agree that substrate generality is an important consideration. We have focused on 2-aryl pyrrolidines because of their central role in pharmaceuticals and thus, are an important class of substrates on their own (see *Nat Biotechnol* **2017**, *35*, 694.; *Eur. J. Med. Chem.* **2017**, *130*, 15.; *Bioorg. Med. Chem. Lett.* **2010**, *20*, 1436. and *J. Med. Chem.* **2016**, *59*, 9337.) In addition, they are used in chiral amine ligands and organocatalysts. The *direct catalytic photochemical deracemization of this scaffold* constitutes a significant impact in synthetic organic chemistry.

To our knowledge, prior photochemical deracemization studies have largely focused on α-Aryl ketones, Lactams, Enolizable carbonyl compounds, Ester derivatives.

Direct catalytic deracemization of cyclic amines bearing a stereogenic C(sp^3)–H bond adjacent to nitrogen remains limited, and the few reported examples predominantly focus on alkyl-substituted stereocenters, typically with limited scope.

Thus, the current work addresses a substrate class that is both synthetically important and underexplored in the context of reversible HAT–based deracemization.

The free N–H functionality is mechanistically purposeful as it enables defined hydrogen-bonding interactions within the catalyst assembly and it facilitates controlled positioning of the substrate relative to the chiral phosphate. Importantly, the urea group serves as a readily removable protecting group (see Fig. 3B, Box 4) and does not represent a synthetic dead-end.

Comment 8: *To strengthen the study, the authors should explore other amine derivatives, including those with different ring sizes, and examine alkyl-substituted stereocenters.*

Response 8: Upon suggestion of the referee, we have concentrated our efforts also on expanding the class of substrates that can undergo photochemical deracemization under the current optimized reaction conditions. [redacted]

[redacted]

[REDACTION]
Includes Information for Future Publication

[redacted]

[redacted]

[redacted]

[redacted]

[redacted]

Comment 9: *2. The claim on page 5 (lines 85–89) that "successful photochemical deracemizations through reversible C(sp^3)–H cleavage have only been realized with highly specialized substrates" is an overstatement and overlooks several pertinent examples, for example, pyridylketones (J. Am. Chem. Soc. 2021, 13393), δ-and γ- lactams (J. Am. Chem. Soc. 2025, 1186), α-, β-, and γ-arylated ketones and esters (J. Am. Chem. Soc. 2025, 14625; J. Am. Chem. Soc. 2025, 15307; CCS Chem. 2026, doi: 10.31635/ccschem.025.202506718). These works should be cited and discussed to provide a balanced view of the field. The authors should also clarify what defines a "highly specialized substrate" and explain why their own substrates, which possess a specific hydrogen-bond donor, are not considered as such.*

Response 9: We agree that it may be a better strategy to mention the seminal works and we have done so. The revised manuscript now states: *"Photochemical deracemization is emerging as a powerful strategy to convert a racemic mixture into enantioenriched material. Seminal work has been reported by Bach, Knowles and Miller, Luo, Gilmour, Zuo, and Fu and Liu."*

The deracemization of pyridylketones (*J. Am. Chem. Soc.* **2021**, *143*, 13393) requires the pyridyl group for it to occur and cannot be cleaved afterwards. The report on the deracemization of δ- and γ-lactams (*J. Am. Chem. Soc.* **2025**, *147*, 1186) shows only few entries of deracemization while most of the substrate scope is an enantioselective deuteration. Additionally, the substrates need to be *N*-arylated. In view of the underlying mechanism, the deracemization of α-, β-, and γ-arylated ketones and esters is restricted to electron-rich aryl substituents (*J. Am. Chem. Soc.* **2025**, 147, 14625; *J. Am. Chem. Soc*. **2025**, *147*, 15307.) or require an enolizable stereocenter (CCS Chem. 2026, doi: 10.31635/ccschem.025.202506718).

In our substrates electron-rich and electron-deficient as well as bulky aryl substituents are tolerated on a general molecular scaffold such as pyrrolidine. The free amine can be obtained upon deprotection.

Comment 10: *Overall, the manuscript presents an interesting catalytic system for photochemical deracemization, but the proposed mechanism is not supported by fundamental principles and requires substantial revision. Furthermore, the substrate scope is limited, and the discussion lacks appropriate context from recent literature. Once these issues are properly addressed, this work should be suitable for a more specialized journal whose readers could better appreciate the subtle mechanistic difference to the known deracemization systems.*

Response 10: We thank the reviewer for this overall assessment and for recognizing the catalytic system as interesting. With these revisions, we believe the manuscript now clearly establishes a mechanistically distinct and far-reaching paradigm for enantioselective HAT. While substrate breadth can continue to expand in future work, the present study establishes a new conceptual mode of stereocontrol in radical chemistry. By non-covalent catalyst assembly we can render an achiral HAT catalyst effectively chiral. We therefore believe the revised manuscript addresses the reviewer's concerns.

Point-to-Point response : Reviewer #1

Referee #1 (Remarks to the Author):

Comment 1: *I remain strongly in favor of publication of this important synthetic contribution, and I commend the authors for thoroughly addressing all the reviewers' comments to the best of their ability.*

However, I also remain skeptical that the new mechanism addresses the key issue concerning the principle of microscopic reversibility.

As drawn now, B to D are still the intermediates that would contribute to the same transition state. The proposed thione-thiol photo-tautomerization (drawn in between) is a reasonable complementary mechanism, but seems irrelevant to the key, shared transition state. The issue is that this T.S. is shared, not that there is a separate tautomerization possible. (To be fair, I'm not totally convinced that the radicals would wait around for another photon to excite them and mediate this isomerization, but that is beside the point).

Overall, I empathize with the authors that it is hard to rationalize the Stern-Volmer quenching studies, which caused them to rule out a more "typical" Ir oxidation of the amine (followed by alpha proton loss to afford the "net" HAA product) that conforms with the principle of microscopic reversibility. However, I think it is simply an issue of solubility (conveniently provided by the self-assembled catalyst complex), which I hoped they would find a solution to via the new triple combination S-V experiments. Thus, while they didn't figure out this solubility issue (heterogeneity is a well-known issue in the study of kinetics), I am not sure that the overly complex (and still seemingly not quite right) explanation provided is needed.

Anyways, this is merely my strong opinion, but the authors are free to choose to pose whichever mechanistic hypothesis they believe the data is suggesting. Either way, I do NOT believe it negates from the synthetic achievement of this privileged (in med chem especially) pyrrolidine deracemization (see their response to Reviewer 4 about the difference in substrate classes now possible versus previous carbonyl-based deracemizations). And I think the claims and analysis of the experiments are all sound, and they may very well contribute to a healthy discourse in the future scientific literature.

Response 1: We thank the reviewer for finding our work suitable for publication in *Nature* and for appreciating the quality of our revisions.

Point-to-Point response : Reviewer #2

Referee #2 (Remarks to the Author):

***Comment 1**:* The authors have satisfactorily addressed most of the concerns, including those of the other reviewers. The Job´s plot provided cannot be used to derive a stoichiometry; it rather indicates that there is a multiple step equilibrium and not the formation of a 1:1 assembly. Despite some remaining doubts on the mechanism I consider the manuscript suitable for publication.

Response 1:

We thank reviewer #2 for the positive assessment of our work and for considering the manuscript suitable for publication in *Nature*.

Point-to-Point response : Reviewer #3

Referee #3 (Remarks to the Author):

Comment 1: *I co-reviewed this manuscript with one of the reviewers who provided the listed reports.*

Response 1: We thank reviewer #3 for evaluating our work.

Point-to-Point response : Reviewer #4

Referee #4 (Remarks to the Author):

Comment 1: *In the revised manuscript, the authors corrected their mechanism based on the photo-tautomerization of 2-mercaptopyridines, proposing that a nitrogen radical undergoes HAA while the S-H bond is responsible for HAD. This proposal indeed circumvents the principle of macroscopic reversibility, however, I could not find any experimental or computational evidence that supports the existence of the nitrogen radical, as well as its capability to undergo HAA. Given that this mechanism is the foundation of the work's novelty and claim to an "unprecedented" strategy, it is imperative that the authors provide direct evidence for its validity. Notably, all the seminar work cited by the authors (ref. 14, 18, 48-52) have experimental and/or computational evidences for the key intermediates involved. Substantial additional work is required to address the concerns below*

Response 1: Thank you for a thorough reflection on the revised mechanism. We truly appreciate your effort. The proposed mechanism is based on solid experimental evidence, and it is supported by literature. The novelty of our work goes *beyond* the proposed mechanism as also agreed by reviewer #1. This includes that by pairing chiral phosphate ions with commercial 2-mercaptopyridines, we can render an achiral HAT catalyst effectively chiral and thereby access a previously inaccessible space of chiral HAT catalysts and the photochemical deracemization of pharmaceutically relevant scaffolds such as 2-aryl pyrrolidines.

Our experimental evidence suggests the formation and involvement of a chiral assembly shaped by non-covalent interactions between a chiral potassium phosphate ion, the potassium salt of 2-mercaptoypridine and the substrate in the enantioselective steps. Thus, embarking in a theoretical study of such a complex system represents a separate future project.

Detailed comments

Comment 2: *1) There is no experimental or computational evidence to support the proposed mechanism. The authors should conduct spin population analysis to check if nitrogen radical exists or not. If so, what are the BDEs of the N-H bond and the C-H bond of the substrate? These data could indicate whether the proposed HAA by the nitrogen radical is thermodynamically favorable. Furthermore, low-temperature EPR experiments should be attempted to experimentally detect the nitrogen radical in the absence of substrate.*

Response 2: We thank the reviewer for the effort to further clarify our mechanistic proposal.

The invoked radical of 2-mercaptopyridine is more accurately described as *delocalized* rather than a *nitrogen- or sulfur*-centered radical species as established in the literature (*Inorg. Chem.* **2003**, *42*, 8494). Assigning the site of HAT based on spin population analysis of the delocalized 2-mercaptopyridine radical is ***not*** meaningful to a more complex system such as ours and thus, cannot be used to rationalize a "N- vs. S-centered" HAA. Control experiments (Fig. 5B of the manuscript) have established that HAA abstraction requires the chiral potassium phosphate and the potassium salt of 2-mercaptopyridine to assemble via a network of non-covalent interactions to effectively occur. Consequently, spin population analysis of the free radical is not representative of the catalytically relevant species.

Literature supports the existence of *radical character on the nitrogen atom* as these delocalized radicals can be photogenerated through homolytic N–O bond cleavage of *N*-Hydroxypyridine-2(1*H*)-thione (*J. Am. Chem. Soc.* **1996**, *118,* 10113; *Photochemistry and Photobiology* **1995**, *61*, 269).

$$\text{(2-S-PyrN-O}^-\text{)}^* + \text{H}^+ \rightarrow \text{2-S-Pyr(}^{\cdot}\text{N)} + {}^{\cdot}\text{OH} \qquad (13)$$

And through photooxidation of 2-Mercaptopyridine (*Photochemistry and Photobiology* **1994**, *60*, 442).

$$^3\text{(2-S-PyrH)}^* + \text{O}_2 \rightarrow \text{2-S-Pyr(N}^{\cdot}\text{)} + \text{O}_2^{\cdot -} + \text{H}^+ \qquad (8)$$

The BDE of the C–H bond of the substrate is estimated to *83.9 kcal/mol* and the BDE of the N–H bond of the thione tautomer *87.0 kcal/mol* (*Nat Commun* **2020** *11*, 2328, *Sci Data* **2020** *7*, 244, *Digit. Discov.* **2023** *2*, 1900). Therefore, the proposed HAA generating the thione tautomer is thermodynamically favored and feasible. This data is consistent with our proposed mechanism.

The analysis of delocalized 2-mercaptopyridine radicals by EPR has been attempted but only indirect evidence was obtained upon reaction with DMPO as a trapping agent, suggesting that the photogenerated radicals are *highly reactive transient species*. (*Photochemistry and Photobiology* **1994**, *60*, 442; *Photochemistry and Photobiology* **1994**, *60*, 450; *Photochemistry and Photobiology* **1995**, *61*, 269).

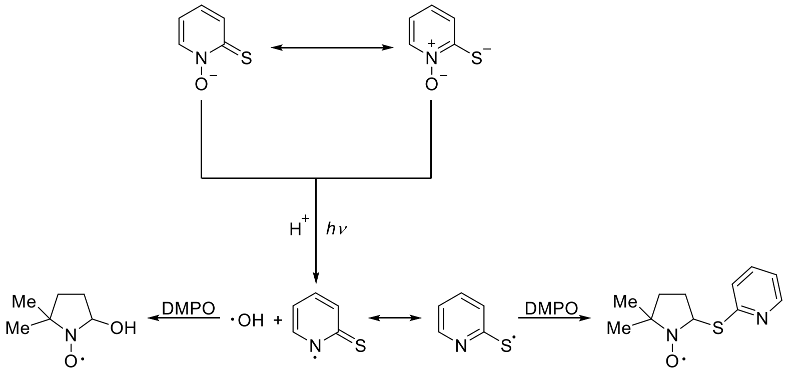

Delocalized 2-mercaptopyridine radicals can be photogenerated via N-O bond cleavage of *N*-Hydroxypyridine-2(1*H*)-thione (*Photochemistry and Photobiology* **1995**, *61*, 269) and from other related compounds (*Photochemistry and Photobiology* **1994**, *60*, 442, see eq 8). This implies radical character on the nitrogen, though only sulfur adducts are observed with DMPO trapping. This further supports the delocalized nature of this radical. This trapping of the radical at sulfur is

not representative of the catalysis given the different nature of HAT and the addition to sp^2-hybridized carbons.

Attempting to generate this radical in *the absence of substrate* is likely to lead to quenching of the radical via dimerization or other pathways (e.g. HAA with solvent) before any measurement can be conducted. In view of the high reactivity of this *transient delocalized* radical and the underlying complexity of our system (chiral assembly via non-covalent interactions), low temperature EPR will not yield a meaningful result.

Comment 3: *2) As noted by the author, HAA by the thiyl radical is also possible. However, the authors excluded this possibility based on the relative stability of the two tautomers. This argument is not supported by the Hammond postulate as HAA reactions are generally exergonic processes that involve early transition states, that is, the stability of the products has little influence on the selectivity of HAA reactions. The authors should provide more solid evidences to prove/disprove the alternative pathway—HAA by the thiyl radical and HAD by the N-H bond.*

Response 3: We agree that, in principle, hydrogen atom abstraction can occur at nitrogen or at sulfur. However, we consider the alternative pathway suggested by the reviewer highly unlikely in our study for the following reasons.
Literature precedents establish that 2-mercaptopyridines exist predominantly as the thione tautomer in the ground-state, whereas access to thiol-like reactivity is enabled upon photochemical excitation (*Struct. Dyn.* **2017**, *4*, 044021).

Accordingly, we propose a mechanism in which hydrogen atom abstraction generates the thione tautomer and hydrogen atom delivery occurs from the thiol tautomer, which can only be accessed under irradiation. Therefore, the proposed mechanism is consistent with the directionality imposed by the underlying tautomeric preference in the ground state and the photoinduced tautomerization to the thiol tautomer.
In contrast, the alternative pathway involving thiyl radical-mediated HAA followed by thione-mediated hydrogen atom delivery (from N-H bond) is less consistent with the established ground-state tautomeric bias and would importantly require additional energetic and/or kinetic steps, rendering it mechanistically less plausible within the present system.
We therefore consider that our currently proposed pathway represents the most consistent interpretation of the available data and literature precedent, while acknowledging that strict exclusion of other pathways is neither possible nor necessary.

Comment 4: *3) The authors claim that both HAA and HAD are enantioselective and the overall enantioselectivity is the synergistic effect of the two steps. However, neither selectivity is experimentally determined. Therefore, it's unknown which step is more important in stereocontrol. In the first round of review, Reviewer 2 also mentioned that the authors should "deconvolve the respective contributions of the claimed enantioselective HAA and HAD". Notably, most of the seminal works cited by the authors have experimentally determined at least one of the two selectivities, providing valuable insights for future catalyst development. This is particularly important for an unprecedented deracemization system.*

Response 4:

We have addressed this concern in the previous round of our revisions directly to reviewer #2.

We agree that deconvolution of HAA and HAD contributions would be informative. However, in

the present system, both steps are mediated by a single assembly, making direct separation of these contributions experimentally challenging. Importantly, in most of the seminal work proceeding through HAT enabled deracemization, only one HAT step is enenatioselective (either HAA or HAD). This makes the deconvolution of the contributions of HAA or HAD to the observed enantiomeric excess feasible.

Comment 5*: 4) The interpretation of the experiments using excess PySH-6 or thiophenol is still confusing (Fig. 5G and SI, P74). The authors proposed an in-cage HAA/HAD process, , yet addition of 20 mol% of PySH-6 significantly influence the er of the product. How does PySH-6 influence the in-cage, quasi-intramolecular HAD step?*

Response 5: In this context, the addition of external PySH-6 may perturb the equilibrium and stoichiometry of the catalyst assembly, thereby influencing the efficiency and selectivity of hydrogen atom transfer processes. The experiment reinforces that the external achiral HAD catalyst competes with the enantioselective HAD performed by the chiral assembly.

Comment 6: *5) In the first round of review, this reviewer suggested that several closely related works should be cited. However, none of them was cited in the revised manuscript, even the very related work where a chiral phosphate and a non-chiral aryl thiol was utilized for photochemical deracemization (CCS Chem. 2026, doi: 10.31635/ccschem.025.202506718). I understand that the authors did some experiments and claimed that their mechanism is different, but that does not mean this reference should be excluded. I am aware that this journal has some guidelines on the number of citations, but I don't think the editor would ask the authors to exclude closely related references merely to keep the citation count within a certain limit. If the authors are confident about their novelty, they should cite and discuss this work in the introduction/Fig. 2, and then highlight their own novelty via subsequent mechanistic evidences. Otherwise, the readers would be confused as to why the current work deserves publication in Nature when a very similar system has already been published. For the rest suggested references, the authors tried very hard to find various reasons to exclude them. They also explained why their 5-membered, N-CONHPh, 2-aryl pyrrolidine substrates are not limited--because electron-rich and electron-deficient as well as bulky aryl substituents are tolerated. Notably, even the supportive "Reviewer 1" mentioned that "This critique is a little too broad, and could easily be applied to this work too", indicating that the current work is also limited in scope.*

Response 6: Figure 2 in the manuscript summarizes groundbreaking work in the field of asymmetric catalysis (not in photochemical deracemizations). More specifically, the use of chiral anions that are paired with *achiral* catalysts to render those effectively chiral is discussed. As stated in the manuscript *"the underlying principles of these conceptual catalyst designs have only been suitable for transformations operating via ionic intermediates"*. Our report describes the first example in which this concept is applicable to transformations proceeding through *neutral radicals*. While first demonstrated on the deracemization of 2-aryl pyrrolidines our catalytic system is a significant breakthrough in the field and will lead to the discovery of multiple enantioselective radical transformations which is an outstanding challenge. Discussing the deracemization of enolizable tetralones *(CCS Chem. 2026, doi: 10.31635/ccschem.025.202506718)* is therefore unrelated and out of place. We have now added this citation together with the cited reviews on photochemical deracemizations (see citation 48 in the revised manuscript).

Reviewer #1 agrees that the photochemical deracemization of 2-aryl pyrrolidines is a significant advancement compared to the previous deracemizations of carbonyl-based deracemizations.

In the first round of revision reviewer #1 stated: ***"...Indeed, the concept is quite well explored, optimized, and evaluated with a range of interesting, drug like pyrrolidines being deracemized or even interconverted. The selectivity is consistently high across these examples - illustrating high robustness and utility."***

Also ***"Overall, the authors have done a thorough job describing the reaction development, mechanistic elucidation, excellent scope of reactivity, and synthetic utility."***

And **"Figure 4 - These scope studies are very well done. Excellent choices of widely ranging and useful molecules."**

In this round of revisions reviewer #1 states***"...this privileged (in med chem especially) pyrrolidine deracemization (see their response to Reviewer 4 about the difference in substrate classes now possible versus previous carbonyl-based deracemizations)"***

Comment 7: *6) To better define the scope and limitations of the current approach, the results of 2-alkyl/4/6-membered substrates should be added in the text and the SI. Currently, I only see these results in the authors' response letter.*

Response 7: Thank you for this suggestion. We agree that adding the results of 2-alkyl/4/6-membered substrates would better define the scope and limitations of our study. We have added this information to the supporting information.

Comment 8: *In summary, this reviewer remains unconvinced that the current manuscript meets the high standard expected for Nature. However, if the authors can fully address the above issues, I may reconsider my position.*

Response 8:

We thank reviewer 4 for finding our work interesting and for the overall assessment.
